# Supplementary material for: A gradient green-beard gene in fission yeast
Source: EMBO Rep. 2026 Mar 16;27(8):1904–17. doi: 10.1038/s44319-026-00748-x (PMC13121626; doi:10.1038/s44319-026-00748-x)
Supplement: Supplementary file 1 — Appendix [file 44319_2026_748_MOESM1_ESM.pdf]

Appendix

A Gradient Green-beard Gene in Fission Yeast

Zhiwei Wu and Guan-Zhu Han*

*Corresponding author. Email: guanzhu@njnu.edu.cn

List of Appendix Figures and Tables

Appendix Figures

Appendix Figure S1………………..page 2

Appendix Figure S2………………..page 3

Appendix Figure S3………………..page 4

Appendix Figure S4………………..page 5

Appendix Figure S5………………..page 6

Appendix Figure S6………………..page 7

Appendix Figure S7………………..page 8

Appendix Tables

Appendix Table S1…………………page 9

Appendix Table S2…………………page 30

Appendix Figures

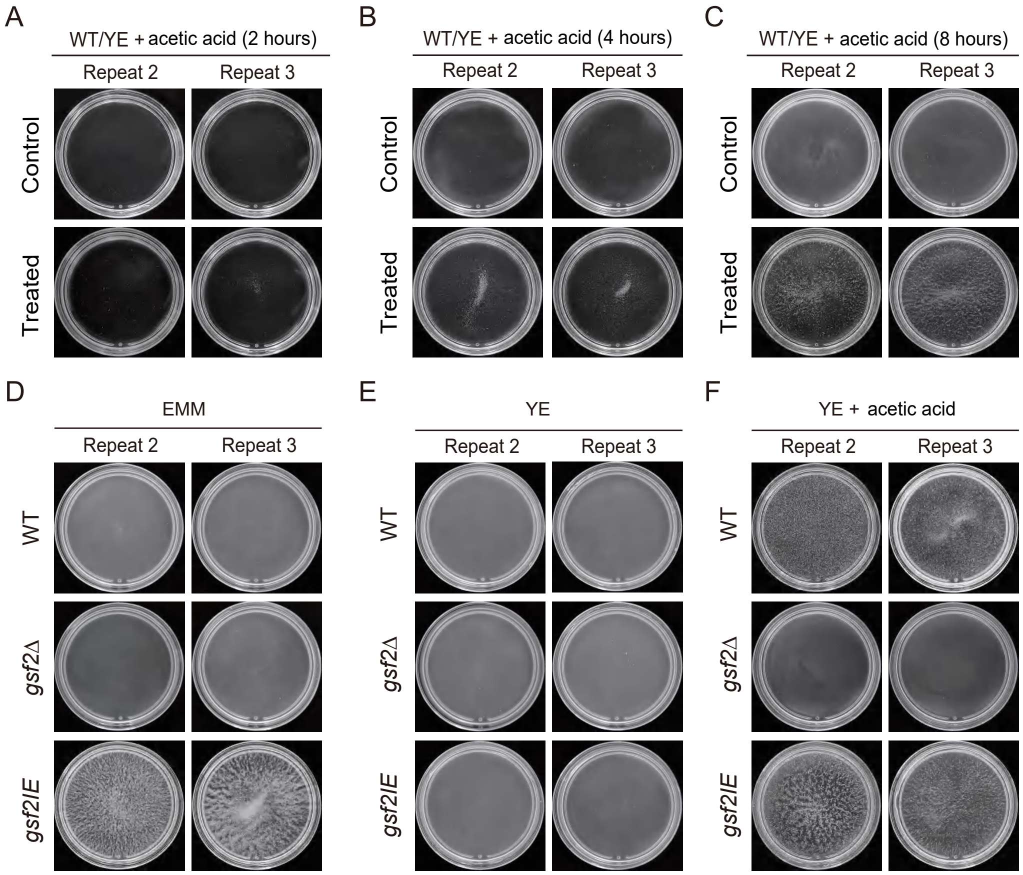

Appendix Figure S1. *gsf2* confers strong flocculation in *S. pombe*. (A), (B) and (C) The phenotypes of the WT cells after 2h, 4h, and 8h with and without 0.1% acetic acid treatment. Each experiment was performed with three independent biological replicates. (D), (E) and (F) The phenotypes of the yeast strains WT, *gsf2IE*, and *gsf2*Δ under different culture condition, including EMM media, YE media, and YE media with 0.1% acetate acid. Each experiment was performed with three independent biological replicates.

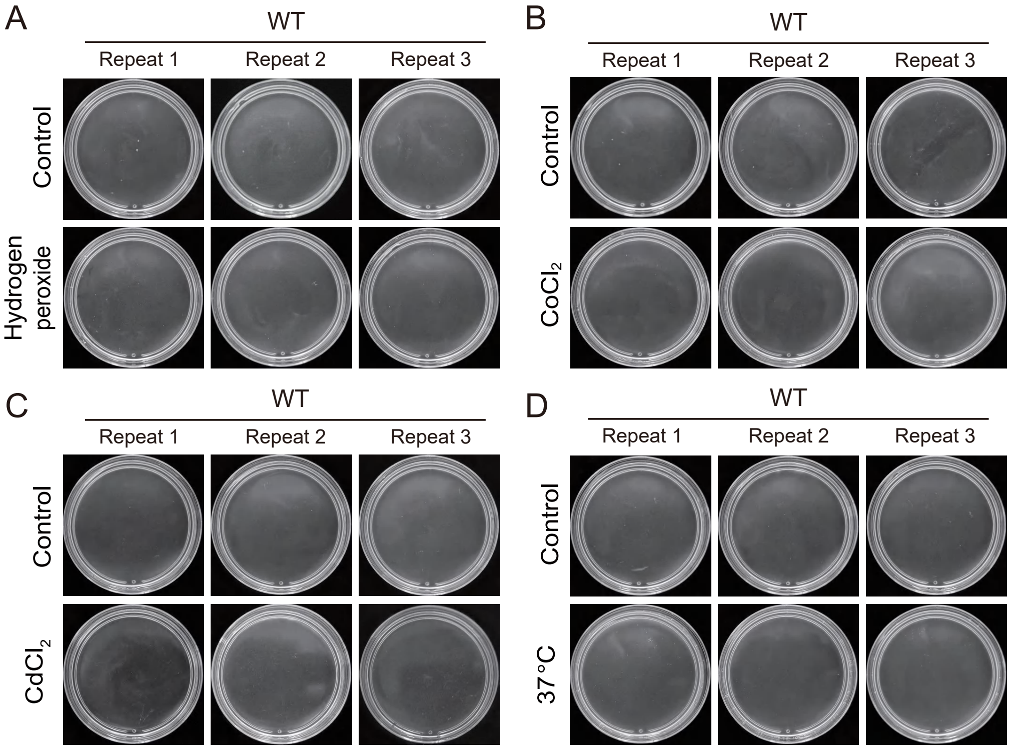

Appendix Figure S2. The phenotypes of WT cells under different stress challenges. The phenotypes of the WT cells cultured in YE media with 2.5 mM hydrogen peroxide (A), YE media with 0.3 mM $CoCl_2$ (B), YE media with 0.02 mM $CdCl_2$ (C), and cultured in 37°C (D). Each experiment was performed with three independent biological replicates.

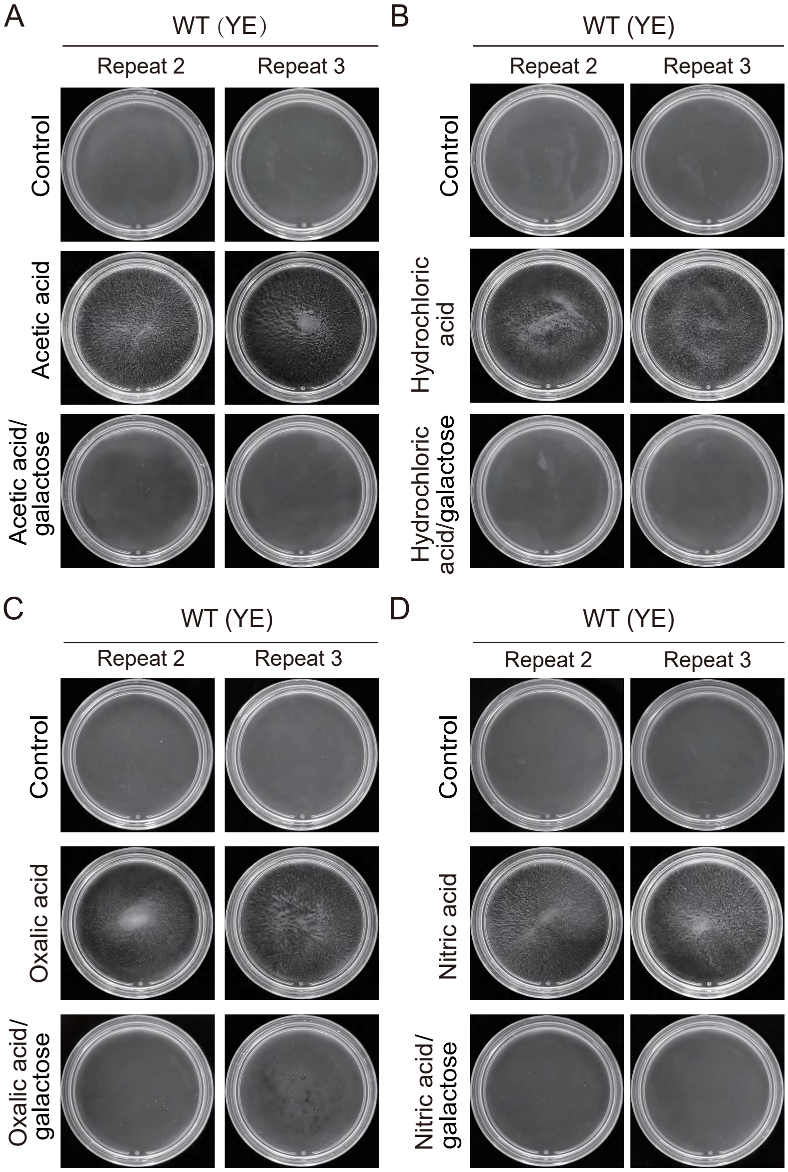

Appendix Figure S3. The phenotypes of WT cells under different stress challenges. The phenotypes of the WT cells without and with treatment with 0.1% acetic acid (A), 5 mM hydrochloric acid (B), 2.5 mM oxalic acid (C), and 4 mM nitric acid (D) and with adding acid and galactose. Each experiment was performed with three independent biological replicates.

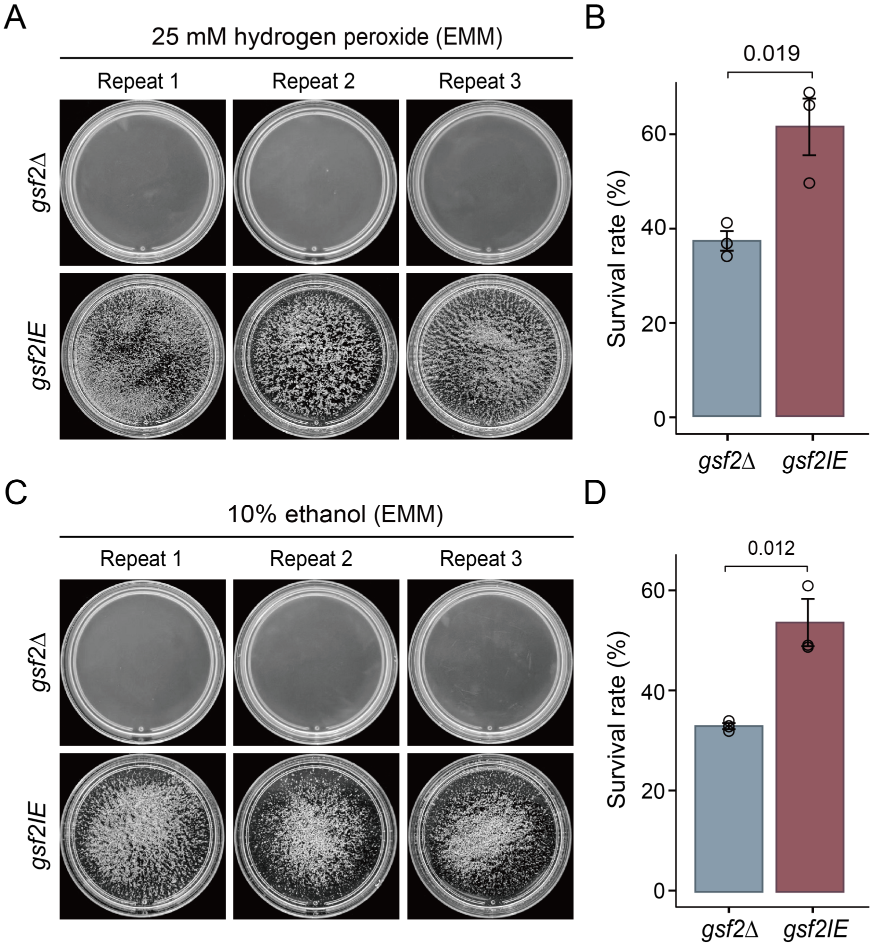

Appendix Figure S4. Survival of yeast cells under stress challenges. (A) The phenotypes of the *gsf2IE* and the *gsf2*Δ strains in EMM liquid media with 25 mM hydrogen peroxide. (B) The survival rates of the *gsf2IE* and the *gsf2*Δ strains in EMM liquid media with 25 mM hydrogen peroxide. (C) The phenotypes of the *gsf2IE* and the *gsf2*Δ strains in EMM liquid media with 10% ethanol. (D) The survival rates of the *gsf2IE* and the *gsf2*Δ strains in EMM liquid media with 10% ethanol.

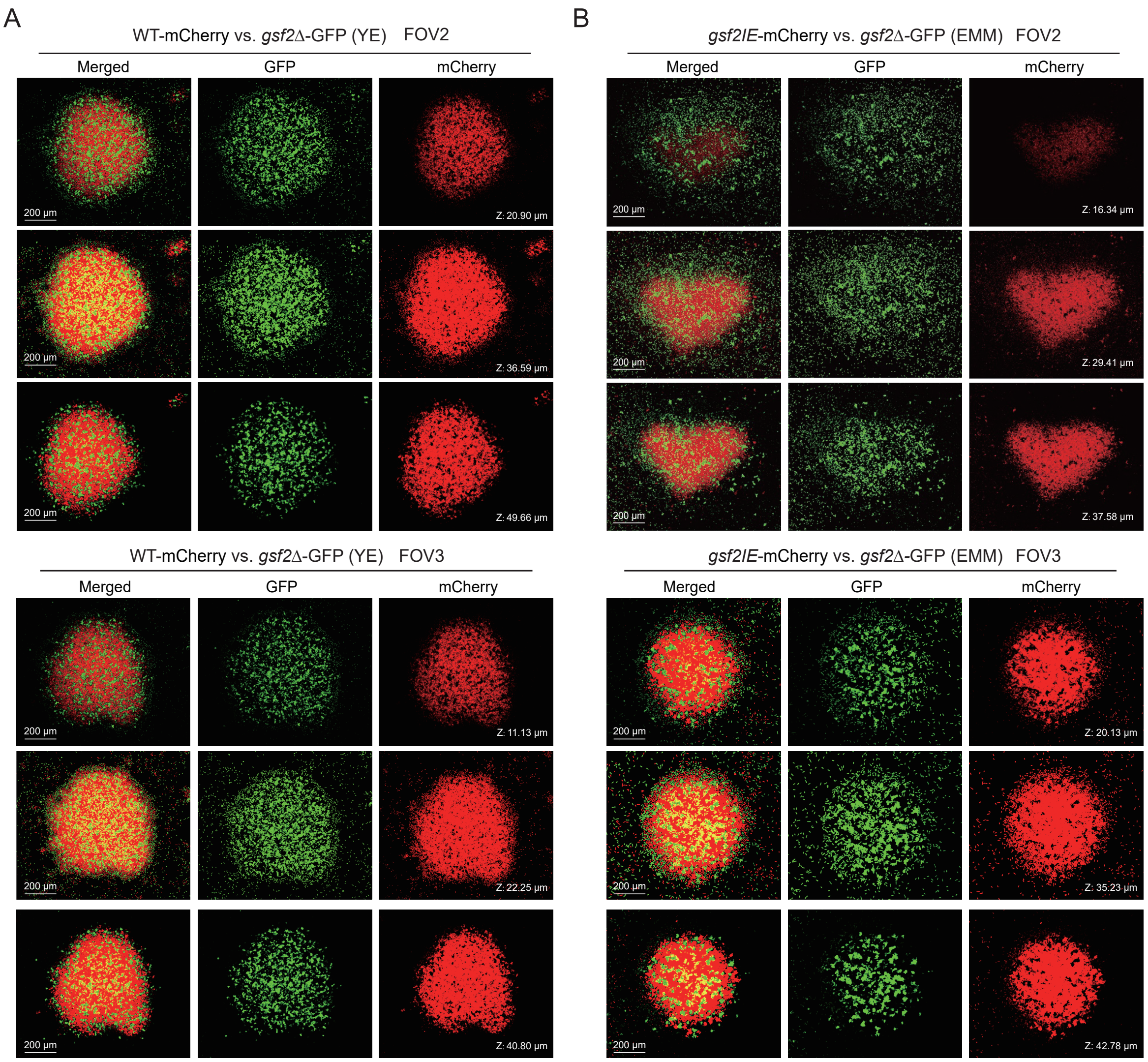

Appendix Figure S5. Spatial arrangement of the mixture of the *gsf2*-expressing and non-expressing cells in the flocculation. We performed 3D reconstruction of flocs using laser confocal microscopy, analyzing three independent fields of view (FOVs) per condition. (A) The floc was formed by an equally mixed population of WT-mCherry and *gsf2*Δ-GFP, induced with 0.1% acetic acid. (B) The floc was formed by an equally mixed population of *gsf2IE*-mCherry and *gsf2*Δ-GFP in EMM medium. The Z value represents the distance of the scanned layer to the bottom of the floc in the Z-dimension.

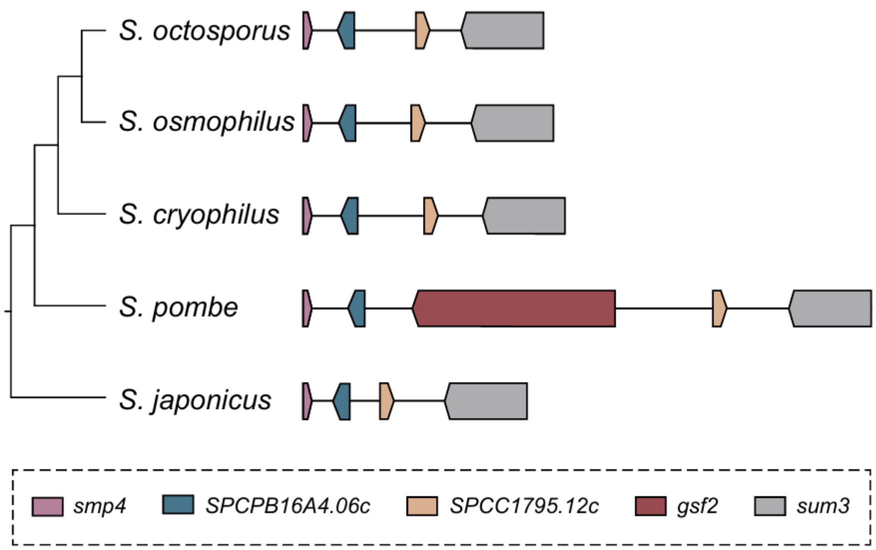

Appendix Figure S6. The synteny of *gsf2* across *Schizosaccharomyces* species. The syntenic region of the *gsf2* gene in five *Schizosaccharomyces* species is shown.

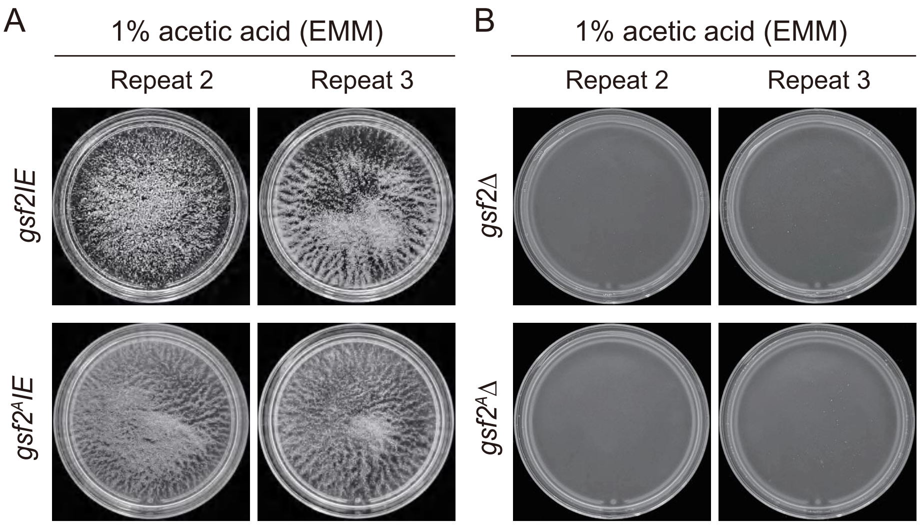

Appendix Figure S7. Gsf2 variation affects flocculation intensity. (A) The flocculation status of *gsf2*^{A}*IE* and *gsf2IE* in EMM media with 1% acetic acid. (B) The flocculation status of *gsf2*Δ and *gsf2*^{A}Δ in EMM media with 1% acetic acid.

Appendix Tables

Appendix Table S1. The Gsf2 protein sequences in globally sampled strains of *S. pombe*

Strains	Sequences
972h^{-}	MSVRRFLSTSARALLFTAALLPSLTSGLPSGNVRILQKGMEPEDYLSSASQNEVPHDISLPKTELADPNFLVDDMP<br>TLLGRDAAVDPSMFTSTFTVKNGNDANYITASPVSNDASMTAISTFTSGKEASYAIQASPSTFLPDSTTTSGSQVS<br>NAVEASSTFVADTTSTSCNPATVLIVTTSGSTSTSCPPPTTILIVTVPTTTTTTTVGYPGSVTTTLTGTPSNGTVIDTV<br>EVPTTTNYGYTTITTGYTGSTTLTTTVPHSGNETGPTTVYVETPYPTTVTTTTTVGYPGSVTTTLTGAPSNGTVIDT<br>VEVPTTTNYGYTTVTTGYTGSTTLTTTVPHSGNETGPTTVYVETPYPTTVTTTTTVGYPGSVTTTLTGAPSNGTVI<br>DTVEVPTTTNYGYTTVTTGYTGSTTLTTTVPHSGNETGPTTVYVETPYPTTVTTTTTVGYPGSVTTTLTGAPSNGT<br>VIDTVEIPTTTNYGYTTITTGYTGSTTLTTTVPHSGNETGPTTVYVETPYPTTVTTTTTVGYPGSVTTTLTGAPSNG<br>TVIDTVEVPTTTNYGYTTITTGYTGSTTLTTTVPHSGNETGPTTVYVETPYPTTVTTTTTVGYPGSVTTTLTGAPSN<br>GTVIDTVEVPTTTNYGYTTVTTGYTGSTTLTTTVPHSGNETGPTTVYVETPYPTTVTTTTTVGYSGSVTTTLTGSG<br>SNSIVTETVDVPTTTSVNYGYTTITTGWTGSTTLTSIVTHSGSETGPTTVYIETPSVSATTTTTTIGYSGSLTTTLTGS<br>SGPVVTNTVEIPYGNSSYIIPTTIVTGTVTTVTTGYTGTETSTVTVIPTGTTGTTTVVIQTPTTVTATETDIVTVTTGY<br>TGTETSTVTVTPTGTSTGTTTVVIQTPTTVTATETDIVTVTTGYTGTETSTVTVTPTGTSTGTTTVVIQTPTTVTATE<br>TDIVTVTTGYTGTETSTVTVTPTGTSTGTTTVVIQTPTTVTATETDIVTVTTGYTGTETSTVTVTPTGTSTGTTTVVI<br>QTPTTVTATETDIVTVTTGYTGTETSTVTVTPTGTSTGTTTVVIQTPTTVTATETDIVTVTTGYTGTETSTVTVTPT<br>GTSTGTTTVVIQTPTTVTATETDIVTVTTGYTGTETSTVTVTPTGTATGTTTVVINTPTTTGSEVLPTTGATGTAGT<br>ETQLTTATEVQPTTGATGTAGTETQVTTGTETQATTATETQATTATEVQTTTGATGTAGTETQATTATEVQPTTG<br>ATGTAGTETQVTTATEVQPTTGATGTAGTETQVTTGTETQATTATETQATTATEVQTTTGATGTAGTETQATTA<br>TEVQPTTGATGTAGTETQVTTATEVQPTTGATGTAGTETQVTTGTETQATTATETQATTATEVQTTTGATGTAG<br>TETQVTTATEVQPTTAVTETSSSGYYTTIVSSTVVSTVVPGSTVYPVTHVTTTTGVSGESSAFTYTTSSTQYEPSTV<br>VTTSYYTTSVYTSAPATETVSSTEAPESSTVTSNPIYQGSGTSTWSTVRQWNGSATYNYTYYTTGGFTGGNNTNV<br>TGLYPSSAGANKPIAYLTFVSLFVYIVTLI
WT[A]	MSVRRFLSTSARALLFTAALLPSLTSGLPSGNVRILQKGMEPEDYLSSASQNEVPHDISLPKTELADPNFLVDDMPTLLG<br>RDAAVDPSMFTSTFTVKNGNDANYITASPVSNDASMTAISTFTSGKEASYAIQASPSTFLPDSTTTSGSQVSNAVEASST<br>FVADTTSTSCNPATVLIVTTSGSTSTSCPPPTTILIVTVPTTTTTTTVGYPGSVTTTLTGTPSNGTVIDTVEVPTTTNYGYT<br>TITTGYTGSTTLTTTVPHSGNETGPTTVYVETPYPTTVTTTTTVGYPGSVTTTLTGAPSNGTVIDTVEVPTTTNYGYTTV<br>TTGYTGSTTLTTTVPHSGNETGPTTVYVETPYPTTVTTTTTVGYPGSVTTTLTGAPSNGTVIDTVEVPTTTNYGYTTITT<br>GYTGSTTLTTTVPHSGNETGPTTVYVETPYPTTVTTTTTVGYPGSVTTTLTGAPSNGTVIDTVEVPTTTNYGYTTVTTG<br>YTGSTTLTTTVPHSGNETGPTTVYVETPYPTTVTTTTTVGYPGSVTTTLTGAPSNGTVIDTVEVPTTTNYGYTTVTTGYT<br>GSTTLTTTVPHSGNETGPTTVYVETPYPTTVTTTTTVGYPGSVTTTLTGTPSNGTVIDTVEVPTTTNYGYTTITTGYTGST<br>TLTTTVPHSGNETGPTTVYVETPYPTTVTTTTTVGYPGSVTTTLTGAPSNGTVIDTVEVPTTTNYGYTTVTTGYTGSTTL<br>TTTVPHSGNETGPTTVYVETPYPTTVTTTTTVGYPGSVTTTLTGAPSNGTVIDTVEVPTTTNYGYTTVTTGYTGSTTLTT

TVPHSGNETGPTTVYVETPYPTTVTTTTTVGYPGSVTTTLTGAPSNGTVIDTVEVPTTTNYGYTTVTTGYTGSTTLTTTV
PHSGNETGPTTVYVETPYPTTVTTTTTVGYPGSVTTTLTGAPSNGTVIDTVEVPTTTNYGYTTITTGYTGSTTLTTTVPH
SGNETGPTTVYVETPYPTTVTTTTTVGYPGSVTTTLTGAPSNGTVIDTVEVPTTTNYGYTTVTTGYTGSTTLTTTVPHSG
NETGPTTVYVETPYPTTVTTTTTVGYPGSVTTTLTGAPSNGTVIDTVEVPTTTNYGYTTVTTGYTGSTTLTTTVPHSGNE
TGPTTVYVETPYPTTVTTTTTVGYPGSVTTTLTGAPSNGTVIDTVEVPTTTNYGYTTVTTGYTGSTTLTTTVPHSGNETG
PTTVYVETPYPTTVTTTTTVGYPGSVTTTLTGAPSNGTVIDTVEVPTTTNYGYTTITTGYTGSTTLTTTVPHSGNETGPTT
VYVETPYPTTVTTTTTVGYPGSVTTTLTGAPSNGTVIDTVEVPTTTNYGYTTVTTGYTGSTTLTTTVPHSGNETGPTTV
YVETPYPTTVTTTTTVGYPGSVTTTLTGAPSNGTVIDTVEVPTTTNYGYTTVTTGYTGSTTLTTTVPHSGNETGPTTVY
VETPYPTTVTTTTTVGYPGSVTTTLTGAPSNGTVIDTVEVPTTTNYGYTTVTTGYTGSTTLTTTVPHSGNETGPTTVYVE
TPYPTTVTTTTTVGYPGSVTTTLTGAPSNGTVIDTVEVPTTTNYGYTTITTGYTGSTTLTTTVPHSGNETGPTTVYVETP
YPTTVTTTTTVGYPGSVTTTLTGAPSNGTVIDTVEVPTTTNYGYTTVTTGYTGSTTLTTTVPHSGNETGPTTVYVETPYP
TTVTTTTTVGYPGSVTTTLTGAPSNGTVIDTVEVPTTTNYGYTTVTTGYTGSTTLTTTVPHSGNETGPTTVYVETPYPTT
VTTTTTVGYPGSVTTTLTGTPSNGTVIDTVEVPTTTNYGYTTITTGYTGSTTLTTTVPHSGNETGPTTVYVETPYPTTVT
TTTTVGYPGSVTTTLTGAPSNGTVIDTVEVPTTTNYGYTTVTTGYTGSTTLTTTVPHSGNETGPTTVYVETPYPTTVTTT
TTVGYPGSVTTTLTGAPSNGTVIDTVEVPTTTNYGYTTVTTGYTGSTTLTTTVPHSGNETGPTTVYVETPYPTTVTTTTT
VGYPGSVTTTLTGAPSNGTVIDTVEVPTTTNYGYTTVTTGYTGSTTLTTTVPHSGNETGPTTVYVETPYPTTVTTTTTV
GYPGSVTTTLTGAPSNGTVIDTVEVPTTTNYGYTTITTGYTGSTTLTTTVPHSGNETGPTTVYVETPYPTTVTTTTTVGY
PGSVTTTLTGAPSNGTVIDTVEVPTTTNYGYTTVTTGYTGSTTLTTTVPHSGNETGPTTVYVETPYPTTVTTTTTVGYPG
SVTTTLTGAPSNGTVIDTVEVPTTTNYGYTTVTTGYTGSTTLTTTVPHSGNETGPTTVYVETPYPTTVTTTTTVGYPGSV
TTTLTGAPSNGTVIDTVEVPTTTNYGYTTVTTGYTGSTTLTTTVPHSGNETGPTTVYVETPYPTTVTTTTTVGYPGSVTT
TLTGAPSNGTVIDTVEVPTTTNYGYTTITTGYTGSTTLTTTVPHSGNETGPTTVYVETPYPTTVTTTTTVGYPGSVTTTL
TGAPSNGTVIDTVEVPTTTNYGYTTVTTGYTGSTTLTTTVPHSGNETGPTTVYVETPYPTTVTTTTTVGYPGSVTTTLTG
APSNGTVIDTVEIPTTTNYGYTTITTGYTGSTTLTTTVPHSGNETGPTTVYVETPYPTTVTTTTTVGYPGSVTTTLTGAPS
NGTVIDTVEVPTTTNYGYTTITTGYTGSTTLTTTVPHSGNETGPTTVYVETPYPTTVTTTTTVGYPGSVTTTLTGAPSNG
TVIDTVEVPTTTNYGYTTVTTGYTGSTTLTTTVPHSGNETGPTTVYVETPYPTTVTTTTTVGYPGSVTTTLTGAPSNGTV
IDTVEVPTTTNYGYTTVTTGYTGSTTLTTTVPHSGNETGPTTVYVETPYPTTVTTTTTVGYSGSVTTTLTGSGSNSIVTET
VDVPTTTSVNYGYTTITTGWTGSTTLTSIVTHSGSETGPTTVYIETPSVSATTTTTTIGYSGSLTTTLTGSSGPVVTNTVEI
PYGNSSYIIPTTIVTGTVTTVTTGYTGTETSTVTVIPTGTTGTTTVVIQTPTTVTATETDIVTVTTGYTGTETSTVTVTPTG
TSTGTTTVVIQTPTTVTATETDIVTVTTGYTGTETSTVTVTPTGTSTGTTTVVIQTPTTVTATETDIVTVTTGYTGTETST
VTVTPTGTSTGTTTVVIQTPTTVTATETDIVTVTTGYTGTETSTVTVTPTGTSTGTTTVVIQTPTTVTATETDIVTVTTGY
TGTETSTVTVTPTGTSTGTTTVVIQTPTTVTATETDIVTVTTGYTGTETSTVTVTPTGTSTGTTTVVIQTPTTVTATETDIV
TVTTGYTGTETSTVTVTPTGTATGTTTVVINTPTTTGSEVLPTTGATGTAGTETQLTTATEVQPTTGATGTAGTETQVTT
GTETQATTATETQATTATEVQTTTGATGTAGTETQATTATEVQPTTGATGTAGTETQVTTATEVQPTTGATGTAGTET
QVTTGTETQATTATETQATTATEVQTTTGATGTAGTETQATTATEVQPTTGATGTAGTETQVTTATEVQPTTGATGTA
GTETQVTTGTETQATTATETQATTATEVQTTTGATGTAGTETQVTTATEVQPTTAVTETSSSGYYTTIVSSTVVSTVVP
GSTVYPVTHVTTTTGVSGESSAFTYTTSSTQYEPSTVVTTSYYTTSVYTSAPATETVSSTEAPESSTVTSNPIYQGSGTST
WSTVRQWNGSATYNYTYYTTGGFTGGNNTNVTGLYPSSAGANKPIAYLTFVSLFVYIVTLI

WT	MSVRRFLSTSARALLFTAALLPSLTSGLPSGNVRILQKGMEPEDYLSSASQNEVPHDISLPKTELADPNFLVDDMPTLLG<br>RDAAVDPSMFTSTFTVKNGNDANYITASPVSNDASMTAISTFTSGKEASYAIQASPSTFLPDSTTTSGSQVSNAVEASST<br>FVADTTSTSCNPATVLIVTTSGSTSTSCPPPTTILIVTVPTTTTTTTVGYPGSVTTTLTGTPSNGTVIDTVEVPTTTNYGYT<br>TITTGYTGSTTLTTTVPHSGNETGPTTVYVETPYPTTVTTTTTVGYPGSVTTTLTGAPSNGTVIDTVEVPTTTNYGYTTV<br>TTGYTGSTTLTTTVPHSGNETGPTTVYVETPYPTTVTTTTTVGYPGSVTTTLTGAPSNGTVIDTVEVPTTTNYGYTTVTT<br>GYTGSTTLTTTVPHSGNETGPTTVYVETPYPTTVTTTTTVGYPGSVTTTLTGAPSNGTVIDTVEVPTTTNYGYTTVTTG<br>YTGSTTLTTTVPHSGNETGPTTVYVETPYPTTVTTTTTVGYPGSVTTTLTGAPSNGTVIDTVEVPTTTNYGYTTITTGYT<br>GSTTLTTTVPHSGNETGPTTVYVETPYPTTVTTTTTVGYPGSVTTTLTGAPSNGTVIDTVEVPTTTNYGYTTVTTGYTGS<br>TTLTTTVPHSGNETGPTTVYVETPYPTTVTTTTTVGYPGSVTTTLTGAPSNGTVIDTVEVPTTTNYGYTTVTTGYTGSTT<br>LTTTVPHSGNETGPTTVYVETPYPTTVTTTTTVGYPGSVTTTLTGAPSNGTVIDTVEVPTTTNYGYTTVTTGYTGSTTLT<br>TTVPHSGNETGPTTVYVETPYPTTVTTTTTVGYPGSVTTTLTGAPSNGTVIDTVEVPTTTNYGYTTITTGYTGSTTLTTT<br>VPHSGNETGPTTVYVETPYPTTVTTTTTVGYPGSVTTTLTGAPSNGTVIDTVEVPTTTNYGYTTVTTGYTGSTTLTTTVP<br>HSGNETGPTTVYVETPYPTTVTTTTTVGYPGSVTTTLTGAPSNGTVIDTVEIPTTTNYGYTTITTGYTGSTTLTTTVPHSG<br>NETGPTTVYVETPYPTTVTTTTTVGYPGSVTTTLTGAPSNGTVIDTVEVPTTTNYGYTTITTGYTGSTTLTTTVPHSGNE<br>TGPTTVYVETPYPTTVTTTTTVGYPGSVTTTLTGAPSNGTVIDTVEVPTTTNYGYTTVTTGYTGSTTLTTTVPHSGNETG<br>PTTVYVETPYPTTVTTTTTVGYPGSVTTTLTGAPSNGTVIDTVEVPTTTNYGYTTVTTGYTGSTTLTTTVPHSGNETGPT<br>TVYVETPYPTTVTTTTTVGYSGSVTTTLTGSGSNSIVTETVDVPTTTSVNYGYTTITTGWTGSTTLTSIVTHSGSETGPTT<br>VYIETPSVSATTTTTTIGYSGSLTTTLTGSSGPVVTNTVEIPYGNSSYIIPTTIVTGTVTTVTTGYTGTETSTVTVIPTGTTG<br>TTTVVIQTPTTVTATETDIVTVTTGYTGTETSTVTVTPTGTSTGTTTVVIQTPTTVTATETDIVTVTTGYTGTETSTVTVT<br>PTGTSTGTTTVVIQTPTTVTATETDIVTVTTGYTGTETSTVTVTPTGTSTGTTTVVIQTPTTVTATETDIVTVTTGYTGTE<br>TSTVTVTPTGTSTGTTTVVIQTPTTVTATETDIVTVTTGYTGTETSTVTVTPTGTSTGTTTVVIQTPTTVTATETDIVTVTT<br>GYTGTETSTVTVTPTGTSTGTTTVVIQTPTTVTATETDIVTVTTGYTGTETSTVTVTPTGTATGTTTVVINTPTTTGSEVL<br>PTTGATGTAGTETQLTTATEVQPTTGATGTAGTETQVTTGTETQATTATETQATTATEVQTTTGATGTAGTETQATTA<br>TEVQPTTGATGTAGTETQVTTATEVQPTTGATGTAGTETQVTTGTETQATTATETQATTATEVQTTTGATGTAGTETQ<br>ATTATEVQPTTGATGTAGTETQVTTATEVQPTTGATGTAGTETQVTTGTETQATTATETQATTATEVQTTTGATGTAG<br>TETQVTTATEVQPTTAVTETSSSGYYTTIVSSTVVSTVVPGSTVYPVTHVTTTTGVSGESSAFTYTTSSTQYEPSTVVTTS<br>YYTTSVYTSAPATETVSSTEAPESSTVTSNPIYQGSGTSTWSTVRQWNGSATYNYTYYTTGGFTGGNNTNVTGLYPSSA<br>GANKPIAYLTFVSLFVYIVTLI
EBC7	MSVRRFLSTSARALLFTAALLPSLTSGLPSGNVRILQKGMEPEDYLSSASQNEVPHDISLPKTELADPNFLVDDMP<br>TLLGRDAAVDPSMFTSTFTVKNGNDANYITASPVSNDASMTAISTFTSGKEASYAIQASPSTFLPDSTTTSGSQVS<br>NAVEASSTFVADTTSTSCNPATVLIVTTSGSTSTSCPPPTTILIVTVPTTTTTTTVGYPGSVTTTLTGTPSNGTVIDTV<br>EVPTTTNYGYTTITTGYTGSTTLTTTVPHSGNETGPTTVYVETPYPTTVTTTTTVGYPGSVTTTLTGAPSNGTVIDT<br>VEVPTTTNYGYTTVTTGYTGSTTLTTTVPHSGNETGPTTVYVETPYPTTVTTTTTVGYPGSVTTTLTGAPSNGTVI<br>DTVEVPTTTNYGYTTVTTGYTGSTTLTTTVPHSGNETGPTTVYVETPYPTTVTTTTTVGYPGSVTTTLTGAPSNGT<br>VIDTVEVPTTTNYGYTTVTTGYTGSTTLTTTVPHSGNETGPTTVYVETPYPTTVTTTTTVGYPGSVTTTLTGAPSN<br>GTVIDTVEVPTTTNYGYTTITTGYTGSTTLTTTVPHSGNETGPTTVYVETPYPTTVTTTTTVGYPGSVTTTLTGAPS<br>NGTVIDTVEVPTTTNYGYTTVTTGYTGSTTLTTTVPHSGNETGPTTVYVETPYPTTVTTTTTVGYPGSVTTTLTGA<br>PSNGTVIDTVEVPTTTNYGYTTVTTGYTGSTTLTTTVPHSGNETGPTTVYVETPYPTTVTTTTTVGYPGSVTTTLT

	GTPSNGTVIDTVEVPTTTNYGYTTITTGYTGSTTLTTTVPHSGNETGPTTVYVETPYPTTVTTTTTVGYPGSVTTTL<br>TGAPSNGTVIDTVEVPTTTNYGYTTVTTGYTGSTTLTTTVPHSGNETGPTTVYVETPYPTTVTTTTTVGYPGSVTT<br>TLTGAPSNGTVIDTVEVPTTTNYGYTTVTTGYTGSTTLTTTVPHSGNETGPTTVYVETPYPTTVTTTTTVGYPGSV<br>TTTLTGAPSNGTVIDTVEVPTTTNYGYTTVTTGYTGSTTLTTTVPHSGNETGPTTVYVETPYPTTVTTTTTVGYPG<br>SVTTTLTGAPSNGTVIDTVEVPTTTNYGYTTITTGYTGSTTLTTTVPHSGNETGPTTVYVETPYPTTVTTTTTVGYP<br>GSVTTTLTGAPSNGTVIDTVEVPTTTNYGYTTVTTGYTGSTTLTTTVPHSGNETGPTTVYVETPYPTTVTTTTTVG<br>YPGSVTTTLTGAPSNGTVIDTVEVPTTTNYGYTTVTTGYTGSTTLTTTVPHSGNETGPTTVYVETPYPTTVTTTTT<br>VGYPGSVTTTLTGAPSNGTVIDTVEVPTTTNYGYTTVTTGYTGSTTLTTTVPHSGNETGPTTVYVETPYPTTVTTT<br>TTVGYPGSVTTTLTGAPSNGTVIDTVEVPTTTNYGYTTITTGYTGSTTLTTTVPHSGNETGPTTVYVETPYPTTVTT<br>TTTVGYPGSVTTTLTGAPSNGTVIDTVEVPTTTNYGYTTVTTGYTGSTTLTTTVPHSGNETGPTTVYVETPYPTTV<br>TTTTTVGYPGSVTTTLTGAPSNGTVIDTVEIPTTTNYGYTTITTGYTGSTTLTTTVPHSGNETGPTTVYVETPYPTT<br>VTTTTTVGYPGSVTTTLTGAPSNGTVIDTVEVPTTTNYGYTTITTGYTGSTTLTTTVPHSGNETGPTTVYVETPYPT<br>TVTTTTTVGYPGSVTTTLTGAPSNGTVIDTVEVPTTTNYGYTTVTTGYTGSTTLTTTVPHSGNETGPTTVYVETPY<br>PTTVTTTTTVGYPGSVTTTLTGAPSNGTVIDTVEVPTTTNYGYTTVTTGYTGSTTLTTTVPHSGNETGPTTVYVET<br>PYPTTVTTTTTVGYSGSVTTTLTGSGSNSIVTETVDVPTTTSVNYGYTTITTGWTGSTTLTSIVTHSGSETGPTTVYI<br>ETPSVSATTTTTTIGYSGSLTTTLTGSSGPVVTNTVEIPYGNSSYIIPTTIVTGTVTTVTTGYTGTETSTVTVIPTGTT<br>GTTTVVIQTPTTVTATETDIVTVTTGYTGTETSTVTVTPTGTSTGTTTVVIQTPTTVTATETDIVTVTTGYTGTETST<br>VTVTPTGTSTGTTTVVIQTPTTVTATETDIVTVTTGYTGTETSTVTVTPTGTSTGTTTVVIQTPTTVTATETDIVTVT<br>TGYTGTETSTVTVTPTGTSTGTTTVVIQTPTTVTATETDIVTVTTGYTGTETSTVTVTPTGTSTGTTTVVIQTPTTVT<br>ATETDIVTVTTGYTGTETSTVTVTPTGTSTGTTTVVIQTPTTVTATETDIVTVTTGYTGTETSTVTVTPTGTATGTT<br>TVVINTPTTTGSEVLPTTGATGTAGTETQLTTATEVQPTTGATGTAGTETQVTTGTETQATTATETQATTATEVQT<br>TTGATGTAGTETQATTATEVQPTTGATGTAGTETQVTTATEVQPTTGATGTAGTETQVTTGTETQATTATETQA<br>TTATEVQTTTGATGTAGTETQATTATEVQPTTGATGTAGTETQVTTATEVQPTTGATGTAGTETQVTTGTETQA<br>TTATETQATTATEVQTTTGATGTAGTETQVTTATEVQPTTAVTETSSSGYYTTIVSSTVVSTVVPGSTVYPVTHVT<br>TTTGVSGESSAFTYTTSSTQYEPSTVVTTSYYTTSVYTSAPATETVSSTEAPESSTVTSNPIYQGSGTSTWSTVRQW<br>NGSATYNYTYYTTGGFTGGNNTNVTGLYPSSAGANKPIAYLTFVSLFVYIVTLI
FL0	MSVRRFLSTSARALLFTAALLPSLTSGLPSGNVRILQKGMEPEDYLSSASQNEVPHDISLPKTELADPNFLVDDMP<br>TLLGRDAAVDPSMFTSTFTVKNGNDANYITASPVSNDASMTAISTFTSGKEASYAIQASPSTFLPDSTTTSGSQVS<br>NAVEASSTFVADTTSTSCNPATVLIVTTSGSTSTSCPPPTTILIVTVPTTTTTTTVGYPGSVTTTLTGTPSNGTVIDTV<br>EVPTTTNYGYTTITTGYTGSTTLTTTVPHSGNETGPTTVYVETPYPTTVTTTTTVGYPGSVTTTLTGAPSNGTVIDT<br>VEVPTTTNYGYTTVTTGYTGSTTLTTTVPHSGNETGPTTVYVETPYPTTVTTTTTVGYPGSVTTTLTGAPSNGTVI<br>DTVEVPTTTNYGYTTITTGYTGSTTLTTTVPHSGNETGPTTVYVETPYPTTVTTTTTVGYPGSVTTTLTGAPSNGT<br>VIDTVEVPTTTNYGYTTVTTGYTGSTTLTTTVPHSGNETGPTTVYVETPYPTTVTTTTTVGYPGSVTTTLTGAPSN<br>GTVIDTVEVPTTTNYGYTTVTTGYTGSTTLTTTVPHSGNETGPTTVYVETPYPTTVTTTTTVGYPGSVTTTLTGTP<br>SNGTVIDTVEVPTTTNYGYTTITTGYTGSTTLTTTVPHSGNETGPTTVYVETPYPTTVTTTTTVGYPGSVTTTLTGA<br>PSNGTVIDTVEVPTTTNYGYTTVTTGYTGSTTLTTTVPHSGNETGPTTVYVETPYPTTVTTTTTVGYPGSVTTTLT

GAPSNGTVIDTVEVPTTTNYGYTTVTTGYTGSTTLTTTVPHSGNETGPTTVYVETPYPTTVTTTTTVGYPGSVTTT
LTGAPSNGTVIDTVEVPTTTNYGYTTVTTGYTGSTTLTTTVPHSGNETGPTTVYVETPYPTTVTTTTTVGYPGSVT
TTLTGAPSNGTVIDTVEVPTTTNYGYTTITTGYTGSTTLTTTVPHSGNETGPTTVYVETPYPTTVTTTTTVGYPGSV
TTTLTGAPSNGTVIDTVEVPTTTNYGYTTVTTGYTGSTTLTTTVPHSGNETGPTTVYVETPYPTTVTTTTTVGYPG
SVTTTLTGAPSNGTVIDTVEVPTTTNYGYTTVTTGYTGSTTLTTTVPHSGNETGPTTVYVETPYPTTVTTTTTVGY
PGSVTTTLTGAPSNGTVIDTVEVPTTTNYGYTTVTTGYTGSTTLTTTVPHSGNETGPTTVYVETPYPTTVTTTTTV
GYPGSVTTTLTGAPSNGTVIDTVEVPTTTNYGYTTITTGYTGSTTLTTTVPHSGNETGPTTVYVETPYPTTVTTTTT
VGYPGSVTTTLTGAPSNGTVIDTVEVPTTTNYGYTTVTTGYTGSTTLTTTVPHSGNETGPTTVYVETPYPTTVTTT
TTVGYPGSVTTTLTGAPSNGTVIDTVEVPTTTNYGYTTVTTGYTGSTTLTTTVPHSGNETGPTTVYVETPYPTTVT
TTTTVGYPGSVTTTLTGAPSNGTVIDTVEVPTTTNYGYTTVTTGYTGSTTLTTTVPHSGNETGPTTVYVETPYPTT
VTTTTTVGYPGSVTTTLTGAPSNGTVIDTVEVPTTTNYGYTTITTGYTGSTTLTTTVPHSGNETGPTTVYVETPYPT
TVTTTTTVGYPGSVTTTLTGAPSNGTVIDTVEVPTTTNYGYTTVTTGYTGSTTLTTTVPHSGNETGPTTVYVETPY
PTTVTTTTTVGYPGSVTTTLTGAPSNGTVIDTVEVPTTTNYGYTTVTTGYTGSTTLTTTVPHSGNETGPTTVYVET
PYPTTVTTTTTVGYPGSVTTTLTGTPSNGTVIDTVEVPTTTNYGYTTITTGYTGSTTLTTTVPHSGNETGPTTVYVE
TPYPTTVTTTTTVGYPGSVTTTLTGAPSNGTVIDTVEVPTTTNYGYTTVTTGYTGSTTLTTTVPHSGNETGPTTVY
VETPYPTTVTTTTTVGYPGSVTTTLTGAPSNGTVIDTVEVPTTTNYGYTTVTTGYTGSTTLTTTVPHSGNETGPTT
VYVETPYPTTVTTTTTVGYPGSVTTTLTGAPSNGTVIDTVEVPTTTNYGYTTVTTGYTGSTTLTTTVPHSGNETGP
TTVYVETPYPTTVTTTTTVGYPGSVTTTLTGAPSNGTVIDTVEVPTTTNYGYTTITTGYTGSTTLTTTVPHSGNETG
PTTVYVETPYPTTVTTTTTVGYPGSVTTTLTGAPSNGTVIDTVEVPTTTNYGYTTVTTGYTGSTTLTTTVPHSGNE
TGPTTVYVETPYPTTVTTTTTVGYPGSVTTTLTGAPSNGTVIDTVEVPTTTNYGYTTVTTGYTGSTTLTTTVPHSG
NETGPTTVYVETPYPTTVTTTTTVGYPGSVTTTLTGAPSNGTVIDTVEVPTTTNYGYTTVTTGYTGSTTLTTTVPH
SGNETGPTTVYVETPYPTTVTTTTTVGYPGSVTTTLTGAPSNGTVIDTVEVPTTTNYGYTTITTGYTGSTTLTTTVP
HSGNETGPTTVYVETPYPTTVTTTTTVGYPGSVTTTLTGAPSNGTVIDTVEVPTTTNYGYTTVTTGYTGSTTLTTT
VPHSGNETGPTTVYVETPYPTTVTTTTTVGYPGSVTTTLTGAPSNGTVIDTVEIPTTTNYGYTTITTGYTGSTTLTT
TVPHSGNETGPTTVYVETPYPTTVTTTTTVGYPGSVTTTLTGAPSNGTVIDTVEVPTTTNYGYTTVTTGYTGSTTL
TTTVPHSGNETGPTTVYVETPYPTTVTTTTTVGYPGSVTTTLTGAPSNGTVIDTVEVPTTTNYGYTTVTTGYTGST
TLTTTVPHSGNETGPTTVYVETPYPTTVTTTTTVGYPGSVTTTLTGAPSNGTVIDTVEVPTTTNYGYTTITTGYTGS
TTLTTTVPHSGNETGPTTVYVETPYPTTVTTTTTVGYPGSVTTTLTGAPSNGTVIDTVEVPTTTNYGYTTVTTGYT
GSTTLTTTVPHSGNETGPTTVYVETPYPTTVTTTTTVGYSGSVTTTLTGSGSNSIVTETVDVPTTTSVNYGYTTITT
GWTGSTTLTSIVTHSGSETGPTTVYIETPSVSATTTTTTIGYSGSLTTTLTGSSGPVVTNTVEIPYGNSSYIIPTTIVTG
TVTTVTTGYTGTETSTVTVIPTGTTGTTTVVIQTPTTVTATETDIVTVTTGYTGTETSTVTVTPTGTSTGTTTVVIQT
PTTVTATETDIVTVTTGYTGTETSTVTVTPTGTSTGTTTVVIQTPTTVTATETDIVTVTTGYTGTETSTVTVTPTGTS
TGTTTVVIQTPTTVTATETDIVTVTTGYTGTETSTVTVTPTGTSTGTTTVVIQTPTTVTATETDIVTVTTGYTGTETS
TVTVTPTGTSTGTTTVVIQTPTTVTATETDIVTVTTGYTGTETSTVTVTPTGTSTGTTTVVIQTPTTVTATETDIVTV
TTGYTGTETSTVTVTPTGTATGTTTVVINTPTTTGSEVLPTTGATGTAGTETQLTTATEVQPTTGATGTAGTETQV
TTGTETQATTATETQATTATEVQTTTGATGTAGTETQATTATEVQPTTGATGTAGTETQVTTATEVQPTTGATGT

	AGTETQVTTGTETQATTATETQATTATEVQTTTGATGTAGTETQATTATEVQPTTGATGTAGTETQVTTATEVQ<br>PTTGATGTAGTETQVTTGTETQATTATETQATTATEVQTTTGATGTAGTETQVTTATEVQPTTAVTETSSSGYYT<br>TIVSSTVVSTVVPGSTVYPVTHVTTTTGVSGESSAFTYTTSSTQYEPSTVVTTSYYTTSVYTSAPATETVSSTEAPE<br>SSTVTSNPIYQGSGTSTWSTVRQWNGSATYNYTYYTTGGFTGGNNTNVTGLYPSSAGANKPIAYLTFVSLFVYIV<br>TLI
JB760	MSVRRFLSTSARALLFTAALLPSLTSGLPSGNVRILQKGMEPEDYLSSASQNEVPHDISLPKTELADPNFLVDDMP<br>TLLGRDAAVDPSMFTSTFTVKNGNDANYITASPVSNDASMTAISTFTSGKEASYAIQASPSTFLPDSTTTSGSQVS<br>NAVEASSTFVADTTSTSCNPATVLIVTTSGSTSTSCPPPTTILIVTVPTTTTTTTVGYPGSVTTTLTGTPSNGTVIDTV<br>EVPTTTNYGYTTITTGYTGSTTLTTTVPHSGNETGPTTVYVETPYPTTVTTTTTVGYPGSVTTTLTGSPSNGTVIDT<br>VEIPTTTNYGYTTITTGYTGSTTLTTTVPHSGNETGPTTVYVETPYPTTVTTTTTVGYPGSVTTTLTGAPSNGTVID<br>TVEVPTTTNYGYTTVTTGYTGSTTLTTTVPHSGNETGPTTVYVETPYPTTVTTTTTVGYPGSVTTTLTGAPSNGTV<br>IDTVEVPTTTNYGYTTVTTGYTGSTTLTTTVPHSGNETGPTTVYVETPYPTTVTTTTTVGYPGSVTTTLTGAPSNG<br>TVIDTVEVPTTTNYGYTTVTTGYTGSTTLTTTVPHSGNETGPTTVYVETPYPTTVTTTTTVGYPGSVTTTLTGAPS<br>NGTVIDTVEVPTTTNYGYTTITTGYTGSTTLTTTVPHSGNETGPTTVYVETPYPTTVTTTTTVGYPGSVTTTLTGAP<br>SNGTVIDTVEVPTTTNYGYTTVTTGYTGSTTLTTTVPHSGNETGPTTVYVETPYPTTVTTTTTVGYPGSVTTTLTG<br>APSNGTVIDTVEVPTTTNYGYTTITTGYTGSTTLTTTVPHSGNETGPTTVYVETPYPTTVTTTTTVGYPGSVTTTLT<br>GAPSNGTVIDTVEVPTTTNYGYTTVTTGYTGSTTLTTTVPHSGNETGPTTVYVETPYPTTVTTTTTVGYPGSVTTT<br>LTGSGSNSIVTETVDVPTTTSVNYGYTTITTGWTGSTTLTSIVTHSGSETGPTTVYIETPSVSATTTTTTIGYSGSLTT<br>TLTGSSGPVVTNTVEIPYGNSSYIIPTTIVTGTVTTVTTGYTGTETSTVTVIPTGTTGTTTVVIQTPTTVTATETDIVT<br>VTTGYTGTETSTVTVTPTGTSTGTTTVVIQTPTTVTATETDIVTVTTGYTGTETSTVTVTPTGTSTGTTTVVIQTPTT<br>VTATETDIVTVTTGYTGTETSTVTVTPTGTSTGTTTVVIQTPTTVTATETDIVTVTTGYTGTETSTVTVTPTGTSTG<br>TTTVVIQTPTTVTATETDIVTVTTGYTGTETSTVTVTPTGTSTGTTTVVIQTPTTVTATETDIVTVTTGYTGTETSTV<br>TVTPTGTSTGTTTVVIQTPTTVTATETDIVTVTTGYTGTETSTVTVTPTGTATGTTTVVINTPTTTGSEVLPTTGAT<br>GTAGTETQLTTATEVQPTTGATGTAGTETQVTTGTETQATTATETQATTATEVQTTTGATGTAGTETQATTATE<br>VQPTTGATGTAGTETQVTTATEVQPTTGATGTAGTETQVTTGTETQATTATETQATTATEVQTTTGATGTAGTE<br>TQVTTATEVQPTTAVTETSSSGYYTTIVSSTVVSTVVPGSTVYPVTHVTTTTGVSGESSAFTYTTSSTQYEPSTVVT<br>TSYYTTSVYTSAPATETVSSTEAPESSTVTSNPIYQGSGTSTWSTVRQWNGSATYNYTYYTTGGFTGGNNTNVTG<br>LYPSSAGANKPIAYLTFVSLFVYIVTLI
JB4	MSVRRFLSTSARALLFTAALLPSLTSGLPSGNVRILQKGMEPEDYLSSASQNEVPHDISLPKTELADPNFLVDDMP<br>TLLGRDAAVDPSMFTSTFTVKNGNDANYITASPVSNDASMTAISTFTSGKEASYAIQASPSTFLPDSTTTSGSQVS<br>NAVEASSTFVADTTSTSCNPATVLIVTTSGSTSTSCPPPTTILIVTVPTTTTTTTVGYPGSVTTTLTGTPSNGTVIDTV<br>EVPTTTNYGYTTITTGYTGSTTLTTTVPHSGNETGPTTVYVETPYPTTVTTTTTVGYPGSVTTTLTGSPSNGTVIDT<br>VEIPTTTNYGYTTITTGYTGSTTLTTTVPHSGNETGPTTVYVETPYPTTVTTTTTVGYPGSVTTTLTGSPSNGTVIDT<br>VEIPTTTNYGYTTITTGYTGSTTLTTTVPHSGNETGPTTVYVETPYPTTVTTTTTVGHPGSVTTTLTGAPSNGTVID<br>TVEVPTTTNYGYTTVTTGYTGSTTLTTTVPHSGNETGPTTVYVETPYPTTVTTTTTVGYPGSVTTTLTGSPSNGTV<br>IDTVEIPTTTNYGYTTITTGYTGSTTLTTTVPHSGNETGPTTVYVETPYPTTVTTTTTVGYPGSVTTTLTGSPSNGTV

	IDTVEIPTTTNYGYTTITTGYTGSTTLTTTVPHSGNETGPTTVYVETPYPTTVTTTTTVGYPGSVTTTLTGAPSNGT<br>VIDTVEVPTTTNYGYTTVTTGYTGSTTLTTTVPHSGNETGPTTVYVETPYPTTVTTTTTVGYPGSVTTTLTGAPSN<br>GTVIDTVEIPTTTNYGYTTITTGYTGSTTLTTTVPHSGNETGPTTVYVETPYPTTVTTTTTVGYPGSVTTTLTGAPS<br>NGTVIDTVEVPTTTNYGYTTVTTGYTGSTTLTTTVPHSGNETGPTTVYVETPYPTTVTTTTTVGYSGSVTTTLTGS<br>GSNSIVTETVDVPTTTSVNYGYTTITTGWTGSTTLTSIVTHSGSETGPTTVYIETPSVSATTTTTTIGYSGSLTTTLTG<br>SSGPVVTNTVEIPYGNSSYIIPTTIVTGTVTTVTTGYTGTETSTVTVIPTGTTGTTTVVIQTPTTVTATETDIVTVTTG<br>YTGTETSTVTVTPTGTSTGTTTVVIQTPTTVTATETDIVTVTTGYTGTETSTVTVTPTGTSTGTTTVVIQTPTTVTAT<br>ETDIVTVTTGYTGTETSTVTVTPTGTATGTTTVVINTPTTTGSEVLPTTGATGTAGTETQLTTATEVQPTTGATGT<br>AGTETQVTTGTETQATTATETQATTATEVQTTTGATGTAGTETQATTATEVQPTTGATGTAGTETQVTTATEVQ<br>PTTGATGTAGTETQVTTGTETQATTATGTQATTATEVQTTTGATGTAGTETQVTTATEVQPTTAVTETSSSGYYT<br>TIVSSTVVSTVVPGSTVYPVTHVTTTTGVSGESSAFTYTTSSTQYEPSTVVTTSYYTTSVYTSAPATETVSSTEAPE<br>SSTVTSNPIYQGSGTSTWSTVRQWNGSATYNYTYYTTGGFTGGNNTNVTGLYPSSAGANKPIAYLTFVSLFVYIV<br>TLI
JB22	MSVRRFLSTSARALLFTAALLPSLTSGLPSGNVRILQKGMEPEDYLSSASQNEVPHDISLPKTELADPNFLVDDMP<br>TLLGRDAAVDPSMFTSTFTVKNGNDANYITASPVSNDASMTAISTFTSGKEASYAIQASPSTFLPDSTTTSGSQVS<br>NAVEASSTFVADTTSTSCNPATVLIVTTSGSTSTSCPPPTTILIVTVPTTTTTTTVGYPGSVTTTLTGTPSNGTVIDTV<br>EVPTTTNYGYTTITTGYTGSTTLTTTVPHSGNETGPTTVYVETPYPTTVTTTTTVGYPGSVTTTLTGAPSNGTVIDT<br>VEVPTTTNYGYTTVTTGYTGSTTLTTTVPHSGNETGPTTVYVETPYPTTVTTTTTVGYPGSVTTTLTGAPSNGTVI<br>DTVEVPTTTNYGYTTVTTGYTGSTTLTTTVPHSGNETGPTTVYVETPYPTTVTTTTTVGYPGSVTTTLTGAPSNGT<br>VIDTVEIPTTTNYGYTTITTGYTGSTTLTTTVPHSGNETGPTTVYVETPYPTTVTTTTTVGYPGSVTTTLTGAPSNG<br>TVIDTVEVPTTTNYGYTTITTGYTGSTTLTTTVPHSGNETGPTTVYVETPYPTTVTTTTTVGYPGSVTTTLTGAPSN<br>GTVIDTVEVPTTTNYGYTTVTTGYTGSTTLTTTVPHSGNETGPTTVYVETPYPTTVTTTTTVGYPGSVTTTLTGAP<br>SNGTVIDTVEVPTTTNYGYTTVTTGYTGSTTLTTTVPHSGNETGPTTVYVETPYPTTVTTTTTVGYSGSVTTTLTG<br>SGSNSIVTETVDVPTTTSVNYGYTTITTGWTGSTTLTSIVTHSGSETGPTTVYIETPSVSATTTTTTIGYSGSLTTTLT<br>GSSGPVVTNTVEIPYGNSSYIIPTTIVTGTVTTVTTGYTGTETSTVTVIPTGTTGTTTVVIQTPTTVTATETDIVTVTT<br>GYTGTETSTVTVTPTGTSTGTTTVVIQTPTTVTATETDIVTVTTGYTGTETSTVTVTPTGTSTGTTTVVIQTPTTVT<br>ATETDIVTVTTGYTGTETSTVTVTPTGTSTGTTTVVIQTPTTVTATETDIVTVTTGYTGTETSTVTVTPTGTSTGTT<br>TVVIQTPTTVTATETDIVTVTTGYTGTETSTVTVTPTGTSTGTTTVVIQTPTTVTATETDIVTVTTGYTGTETSTVT<br>VTPTGTSTGTTTVVIQTPTTVTATETDIVTVTTGYTGTETSTVTVTPTGTATGTTTVVINTPTTTGSEVLPTTGATG<br>TAGTETQLTTATEVQPTTGATGTAGTETQVTTGTETQATTATETQATTATEVQTTTGATGTAGTETQATTATEV<br>QPTTGATGTAGTETQVTTATEVQPTTGATGTAGTETQVTTGTETQATTATETQATTATEVQTTTGATGTAGTET<br>QVTTATEVQPTTAVTETSSSGYYTTIVSSTVVSTVVPGSTVYPVTHVTTTTGVSGESSAFTYTTSSTQYEPSTVVTT<br>SYYTTSVYTSAPATETVSSTEAPESSTVTSNPIYQGSGTSTWSTVRQWNGSATYNYTYYTTGGFTGGNNTNVTGL<br>YPSSAGANKPIAYLTFVSLFVYIVTLI

JB758	MSVRRFLSTSARALLFTAALLPSLTSGLPSGNVRILQKGMEPEDYLSSVASQNEVPHDISLPKTELADPNFLVDDM<br>PTLLGRDAAVDPSMFTSTFTVKNGNDANYITASPVSNDASMTAISTFTSGKEASYAIQASPSTFLPDSTTTSGSQVS<br>NAVEASSTFVADTTSTSCNPATVLIVTTSGSTSTSCPPPTTILIVTVPTTTTTTTVGYPGSVTTTLTGTPSNGTVIDTV<br>EVPTTTNYGYTTITTGYTGSTTLTTTVPHSGNETGPTTVYVETPYPTTVTTTTTVGYPGSVTTTLTGSPSNGTVIDT<br>VEVPTTTNYGYTTITTGYTGSTTLTTTVPHSGNETGPTTVYVETPYPTTVTTTTTVGYPGSVTTTLTGSPSNGTVID<br>TVEVPTTTNYGYTTVTTGYTGSTTLTTTVPHSGNETGPTTVYVETPYPTTVTTTTTVGYPGSVTTTLTGSPSNGTV<br>IDTVEVPTTTNYGYTTITTGYTGSTTLTTTVPHSGNETGPTTVYVETPYPTTVTTTTTVGYPGSVTTTLTGSPSNGT<br>VIDTVEVPTTTNYGYTTVTTGYTGSTTLTTTVPHSGNETGPTTVYVETPYPTTVTTTTTVGYPGSVTTTLTGSPSN<br>GTVIDTVEVPTTTNYGYTTITTGYTGSTTLTTTVPHSGNETGPTTVYVETPYPTTVTTTTTVGYPGSVTTTLTGSPS<br>NGTVIDTVEVPTTTNYGYTTITTGYTGSTTLTTTVPHSGNETGPTTVYVETPYPTTVTTTTTVGYPGSVTTTLTGSP<br>SNGTVIDTVEVPTTTNYGYTTITTGYTGSTTLTTTVPHSGNETGPTTVYVETPYPTTVTTTTTVGYPGSVTTTLTGS<br>PSNGTVIDTVEVPTTTNYGYTTVTTGYTGSTTLTTTVPHSGNETGPTTVYVETPTSSATTPSTVTITTTVGYPGSVT<br>TTLTGSPSNGTVIDTVEVPTTTNYGYTTITTGYTGSTTLTTTVPHSGNETGPTTVYVETPYPTTVTTTTTVGYPGSV<br>TTTLTGSPSNGTVIDTVEVPTTTNYGYTTVTTGYTGSTTLTTTVPHSGNETGPTTVYVETPYPTTVTTTTTVGYPG<br>SVTTTLTGSPSNGTVIDTVEVPTTTNYGYTTITTGYTGSTTLTTTVPHSGNETGPTTVYVETPYPTTVTTTTTVGYP<br>GSVTTTLTGSPSNGTVIDTVEVPTTTNYGYTTITTGYTGSTTLTTTVPHSGNETGPTTVYVETPTSSATTPSTVTITT<br>TVGYPGSVTTTLTGSPSNGTVIDTVEVPTTTNYGYTTVTTGYTGTTTLTTTVPHSGNETGPTTVYVETPTISTVSSV<br>STVTTTTTVGYSGSVTTTLTGSGSNSIVTETVEVPTTTSVSYGYTTITTGWTGSTTLTSIVTHSGSETGPTTVYIETPS<br>VSATTTTTTIGYSGSLTTTLTGSSGPVVTNTVEIPYGNSSYIIPTTIVTGTVTTVTTGYTGTETSTVTVTPTGSTGTTT<br>VVIQTPTTVTATETDIVTVTTGYTGTETSTVTVTPTGTSTGTTTVVIQTPTTVTATETDIVTVTTGYTGTETSTVTV<br>TPTGTSTGTTTVVIQTPTTVTATETDIVTVTTGYTGTETSTVTVTPTGTATGTTTVVINTPTTTGSEVVPTTGATGT<br>AGTETQVTTATEVQPTTGATGTAGTETQVTTGTETQATTATETQATTATETQATTATEVQPTTGATGTAGTETQ<br>VTTGTETQATTATETQATTATEVQPTTGATGTAGTETQVTTATEVQPTTAVTETSSSGYYTTIVSSTVVSTVVPGS<br>TVYPVTHVTTTTGASGESSAFTYTTSSTQYEPSTVVTTSYYTTSVYTSAPATETVSSTEAPESSTVTSNPIYQGSGT<br>STWSTVRQWNGSATYNYTYYTTGGFTGGNNTNVTGLYPSSAGANKPIAYLTFVSLFVYIVTLI
JB837	MSVRRFLSTSARALLFTAALLPSLTSGLPSGNVRILQKGMEPEDYLSSVASQNEVPHDISLPKTELADPNFLVDDM<br>PTLLGRDAAVDPSMFTSTFTVKNGNDANYITASPVSNDASMTAISTFTSGKEASYAIQASPSTFLPDSTTTSGSQVS<br>NAVEASSTFVADTTSTSCNPATVLIVTTSGSTSTSCPPPTTILIVTVPTTTTTTTVGYPGSVTTTLTGSPSNGTVIDTV<br>EVPTTTNYGYTTITTGYTGTTTLTTTVPHSGNETGPTTVYVETPYPTTVTTTTTVGYPGSVTTTLTGSPSNGTVIDT<br>VEVPTTTNYGYTTITTGYTGTTTLTTTVPHSGNETGPTTVYVETPYPTTVTTTTTVGYPGSVTTTLTGSPSNGTVID<br>TVEVPTTTNYGYTTITTGYSGTTTLTTTVPHSGNETGPTTVYVETPTSSATTPSTVTITTTVGYPGSVTTTLTGSPSN<br>GTVIDTVEVPTTTNYGYTTITTGYTGSTTLTTTVPHSGNETGPTTVYVETPYPTTVTTTTTVGYPGSVTTTLTGSPS<br>NGTVIDTVEVPTTTNYGYTTITTGYTGTTTLTTTVPHSGNETGPTTVYVETPYPTTVTTTTTVGYPGSVTTTLTGSP<br>SNGTVIDTVEVPTTTNYGYTTITTGYTGTTTLTTTVPHSGNETGPTTVYVETPYPTTVTTTTTVGYPGSVTTTLTGS<br>PSNGTVIDTVEVPTTTNYGYTTITTGYTGSTTLITTVPHSGNETGPTTVYVETPYPTTVTTTTTVGYPGSVTTTLTG<br>SGSNSIVTETVEVPTTTSVNYGYTTITTGWTGSTTLTSIVTHSGSETGPTTVYIETPSVSATTTTTTIGYSGSLTTTLT

	GSSGPVVTNTVEIPYGNSSYIIPTTIVTGTVTTVTTGYTGTETSTVTVTPTGSTGTTTVVIQTPTTVTATETDIVTVTT<br>GYTGTETSTVTVTPTGTSTGTTTVVIQTPTTVTATETDIVTVTTGYTGTETSTVTVTPTGTSTGTTTVVIQTPTTVT<br>ATETDIVTVTTGYTGTETSTVTVTPTGTSTGTTTVVIQTPTTVTATETDIVTVTTGYTGTETSTVTVTPTGTSTGTT<br>TVVIQTPTTVTATETDIVTVTTGYTGTETSTVTVTPTGTSTGTTTVVINTPTTTGSEVLPTTGATGTAGTETQVTTG<br>TETQATTATETQATTATEVLPTTGATGTAGTETQVTTGTETQATTATETQATTATEVQPTTGATGTAGTETQVTT<br>ATEVQPTTGATGTAGTETQVTTGTETQATTATETQATTATEVQPTTGATGTAGTETQVTTATEVQPTTGATGTA<br>GTETQVTTGTETQATTATETQATTATETQATTATEVQPTTGATGTAGTETQVTTGTETQATTATETQATTATEVQ<br>PTTGATGTAGTETQVTTATEVQPTTAVTETSSSGYYTTIVSSTVLSTVVPGSTVYPVTHVTTTTGASGESSAFTYTT<br>SSTQYEPSTVVTTSYYTTSVYTSAPATETVSSTEAPESSTVTSNPIYQGSGTSTWSTVRQWNGSATYNYTYYTTGG<br>FTGGNNTNVTGLYPSSAGANKPIAYLTFVSLFVYIVTLI
JB840	MSVRRFLSTSARALLFTAALLPSLTSGLPSGNVRILQKGMEPEDYLSSVASQNEVPHDISLPKTELADPNFLVDDM<br>PTLLGRDAAVDPSMFTSTFTVKNGNDANYITASPVSNDASMTAISTFTSGKEASYAIQASPSTFLPDSTTTSGSQVS<br>NAVEASSTFVADTTSTSCNPATVLIVTTSGSTSTSCPPPTTILIVTVPTTTTTTTVGYPGSVTTTLTGSPSNGTVIDTV<br>EVPTTTNYGYTTVTTGYTGSTTLTTTVPHSGNETGPTTVYVETPYPTTVTTTTTVGYPGSVTTTLTGSPSNGTVID<br>TVEVPTTTNYGYTTITTGYTGSTTLTTTVPHSGNETGPTTVYVETPYPTTVTTTTTVGYPGSVTTTLTGSPSNGTVI<br>DTVEVPTTTNYGYTTITTGYTGSTTLTTTVPHSGNETGPTTVYVETPYPTTVTTTTTVGYPGSVTTTLTGSPSNGT<br>VIDTVEVPTTTNYGYTTITTGYTGSTTLTTTVPHSGNETGPTTVYVETPYPTTVTTSTTVGYPGSVTTTLTGSPSNG<br>TVIDTVEVPTTTNYGYTTVTTGYTGTTTLTTTVPHSGNETGPTTVYVETPTSSATTPSTVTITTTVGYPGSVTTTLT<br>GSPSNGTVIDTVEVPTTTNYGYTTVTTGYTGTTTLTTTVPHSGNETGPTTVYVETPPSSATTPSTVTITTTVGYPGS<br>VTTTLTGTPSNGTVIDTVEVPTTTNYGYTTVTTGYTGTTTLTTTVPHSGNETGPTTVYVETPTSSATTPSTVTITTT<br>VGYPGSVTTTLTGCPSNGTVIDTVEVPTTTSYGYTTVTTGYTGTTTLTTTVPHSGNETGPTTVYVETPTISTVSSVS<br>TVTTTTTVGYSGSVTTTLTGSGSNSIVTETVEVPTTTTDYGYTTVTTGYTGSTTLTTTVPHSGNETGPTTVYVETPT<br>ISTVSSVSTVTTTTTVGYSGSVTTTLTGSGSNSIVTETVEVPTTTSVSYGYTTITTGWTGSTTLTSIVTHSGSETGPTT<br>VYIETPSVSATTTTTTIGYSGSLTTTLTGSSGPVVTNTVEIPYGNSSYIIPTTIVTGTVTTVTTGYTGTETSTVTVTPT<br>GSTGTTTVVIQTPTTVTATETDIVTVTTGYTGTETSTVTVTPTGTSTGTTTVVIQTPSTVTATETDIVTVTTGYTGTE<br>TSTVTVTPTGTSTGTTTVVIQTPSTVTATETDIVTVTTGYTGTETSTVTVTPTGTSTGTTTVVIQTPSTVTATETDIV<br>TVTTGYTGTETSTITVTPTGAATGTTTVVINTPTTTGSEVVPTTGATGTAGTETQATIATEVQTTTGATGTAGTET<br>QVTTGTETQATTATETQATTATETQATTATEVQPTTGATGTAGTETQVTTGTETQATTATEVQPTTGATGTAGTE<br>TQVTTATEVQPTTAVTETSSSGYYTTIVSSTVVSTVVPGSTVYPVTHVTTTTGASGESSAFTYTTSSTQYEPSTVVT<br>TSYYTTSVYTSAPASETVSSTEAPESSTVTSNPIYQGSGTSTWSTVRQWNGSATYNYTYYTTGGFTGGNNTNVTG<br>LYPSSAGANKPIAYLTFVSLFVYIVTLI
JB842	MSVRRFLSTSARALLFTAALLPSLTSGLPSGNVRILQKGMEPEDYLSSVASQNEVPHDISLPKTELADPNFLVDDM<br>PTLLGRDAAVDPSMFTSTFTVKNGNDANYITASPVSNDASMTAISTFTSGKEASYAIQASPSTFLPDSTTTSGSQVS<br>NAVEASSTFVADTTSTSCNPATVLIVTTSGSTSTSCPPPTTILIVTVPTTTTTTTVGYPGSVTTTLTGSPSNGTVIDTV<br>EVPTTTNYGYTTITTGYTGSTTLTTTVPHSGNETGPTTVYVETPYPTTVTTTTTVGYPGSVTTTLTGSPSNGTVIDT

	VEVPTTTNYGYTTITTGYTGSTTLTTTVPHSGNETGPTTVYVETPYPTTVTTTTTVGYPGSVTTTLTGSPSNGTVID<br>TVEVPTTTNYGYTTITTGYTGSTTLTTTVPHSGNETGPTTVYVETPYPTTVTTTTTVGYPGSVTTTLTGSPSNGTVI<br>DTVEVPTTTNYGYTTITTGYTGSTTLTTTVPHSGNETGPTTVYVETPTISTVSSVSTVTTTTTVGYSGSVTTTLTGS<br>GSNSIVTETVEVPTTTSVSYGYTTITTGWTGSTTLTSIVTHSGSETGPTTVYIETPSVSATTTTTTIGYSGSLTTTLTG<br>SSGPVVTNTVEIPYGNSSYIIPTAIVTGTVTTVTTGYTGTETSTVTVTPTGSTGTTTVVIQTPTTVTATETDIVTVTT<br>GYTGTETSTVTVTPTGTSTGTTTVVIQTPTTVTATETDIVTVTTGYTGTETSTVTVTPTGTSTGTTTVVIQTPTTVT<br>ATETDIVTVTTGYTGTETSTITVTPTGAATGTTTVVINTPTTTGSEVVPTTGATGTAGTETQVTTGTETQATTATEV<br>QPTTGATGTAGTETQVTTGTETQATTATETQATTATETQATIATEVQTTTGATGTAGTETQVTTGTETQATTATE<br>TQATTATETQATTATEVQPTTGATGTAGTETQVTTGTETQATTATETQATTATETQATIATEVQTTTGATGTAGT<br>ETQVTTGTETQATTATETQATTATETQATTATEVQPTTGATGTAGTETQVTTGTETQATTATETQATTATETQAT<br>IATEVQTTTGATGTAGTETQVTTGTETQATTATETQATTATETQATTATEVQPTTGATGTAGTETQVTTGTETQA<br>TTATETQATTATETQATIATEVQTTTGATGTAGTETQVTTGTETQATTATETQATTATETQATTATEVQPTTGAT<br>GTAGTETQVTTGTETQATTATETQATTATETQATIATEVQTTTGATGTAGTETQVTTGTETQATTATETQATTAT<br>ETQATTATEVQPTTGATGTAGTETQVTTGTETQATTATETQATTATETQATIATEVQTTTGATGTAGTETQVTTG<br>TETQATTATETQATTATETQATTATEVQPTTGATGTAGTETQVTTGTETQATTATETQATTATETQATIATEVQT<br>TTGATGTAGTETQVTTGTETQATTATETQATTATETQATTATEVQPTTGATGTAGTETQVTTGTETQATTATETQ<br>ATTATETQATIATEVQTTTGATGTAGTETQVTTGTETQATTATETQATTATETQATTATEVQPTTGATGTAGTET<br>QVTTGTETQATTATETQATTATETQATIATEVQTTTGATGTAGTETQVTTGTETQATTATETQATTATETQATTA<br>TEVQPTTGATGTAGTETQVTTGTETQATTATETQATTATETQATIATEVQTTTGATGTAGTETQVTTGTETQATT<br>ATETQATTATETQATTATEVQPTTGATGTAGTETQVTTGTETQATTATETQATTATETQATIATEVQTTTGATGT<br>AGTETQVTTGTETQATTATETQATTATETQATTATEVQPTTGATGTAGTETQVTTGTETQATTATETQATTATET<br>QATIATEVQTTTGATGTAGTETQVTTGTETQATTATETQATTATETQATTATEVQPTTGATGTAGTETQVTTGTE<br>TQATTATETQATTATETQATIATEVQTTTGATGTAGTETQVTTGTETQATTATETQATTATETQATTATEVQPTT<br>GATGTAGTETQVTTGTETQATTATETQATTATETQATIATEVQTTTGATGTAGTETQVTTGTETQATTATETQAT<br>TATETQATTATEVQPTTGATGTAGTETQVTTGTETQATTATETQATTATETQATIATEVQTTTGATGTAGTETQV<br>TTGTETQATTATETQATTATETQATTATEVQPTTGATGTAGTETQVTTGTETQATTATETQATTATETQATIATE<br>VQTTTGATGTAGTETQVTTGTETQATTATETQATTATETQATTATEVQPTTGATGTAGTETQVTTGTETQATTA<br>TETQATTATETQATIATEVQTTTGATGTAGTETQVTTGTETQATTATETQATTATETQATTATEVQPTTGATGTA<br>GTETQVTTGTETQATTATETQATTATETQATIATEVQTTTGATGTAGTETQVTTGTETQATTATETQATTATETQ<br>ATTATETQATTATEVQPTTAVTETSSSGYYTTIVSSTVVSTVVPGSTVYPVTHVTTTTGASGESSAFTYTTSSTQYE<br>PSTVVTTSYYTTSVYTSAPASETVSSTEAPESSTVTSNPIYQGSGTSTWSTVRQWNGSATYNYTYYTTGGFTGGN<br>NTNVTGLYPSSAGANKPIAYLTFVSLFVYIVTLI
JB853	MSVRRFLSTSARALLFTAALLPSLTSGLPSGNVRILQKGMEPEDYLSSVASQNEVPHDISLPKTELADPNFLVDDM<br>PTLLGRDAAVDPSMFTSTFTVKNGNDANYITASPVSNDASMTAISTFTSGKEASYAIQASPSTFLPDSTTTSGSQVS<br>NAVEASSTFVADTTSTSCNPATVLIVTTSGSTSTSCPPPTTILIVTVPTTTTTTTVGYPGSVTTTLTGSPSNGTVIDTV<br>EVPTTTNYGYTTITTGYTGSTTLTTTVPHSGNETGPTTVYVETPYPTTVTTTTTVGYPGSVTTTLTGSPSNGTVIDT

	VEVPTTTNYGYTTITTGYTGSTTLTTTVPHSGNETGPTTVYVEIPYPTTVTTTTTVGYPGSVTTTLTGSPSNGTVID<br>TVEVPTTTNYGYTTITTGYTGSTTLTTTVPHSGNETGPTTVYVETPYPTTVTTTTTVGYPGSVSTTLTGSPSNGTVI<br>DTVEVPTTTNYGYTTITTGYTGSTTLTTTVPHSGNETGPTTVYVEIPYPTTVTTTTTVGYPGSVTTTLTGSPSNGTV<br>IDTVEVPTTTNYGYTTITTGYTGSTTLTTTVPHSGNETGPTTVYVETPYPTTVTTTTTVGYPGSVTTTLTGSPSNGT<br>VIDTVEVPTTTNYGYTTITTGYTGSTTLTTTVPHSGNETGPTTVYVETPYPTTVTTTTTVGYPGSVTTTLTGSPSNG<br>TVIDTVEVPTTTNYGYTTITTGYTGSTTLTTTVPHSGNETGPTTVYVETPTSSATTPSMVTITTTVGYPGSVTTTLT<br>GSPSNGTVIDTVEVPTTTNYGYTTVTTGYTGTTTLTTTVPHSGNETGPTTVYVETPTISTVSSVSTVTTTTTVGYSG<br>SVTTTLTGSGSNSIVTETVEVPTTTSVSYGYTTITTGWTGSTTLTSIVTHSGSETGPTTVYIETPSVSATTTTTTIGYS<br>GSLTTTLTGSSGPVVTNTVEIPYGNSSYIIPTAIVTGTVTTVTTGYTGTETSTVTVTPTGSTGTTTVVIQTPTTVTAT<br>ETDIVTVTTGYTGTETSTVTVTPTGTSTGTTTVVIQTPTTVTATETDIVTVTTGYTGTETSTVTVTPTGTSTGTTTV<br>VIQTPTTVTATETDIVTVTTGYTGTETSTITVTPTGAATGTTTVVINTPTTTGSEVVPTTGATGTAGTETQVTTGTE<br>TQATTATEVQPTTGATGTAGTETQVTTGTETQATTATETQATTATETQATIATEVQTTTGATGTAGTETQVTTGT<br>ETQATTATETQATTATETQATTATEVQPTTGATGTAGTETQVTTGTETQATTATETQATTATETQATIATEVQTT<br>TGATGTAGTETQVTTGTETQATTATETQATTATETQATTATEVQPTTGATGTAGTETQVTTATEVQPTTAVTETS<br>SSGYYTTIVSSTVVSTVVPGSTVYPVTHVTTTGASGESSAFTYTTSSTQYEPSTVVTTSYYTTSVYTSAPASETVS<br>STEAPESSTVTSNPIYQGSGTSTWSTVRQWNGSATYNYTYYTTGGFTGGNNTNVTGLYPSSAGANKPIAYLTFVS<br>LFVYIVTLI
JB858	MSVRRFLSTSARALLFTAALLPSLTSGLPSGNVRILQKGMEPEDYLSSVASQNEVPHDISLPKTELADPNFLVDDM<br>PTLLGRDAAVDPSMFTSTFTVKNGNDANYITASPVSNDASMTAISTFTSGKEASYAIQASPSTFLPDSTTTSGSQVS<br>NAVEASSTFVADTTSTSCNPATVLIVTTSGSTSTSCPPPTTILIVTVPTTTTTTTVGYPGSVTTTLTGSPSNGTVIDTV<br>EVPTTTNYGYTTITTGYTGSTTLTTTVPHSGNETGPTTVYVETPYPTTVTTTTTVGYPGSVTTTLTGSPSNGTVIDT<br>VEVPTTTNYGYTTITTGYTGSTTLTTTVPHSGNETGPTTVYVETPYPTTVTTTTTVGYPGSVTTTLTGSPSNGTVID<br>TVEVPTTTNYGYTTITTGYTGSTTLTTTVPHSGNETGPTTVYVETPYPTTVTTTTTVGYPGSVTTTLTGSPSNGTVI<br>DTVEVPTTTNYGYTTITTGYTGSTTLTTTVPHSGNETGPTTVYVETPTISTVSSVSTVTTTTTVGYSGSVTTTLTGS<br>GSNSIVTETVEVPTTTSVSYGYTTITTGWTGSTTLTSIVTHSGSETGPTTVYIETPSVSATTTTTTIGYSGSLTTTLTG<br>SSGPVVTNTVEIPYGNSSYIIPTAIVTGTVTTVTTGYTGTETSTVTVTPTGSTGTTTVVIQTPTTVTATETDIVTVTT<br>GYTGTETSTVTVTPTGTSTGTTTVVIQTPTTVTATETDIVTVTTGYTGTETSTVTVTPTGTSTGTTTVVIQTPTTVT<br>ATETDIVTVTTGYTGTETSTITVTPTGAATGTTTVVINTPTTTGSEVVPTTGATGTAGTETQVTTGTETQATTATEV<br>QPTTGATGTAGTETQVTTGTETQATTATETQATTATETQATIATEVQTTTGATGTAGTETQVTTGTETQATTATE<br>TQATTATETQATTATEVQPTTGATGTAGTETQVTTGTETQATTATETQATTATETQATIATEVQTTTGATGTAGT<br>ETQVTTGTETQATTATETQATTATETQATTATEVQPTTGATGTAGTETQVTTGTETQATTATETQATTATETQAT<br>IATEVQTTTGATGTAGTETQVTTGTETQATTATETQATTATETQATTATEVQPTTGATGTAGTETQVTTGTETQA<br>TTATETQATTATETQATIATEVQTTTGATGTAGTETQVTTGTETQATTATETQATTATETQATTATEVQPTTGAT<br>GTAGTETQVTTGTETQATTATETQATTATETQATIATEVQTTTGATGTAGTETQVTTGTETQATTATETQATTAT<br>ETQATTATEVQPTTGATGTAGTETQVTTGTETQATTATETQATTATETQATIATEVQTTTGATGTAGTETQVTTG<br>TETQATTATETQATTATETQATTATEVQPTTGATGTAGTETQVTTGTETQATTATETQATTATETQATIATEVQT

	TTGATGTAGTETQVTTGTETQATTATETQATTATETQATIATEVQTTTGATGTAGTETQVTTGTETQATTATETQ<br>ATTATETQATTATEVQPTTGATGTAGTETQVTTGTETQATTATETQATTATETQATIATEVQTTTGATGTAGTET<br>QVTTGTETQATTATETQATTATETQATTATEVQPTTGATGTAGTETQVTTGTETQATTATETQATTATETQATIA<br>TEVQTTTGATGTAGTETQVTTGTETQATTATETQATTATETQATTATEVQPTTGATGTAGTETQVTTGTETQATT<br>ATETQATTATETQATIATEVQTTTGATGTAGTETQVTTGTETQATTATETQATTATETQATTATEVQPTTGATGT<br>AGTETQVTTGTETQATTATETQATTATETQATIATEVQTTTGATGTAGTETQVTTGTETQATTATETQATTATET<br>QATTATEVQPTTGATGTAGTETQVTTGTETQATTATETQATTATETQATIATEVQTTTGATGTAGTETQVTTGTE<br>TQATTATETQATTATETQATTATEVQPTTGATGTAGTETQVTTGTETQATTATETQATTATETQATIATEVQTTT<br>GATGTAGTETQVTTGTETQATTATETQATTATETQATTATEVQPTTGATGTAGTETQVTTGTETQATTATETQA<br>TTATETQATIATEVQTTTGATGTAGTETQVTTGTETQATTATETQATTATETQATTATEVQPTTAVTETSSSGYYT<br>TIVSSTVVSTVVPGSTVYPVTHVTTTTGASGESSAFTYTTSSTQYEPSTVVTTSYYTTSVYTSAPASETVSSTEAPES<br>STVTSNPIYQGSGTSTWSTVRQWNGSATYNYTYYTTGGFTGGNNTNVTGLYPSSAGANKPIAYLTFVSLFVYIVT<br>LI
JB869	MSVRRFLSTSARALLFTAALLPSLTSGLPSGNVRILQKGMEPEDYLSSASQNEVPHDISLPKTELADPNFLVDDMP<br>TLLGRDAAVDPSMFTSTFTVKNGNDANYITASPVSNDASMTAISTFTSGKEASYAIQASPSTFLPDSTTTSGSQVS<br>NAVEASSTFVADTTSTSCNPATVLIVTTSGSTSTSCPPPTTILIVTVPTTTTTTTVGYPGSVTTTLTGTPSNGTVIDTV<br>EVPTTTNYGYTTITTGYTGSTTLTTTVPHSGNETGPTTVYVETPYPTTVTTTTTVGYPGSVTTTLTGAPSNGTVIDT<br>VEVPTTTNYGYTTITTGYTGSTTLTTTVPHSGNETGPTTVYVETPYPTTVTTTTTVGYPGSVTTTLTGAPSNGTVID<br>TVEVPTTTNYGYTTITTGYTGSTTLTTTVPHSGNETGPTTVYVETPYPTTVTTTTTVGYPGSVTTTLTGAPSNGTVI<br>DTVEVPTTTNYGYTTITTGYTGSTTLTTTVPHSGNETGPTTVYVETPYPTTVTTTTTVGYPGSVTTTLTGAPSNGT<br>VIDTVEVPTTTNYGYTTVTTGYTGSTTLTTTVPHSGNETGPTTVYVETPYPTTVTTTTTVGYPGSVTTTLTGAPSN<br>GTVIDTVEVPTTTNYGYTTITTGYTGSTTLTTTVPHSGNETGPTTVYVETPYPTTVTTTTTVGYPGSVTTTLTGAPS<br>NGTVIDTVEVPTTTNYGYTTVTTGYTGSTTLTTTVPHSGNETGPTTVYVETPYPTTVTTTTTVGYPGSVTTTLTGA<br>PSNGTVIDTVEVPTTTNYGYTTITTGYTGSTTLTTTVPHSGNETGPTTVYVETPYPTTVTTTTTVGYPGSVTTTLTG<br>APSNGTVIDTVEVPTTTNYGYTTITTGYTGSTTLTTTVPHSGNETGPTTVYVETPYPTTVTTTTTVGYPGSVTTTLT<br>GAPSNGTVIDTVEVPTTTNYGYTTITTGYTGSTTLTTTVPHSGNETGPTTVYVETPYPTTVTTTTTVGYPGSVTTTL<br>TGAPSNGTVIDTVEVPTTTNYGYTTITTGYTGSTTLTTTVPHSGNETGPTTVYVETPYPTTVTTTTTVGYPGSVTTT<br>LTGAPSNGTVIDTVEVPTTTNYGYTTITTGYTGSTTLTTTVPHSGNETGPTTVYVETPYPTTVTTTTTVGYPGSVTT<br>TLTGAPSNGTVIDTVEVPTTTNYGYTTITTGYTGSTTLTTTVPHSGNETGPTTVYVETPYPTTVTTTTTVGYPGSVT<br>TTLTGAPSNGTVIDTVEVPTTTNYGYTTITTGYTGSTTLTTTVPHSGNETGPTTVYVETPYPTTVTTTTTVGYPGSV<br>TTTLTGAPSNGTVIDTVEVPTTTNYGYTTITTGYTGSTTLTTTVPHSGNETGPTTVYVETPYPTTVTTTTTVGYPGS<br>VTTTLTGAPSNGTVIDTVEVPTTTNYGYTTITTGYTGSTTLTTTVPHSGNETGPTTVYVETPYPTTVTTTTTVGYPG<br>SVTTTLTGAPSNGTVIDTVEVPTTTNYGYTTITTGYTGSTTLTTTVPHSGNETGPTTVYVETPYPTTVTTTTTVGYP<br>GSVTTTLTGAPSNGTVIDTVEVPTTTNYGYTTITTGYTGSTTLTTTVPHSGNETGPTTVYVETPYPTTVTTTTTVGY<br>PGSVTTTLTGAPSNGTVIDTVEVPTTTNYGYTTVTTGYTGSTTLTTTVPHSGNETGPTTVYVETPYPTTVTTTTTV<br>GYPGSVTTTLTGAPSNGTVIDTVEVPTTTNYGYTTITTGYTGSTTLTTTVPHSGNETGPTTVYVETPYPTTVTTTTT

	VGYPGSVTTTLTGAPSNGTVIDTVEVPTTTNYGYTTITTGYTGSTTLTTTVPHSGNETGPTTVYVETPYPTTVTTTT<br>TVGYPGSVTTTLTGAPSNGTVIDTVEVPTTTNYGYTTVTTGYTGSTTLTTTVPHSGNETGPTTVYVETPYPTTVTT<br>TTTVGYPGSVTTTLTGAPSNGTVIDTVEVPTTTNYGYTTVTTGYTGSTTLTTTVPHSGNETGPTTVYVETPYPTTV<br>TTTTTVGYSGSVTTTLTGSGSNSIVTETVDVPTTTSVNYGYTTITTGWTGSTTLTSIVTHSGSETGPTTVYIETPSVS<br>ATTTTTTIGYSGSLTTTLTGSSGPVVTNTVEIPYGNSSYIIPTTIVTGTVTTVTTGYTGTETSTVTVIPTGTTGTTTVV<br>IQTPTTVTATETDIVTVTTGYTGTETSTVTVTPTGTSTGTTTVVIQTPTTVTATETDIVTVTTGYTGTETSTVTVTPT<br>GTSTGTTTVVIQTPTTVTATETDIVTVTTGYTGTETSTVTVTPTGTSTGTTTVVIQTPTTVTATETDIVTVTTGYTG<br>TETSTVTVTPTGTSTGTTTVVIQTPTTVTATETDIVTVTTGYTGTETSTVTVTPTGTSTGTTTVVIQTPTTVTATETD<br>IVTVTTGYTGTETSTVTVTPTGTSTGTTTVVIQTPTTVTATETDIVTVTTGYTGTETSTVTVTPTGTSTGTTTVVIQT<br>PTTVTATETDIVTVTTGYTGTETSTVTVTPTGTATGTTTVVINTPTTTGSEVLPTTGATGTAGTETQLTTATEVQPT<br>TGATGTAGTETQVTTGTETQATTATETQATTATEVQTTTGATGTAGTETQATTATEVQPTTGATGTAGTETQVT<br>TATEVQPTTGATGTAGTETQVTTGTETQATTATETQATTATEVQTTTGATGTAGTETQVTTATEVQPTTAVTETS<br>SSGYYTTIVSSTVVSTVVPGSTVYPVTHVTTTTGVSGESSAFTYTTSSTQYEPSTVVTTSYYTTSVYTSAPATETVS<br>STEAPESSTVTSNPIYQGSGTSTWSTVRQWNGSATYNYTYYTTGGFTGGNNTNVTGLYPSSAGANKPIAYLTFVS<br>LFVYIVTLI
JB872	MSVRRFLSTSARALLFTAALLPSLTSGLPSGNVRILQKGMEPEDYLSSVASQNEVPHDISLPKTELADPNFLVDDM<br>PTLLGRDAAVDPSMFTSTFTVKNGNDANYITASPVSNDASMTAISTFTSGKEASYAIQASPSTFLPDSTTTSGSQVS<br>NAVEASSTFVADTTSTSCNPATVLIVTTSGSTSTSCPPPTTILIVTVPTTTTTTTVGYPGSVTTTLTGSPSNGTVIDTV<br>EVPTTTNYGYTTITTGYTGSTTLTTTVPHSGNETGPTTVYVETPYPTTVTTTTTVGYPGSVTTTLTGSPSNGTVIDT<br>VEVPTTTNYGYTTITTGYTGSTTLTTTVPHSGNETGPTTVYVEIPYPTTVTTTTTVGYPGSVTTTLTGSPSNGTVID<br>TVEVPTTTNYGYTTITTGYTGSTTLTTTVPHSGNETGPTTVYVETPYPTTVTTTTTVGYPGSVTTTLTGSPSNGTVI<br>DTVEVPTTTNYGYTTITTGYTGSTTLTTTVPHSGNETGPTTVYVEIPYPTTVTTTTTVGYPGSVTTTLTGSPSNGTV<br>IDTVEVPTTTNYGYTTITTGYTGSTTLTTTVPHSGNETGPTTVYVETPYPTTVTTTTTVGYPGSVTTTLTGSPSNGT<br>VIDTVEVPTTTNYGYTTITTGYTGSTTLTTTVPHSGNETGPTTVYVETPYPTTVTTTTTVGYPGSVTTTLTGSPSNG<br>TVIDTVEVPTTTNYGYTTITTGYTGSTTLTTTVPHSGNETGPTTVYVETPYPTTVTTTTTVGYPGSVTTTLTGSPSN<br>GTVIDTVEVPTTTNYGYTTITTGYTGSTTLTTTVPHSGNETGPTTVYVETPYPTTVTTTTTVGYPGSVTTTLTGSPS<br>NGTVIDTVEVPTTTNYGYTTITTGYTGSTTLTTTVPHSGNETGPTTVYVETPYPTTVTTTTTVGYPGSVTTTLTGSP<br>SNGTVIDTVEVPTTTSYGYTTVTTGYTGTTTLTTTVPHSGNETGPTTVYVETPTISTVSSVSTVTTTTTVGYSGSVT<br>TTLTGSGSNSIVTETVEVPTTTTDYGYTTVTTGYTGSTTLTTTVPHSGNETGPTTVYVETPTISTVSSVSTVTTTTTV<br>GYSGSVTTTLTGSGSNSIVTETVEVPTTTSVSYGYTTITTGWTGSTTLTSIVTHSGSETGPTTVYIETPSVSATTTTTT<br>IGYSGSLTTTLTGSSGPVVTNTVEIPYGNSSYIIPTTIVTGTVTTVTTGYTGTETSTVTVTPTGSTGTTTVVIQTPTTV<br>TATETDIVTVTTGYTGTETSTVTVTPTGTSTGTTTVVIQTPTTVTATETDIVTVTTGYTGTETSTVTVTPTGTSTGT<br>TTVVIQTPTTVTATETDIVTVTTGYTGTETSTVTVTPTGTSTGTTTVVIQTPSTVTATETDIVTVTTGYTGTETSTIT<br>VTPTGAATGTTTVVINTPTTTGSEVVPTTGATGTAGTETQVTTATEVQPTTGATGTAGTETQVTTGTETQATTAT<br>ETQATTATETQATIATEVQTTTGATGTAGTETQVTTGTETQATTATETQATTATETQATTATEVQPTTGATGTAG<br>TETQVTTGTETQATTATEVQPTTGATGTAGTETQVTTATEVQPTTAVTETSSSGYYTTIVSSTVVSTVVPGSTVYP

	VTHVTTTTGASGESSAFTYTTSSTQYEPSTVVTTSYYTTSVYTSAPASETVSSTEAPESSTVTSNPIYQGSGTSTWS<br>TVRQWNGSATYNYTYYTTGGFTGGNNTNVTGLYPSSAGANKPIAYLTFVSLFVYIVTLI
JB873	MSVRRFLSTSARALLFTAALLPSLTSGLPSGNVRILQKGMEPEDYLSSVASQNEVPHDISLPKTELADPNFLVDDM<br>PTLLGRDAAVDPSMFTSTFTVKNGNDANYITASPVSNDASMTAISTFTSGKEASYAIQASPSTFLPDSTTTSGSQVS<br>NAVEASSTFVADTTSTSCNPATVLIVTTSGSTSTSCPPPTTILIVTVPTTTTTTTVGYPGSVTTTLTGTPSNGTVIDTV<br>EVPTTTNYGYTTVTTGYTGSTTLTTTVPHSGNETGPTTVYVETPYPTTVTTTTTVGYPGSVTTTLTGSPSNGTVID<br>TVEVPTTTNYGYTTITTGYTGSTTLTTTVPHSGNETGPTTVYVETPYPTTVTTTTTVGYPGSVTTTLTGSPSNGTVI<br>DTVEVPTTTNYGYTTITTGYTGSTTLTTTVPHSGNETGPTTVYVETPYPTTVTTSTTVGYPGSVTTTLTGSPSNGTV<br>IDTVEVPTTTNYGYTTVTTGYTGTTTLTTTVPHSGNETGPTTVYVETPTSSATTPSTVTITTTVGYPGSVTTTLTGS<br>PSNGTVIDTVEVPTTTNYGYTTVTTGYTGTTTLTTTVPHSGNETGPTTVYVETPTSSATTPSTVTITTTVGYPGSVT<br>TTLTGTPSNGTVIDTVEVPTTTNYGYTTVTTGYTGTTTLTTTVPHSGNETGPTTVYVETPTSSATTPSTVTITTTVG<br>YPGSVTTTLTGCPSNGTVIDTVEVPTTTSYGYTTVTTGYTGTTTLTTTVPHSGNETGPTTVYVETPTISTVSSVSTV<br>TTTTTVGYSGSVTTTLTGSGSNSIVTETVEVPTTTTDYGYTTVTTGYTGSTTLTTTVPHSGNETGPTTVYVETPTIS<br>TVSSVSTVTTTTTVGYSGSVTTTLTGSGSNSIVTETVEVPTTTSVSYGYTTITTGWTGSTTLTSIVTHSGSETGPTTV<br>YIETPSVSATTTTTTIGYSGSLTTTLTGSSGPVVTNTVEIPYGNSSYIIPTTIVTGTVTTVTTGYTGTETSTVTVTPTGS<br>TGTTTVVIQTPTTVTATETDIVTVTTGYTGTETSTVTVTPTGTSTGTTTVVIQTPSTVTATETDIVTVTTGYTGTETS<br>TVTVTPTGTSTGTTTVVIQTPSTVTATETDIVTVTTGYTGTETSTVTVTPTGTSTGTTTVVIQTPSTVTATETDIVTV<br>TTGYTGTETSTITVTPTGAATGTTTVVINTPTTTGSEVVPTTGATGTAGTETQATIATEVQTTTGATGTAGTETQV<br>TTGTETQATTATETQATTATETQATTATEVQPTTGATGTAGTETQVTTGTETQATTATEVQPTTGATGTAGTETQ<br>VTTATEVQPTTAVTETSSSGYYTTIVSSTVVSTVVPGSTVYPVTHVTTTTGASGESSAFTYTTSSTQYEPSTVVTTS<br>YYTTSVYTSAPASETVSSTEAPESSTVTSNPIYQGSGTSTWSTVRQWNGSATYNYTYYTTGGFTGGNNTNVTGLY<br>PSSAGANKPIAYLTFVSLFVYIVTLI
JB874	MSVRRFLSTSARALLFTAALLPSLTSGLPSGNVRILQKGMEPEDYLSSVASQNEVPHDISLPKTELADPNFLVDDMPTLL<br>GRDAAVDPSMFTSTFTVKNGNDANYITASPVSNDASMTAISTFTSGKEASYAIQASPSTFLPDSTTTSGSQVSNAVEASS<br>TFVADTTSTSCNPATVLIVTTSGSTSTSCPPPTTILIVTVPTTTTTTTVGYPGSVTTTLTGTPSNGTVIDTVEVPTTTNYGY<br>TTVTTGYTGSTTLTTTVPHSGNETGPTTVYVETPYPTTVTTTTTVGYPGSVTTTLTGSPSNGTVIDTVEVPTTTNYGYTTI<br>TTGYTGSTTLTTTVPHSGNETGPTTVYVETPYPTTVTTTTTVGYPGSVTTTLTGSPSNGTVIDTVEVPTTTNYGYTTITTG<br>YTGSTTLTTTVPHSGNETGPTTVYVETPYPTTVTTTTTVGYPGSVTTTLTGSPSNGTVIDTVEVPTTTNYGYTTVTTGYT<br>GSTTLTTTVPHSGNETGPTTVYVETPYATTVTTTTTVGYPGSVTTTLTGSPSNGTVIDTVEVPTTTNYGYTTVTTGYTGT<br>TTLTTTVPHSGNETGPTTVYVETPYATTVTTTTTVGYPGSVTTTLTGSPSNGTVIDTVEVPTTTNYGYTTITTGYTGSTT<br>LTTTVPHSGNETGPTTVYVETPYPTTVTTTTTVGYPGSVTTTLTGSPSNGTVIDTVEVPTTTNYGYTTVTTGYTGTTTLT<br>TTVPHSGNETGPTTVYVETPTSSATTPSTVTITTTVGYPGSVTTTLTGSPSNGTVIDTVEVPTTTNYGYTTVTTGYTGTTT<br>LTTTVPHSGNETGPTTVYVETPTISTVSSVSTVTTTTTVGYSGSVTTTLTGSGSNSIVTETVEVPTTTTDYGYTTVTTGYT<br>GSTTLTTTVPHSGNETGPTTVYVETPTISTVSSVSTVTTTTTVGYSGSVTTTLTGSGSNSIVTETVEVPTTTSVSYGYTTIT<br>TGWTGSTTLTSIVTHSGSETGPTTVYIETPSVSATTTTTTIGYSGSLTTTLTGSSGPVVTNTVEIPYGNSSYIIPTTIVTGTV<br>TTVTTGYTGTETSTVTVTPTGSTGTTTVVIQTPTTVTATETDIVTVTTGYTGTETSTVTVTPTGTSTGTTTVVIQTPTTVT

	ATETDIVTVTTGYTGTETSTVTVTPTGTSTGTTTVVIQTPTTVTATETDIVTVTTGYTGTETSTVTVTPTGTSTGTTTVVI<br>QTPSTVTATETDIVTVTTGYTGTETSTVTVTPTGTATGTTTVVINTPTTTGSEVLPTTGATGTAGTETQVTTATEVQPTT<br>GATGTAGTETQVTTGTETQATTATETQATTATETQATTATEVQTTTGATGTAGTETQVTTGTETQATTATETQATTAT<br>ETQATTATEVQPTTGATGTAGTETQVTTGTETQATTATETQATTATEVQPTTGATGTAGTETQVTTATEVQPTTAVTE<br>TSSSGYYTTIVSSTVVSTVVPGSTVYPVTHVTTTTGASGESSAFTYTTSSTQYEPSTVVTTSYYTTSVYTSAPATETVSST<br>EAPESSTVTSNPIYQGSGTSTWSTVRQWNGSATYNYTYYTTGGFTGGNNTNVTGLYPSSAGANKPIAYLTFVSLFVYIV<br>TLI
JB879	MSVRRFLSTSARALLFTAALLPSLTSGLPSGNVRILQKGMEPEDYLSSASQNEVPHDISLPKTELADPNFLVDDMPTLLG<br>RDAAVDPSMFTSTFTVKNGNDANYITASPVSNDASMTAISTFTSGKEASYAIQASPSTFLPDSTTTSGSQVSNAVEASST<br>FVADTTSTSCNPATVLIVTTSGSTSTSCPPPTTILIVTVPTTTTTTTVGYPGSVTTTLTGTPSNGTVIDTVEVPTTTNYGYT<br>TITTGYTGSTTLTTTVPHSGNETGPTTVYVETPYPTTVTTTTTVGYPGSVTTTLTGSPSNGTVIDTVEIPTTTNYGYTTITT<br>GYTGSTTLTTTVPHSGNETGPTTVYVETPYPTTVTTTTTVGYPGSVTTTLTGAPSNGTVIDTVEVPTTTNYGYTTVTTG<br>YTGSTTLTTTVPHSGNETGPTTVYVETPYPTTVTTTTTVGYPGSVTTTLTGAPSNGTVIDTVEVPTTTNYGYTTVTTGYT<br>GSTTLTTTVPHSGNETGPTTVYVETPYPTTVTTTTTVGYPGSVTTTLTGAPSNGTVIDTVEVPTTTNYGYTTVTTGYTGS<br>TTLTTTVPHSGNETGPTTVYVETPYPTTVTTTTTVGYPGSVTTTLTGAPSNGTVIDTVEVPTTTNYGYTTITTGYTGSTT<br>LTTTVPHSGNETGPTTVYVETPYPTTVTTTTTVGYPGSVTTTLTGAPSNGTVIDTVEVPTTTNYGYTTVTTGYTGSTTLT<br>TTVPHSGNETGPTTVYVETPYPTTVTTTTTVGYPGSVTTTLTGAPSNGTVIDTVEIPTTTNYGYTTITTGYTGSTTLTTTV<br>PHSGNETGPTTVYVETPYPTTVTTTTTVGYPGSVTTTLTGAPSNGTVIDTVEVPTTTNYGYTTITTGYTGSTTLTTTVPH<br>SGNETGPTTVYVETPYPTTVTTTTTVGYPGSVTTTLTGAPSNGTVIDTVEVPTTTNYGYTTVTTGYTGSTTLTTTVPHSG<br>NETGPTTVYVETPYPTTVTTTTTVGYPGSVTTTLTGSGSNSIVTETVDVPTTTSVNYGYTTITTGWTGSTTLTSIVTHSGS<br>ETGPTTVYIETPSVSATTTTTTIGYSGSLTTTLTGSSGPVVTNTVEIPYGNSSYIIPTTIVTGTVTTVTTGYTGTETSTVTVIP<br>TGTTGTTTVVIQTPTTVTATETDIVTVTTGYTGTETSTVTVTPTGTSTGTTTVVIQTPTTVTATETDIVTVTTGYTGTETS<br>TVTVTPTGTSTGTTTVVIQTPTTVTATETDIVTVTTGYTGTETSTVTVTPTGTSTGTTTVVIQTPTTVTATETDIVTVTTG<br>YTGTETSTVTVTPTGTSTGTTTVVIQTPTTVTATETDIVTVTTGYTGTETSTVTVTPTGTSTGTTTVVIQTPTTVTATETDI<br>VTVTTGYTGTETSTVTVTPTGTSTGTTTVVIQTPTTVTATETDIVTVTTGYTGTETSTVTVTPTGTATGTTTVVINTPTTT<br>GSEVLPTTGATGTAGTETQLTTATEVQPTTGATGTAGTETQVTTGTETQATTATETQATTATEVQTTTGATGTAGTET<br>QATTATEVQPTTGATGTAGTETQVTTATEVQPTTGATGTAGTETQVTTGTETQATTATETQATTATEVQTTTGATGTA<br>GTETQVTTATEVQPTTAVTETSSSGYYTTIVSSTVVSTVVPGSTVYPVTHVTTTTGVSGESSAFTYTTSSTQYEPSTVVT<br>TSYYTTSVYTSAPATETVSSTEAPESSTVTSNPIYQGSGTSTWSTVRQWNGSATYNYTYYTTGGFTGGNNTNVTGLYPS<br>SAGANKPIAYLTFVSLFVYIVTLI
JB918	MSVRRFLSTSARALLFTAALLPSLTSGLPSGNVRILQKGMEPEDYLSSASQNEVPHDISLPKTELADPNFLVDDMPTLLG<br>RDAAVDPSMFTSTFTVKNGNDANYITASPVSNDASMTAISTFTSGKEASYAIQASPSTFLPDSTTTSGSQVSNAVEASST<br>FVADTTSTSCNPATVLIVTTSGSTSTSCPPPTTILIVTVPTTTTTTTVGYPGSVTTTLTGTPSNGTVIDTVEVPTTTNYGYT<br>TITTGYTGSTTLTTTVPHSGNETGPTTVYVETPYPTTVTTTTTVGHPGSVTTTLTGAPSNGTVIDTVEVPTTTNYGYTTV<br>TTGYTGSTTLTTTVPHSGNETGPTTVYVETPYPTTVTTTTTVGYPGSVTTTLTGSPSNGTVIDTVEIPTTTNYGYTTITTG<br>YTGSTTLTTTVPHSGNETGPTTVYVETPYPTTVTTTTTVGYPGSVTTTLTGSPSNGTVIDTVEIPTTTNYGYTTITTGYTG<br>STTLTTTVPHSGNETGPTTVYVETPYPTTVTTTTTVGHPGSVTTTLTGAPSNGTVIDTVEIPTTTNYGYTTITTGYTGSTT

	LTTTVPHSGNETGPTTVYVETPYPTTVTTTTTVGYSGSVTTTLTGSGSNSIVTETVDVPTTTSVNYGYTTITTGWTGSTT<br>LTSIVTHSGSETGPTTVYIETPSVSATTTTTTIGYSGSLTTTLTGSSGPVVTNTVEIPYGNSSYIIPTTIVTGTVTTVTTGYTG<br>TETSTVTVIPTGTTGTTTVVIQTPTTVTATETDIVTVTTGYTGTETSTVTVTPTGTSTGTTTVVIQTPTTVTATETDIVTVT<br>TGYTGTETSTVTVTPTGTATGTTTVVINTPTTTGSEVLPTTGATGTAGTETQVTTATEVQPTTGATGTAGTETQVTTGT<br>ETQATTATETQATTATEVQTTTGATGTAGTETQATTATEVQPTTGATGTAGTETQATTATEVQTTTGATGTAGTETQV<br>TTATEVQPTTAVTETSSSGYYTTIVSSTVVSTVVPGSTVYPVTHVTTTTGVSGESSAFTYTTSSTQYEPSTVVTTSYYTTS<br>VYTSAPATETVSSTEAPESSTVTSNPIYQGSGTSTWSTVRQWNGSATYNYTYYTTGGFTGGNNTNVTGLYPSSAGANK<br>PIAYLTFVSLFVYIVTLI
JB929	MSVRRFLSTSARALLFTAALLPSLTSGLPSGNVRILQKGMEPEDYLSSVASQNEVPHDISLPKTELADPNFLVDDMPTLL<br>GRDAAVDPSMFTSTFTVKNGNDANYITASPVSNDASMTAISTFTSGKEASYAIQASPSTFLPDSTTTSGSQVSNAVEASS<br>TFVADTTSTSCNPATVLIVTTSGSTSTSCPPPTTILIVTVPTTTTTTTVGYPGSVTTTLTGTPSNGTVIDTVEVPTTTNYGY<br>TTVTTGYTGSTTLTTTVPHSGNETGPTTVYVETPYPTTVTTTTTVGYPGSVTTTLTGSPSNGTVIDTVEVPTTTNYGYTTI<br>TTGYTGSTTLTTTVPHSGNETGPTTVYVETPYPTTVTTTTTVGYPGSVTTTLTGSPSNGTVIDTVEVPTTTNYGYTTITTG<br>YTGSTTLTTTVPHSGNETGPTTVYVETPYPTTVTTTTTVGYPGSVTTTLTGSPSNGTVIDTVEVPTTTNYGYTTITTGYT<br>GSTTLTTTVPHSGNETGPTTVYVETPYPTTVTTTTTVGYPGSVTTTLTGSPSNGTVIDTVEVPTTTNYGYTTITTGYTGST<br>TLTTTVPHSGNETGPTTVYVETPYPTTVTTTTTVGYPGSVTTTLTGSPSNGTVIDTVEVPTTTNYGYTTVTTGYTGSTTL<br>TTTVPHSGNETGPTTVYVETPYPTTVTTTTTVGYPGSVTTTLTGSPSNGTVIDTVEVPTTTNYGYTTVTTGYTGTTTLTT<br>TVPHSGNETGPTTVYVETPYATTVTTTTTVGYPGSVTTTLTGSPSNGTVIDTVEVPTTTNYGYTTITTGYTGSTTLTTTV<br>PHSGNETGPTTVYVETPYPTTVTTTTTVGYPGSVTTTLTGSPSNGTVIDTVEVPTTTNYGYTTVTTGYTGTTTLTTTVPH<br>SGNETGPTTVYVETPTSSATTPSTVTITTTVGYPGSVTTTLTGSPSNGTVIDTVEVPTTTNYGYTTVTTGYTGTTTLTTTV<br>PHSGNETGPTTVYVETPTISTVSSVSTVTTTTTVGYSGSVTTTLTGSGSNSIVTETVEVPTTTTDYGYTTVTTGYTGSTTL<br>TTTVPHSGNETGPTTVYVETPTISTVSSVSTVTTTTTVGYSGSVTTTLTGSGSNSIVTETVEVPTTTSVSYGYTTITTGWT<br>GSTTLTSIVTHSGSETGPTTVYIETPSVSATTTTTTIGYSGSLTTTLTGSSGPVVTNTVEIPYGNSSYIIPTTIVTGTVTTVTT<br>GYTGTETSTVTVTPTGSTGTTTVVIQTPTTVTATETDIVTVTTGYTGTETSTVTVTPTGTSTGTTTVVIQTPTTVTATETD<br>IVTVTTGYTGTETSTVTVTPTGTSTGTTTVVIQTPTTVTATETDIVTVTTGYTGTETSTVTVTPTGTSTGTTTVVIQTPSTV<br>TATETDIVTVTTGYTGTETSTVTVTPTGTATGTTTVVINTPTTTGSEVLPTTGATGTAGTETQVTTATEVQPTTGATGTA<br>GTETQVTTGTETQATTATETQATTATETQATTATEVQTTTGATGTAGTETQVTTGTETQATTATETQATTATETQATT<br>ATEVQPTTGATGTAGTETQVTTGTETQATTATETQATTATEVQPTTGATGTAGTETQVTTATEVQPTTAVTETSSSGY<br>YTTIVSSTVVSTVVPGSTVYPVTHVTTTTGASGESSAFTYTTSSTQYEPSTVVTTSYYTTSVYTSAPATETVSSTEAPESS<br>TVTSNPIYQGSGTSTWSTVRQWNGSATYNYTYYTTGGFTGGNNTNVTGLYPSSAGANKPIAYLTFVSLFVYIVTLI*
JB938	MSVRRFLSTSARALLFTAALLPSLTSGLPSGNVRILQKGMEPEDYLSSASQNEVPHDISLPKTELADPNFLVDDMPTLLG<br>RDAAVDPSMFTSTFTVKNGNDANYITASPVSNDASMTAISTFTSGKEASYAIQASPSTFLPDSTTTSGSQVSNAVEASST<br>FVADTTSTSCNPATVLIVTTSGSTSTSCPPPTTILIVTVPTTTTTTTVGYPGSVTTTLTGTPSNGTVIDTVEVPTTTNYGYT<br>TITTGYTGSTTLTTTVPHSGNETGPTTVYVETPYPTTVTTTTTVGYPGSVTTTLTGSPSNGTVIDTVEIPTTTNYGYTTITT<br>GYTGSTTLTTTVPHSGNETGPTTVYVETPYPTTVTTTTTVGHPGSVTTTLTGAPSNGTVIDTVEVPTTTNYGYTTVTTG<br>YTGSTTLTTTVPHSGNETGPTTVYVETPYPTTVTTTTTVGYPGSVTTTLTGSPSNGTVIDTVEIPTTTNYGYTTITTGYTG<br>STTLTTTVPHSGNETGPTTVYVETPYPTTVTTTTTVGYPGSVTTTLTGSPSNGTVIDTVEIPTTTNYGYTTITTGYTGSTT

	LTTTVPHSGNETGPTTVYVETPYPTTVTTTTTVGYPGSVTTTLTGAPSNGTVIDTVEVPTTTNYGYTTVTTGYTGSTTLT<br>TTVPHSGNETGPTTVYVETPYPTTVTTTTTVGYPGSVTTTLTGAPSNGTVIDTVEIPTTTNYGYTTITTGYTGSTTLTTTV<br>PHSGNETGPTTVYVETPYPTTVTTTTTVGYPGSVTTTLTGSPSNGTVIDTVEIPTTTNYGYTTITTGYTGSTTLTTTVPHS<br>GNETGPTTVYVETPYPTTVTTTTTVGYPGSVTTTLTGSPSNGTVIDTVEIPTTTNYGYTTITTGYTGSTTLTTTVPHSGNE<br>TGPTTVYVETPYPTTVTTTTTVGYPGSVTTTLTGAPSNGTVIDTVEVPTTTNYGYTTVTTGYTGSTTLTTTVPHSGNETG<br>PTTVYVETPYPTTVTTTTTVGYPGSVTTTLTGAPSNGTVIDTVEIPTTTNYGYTTITTGYTGSTTLTTTVPHSGNETGPTT<br>VYVETPYPTTVTTTTTVGYPGSVTTTLTGAPSNGTVIDTVEVPTTTNYGYTTVTTGYTGSTTLTTTVPHSGNETGPTTV<br>YVETPYPTTVTTTTTVGYSGSVTTTLTGSGSNSIVTETVDVPTTTSVNYGYTTITTGWTGSTTLTSIVTHSGSETGPTTVY<br>IETPSVSATTTTTTIGYSGSLTTTLTGSSGPVVTNTVEIPYGNSSYIIPTTIVTGTVTTVTTGYTGTETSTVTVIPTGTTGTTT<br>VVIQTPTTVTATETDIVTVTTGYTGTETSTVTVTPTGTSTGTTTVVIQTPTTVTATETDIVTVTTGYTGTETSTVTVTPTG<br>TSTGTTTVVIQTPTTVTATETDIVTVTTGYTGTETSTVTVTPTGTSTGTTTVVIQTPTTVTATETDIVTVTTGYTGTETST<br>VTVTPTGTATGTTTVVINTPTTTGSEVLPTTGATGTAGTETQLTTATEVQPTTGATGTAGTETQVTTGTETQATTATET<br>QATTATEVQTTTGATGTAGTETQATTATEVQPTTGATGTAGTETQVTTATEVQPTTGATGTAGTETQVTTGTETQATT<br>ATETQATTATEVQTTTGATGTAGTETQVTTATEVQPTTAVTETSSSGYYTTIVSSTVVSTVVPGSTVYPVTHVTTTTGV<br>SGESSAFTYTTSSTQYEPSTVVTTSYYTTSVYTSAPATETVSSTEAPESSTVTSNPIYQGSGTSTWSTVRQWNGSATYNY<br>TYYTTGGFTGGNNTNVTGLYPSSAGANKPIAYLTFVSLFVYIVTLI
JB939	MSVRRFLSTSARALLFTAALLPSLTSGLPSGNVRILQKGMEPEDYLSSVASQNEVPHDISLPKTELADPNFLVDDMPTLL<br>GRDAAVDPSMFTSTFTVKNGNDANYITASPVSNDASMTAISTFTSGKEASYAIQASPSTFLPDSTTTSGSQVSNAVEASS<br>TFVADTTSTSCNPATVLIVTTSGSTSTSCPPPTTILIVTVPTTTTTTTVGYPGSVTTTLTGTPSNGTVIDTVEVPTTTNYGY<br>TTVTTGYTGSTTLTTTVPHSGNETGPTTVYVETPYPTTVTTTTTVGYPGSVTTTLTGTPSNGTVIDTVEVPTTTNYGYTT<br>VTTGYTGSTTLTTTVPHSGNETGPTTVYVETPYPTTVTTTTTVGYPGSVTTTLTGSPSNGTVIDTVEVPTTTNYGYTTITT<br>GYTGTTTLTTTVPHSGNETGPTTVYVETPYPTTVTTTTTVGYPGSVTTTLTGSPSNGTVIDTVEVPTTTNYGYTTITTGY<br>TGSTTLTTTVPHSGNETGPTTVYVETPYPTTVTTTTTVGYPGSVTTTLTGSPSNGTVIDTVEVPTTTNYGYTTITTGYTGS<br>TTLTTTVPHSGNETGPTTVYVETPYPTTVTTSTTVGYPGSVTTTLTGSPSNGTVIDTVEVPTTTNYGYTTVTTGYTGTTT<br>LTTTVPHSGNETGPTTVYVETPTSSATTPSTVTITTTVGYPGSVTTTLTGSPSNGTVIDTVEVPTTTNYGYTTVTTGYTGT<br>TTLTTTVPHSGNETGPTTVYVETPTSSATTPSTVTITTTVGYPGSVTTTLTGTPSNGTVIDTVEVPTTTNYGYTTVTTGYT<br>GTTTLTTTVPHSGNETGPTTVYVETPTSSATTPSTVTITTTVGYPGSVTTTLTGSPSNGTVIDTVEVPTTTSYGYTTVTTG<br>YTGTTTLTTTVPHSGNETGPTTVYVETPTISTVSSVSTVTTTTTVGYSGSVTTTLTGSGSNSIVTETVEVPTTTSVSYGYT<br>TVTTGYTGSTTLTTTVPHSGNETGPTTVYVETPTISTVSSVSTVTTTTTVGYSGSVTTTLTGSGSNSIVTETVEVPTTTSVS<br>YGYTTITTGWTGSTTLTSIVTHSGSETGPTTVYIETPSVSATTTTTTIGYSGSLTTTLTGSSGPVVTNTVEIPYGNSSYIIPT<br>TIVTGTVTTVTTGYTGTETSTVTVTPTGTSTGTTTVVIQTPTTVTATETDIVTVTTGYTGTETSTVTVTPTGTSTGTTTVV<br>IQTPTTVTATETDIVTVTTGYTGTETSTVTVTPTGTSTGTTTVVIQTPTTVTATETDIVTVTTGYTGTETSTVTVTPTGTST<br>GTTTVVIQTPSTVTATETDIVTVTTGYTGTETSTVTVTPTGTSTGTTTVVIQTPSTVTATETDIVTVTTGYTGTETSTITVT<br>PTGAATGTTTVVINTPTTTGSEVVPTTGATGTAGTETQVTTATEVQPTTGATGTAGTETQVTTGTETQATTATETQATT<br>ATETQATIATEVQTTTGATGTAGTETQVTTGTETQATTATETQATTATETQATTATEVQPTTGATGTAGTETQVTTGTE<br>TQATTATEVQPTTGATGTAGTETQVTTATEVQLTTAVTETSSSGYYTTIVSSTVVSTVVPGSTVYPVTHVTTTTGASGE

	SSAFTYTTSSTQYEPSTVVTTSYYTTSVYTSAPASETVSSTEAPESSTVTSNPIYQGSGTSTWSTVRQWNGSATYNYTYY<br>TTGGFTGGNNTNVTGLYPSSAGANKPIAYLTFVSLFVYIVTLI
JB943	MSVRRFLSTSARALLFTAALLPSLTSGLPSGNVRILQKGMEPEDYLSSVASQNEVPHDISLPKTELADPNFLVDDMPTLL<br>GRDAAVDPSMFTSTFTVKNGNDANYITASPVSNDASMTAISTFTSGKEASYAIQASPSTFLPDSTTTSGSQVSNAVEASS<br>TFVADTTSTSCNPATVLIVTTSGSTSTSCPPPTTILIVTVPTTTTTTTVGYPGSVTTTLTGTPSNGTVIDTVEVPTTTNYGY<br>TTVTTGYTGSTTLTTTVPHSGNETGPTTVYVETPYPTTVTTTTTVGYPGSVTTTLTGSPSNGTVIDTVEVPTTTNYGYTTI<br>TTGYTGSTTLTTTVPHSGNETGPTTVYVETPYPTTVTTTTTVGYPGSVTTTLTGSPSNGTVIDTVEVPTTTNYGYTTITTG<br>YTGSTTLTTTVPHSGNETGPTTVYVETPYPTTVTTTTTVGYPGSVTTTLTGSPSNGTVIDTVEVPTTTNYGYTTITTGYT<br>GSTTLTTTVPHSGNETGPTTVYVETPYPTTVTTSTTVGYPGSVTTTLTGSPSNGTVIDTVEVPTTTNYGYTTVTTGYTGT<br>TTLTTTVPHSGNETGPTTVYVETPTSSATTPSTVTITTTVGYPGSVTTTLTGSPSNGTVIDTVEVPTTTNYGYTTVTTGYT<br>GTTTLTTTVPHSGNETGPTTVYVETPTSSATTPSTVTITTTVGYPGSVTTTLTGTPSNGTVIDTVEVPTTTNYGYTTVTTG<br>YTGTTTLTTTVPHSGNETGPTTVYVETPTSSATTPSTVTITTTVGYPGSVTTTLTGCPSNGTVIDTVEVPTTTSYGYTTVT<br>TGYTGTTTLTTTVPHSGNETGPTTVYVETPTISTVSSVSTVTTTTTVGYSGSVTTTLTGSGSNSIVTETVEVPTTTTDYGY<br>TTVTTGYTGTTTLTTTVPHSGNETGPTTVYVETPTISTVSSVSTVTTTTTVGYSGSVTTTLTGSGSNSIVTETVEVPTTTS<br>VSYGYTTITTGWTGSTTLTSIVTHSGSETGPTTVYIETPSVSATTTTTTIGYSGSLTTTLTGSSGPVVTNTVEIPYGNSSYII<br>PTTIVTGTVTTVTTGYTGTETSTVTVTPTGSTGTTTVVIQTPTTVTATETDIVTVTTGYTGTETSTVTVTPTGTSTGTTTV<br>VIQTPSTVTATETDIVTVTTGYTGTETSTVTVTPTGTSTGTTTVVIQTPSTVTATETDIVTVTTGYTGTETSTVTVTPTGTS<br>TGTTTVVIQTPSTVTATETDIVTVTTGYTGTETSTITVTPTGAATGTTTVVINTPTTTGSEVVPTTGATGTAGTETQATIA<br>TEVQTTTGATGTAGTETQVTTGTETQATTATETQATTATETQATTATEVQPTTGATGTAGTETQVTTGTETQATTATE<br>VQPTTGATGTAGTETQVTTATEVQPTTAVTETSSSGYYTTIVSSTVVSTVVPGSTVYPVTHVTTTTGASGESSAFTYTTS<br>STQYEPSTVVTTSYYTTSVYTSAPASETVSSTEAPESSTVTSNPIYQGSGTSTWSTVRQWNGSATYNYTYYTTGGFTGG<br>NNTNVTGLYPSSAGANKPIAYLTFVSLFVYIVTLI
JB1110	MSVRRFLSTSARALLFTAALLPSLTSGLPSGNVRILQKGMEPEDYLSSVASQNEVPHDISLPKTELADPNFLVDDMPTLL<br>GRDAAVDPSMFTSTFTVKNGNDANYITASPVSNDASMTAISTFTSGKEASYAIQASPSTFLPDSTTTSGSQVSNAVEASS<br>TFVADTTSTSCNPATVLIVTTSGSTSTSCPPPTTILIVTVPTTTTTTTVGYPGSVTTTLTGTPSNGTVIDTVEVPTTTNYGY<br>TTVTTGYTGSTTLTTTVPHSGNETGPTTVYVETPYPTTVTTTTTVGYPGSVTTTLTGSPSNGTVIDTVEVPTTTNYGYTT<br>VTTGYTGTTTLTTTVPHSGNETGPTTVYVETPYPTTVTTTTTVGYPGSVTTTLTGSPSNGTVIDTVEVPTTTNYGYTTIT<br>TGYTGSTTLTTTVPHSGNETGPTTVYVETPYPTTVTTTTTVGYPGSVTTTLTGSPSNGTVIDTVEVPTTTNYGYTTITTG<br>YTGSTTLTTTVPHSGNETGPTTVYVETPYPTTVTTTTTVGYPGSVTTTLTGSPSNGTVIDTVEVPTTTNYGYTTITTGYT<br>GSTTLTTTVPHSGNETGPTTVYVETPYPTTVTTTTTVGYPGSVTTTLTGSPSNGTVIDTVEVPTTTNYGYTTITTGYTGST<br>TLTTTVPHSGNETGPTTVYVETPYPTTVTTTTTVGYPGSVTTTLTGSPSNGTVIDTVEVPTTTNYGYTTITTGYTGSTTLT<br>TTVPHSGNETGPTTVYVETPYPTTVTTTTTVGYPGSVTTTLTGSPSNGTVIDTVEVPTTTNYGYTTITTGYTGSTTLTTTV<br>PHSGNETGPTTVYVETPYPTTVTTTTTVGYPGSVTTTLTGTPSNGTVIDTVEVPTTTNYGYTTVTTGYTGTTTLTTTVPH<br>SGNETGPTTVYVETPTISTVSSVSTVTTTTTVGYSGSVTTTLTGSGSNSIVTETVEVPTTTSVSYGYTTITTGWTGSTTLTS<br>IVTHSGSETGPTTVYIETPSVSATTTTTTIGYSGSLTTTLTGSSGPVVTNTVEIPYGNSSYIIPTAIVTGTVTTVTTGYTGTE<br>TSTVTVTPTGSTGTTTVVIQTPTTVTATETDIVTVTTGYTGTETSTVTVTPTGTSTGTTTVVIQTPTTVTATETDIVTVTT<br>GYTGTETSTVTVTPTGTSTGTTTVVIQTPTTVTATETDIVTVTTGYTGTETSTVTVTPTGTSTGTTTVVIQTPTTVTATET

	DIVTVTTGYTGTETSTVTVIPTGTSTGTTTVVIQTPTTVTATETDIVTVTTGYTGTETSTVTVIPTGTSTGTTTVVIQTPTT<br>VTATETDIVTVTTGYTGTETSTITVTPTGAATGTTTVVINTPTTTGSEVVPTTGATGTAGTETQVTTGTETQATTATETQ<br>ATTATETQATIATEVQTTTGATGTAGTETQVTTGTETQATTATETQATTATETQATTATEVQPTTGATGTAGTETQVTT<br>ATEVQPTTAVTETSSSGYYTTIVSSTVVSTVVPGSTVYPVTHVTTTTGASGESSAFTYTTSSTQYEPSTVVTTSYYTTSV<br>YTSAPASETVSSTEAPESSTVTSNPIYQGSGTSTWSTVRQWNGSATYNYTYYTTGGLTGGNNTNVTGLYPSSAGANKPI<br>AYLTFVSLFVYIVTLI
JB1171	MSVRRFLSTSARALLFTAALLPSLTSGLPSGNVRILQKGMEPEDYLSSVASQNEVPHDISLPKTELADPNFLVDDMPTLL<br>GRDAAVDPSMFTSTFTVKNGNDANYITASPVSNDASMTAISTFTSGKEASYAIQASPSTFLPDSTTTSGSQVSNAVEASS<br>TFVADTTSTSCNPATVLIVTTSGSTSTSCPPPTTILIVTVPTTTTTTTVGYPGSVTTTLTGTPSNGTVIDTVEVPTTTNYGY<br>TTVTTGYTGSTTLTTTVPHSGNETGPTTVYVETPYPTTVTTTTTVGYPGSVTTTLTGSPSNGTVIDTVEVPTTTNYGYTTI<br>TTGYTGSTTLTTTVPHSGNETGPTTVYVETPYPTTVTTTTTVGYPGSVTTTLTGSPSNGTVIDTVEVPTTTNYGYTTITTG<br>YTGSTTLTTTVPHSGNETGPTTVYVETPYPTTVTTTTTVGYPGSVTTTLTGSPSNGTVIDTVEVPTTTNYGYTTVTTGYT<br>GSTTLTTTVPHSGNETGPTTVYVETPYPTTVTTTTTVGYPGSVTTTLTGSPSNGTVIDTVEVPTTTNYGYTTVTTGYTGT<br>TTLTTTVPHSGNETGPTTVYVETPYATTVTTTTTVGYPGSVTTTLTGSPSNGTVIDTVEVPTTTNYGYTTITTGYTGSTT<br>LTTTVPHSGNETGPTTVYVETPYPTTVTTTTTVGYPGSVTTTLTGSPSNGTVIDTVEVPTTTNYGYTTVTTGYTGTTTLT<br>TTVPHSGNETGPTTVYVETPTSSATTPSTVTITTTVGYPGSVTTTLTGSPSNGTVIDTVEVPTTTNYGYTTVTTGYTGTTT<br>LTTTVPHSGNETGPTTVYVETPTISTVSSVSTVTTTTTVGYSGSVTTTLTGSGSNSIVTETVEVPTTTTDYGYTTVTTGYT<br>GSTTLTTTVPHSGNETGPTTVYVETPTISTVSSVSTVTTTTTVGYSGSVTTTLTGSGSNSIVTETVEVPTTTSVSYGYTTIT<br>TGWTGSTTLTSIVTHSGSETGPTTVYIETPSVSATTTTTTIGYSGSLTTTLTGSSGPVVTNTVEIPYGNSSYIIPTTIVTGTV<br>TTVTTGYTGTETSTVTVTPTGSTGTTTVVIQTPTTVTATETDIVTVTTGYTGTETSTVTVTPTGTSTGTTTVVIQTPTTVT<br>ATETDIVTVTTGYTGTETSTVTVTPTGTSTGTTTVVIQTPTTVTATETDIVTVTTGYTGTETSTVTVTPTGTSTGTTTVVI<br>QTPSTVTATETDIVTVTTGYTGTETSTVTVTPTGTATGTTTVVINTPTTTGSEVLPTTGATGTAGTETQVTTATEVQPTT<br>GATGTAGTETQVTTGTETQATTATETQATTATETQATTATEVQTTTGATGTAGTETQVTTGTETQATTATETQATTAT<br>ETQATTATEVQPTTGATGTAGTETQVTTGTETQATTATETQATTATEVQPTTGATGTAGTETQVTTATEVQPTTAVTE<br>TSSSGYYTTIVSSTVVSTVVPGSTVYPVTHVTTTTGASGESSAFTYTTSSTQYEPSTVVTTSYYTTSVYTSAPATETVSST<br>EAPESSTVTSNPIYQGSGTSTWSTVRQWNGSATYNYTYYTTGGFTGGNNTNVTGLYPSSAGANKPIAYLTFVSLFVYIV<br>TLI
JB1174	MSVRRFLSTSARALLFTAALLPSLTSGLPSGNVRILQKGMEPEDYLSSVASQNEVPHDISLPKTELADPNFLVDDMPTLL<br>GRDAAVDPSMFTSTFTVKNGNDANYITASPVSNDASMTAISTFTSGKEASYAIQASPSTFLPDSTTTSGSQVSNAVEASS<br>TFVADTTSTSCNPATVLIVTTSGSTSTSCPPPTTILIVTVPTTTTTTTVGYPGSVTTTLTGTPSNGTVIDTVEVPTTTNYGY<br>TTVTTGYTGSTTLTTTVPHSGNETGPTTVYVETPYPTTVTTTTTVGYPGSVTTTLTGSPSNGTVIDTVEVPTTTNYGYTTI<br>TTGYTGSTTLTTTVPHSGNETGPTTVYVETPYPTTVTTTTTVGYPGSVTTTLTGSPSNGTVIDTVEVPTTTNYGYTTITTG<br>YTGSTTLTTTVPHSGNETGPTTVYVETPYPTTVTTTTTVGYPGSVTTTLTGSPSNGTVIDTVEVPTTTNYGYTTVTTGYT<br>GSTTLTTTVPHSGNETGPTTVYVETPYPTTVTTTTTVGYPGSVTTTLTGSPSNGTVIDTVEVPTTTNYGYTTVTTGYTGT<br>TTLTTTVPHSGNETGPTTVYVETPYATTVTTTTTVGYPGSVTTTLTGSPSNGTVIDTVEVPTTTNYGYTTITTGYTGSTT<br>LTTTVPHSGNETGPTTVYVETPYPTTVTTTTTVGYPGSVTTTLTGSPSNGTVIDTVEVPTTTNYGYTTVTTGYTGTTTLT<br>TTVPHSGNETGPTTVYVETPTSSATTPSTVTITTTVGYPGSVTTTLTGSPSNGTVIDTVEVPTTTNYGYTTVTTGYTGTTT

	LTTTVPHSGNETGPTTVYVETPTISTVSSVSTVTTTTTVGYSGSVTTTLTGSGSNSIVTETVEVPTTTTDYGYTTVTTGYT<br>GSTTLTTTVPHSGNETGPTTVYVETPTISTVSSVSTVTTTTTVGYSGSVTTTLTGSGSNSIVTETVEVPTTTSVSYGYTTIT<br>TGWTGSTTLTSIVTHSGSETGPTTVYIETPSVSATTTTTTIGYSGSLTTTLTGSSGPVVTNTVEIPYGNSSYIIPTTIVTGTV<br>TTVTTGYTGTETSTVTVTPTGSTGTTTVVIQTPTTVTATETDIVTVTTGYTGTETSTVTVTPTGTSTGTTTVVIQTPTTVT<br>ATETDIVTVTTGYTGTETSTVTVTPTGTSTGTTTVVIQTPTTVTATETDIVTVTTGYTGTETSTVTVTPTGTSTGTTTVVI<br>QTPSTVTATETDIVTVTTGYTGTETSTVTVTPTGTATGTTTVVINTPTTTGSEVLPTTGATGTAGTETQVTTATEVQPTT<br>GATGTAGTETQVTTGTETQATTATETQATTATETQATTATEVQTTTGATGTAGTETQVTTGTETQATTATETQATTAT<br>ETQATTATEVQPTTGATGTAGTETQVTTGTETQATTATETQATTATEVQPTTGATGTAGTETQVTTATEVQPTTAVTE<br>TSSSGYYTTIVSSTVVSTVVPGSTVYPVTHVTTTTGASGESSAFTYTTSSTQYEPSTVVTTSYYTTSVYTSAPATETVSST<br>EAPESSTVTSNPIYQGSGTSTWSTVRQWNGSATYNYTYYTTGGFTGGNNTNVTGLYPSSAGANKPIAYLTFVSLFVYIV<br>TLI
JB1180	MSVRRFLSTSARALLFTAALLPSLTSGLPSGNVRILQKGMEPEDYLSSVASQNEVPHDISLPKTELADPNFLVDDMPTLL<br>GRDAAVDPSMFTSTFTVKNGNDANYITASPVSNDASMTAISTFTSGKEASYAIQASPSTFLPDSTTTSGSQVSNAVEASS<br>TFVADTTSTSCNPATVLIVTTSGSTSTSCPPPTTILIVTVPTTTTTTTVGYPGSVTTTLTGTPSNGTVIDTVEVPTTTNYGY<br>TTVTTGYTGSTTLTTTVPHSGNETGPTTVYVETPYPTTVTTTTTVGYPGSVTTTLTGSPSNGTVIDTVEVPTTTNYGYTTI<br>TTGYTGSTTLTTTVPHSGNETGPTTVYVETPYPTTVTTTTTVGYPGSVTTTLTGSPSNGTVIDTVEVPTTTNYGYTTITTG<br>YTGSTTLTTTVPHSGNETGPTTVYVETPYPTTVTTTTTVGYPGSVTTTLTGSPSNGTVIDTVEVPTTTNYGYTTITTGYT<br>GSTTLTTTVPHSGNETGPTTVYVETPYPTTVTTSTTVGYPGSVTTTLTGSPSNGTVIDTVEVPTTTNYGYTTVTTGYTGT<br>TTLTTTVPHSGNETGPTTVYVETPTSSATTPSTVTITTTVGYPGSVTTTLTGSPSNGTVIDTVEVPTTTNYGYTTVTTGYT<br>GTTTLTTTVPHSGNETGPTTVYVETPTSSATTPSTVTITTTVGYPGSVTTTLTGTPSNGTVIDTVEVPTTTNYGYTTVTTG<br>YTGTTTLTTTVPHSGNETGPTTVYVETPTSSATTPSTVTITTTVGYPGSVTTTLTGCPSNGTVIDTVEVPTTTSYGYTTVT<br>TGYTGTTTLTTTVPHSGNETGPTTVYVETPTISTVSSVSTVTTTTTVGYSGSVTTTLTGSGSNSIVTETVEVPTTTTDYGY<br>TTVTTGYTGSTTLTTTVPHSGNETGPTTVYVETPTISTVSSVSTVTTTTTVGYSGSVTTTLTGSGSNSIVTETVEVPTTTS<br>VSYGYTTITTGWTGSTTLTSIVTHSGSETGPTTVYIETPSVSATTTTTTIGYSGSLTTTLTGSSGPVVTNTVEIPYGNSSYII<br>PTTIVTGTVTTVTTGYTGTETSTVTVTPTGSTGTTTVVIQTPTTVTATETDIVTVTTGYTGTETSTVTVTPTGTSTGTTTV<br>VIQTPSTVTATETDIVTVTTGYTGTETSTVTVTPTGTSTGTTTVVIQTPSTVTATETDIVTVTTGYTGTETSTITVTPTGAA<br>TGTTTVVINTPTTTGSEVVPTTGATGTAGTETQATIATEVQTTTGATGTAGTETQVTTGTETQATTATETQATTATETQ<br>ATTATEVQPTTGATGTAGTETQVTTGTETQATTATEVQPTTGATGTAGTETQVTTATEVQPTTAVTETSSSGYYTTIVS<br>STVVSTVVPGSTVYPVTHVTTTTGASGESSAFTYTTSSTQYEPSTVVTTSYYTTSVYTSAPASETVSSTEAPESSTVTSNP<br>IYQGSGTSTWSTVRQWNGSATYNYTYYTTGGFTGGNNTNVTGLYPSSAGANKPIAYLTFVSLFVYIVTLI

Appendix Table S2. Primers used in this study

Primer name	Oligo Sequence (5'-3')	Application
*gsf2*Δ_up_F	GCGGTATGTTCACTAGTATCTTAG	The forward primer used to amplify the upstream homologous arm of the *gsf2* gene for generating the *gsf2* knockout strains
*gsf2*Δ_up_R	GTATTCTGGGCCTCCATGTCAACAAACGATCATGAGGTGTT	The forward primer used to amplify the upstream homologous arm of the *gsf2* gene for generating the *gsf2* knockout strains
*gsf2*Δ_dw_F	GAATGCTGGTCGCTATACTGTGTTGATATATTCAAAAACGGGTAAG	The forward primer used to amplify the upstream homologous arm of the *gsf2* gene for generating the *gsf2* knockout strains
*gsf2*Δ_dw_R	CTAGGATTTCGGGTTGAAATTG	The forward primer used to amplify the upstream homologous arm of the *gsf2* gene for generating the *gsf2* knockout strains
TEF_F	GACATGGAGGCCCAGAATACCCTC	The forward primer used to amplify the HpHMX or KanMX selection tag from the plasmid DNA
TEF_R	CAGTATAGCGACCAGCATTC	The reverse primer used to amplify the HpHMX or KanMX selection tag from the plasmid DNA
nmt41p_F	TCGCCATAAAAGACAGAATAAGTC	The forward primer used to amplify the nmt41 promoter from the plasmid DNA
nmt41p_R	TGATTTAACAAAGCGACTATAAGTCAG	The reverse primer used to amplify the nmt41 promoter from the plasmid DNA
*gsf2IE*_up_F	CAACATGTGCCTAGGCAT	The forward primer used to amplify the upstream homologous arm of the *gsf2* gene for generating the inducible expression strain *gsf2IE*
gsf2^{+}_up_R	TATTCTGGGCCTCCATGTCACCAGGTGTTGTCAATCTG	The reverse primer used to amplify the upstream homologous arm of the *gsf2* gene for generating the inducible expression strain *gsf2*$^{+}$
gsf2^{+}_dw_F	ATAGTCGCTTTGTTAAATCAATGTCTGTTAGAAGGTTTTTATCCA	The forward primer used to amplify the downstream homologous arm of the *gsf2* gene for generating the inducible expression strain *gsf2*$^{+}$
*gsf2IE*_dw_R	CTAATAGCAGTCATGGAAGCG	The reverse primer used to amplify the downstream homologous arm of the *gsf2* gene for generating the inducible expression strain *gsf2IE*
mCherry_F	AGGTGCCTTCGCTTTTCTTTATGTTGAGCAAGGGCGAG	The forward primer used to amplify the mCherry protein gene
mCherry_R	TTTTCGAAATAGTTGAATACTTACTTGTACAGCTCGTCCATG	The reverse primer used to amplify the mCherry protein gene

GFP_F	GTGAATGCTGGTCGCTATACTGGCCC TACAACAACTAAGAAAATG	The forward primer used to amplify the GFP protein gene
GFP_R	GCAAATTAAAGCCTTCGAGCG	The reverse primer used to amplify the GFP protein gene
qPCR_ *gsf2*_F	ACTTCCAAAGACCGAGCTTG	The forward primer of *gsf2* used in qPCR to quantify the expression levels of the *gsf2* gene
qPCR_*gsf2*_R	ATGGAAGCGTCGTTGCTAAC	The reverse primer of *gsf2* used in qPCR to quantify the expression levels of the *gsf2* gene
qPCR_actin_F	CTTTCTACAACGAGCTTCGTGTTG	The forward primer of *Act1* used in qPCR to quantify the expression levels of the *Act1* gene
qPCR_actin_R	GAGTCATCTTCTCACGGTTGGAT	The reverse primer of *Act1* used in qPCR to quantify the expression levels of the *Act1* gene
