# Supplementary figures and images for: A gradient green-beard gene in fission yeast (part 1 of 2)
Source: EMBO Rep. 2026 Mar 16;27(8):1904–17. doi: 10.1038/s44319-026-00748-x (PMC13121626; doi:10.1038/s44319-026-00748-x)

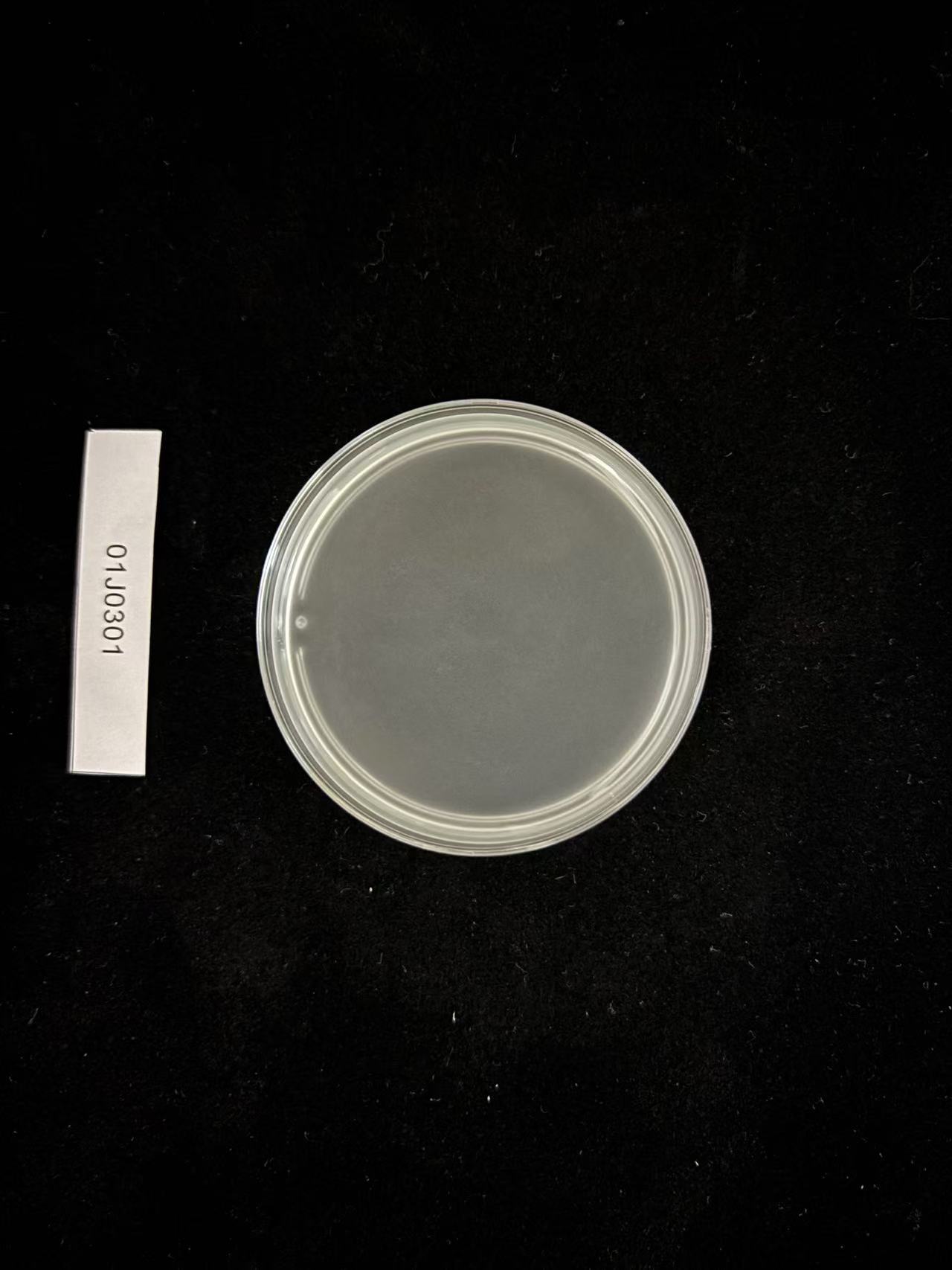

Supplement: Supplementary file 3 — Source data Fig. 1 [file 44319_2026_748_MOESM3_ESM.zip › Figure 1/1F/YE_gsf2IE.jpg]

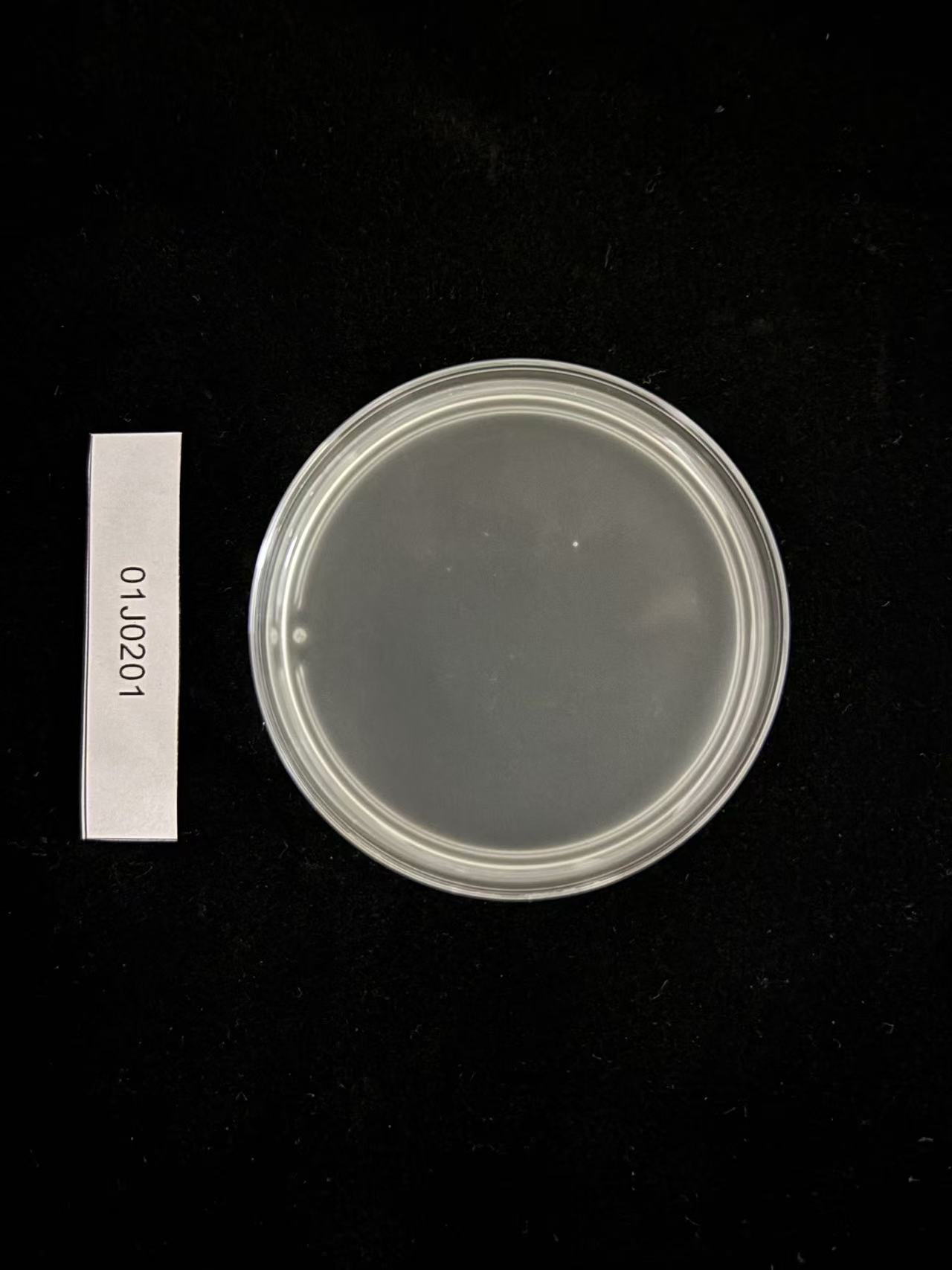

Supplement: Supplementary file 3 — Source data Fig. 1 [file 44319_2026_748_MOESM3_ESM.zip › Figure 1/1F/YE_gsf2Γêå.jpg]

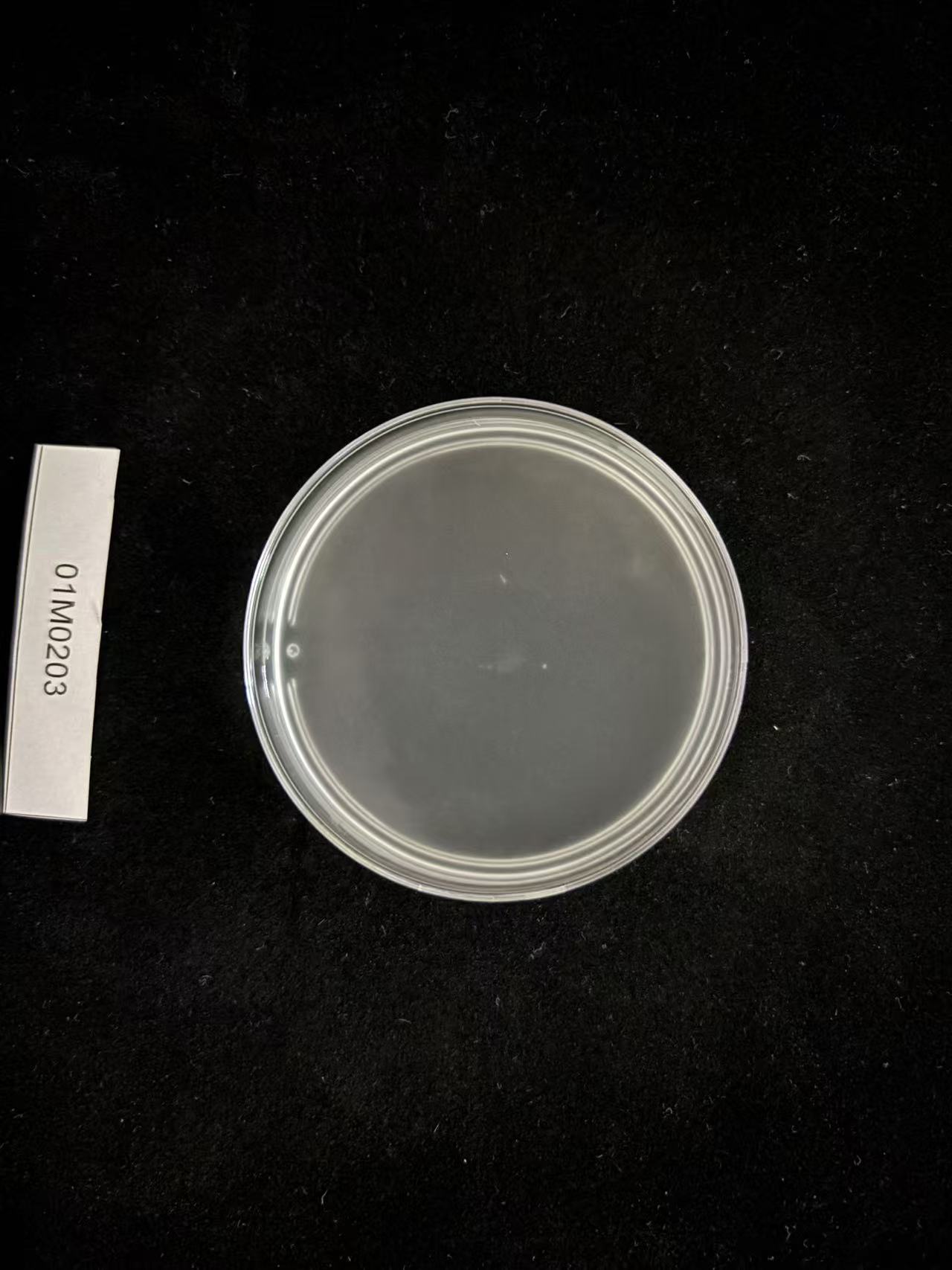

Supplement: Supplementary file 3 — Source data Fig. 1 [file 44319_2026_748_MOESM3_ESM.zip › Figure 1/1F/EMM_gsf2Γêå.jpg]

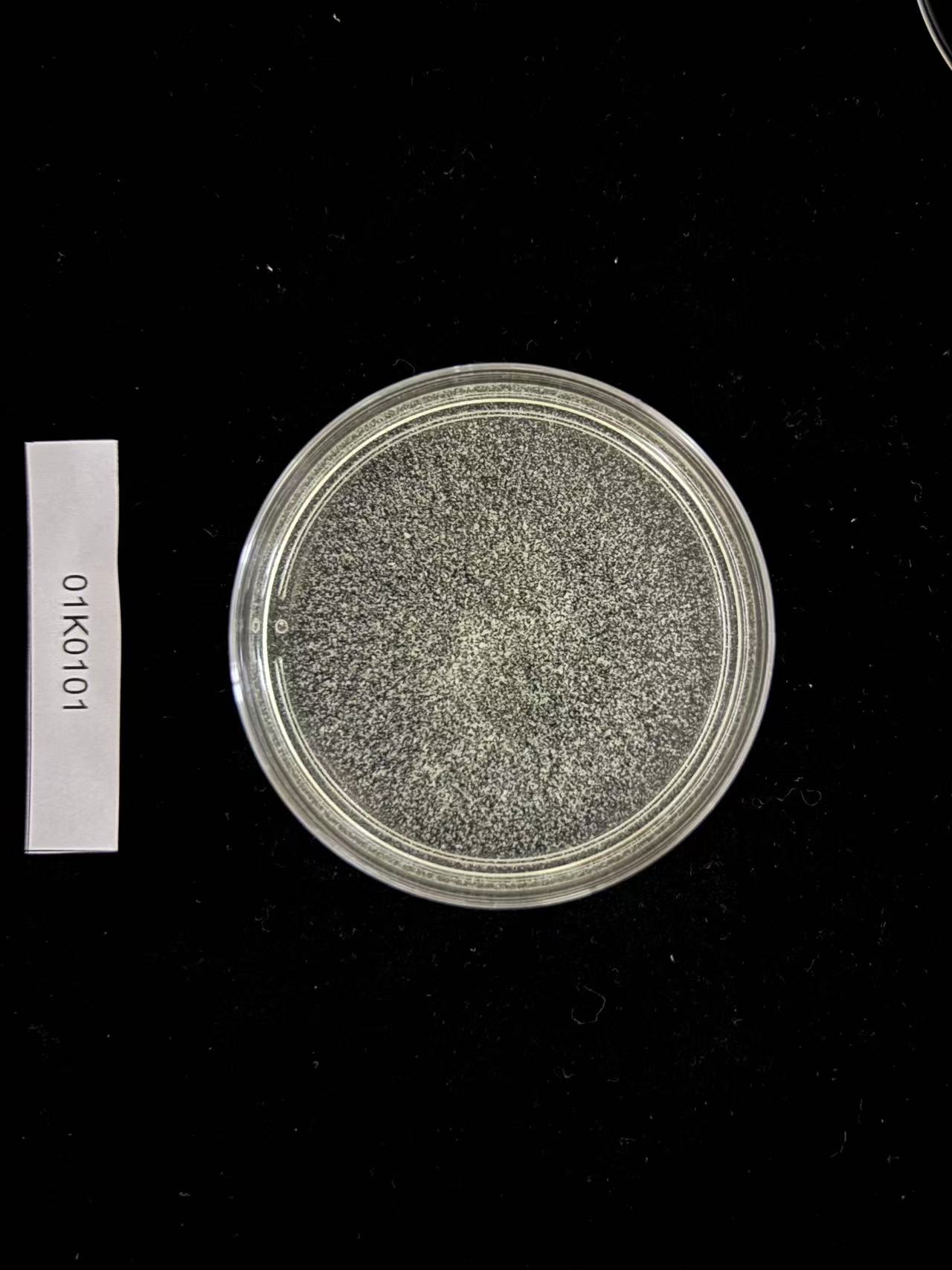

Supplement: Supplementary file 3 — Source data Fig. 1 [file 44319_2026_748_MOESM3_ESM.zip › Figure 1/1F/YE_acetic acid_WT.jpg]

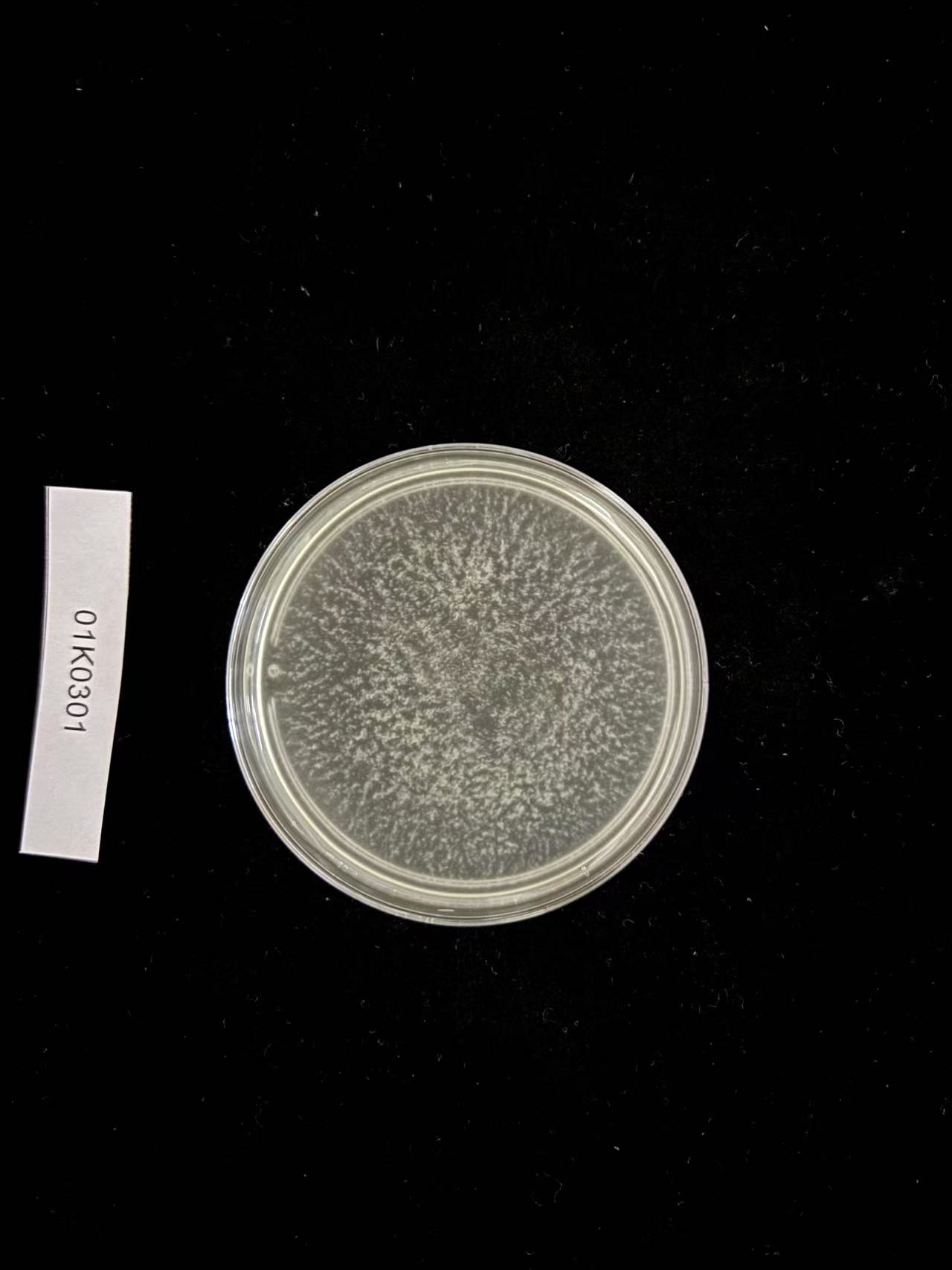

Supplement: Supplementary file 3 — Source data Fig. 1 [file 44319_2026_748_MOESM3_ESM.zip › Figure 1/1F/YE acetic acid_gsf2IE.jpg]

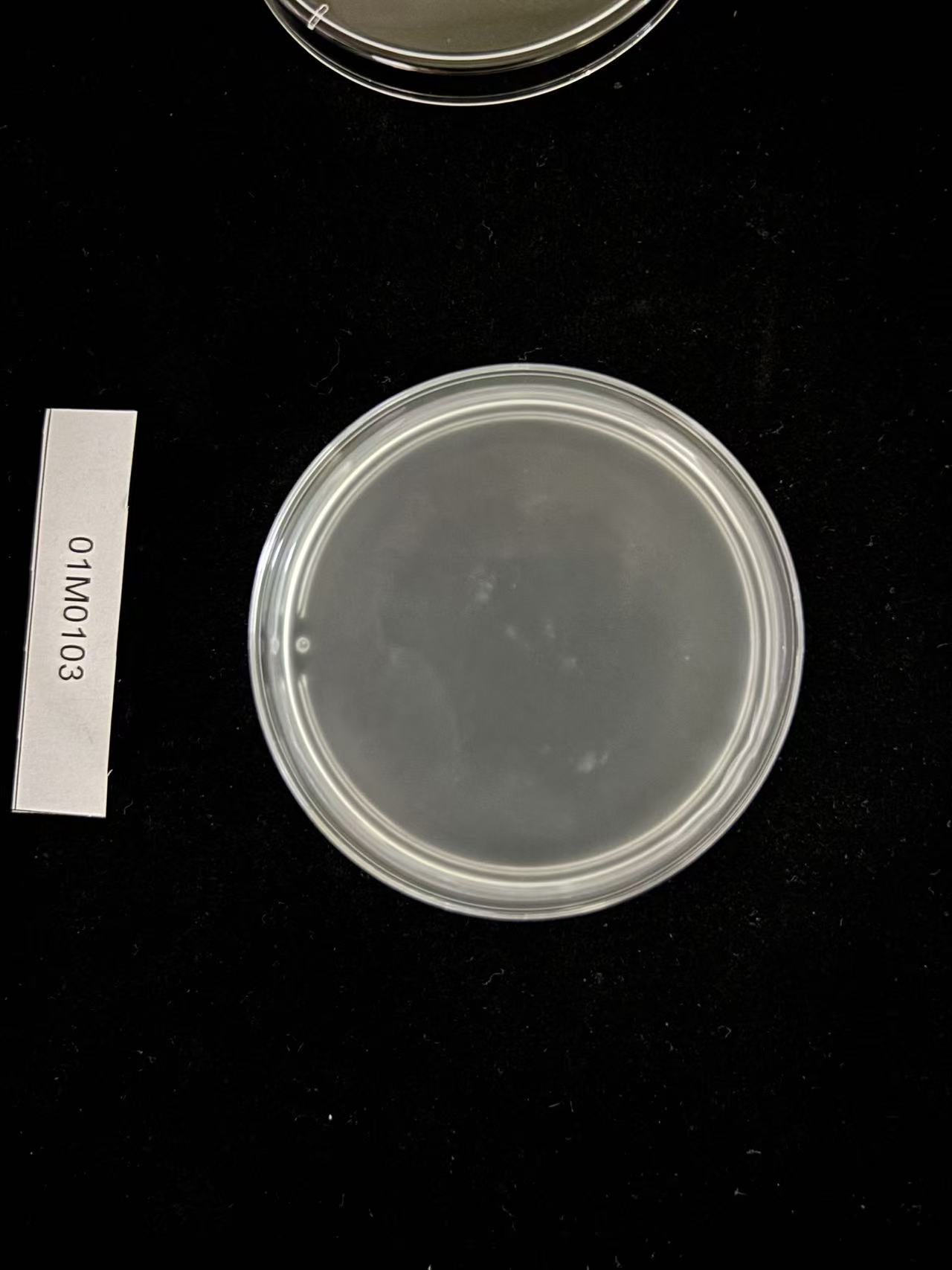

Supplement: Supplementary file 3 — Source data Fig. 1 [file 44319_2026_748_MOESM3_ESM.zip › Figure 1/1F/EMM_WT.jpg]

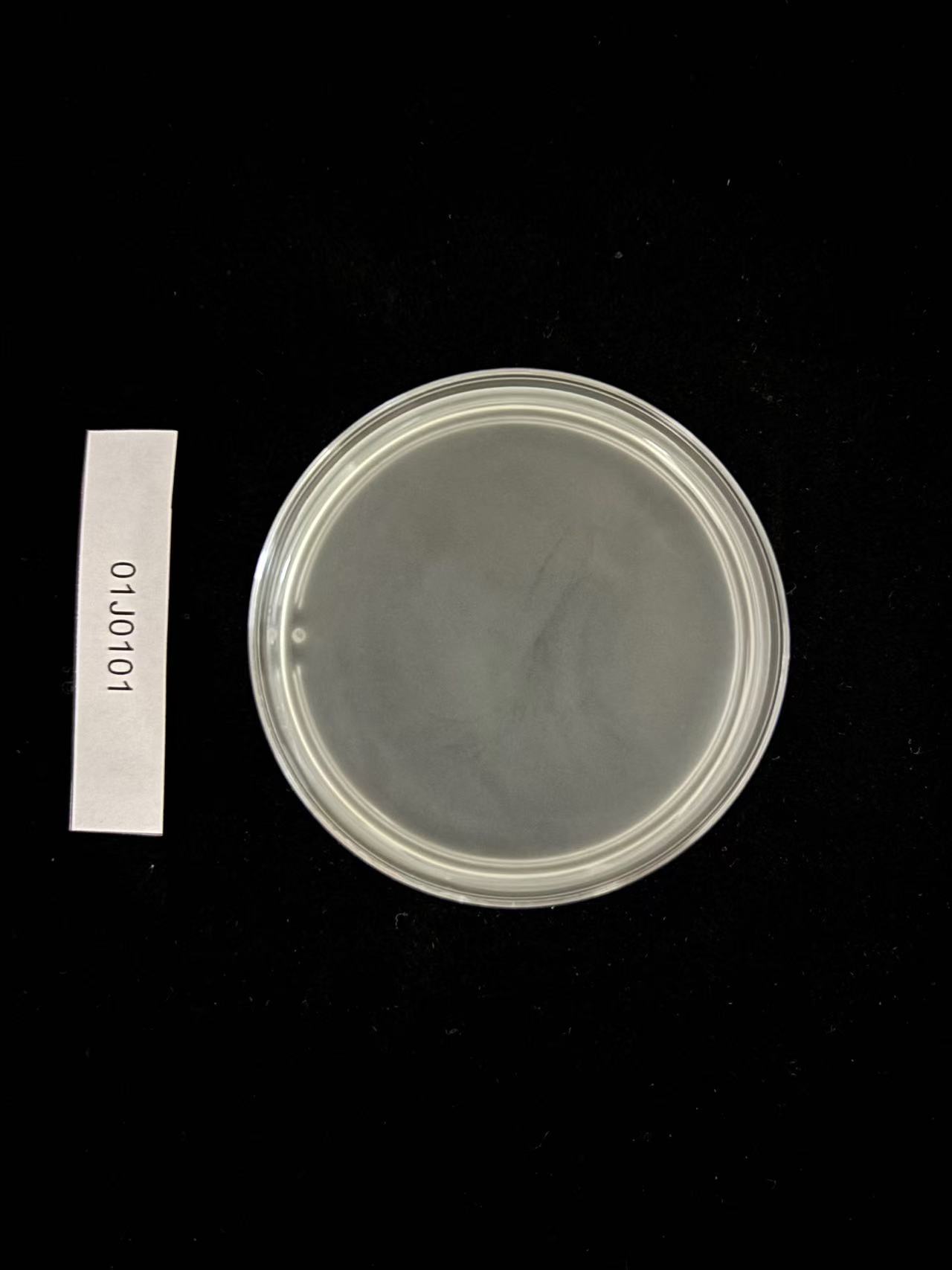

Supplement: Supplementary file 3 — Source data Fig. 1 [file 44319_2026_748_MOESM3_ESM.zip › Figure 1/1F/YE_WT.jpg]

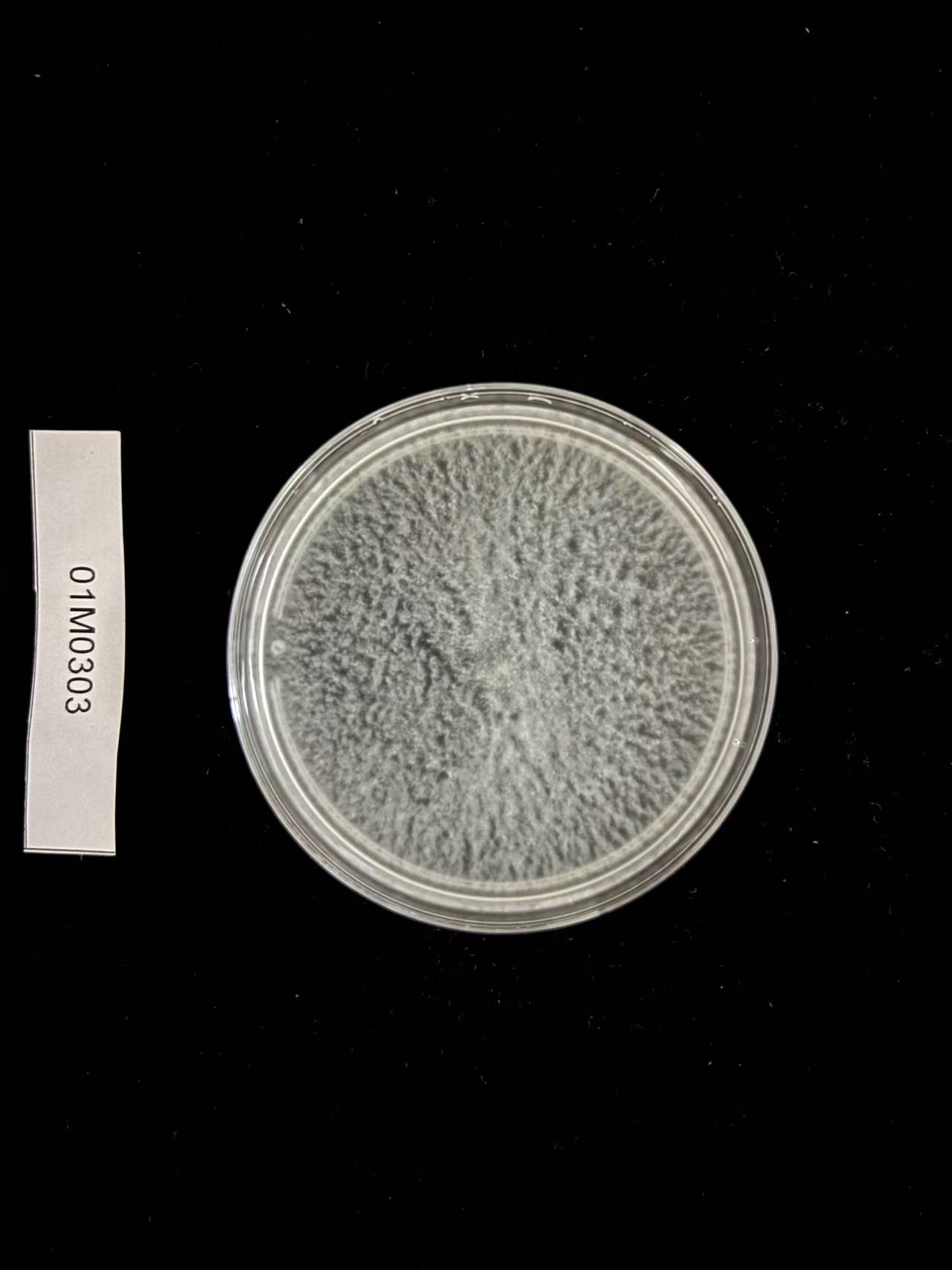

Supplement: Supplementary file 3 — Source data Fig. 1 [file 44319_2026_748_MOESM3_ESM.zip › Figure 1/1F/EMM_gsf2IE.jpg]

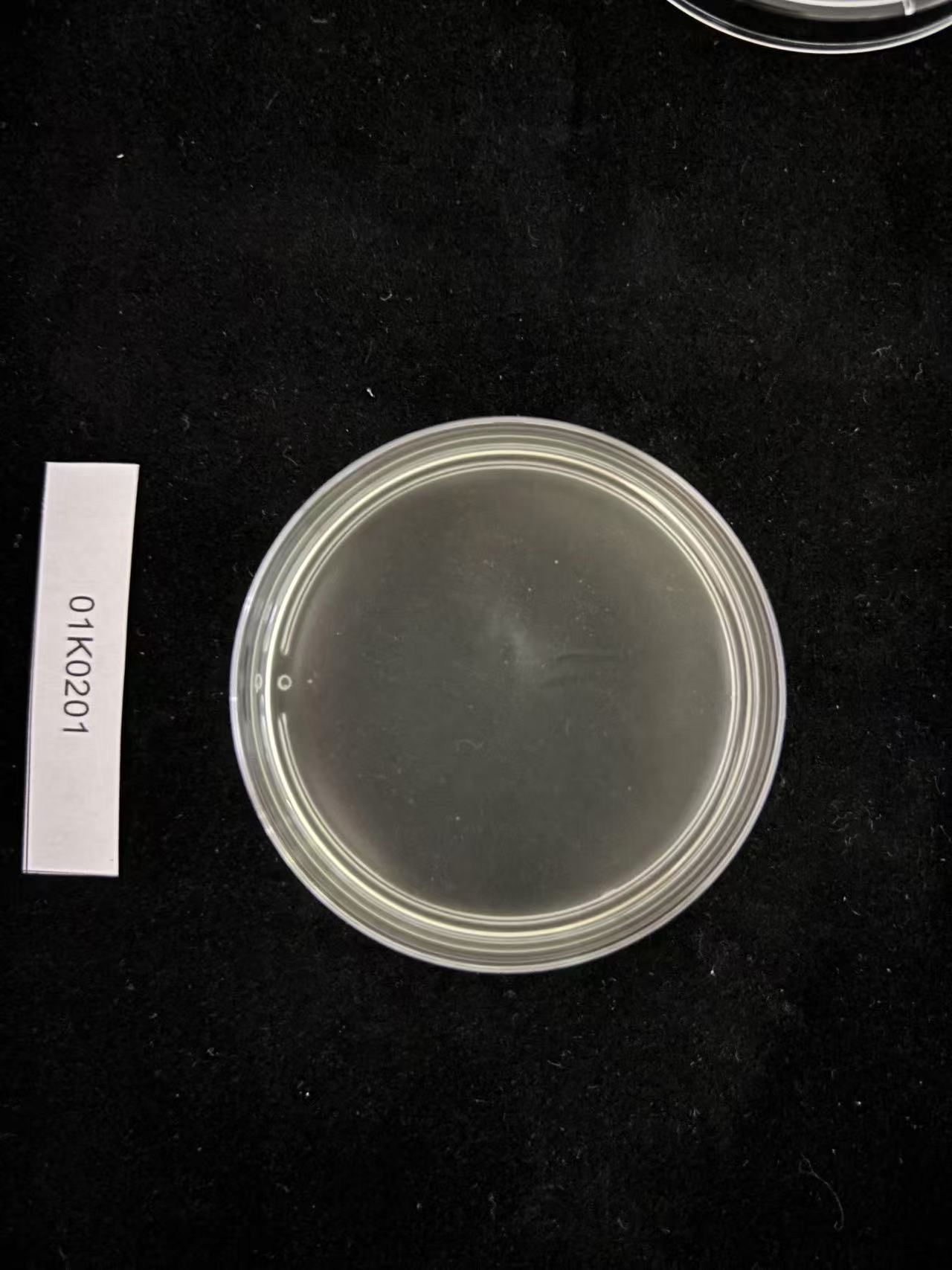

Supplement: Supplementary file 3 — Source data Fig. 1 [file 44319_2026_748_MOESM3_ESM.zip › Figure 1/1F/YE acetic acid_gsf2Γêå.jpg]

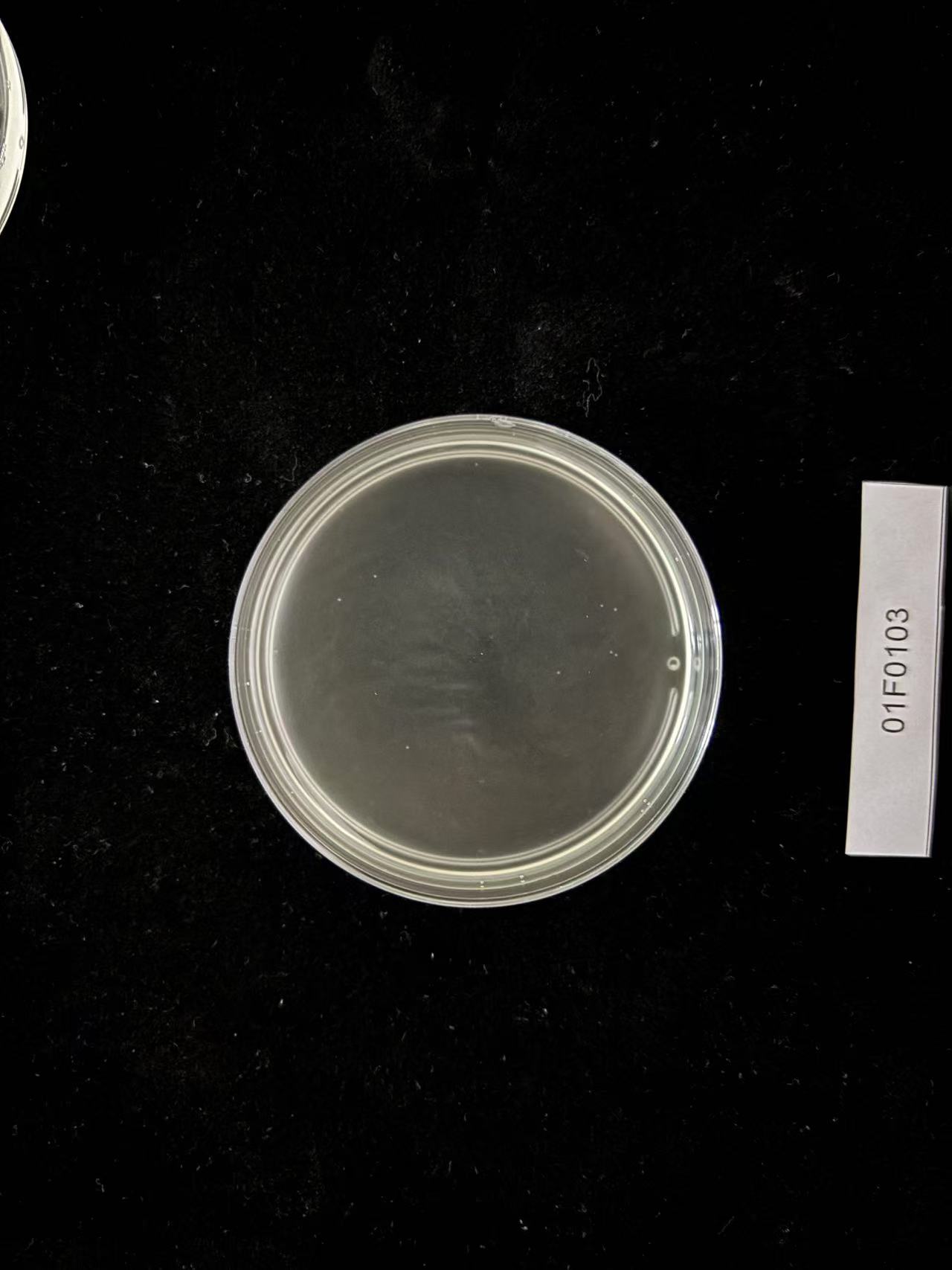

Supplement: Supplementary file 3 — Source data Fig. 1 [file 44319_2026_748_MOESM3_ESM.zip › Figure 1/1B/8 hours_control.jpg]

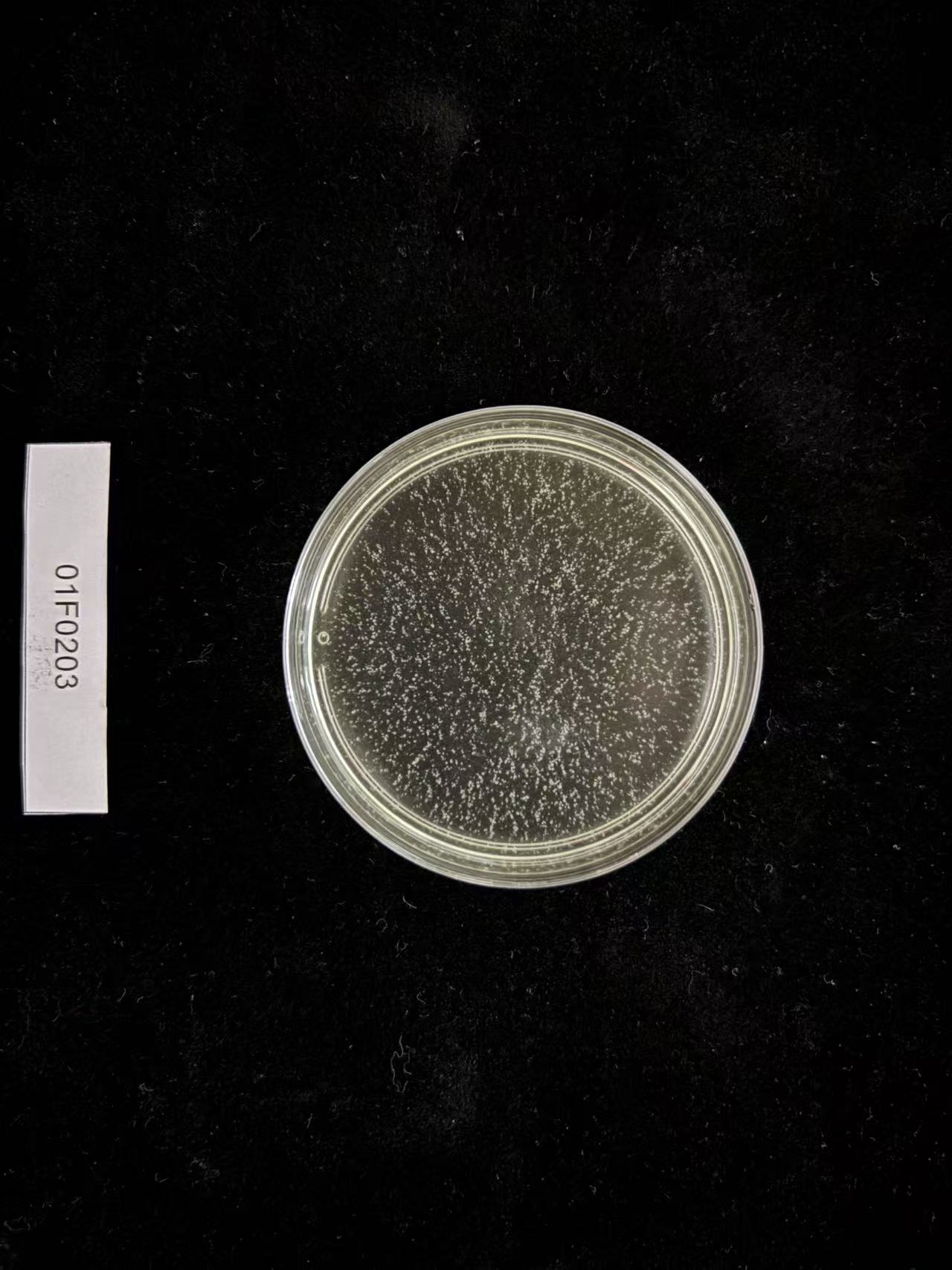

Supplement: Supplementary file 3 — Source data Fig. 1 [file 44319_2026_748_MOESM3_ESM.zip › Figure 1/1B/8 hours_treated.jpg]

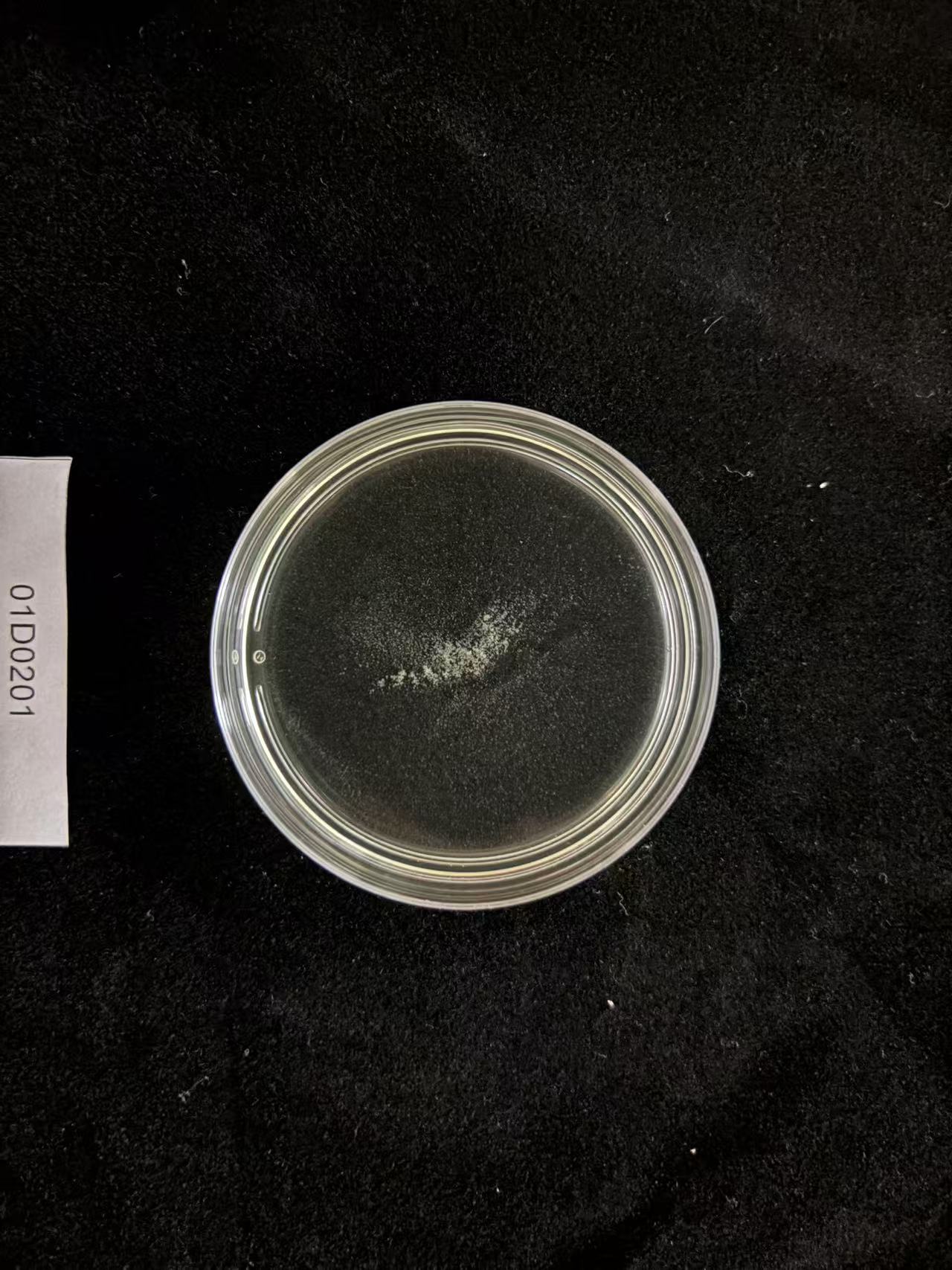

Supplement: Supplementary file 3 — Source data Fig. 1 [file 44319_2026_748_MOESM3_ESM.zip › Figure 1/1B/4 hours_treated.jpg]

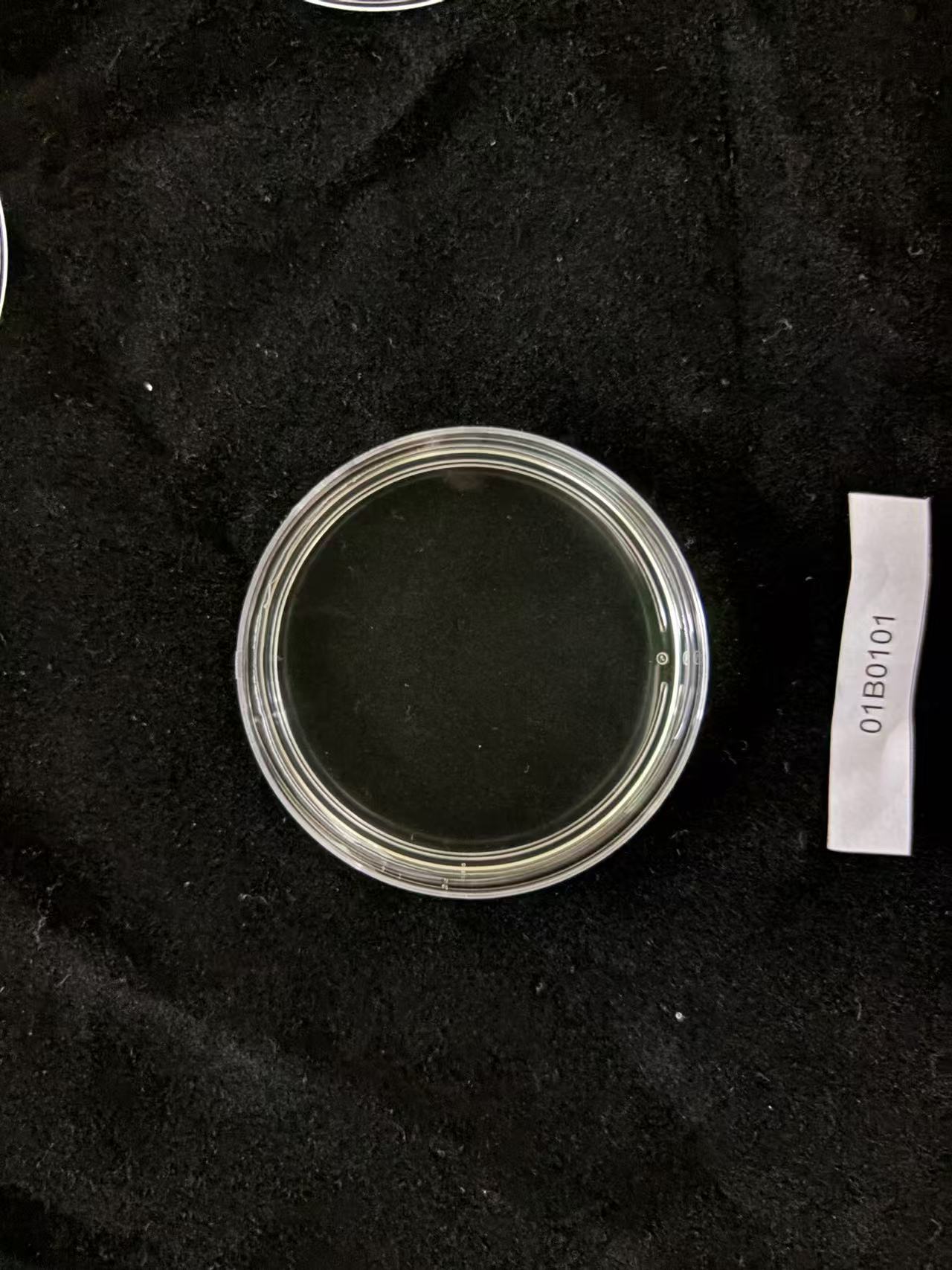

Supplement: Supplementary file 3 — Source data Fig. 1 [file 44319_2026_748_MOESM3_ESM.zip › Figure 1/1B/2 hours_control.jpg]

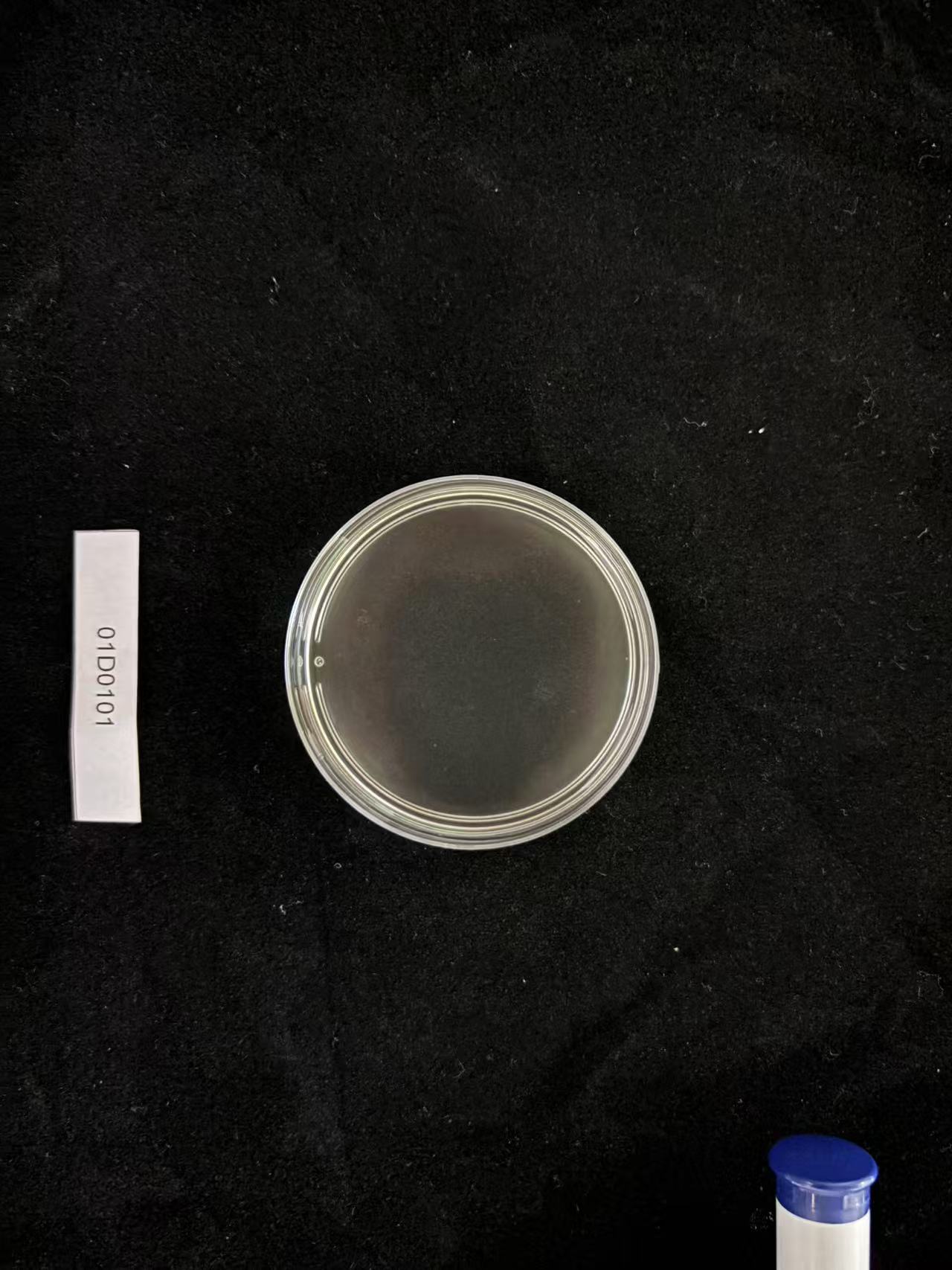

Supplement: Supplementary file 3 — Source data Fig. 1 [file 44319_2026_748_MOESM3_ESM.zip › Figure 1/1B/4 hours_control.jpg]

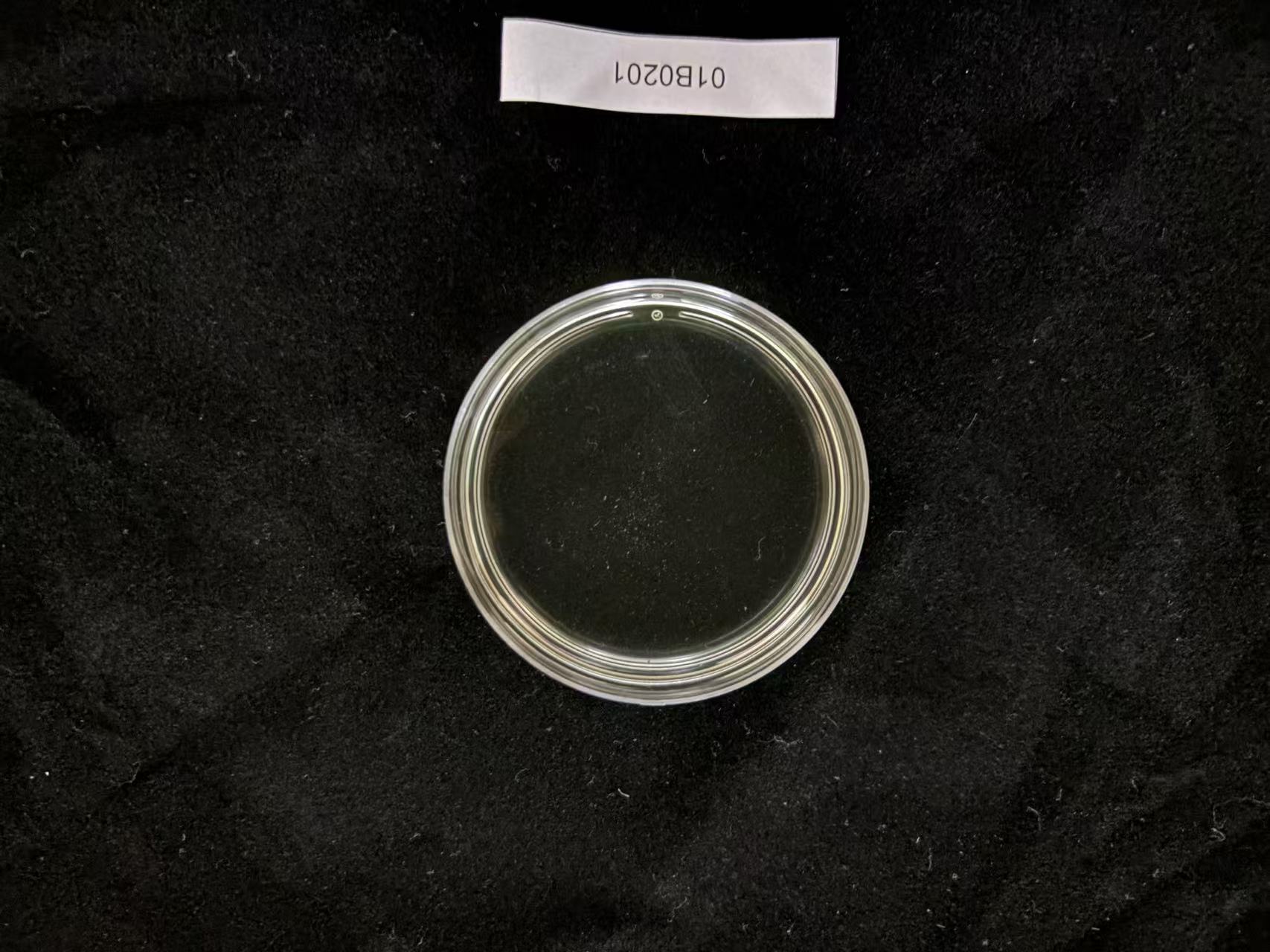

Supplement: Supplementary file 3 — Source data Fig. 1 [file 44319_2026_748_MOESM3_ESM.zip › Figure 1/1B/2 hours_treated.jpg]

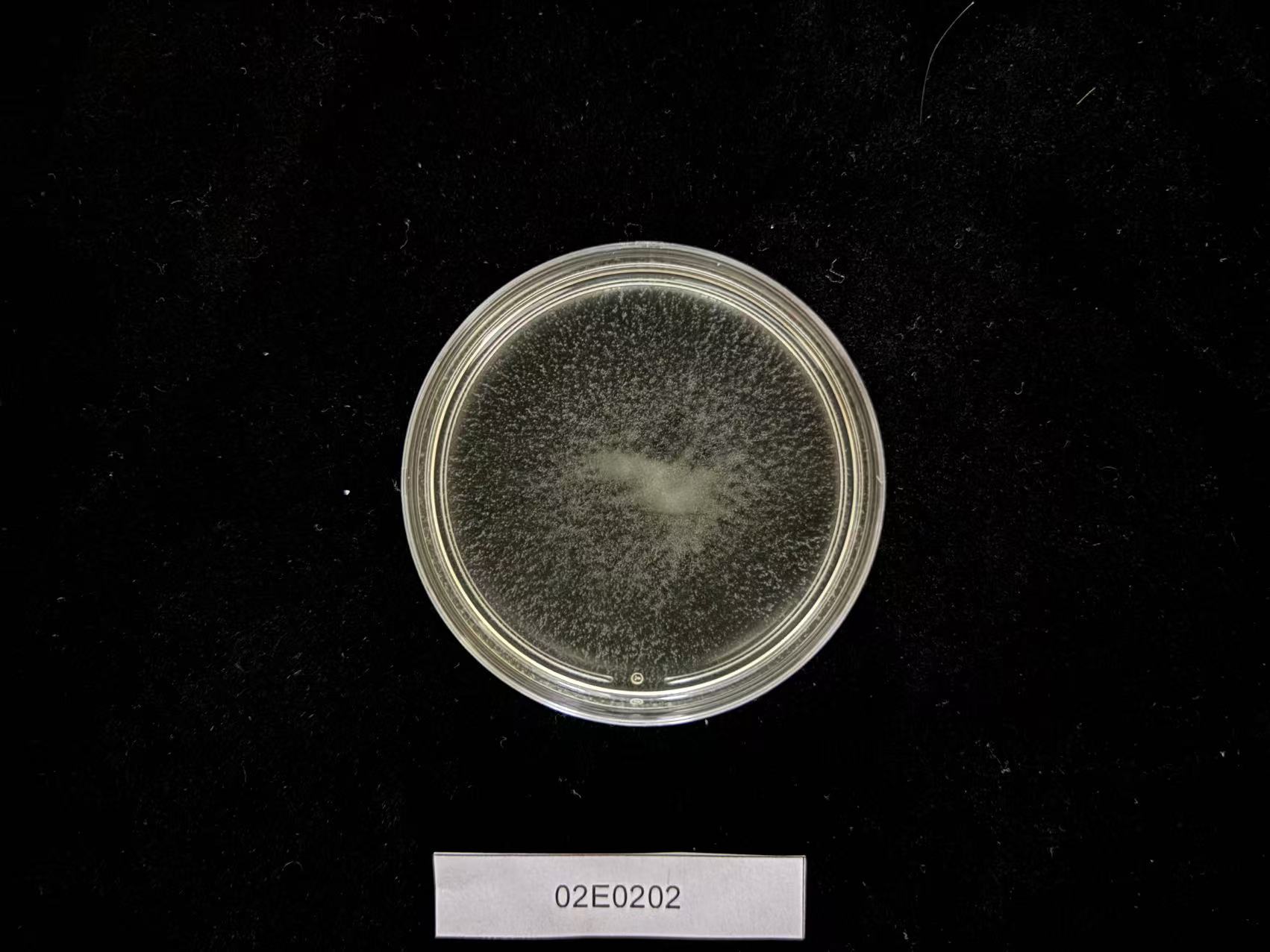

Supplement: Supplementary file 4 — Source data Fig. 2 [file 44319_2026_748_MOESM4_ESM.zip › Figure 2/2B/oxalic acid.jpg]

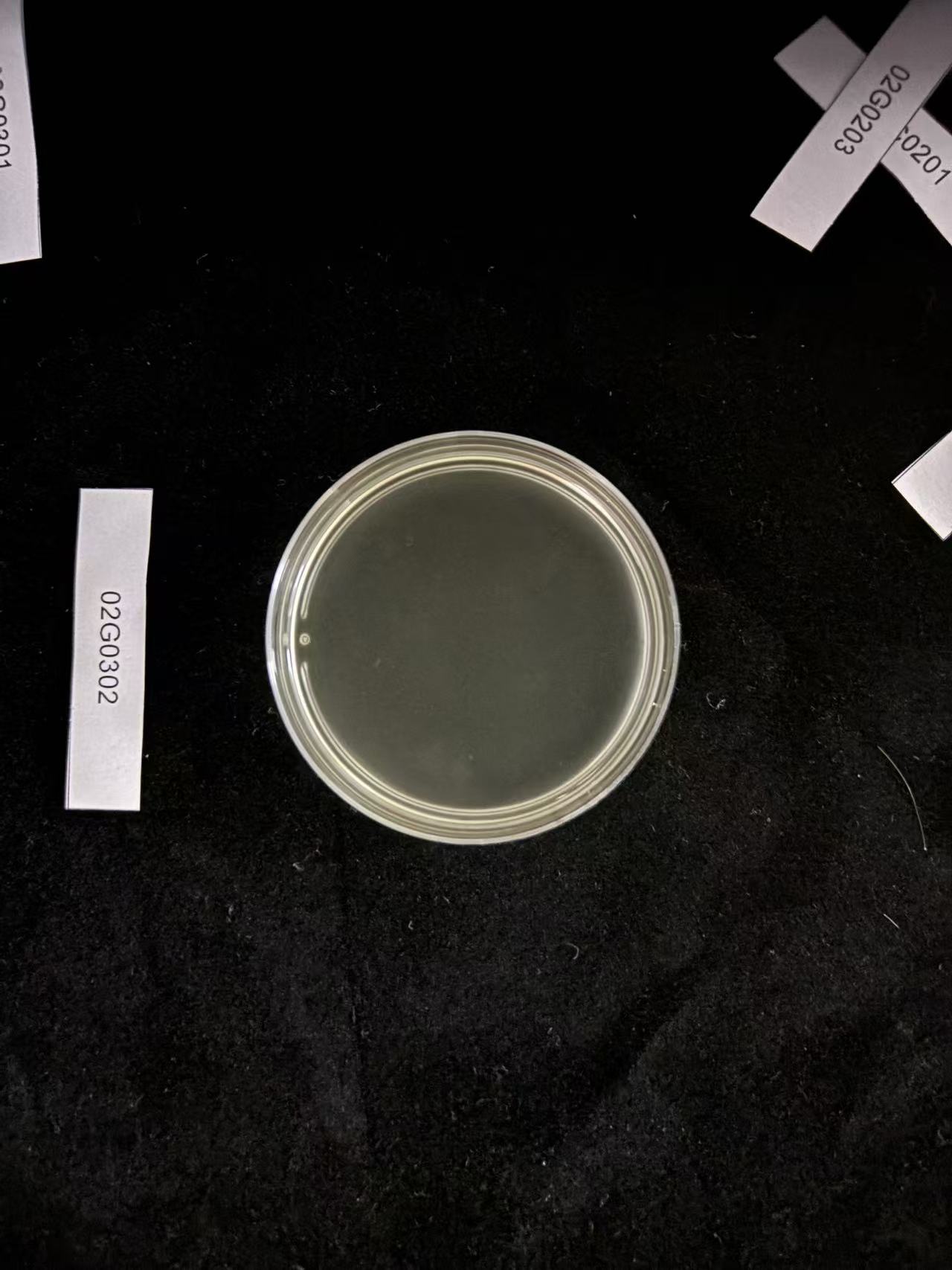

Supplement: Supplementary file 4 — Source data Fig. 2 [file 44319_2026_748_MOESM4_ESM.zip › Figure 2/2B/nitric acid galactose.jpg]

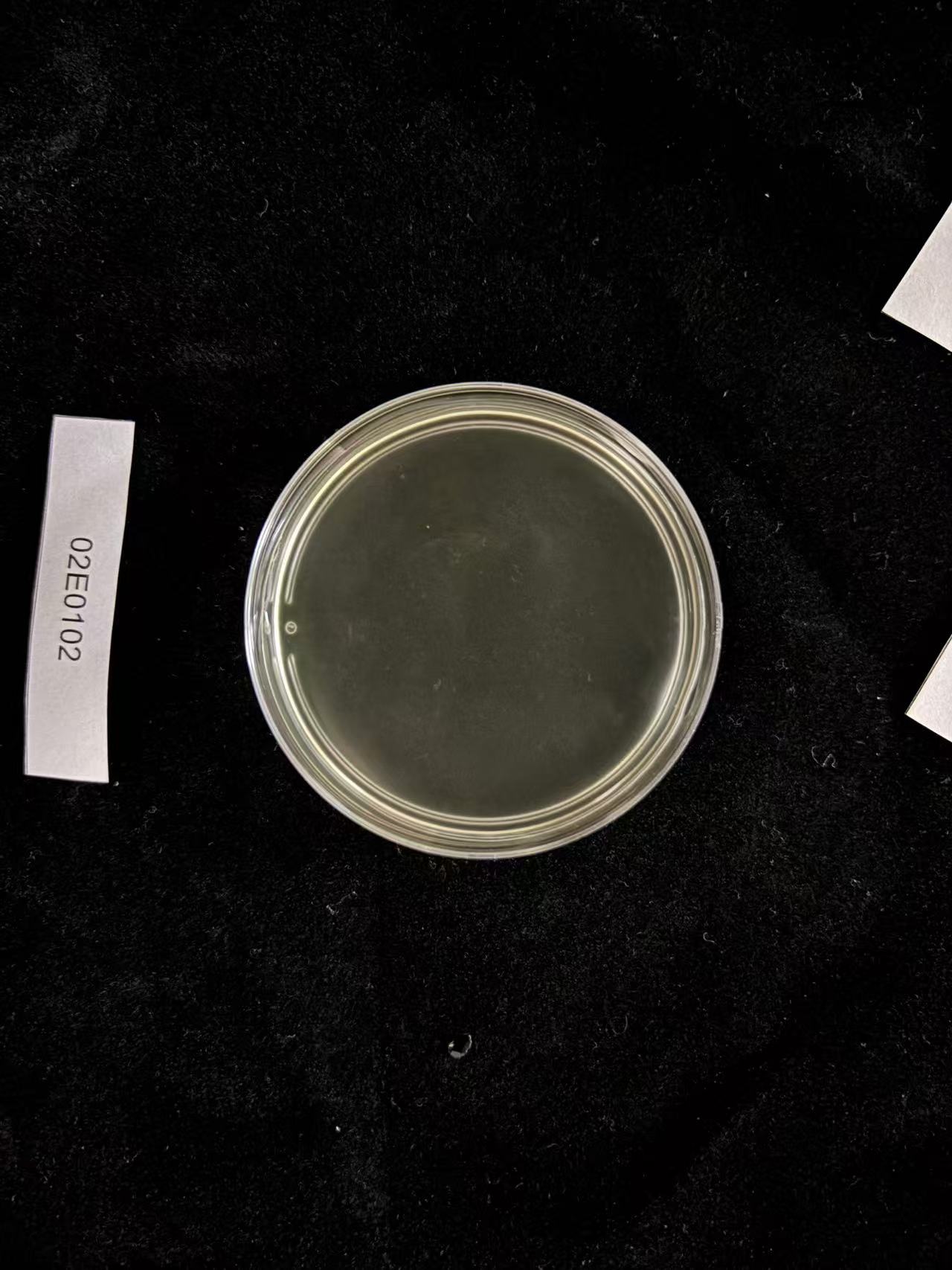

Supplement: Supplementary file 4 — Source data Fig. 2 [file 44319_2026_748_MOESM4_ESM.zip › Figure 2/2B/control(oxalic acid).jpg]

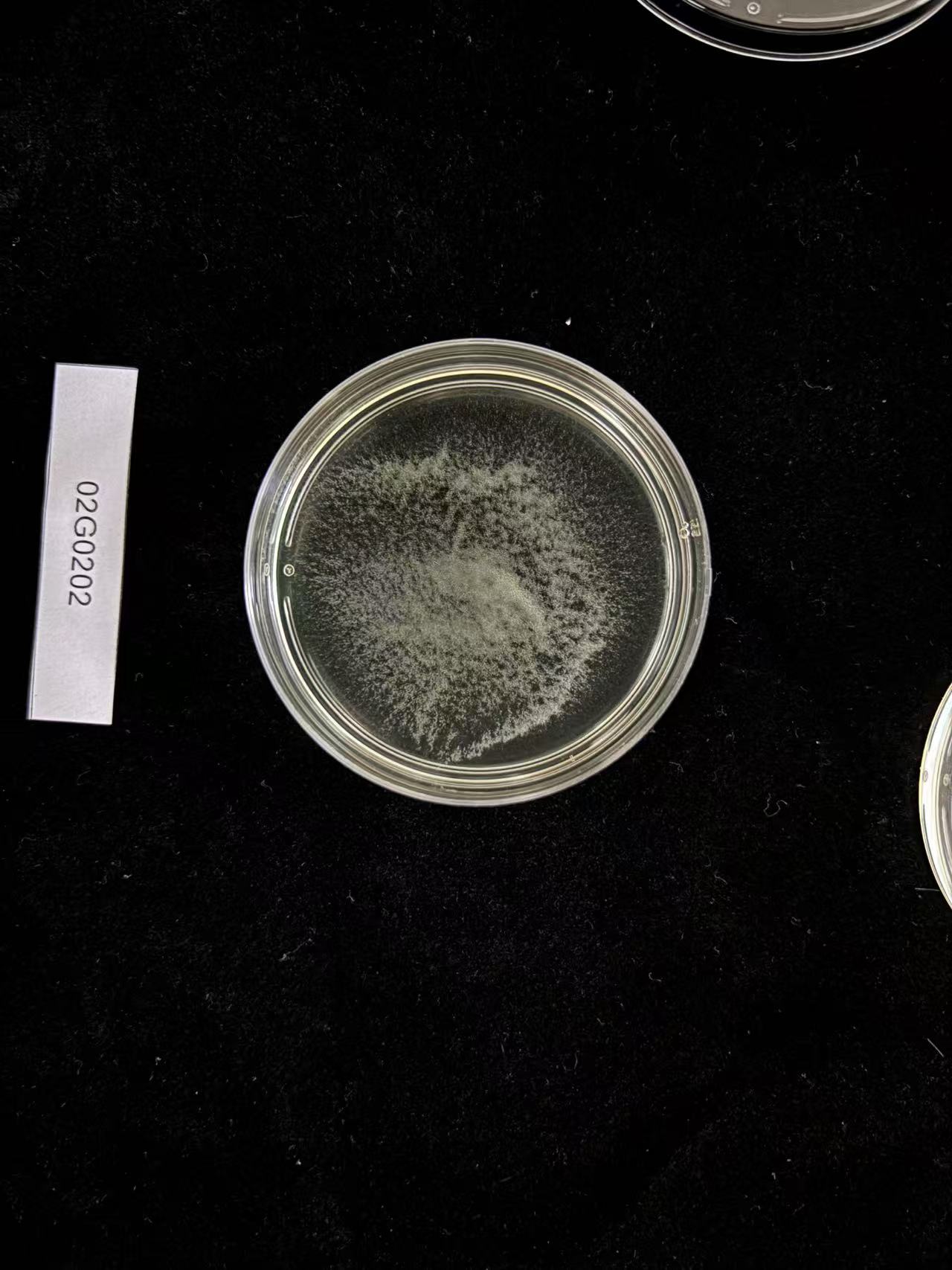

Supplement: Supplementary file 4 — Source data Fig. 2 [file 44319_2026_748_MOESM4_ESM.zip › Figure 2/2B/nitric acid.jpg]

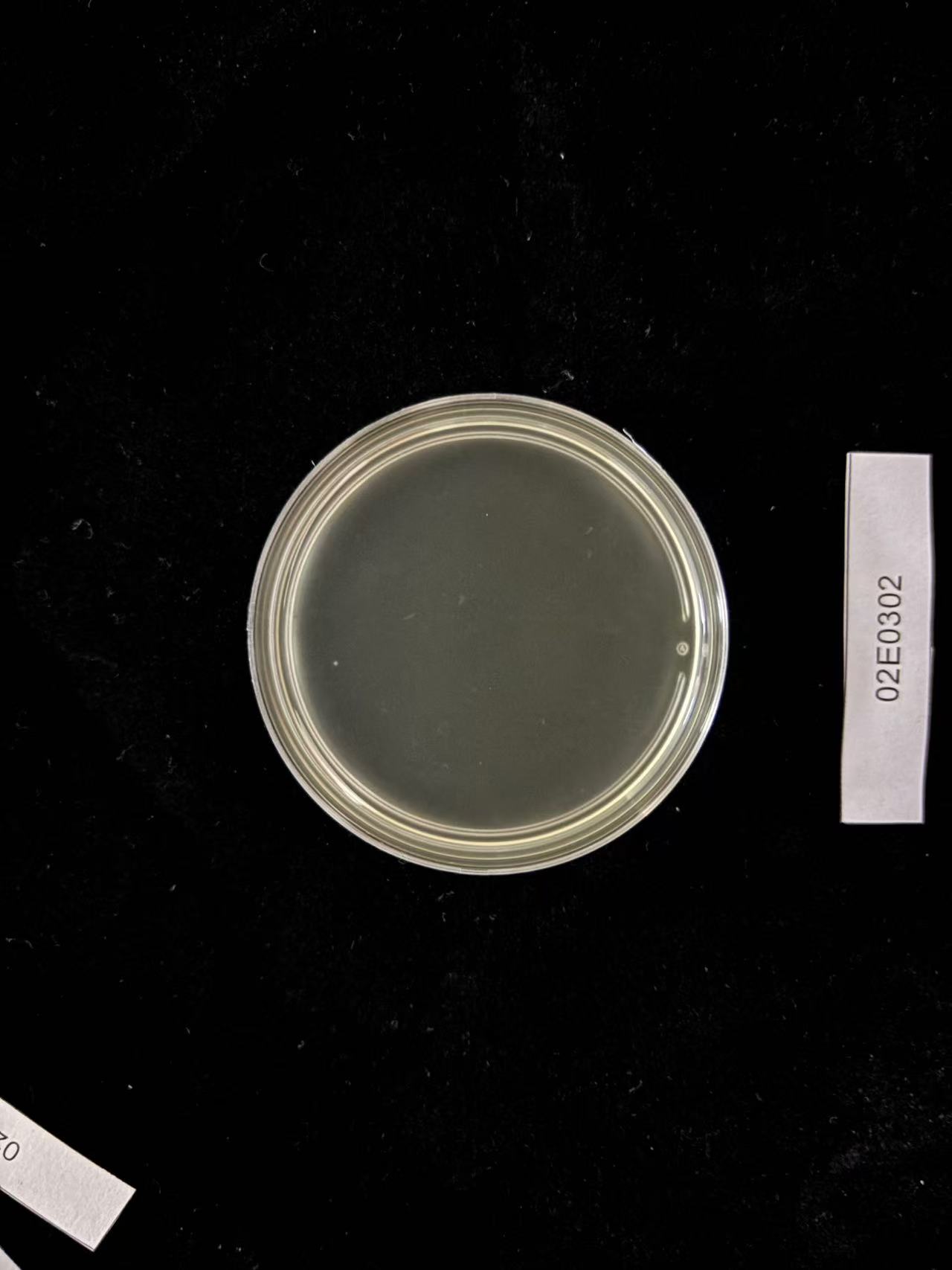

Supplement: Supplementary file 4 — Source data Fig. 2 [file 44319_2026_748_MOESM4_ESM.zip › Figure 2/2B/oxalic acid galactose.jpg]

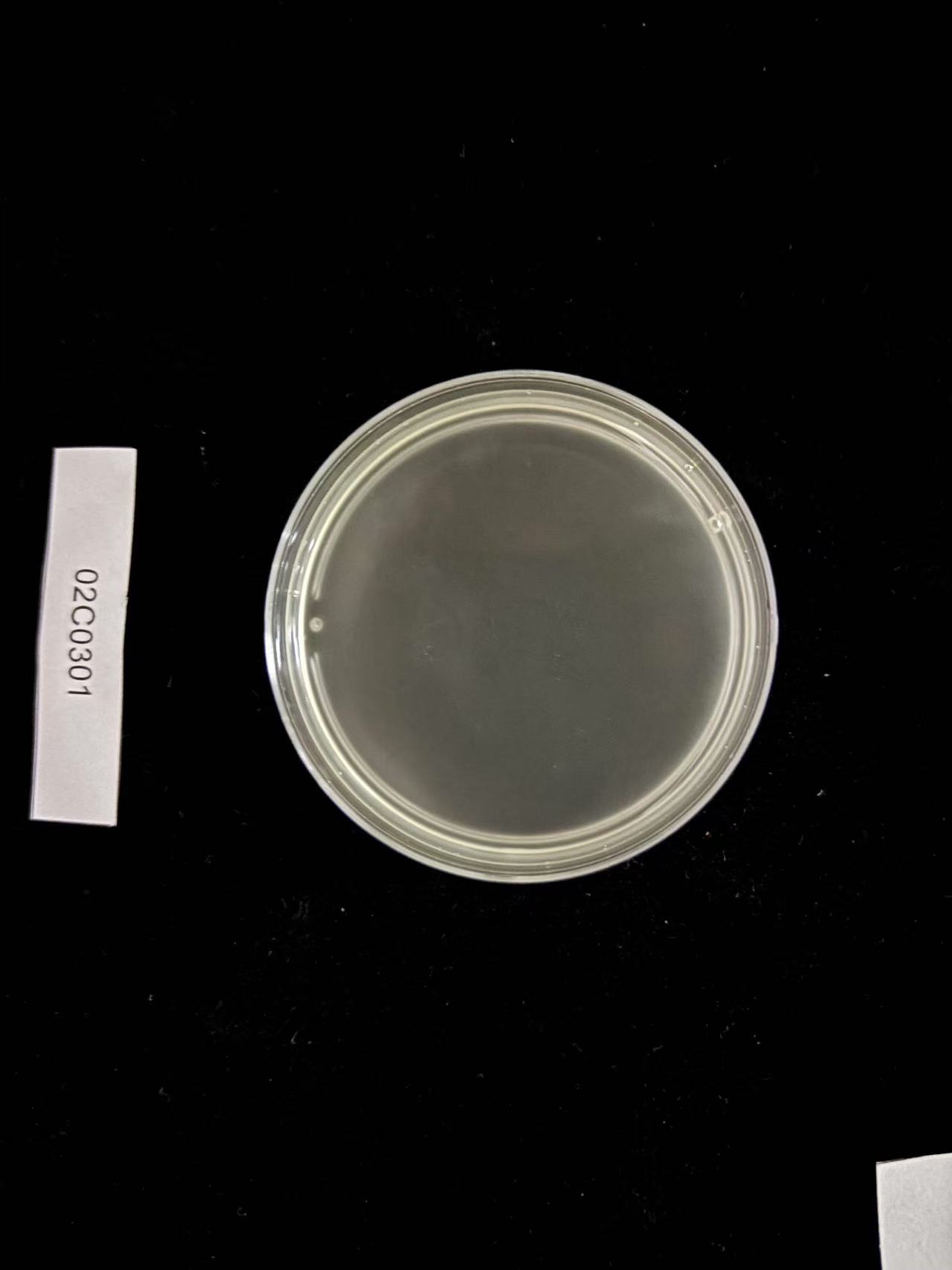

Supplement: Supplementary file 4 — Source data Fig. 2 [file 44319_2026_748_MOESM4_ESM.zip › Figure 2/2B/hydrochloric acid galactose.jpg]

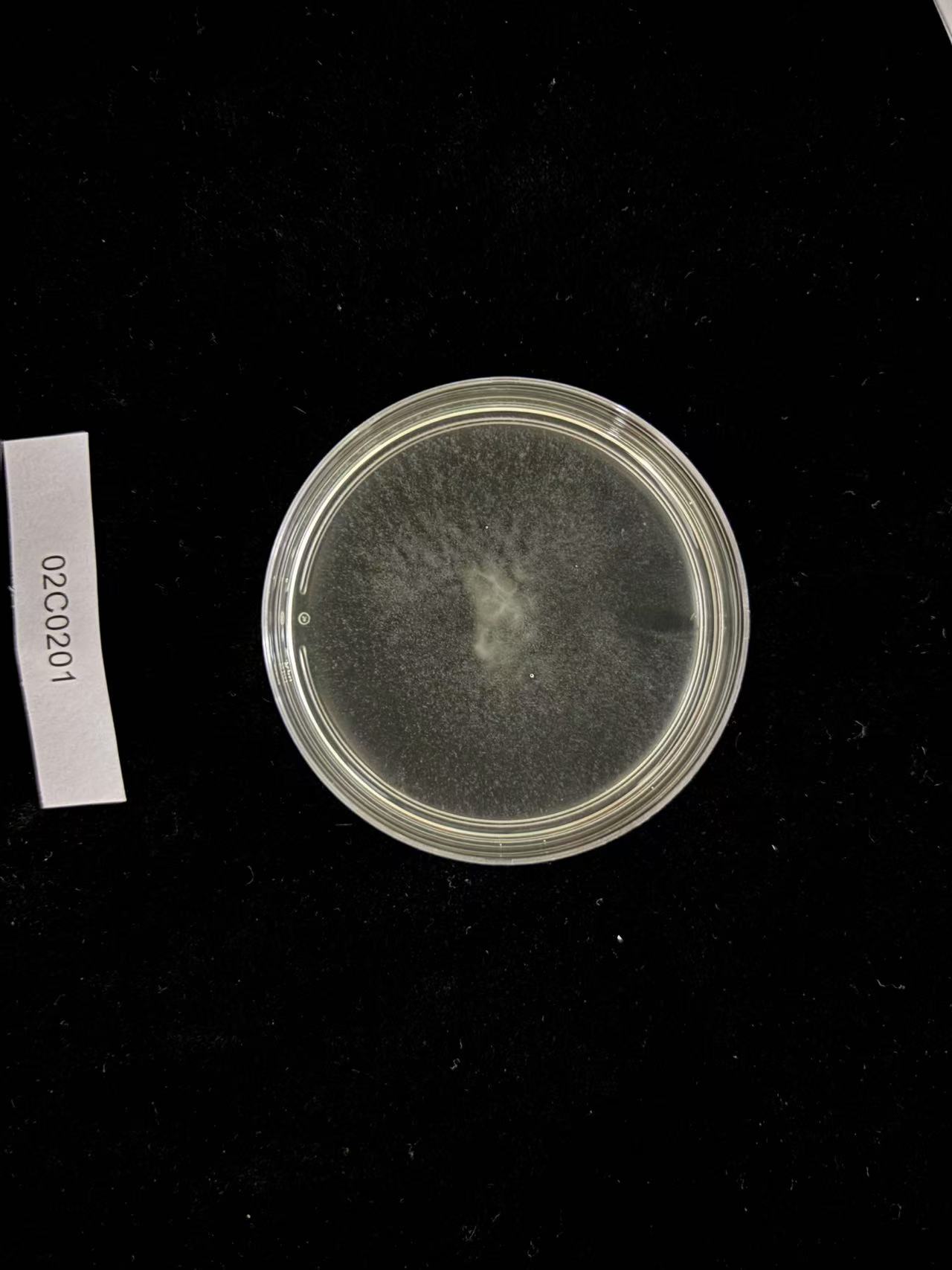

Supplement: Supplementary file 4 — Source data Fig. 2 [file 44319_2026_748_MOESM4_ESM.zip › Figure 2/2B/hydrochloric acid.jpg]

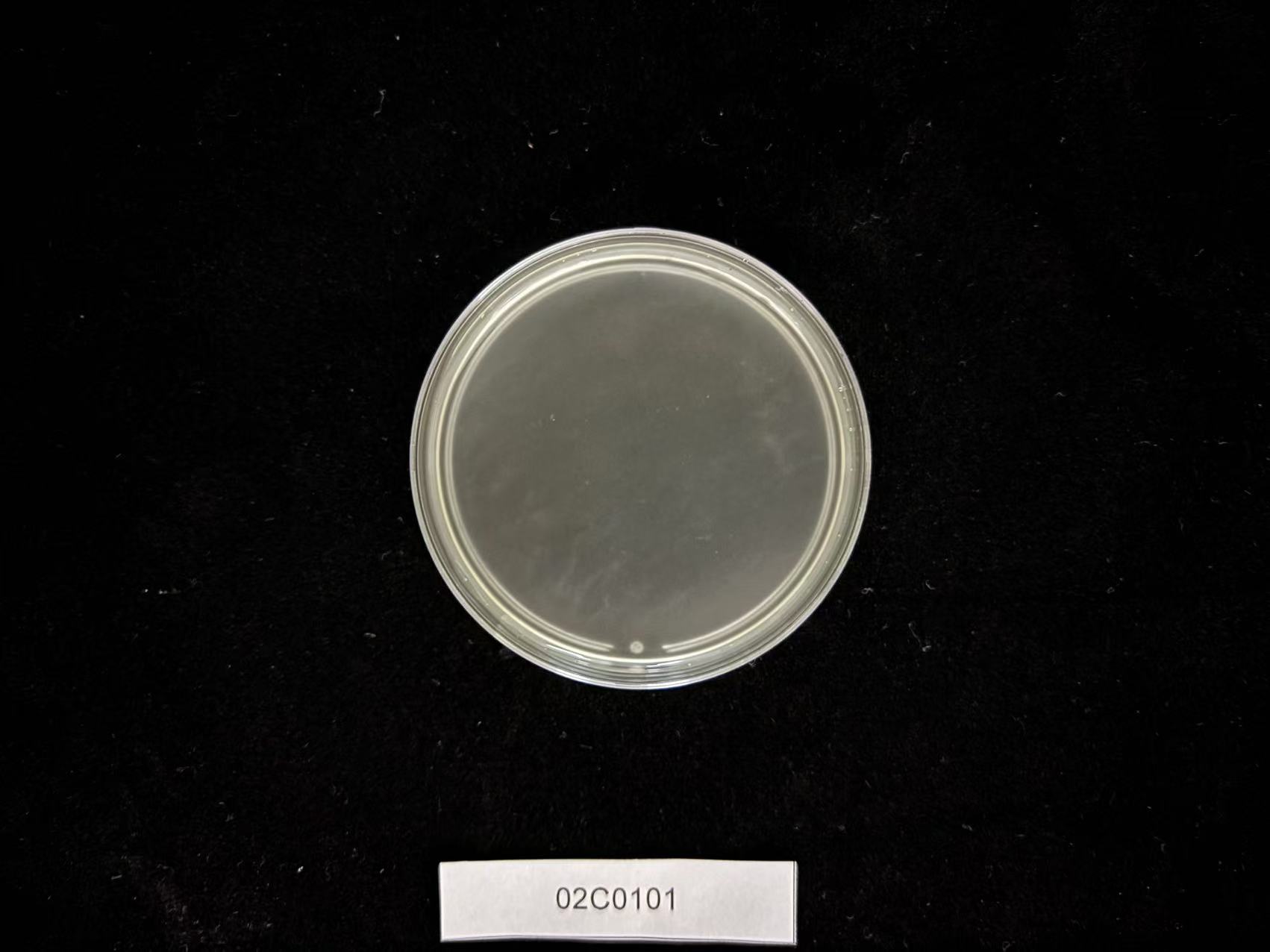

Supplement: Supplementary file 4 — Source data Fig. 2 [file 44319_2026_748_MOESM4_ESM.zip › Figure 2/2B/contral(hydrochloric acid).jpg]

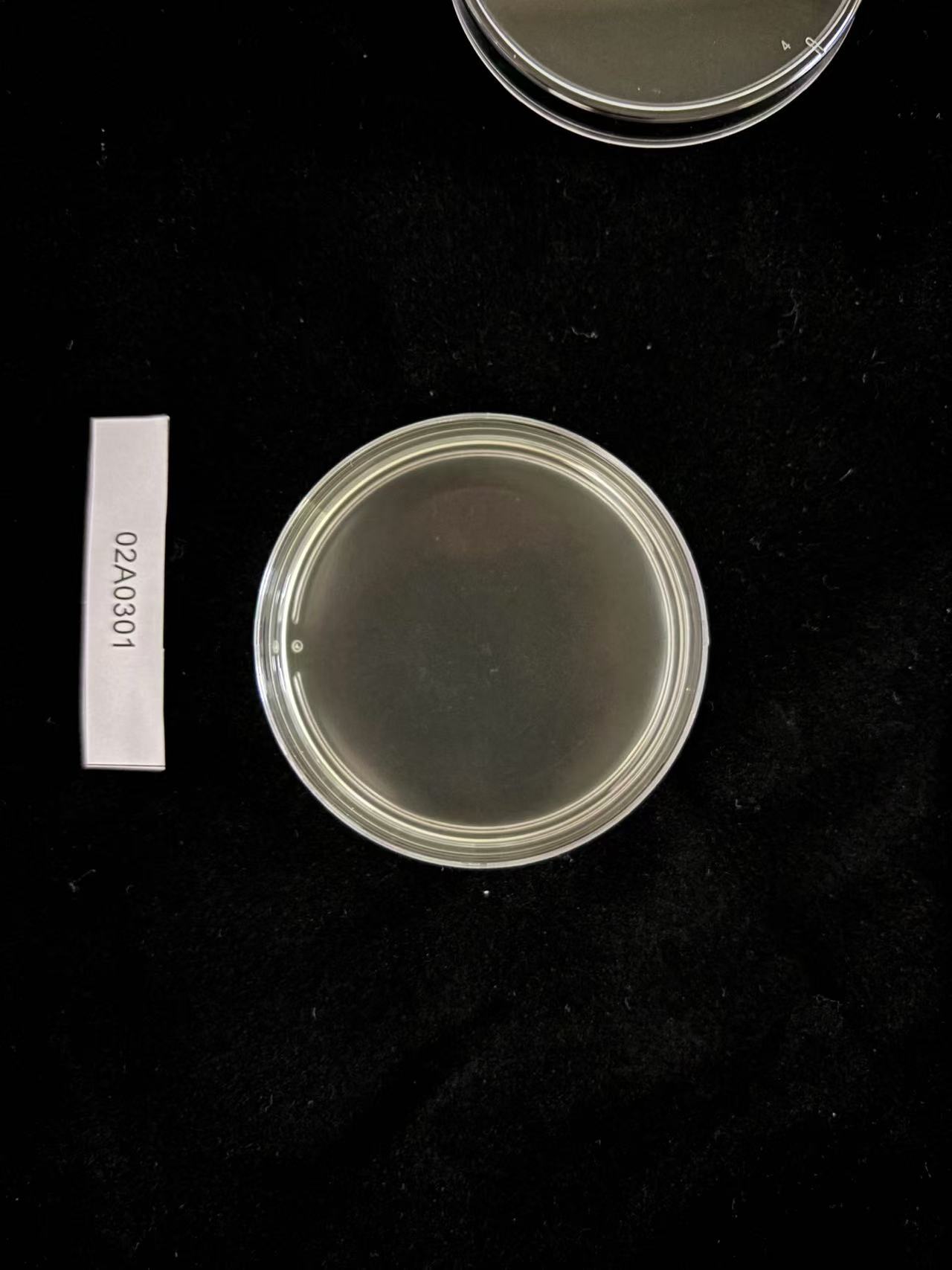

Supplement: Supplementary file 4 — Source data Fig. 2 [file 44319_2026_748_MOESM4_ESM.zip › Figure 2/2B/acetic acid_galactose.jpg]

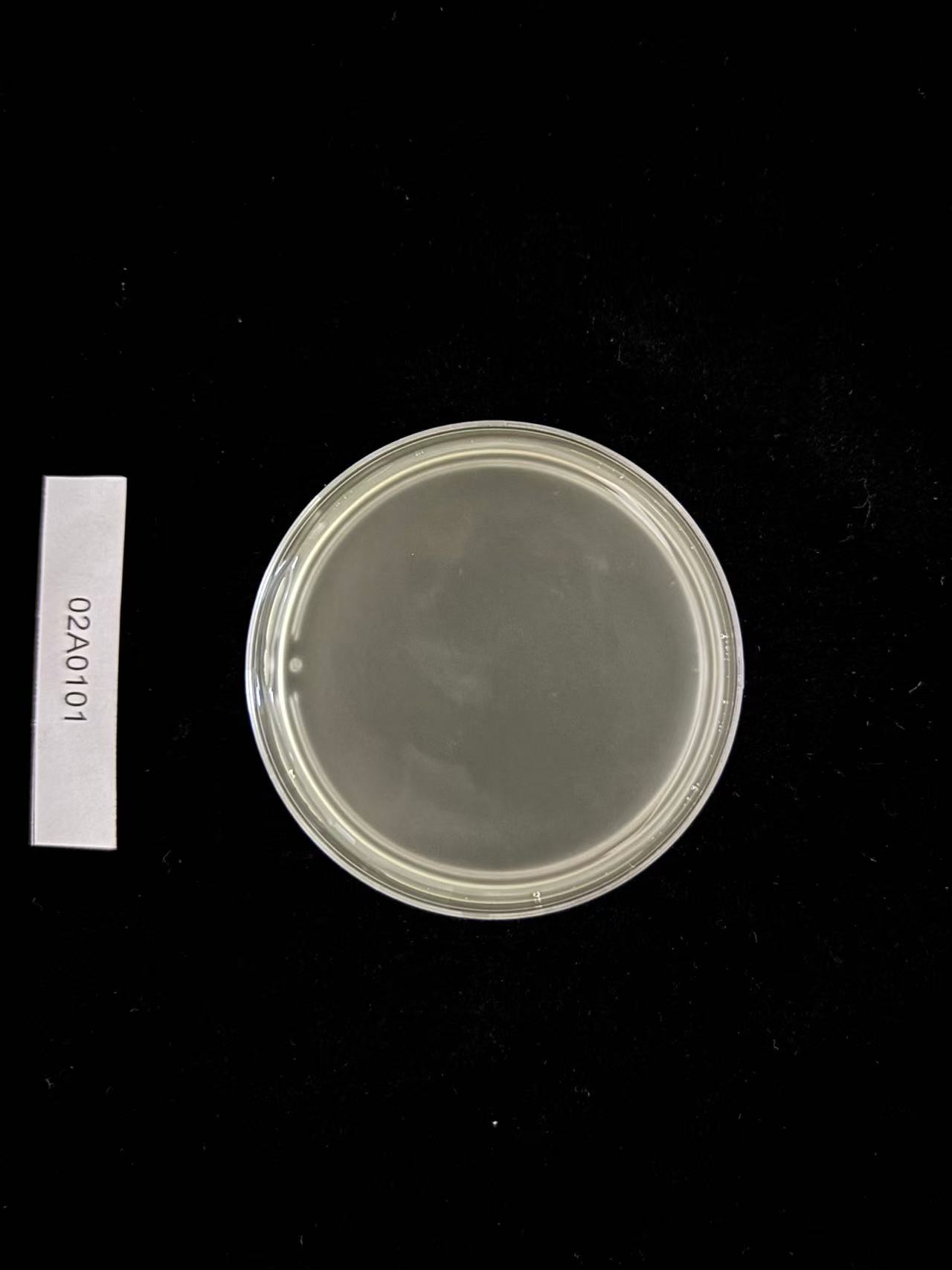

Supplement: Supplementary file 4 — Source data Fig. 2 [file 44319_2026_748_MOESM4_ESM.zip › Figure 2/2B/contral(acetic acid).jpg]

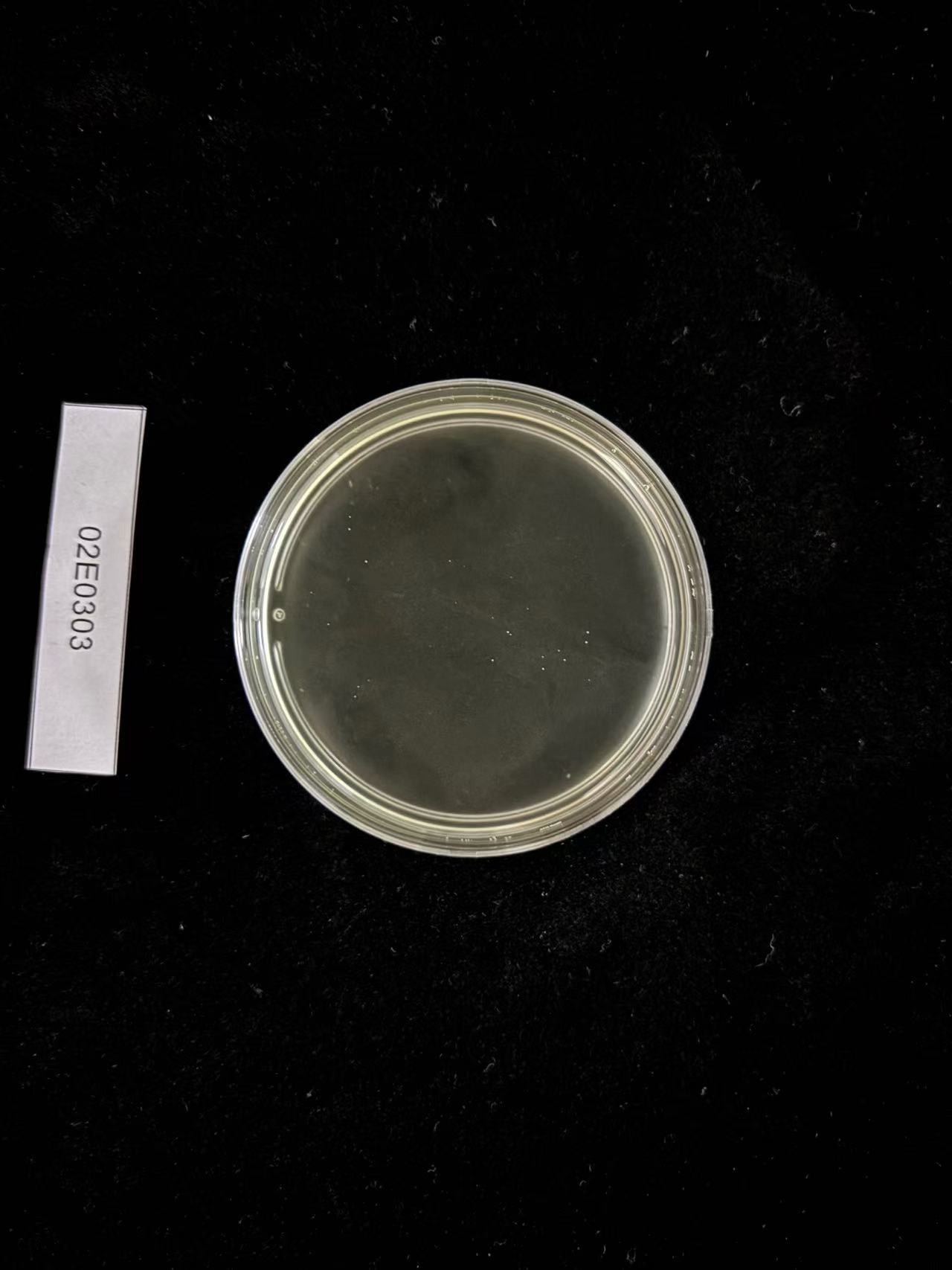

Supplement: Supplementary file 4 — Source data Fig. 2 [file 44319_2026_748_MOESM4_ESM.zip › Figure 2/2B/control(nitric acid).jpg]

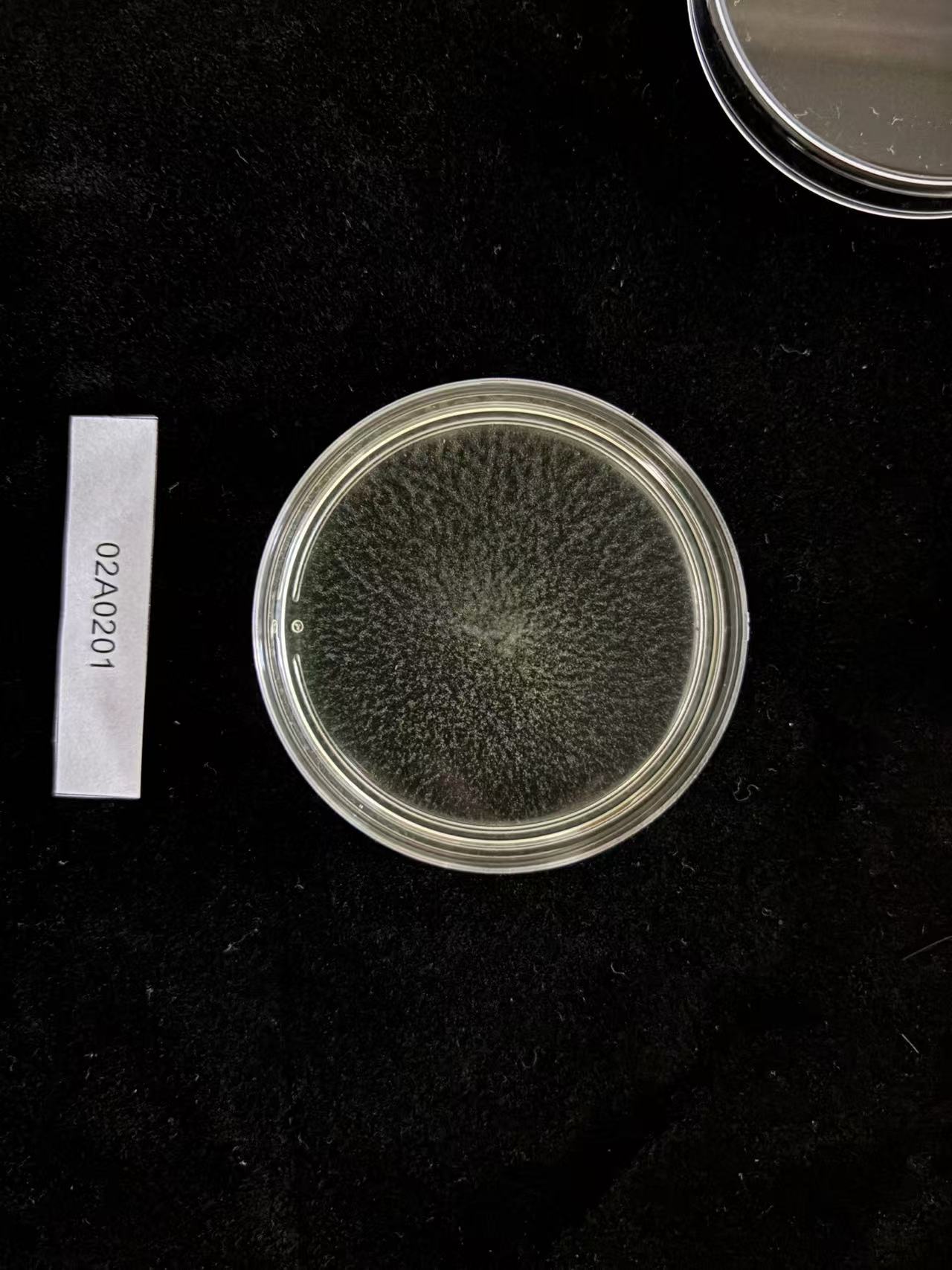

Supplement: Supplementary file 4 — Source data Fig. 2 [file 44319_2026_748_MOESM4_ESM.zip › Figure 2/2B/acetic acid.jpg]

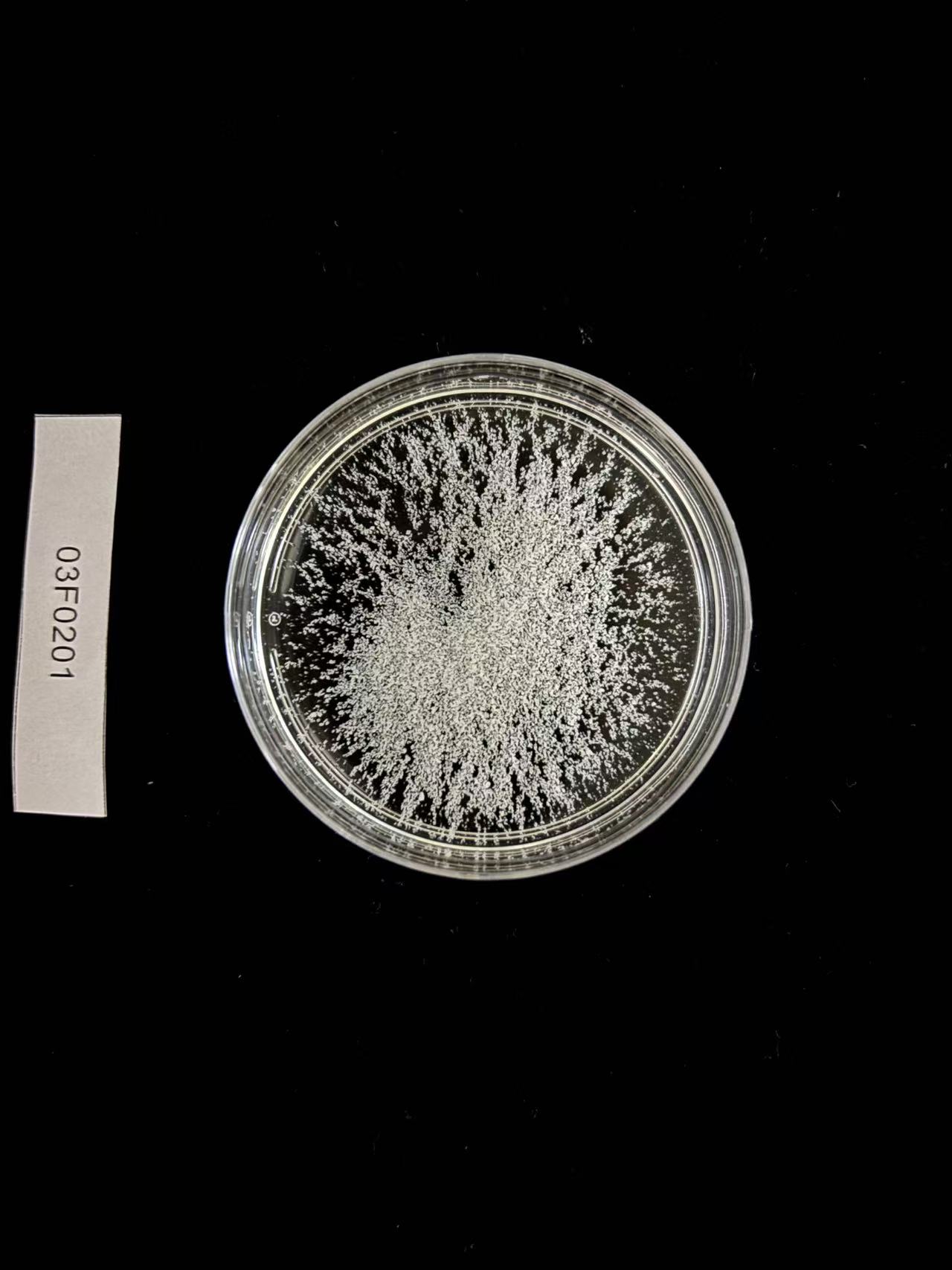

Supplement: Supplementary file 5 — Source data Fig. 3 [file 44319_2026_748_MOESM5_ESM.zip › Figure 3/3K/gsf2IE_Repeat 1.jpg]

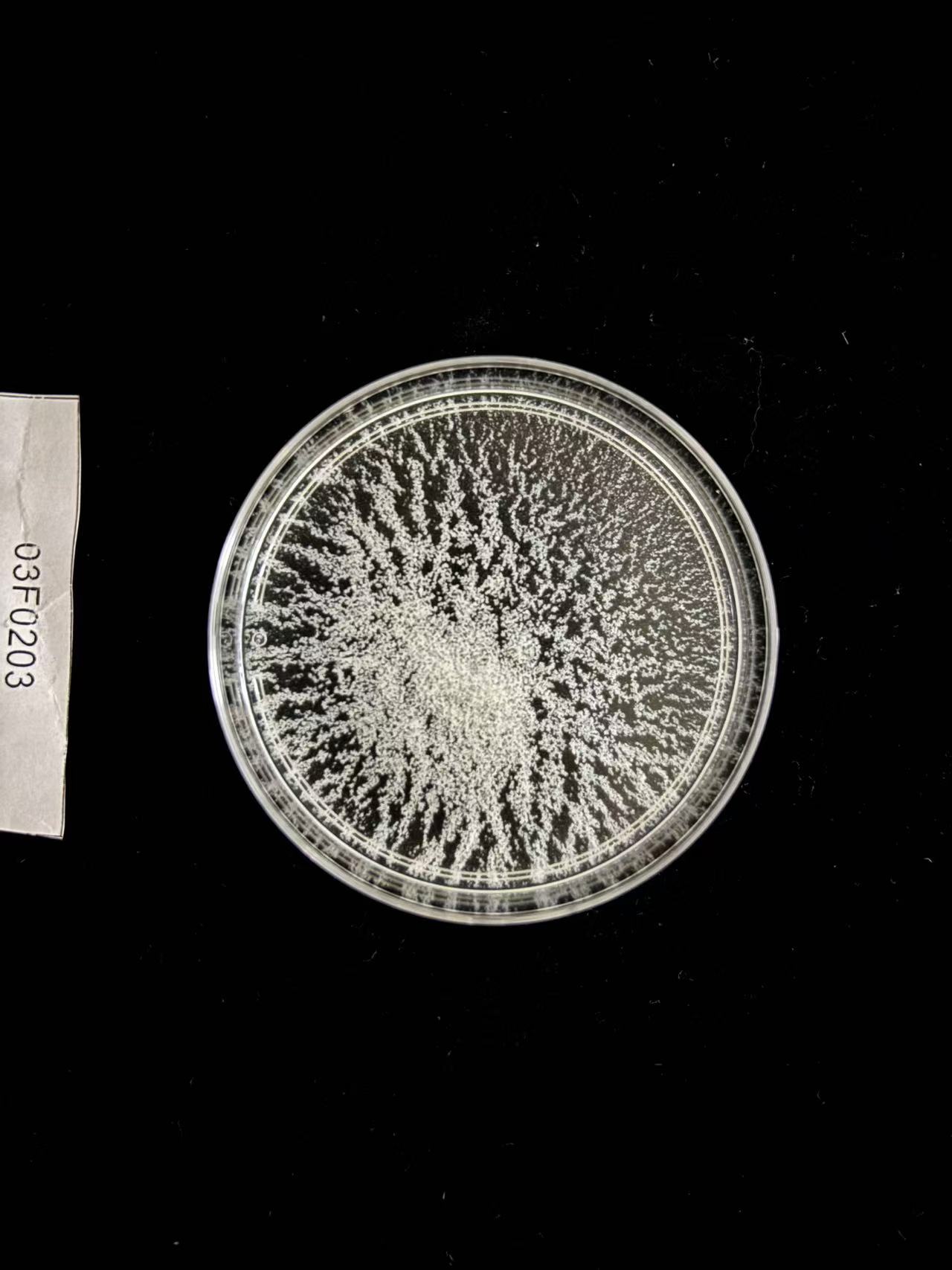

Supplement: Supplementary file 5 — Source data Fig. 3 [file 44319_2026_748_MOESM5_ESM.zip › Figure 3/3K/gsf2IE_Repeat 3.jpg]

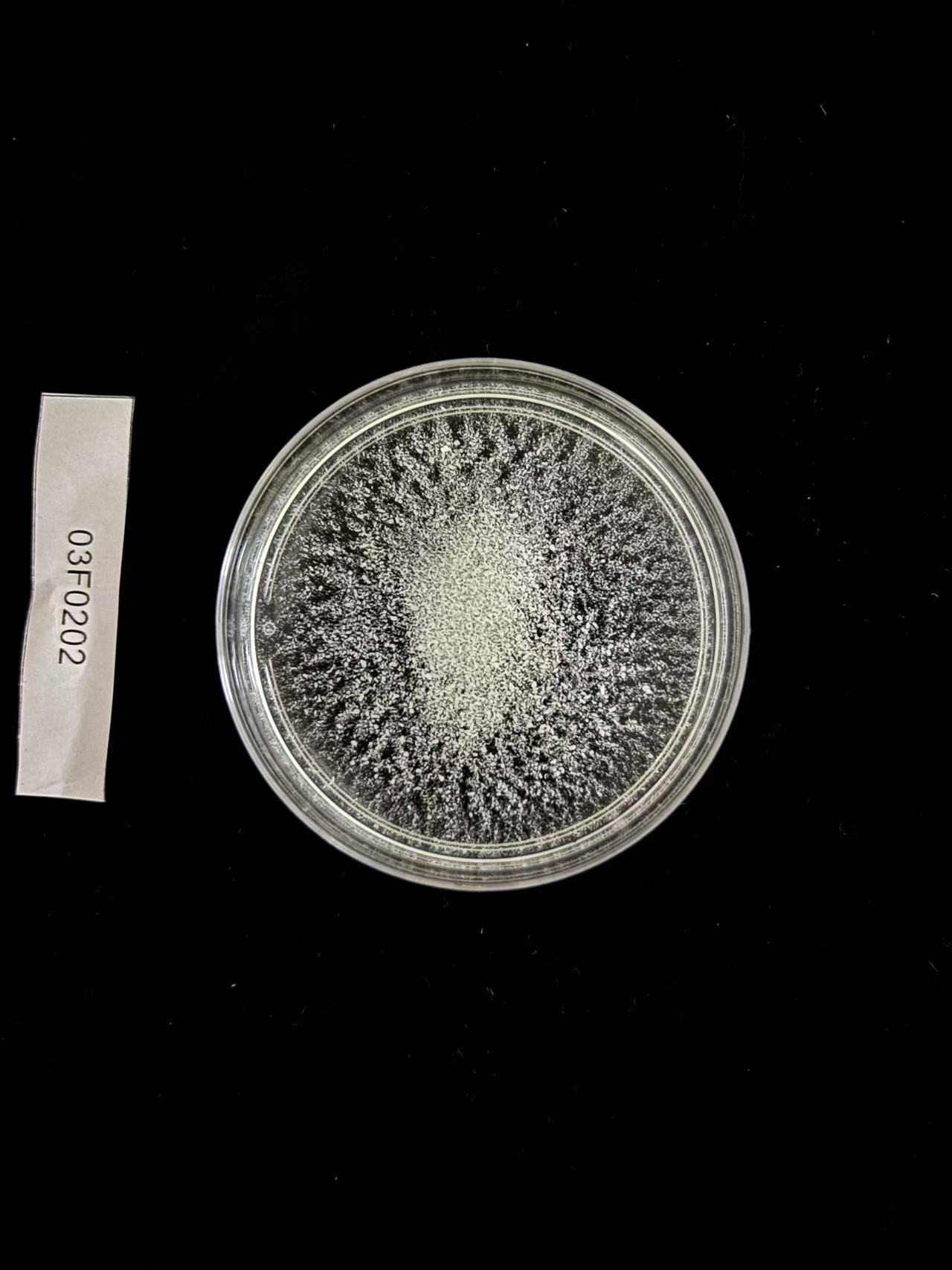

Supplement: Supplementary file 5 — Source data Fig. 3 [file 44319_2026_748_MOESM5_ESM.zip › Figure 3/3K/gsf2IE_Repeat 2.jpg]

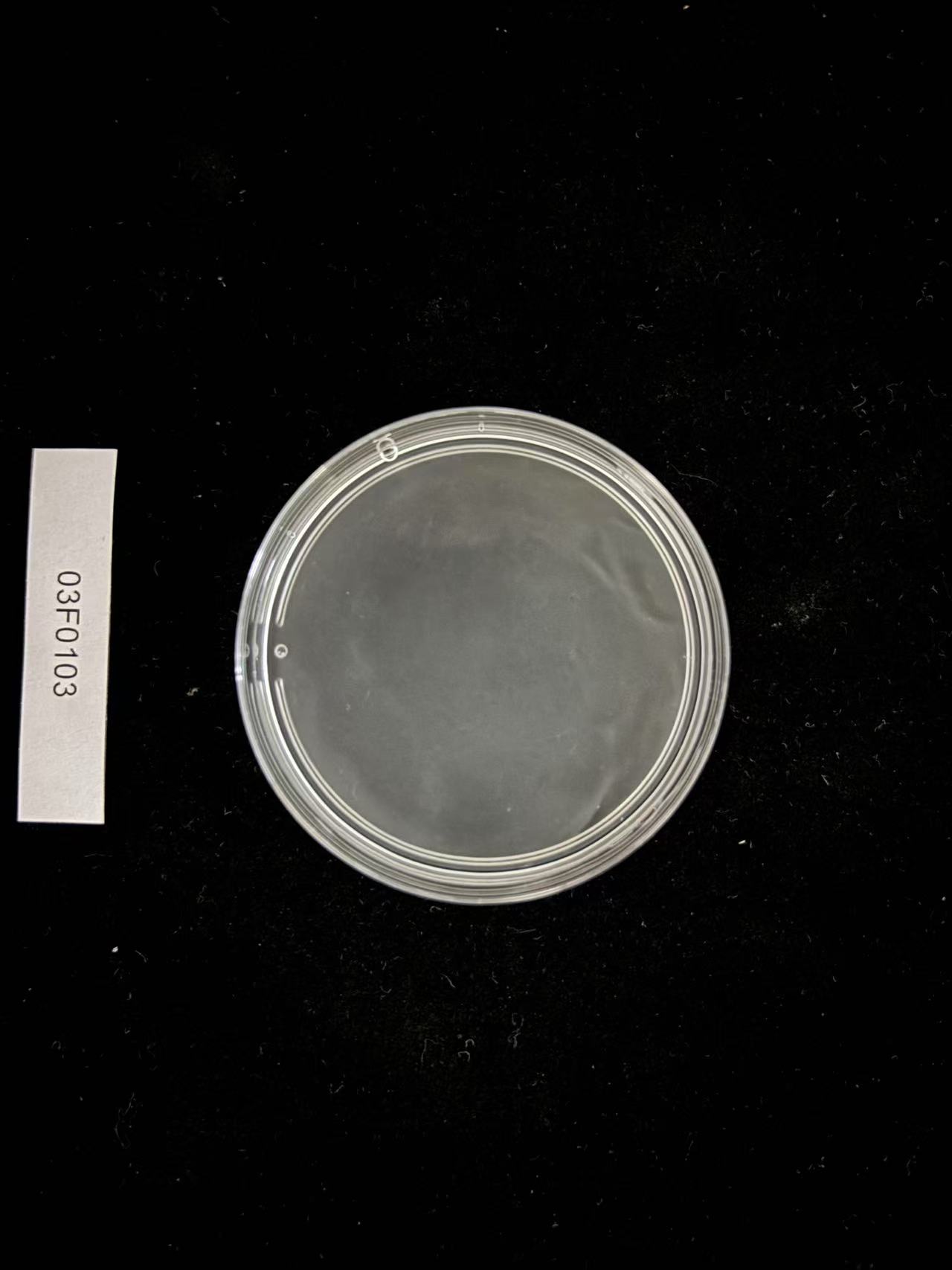

Supplement: Supplementary file 5 — Source data Fig. 3 [file 44319_2026_748_MOESM5_ESM.zip › Figure 3/3K/gsf2Γêå_Repeat 3.jpg]

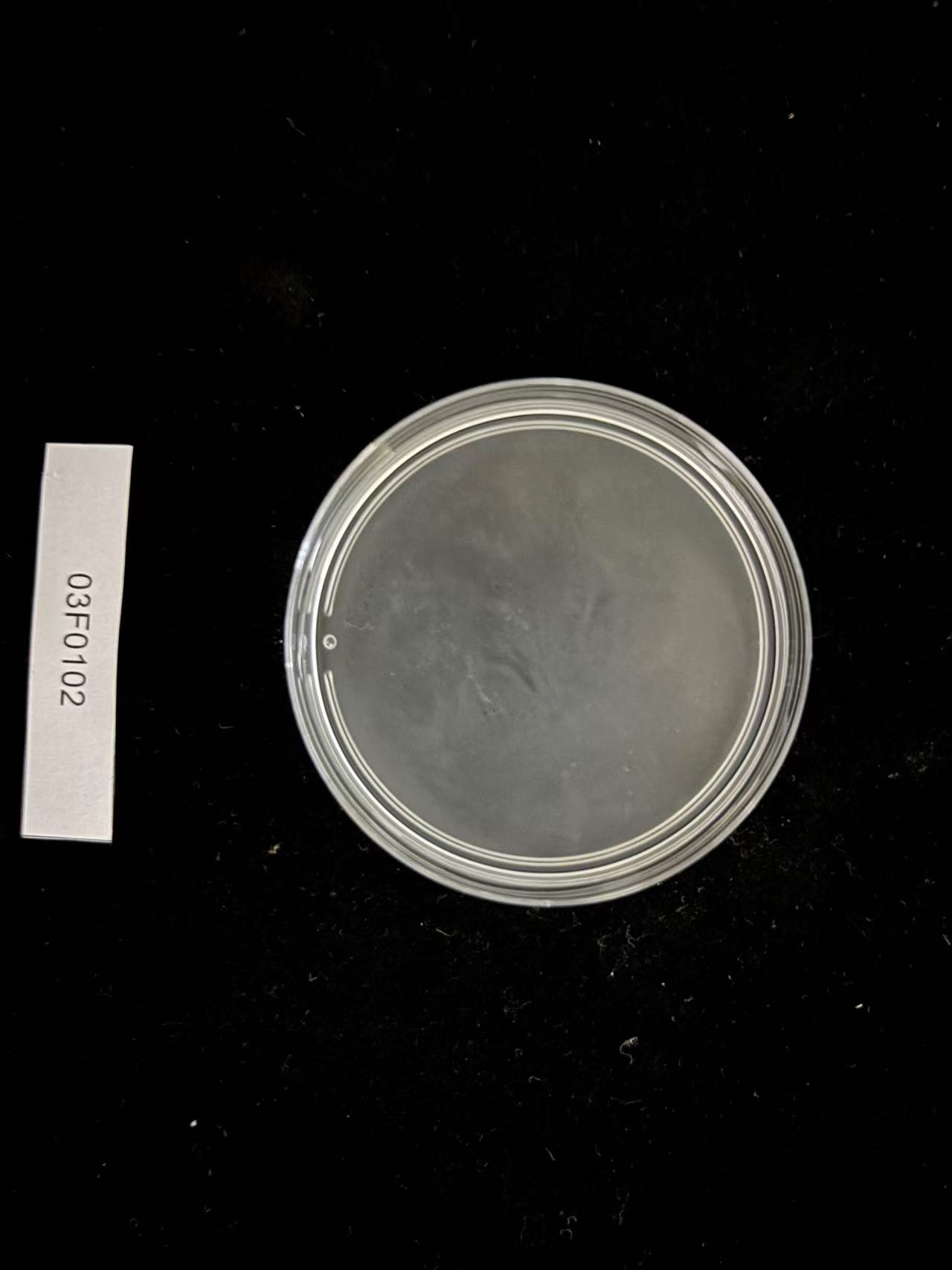

Supplement: Supplementary file 5 — Source data Fig. 3 [file 44319_2026_748_MOESM5_ESM.zip › Figure 3/3K/gsf2Γêå_Repeat 2.jpg]

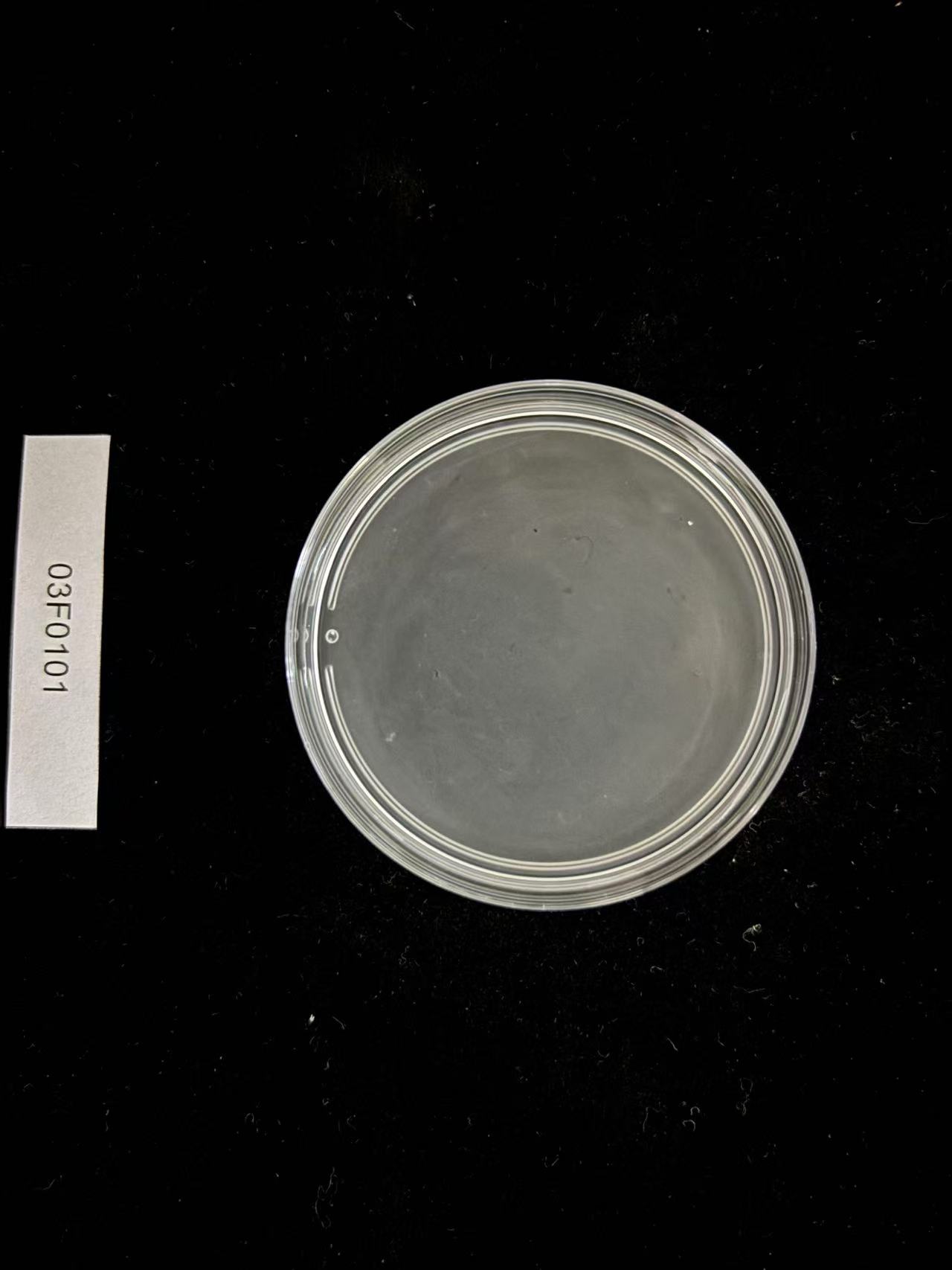

Supplement: Supplementary file 5 — Source data Fig. 3 [file 44319_2026_748_MOESM5_ESM.zip › Figure 3/3K/gsf2Γêå_Repeat 1.jpg]

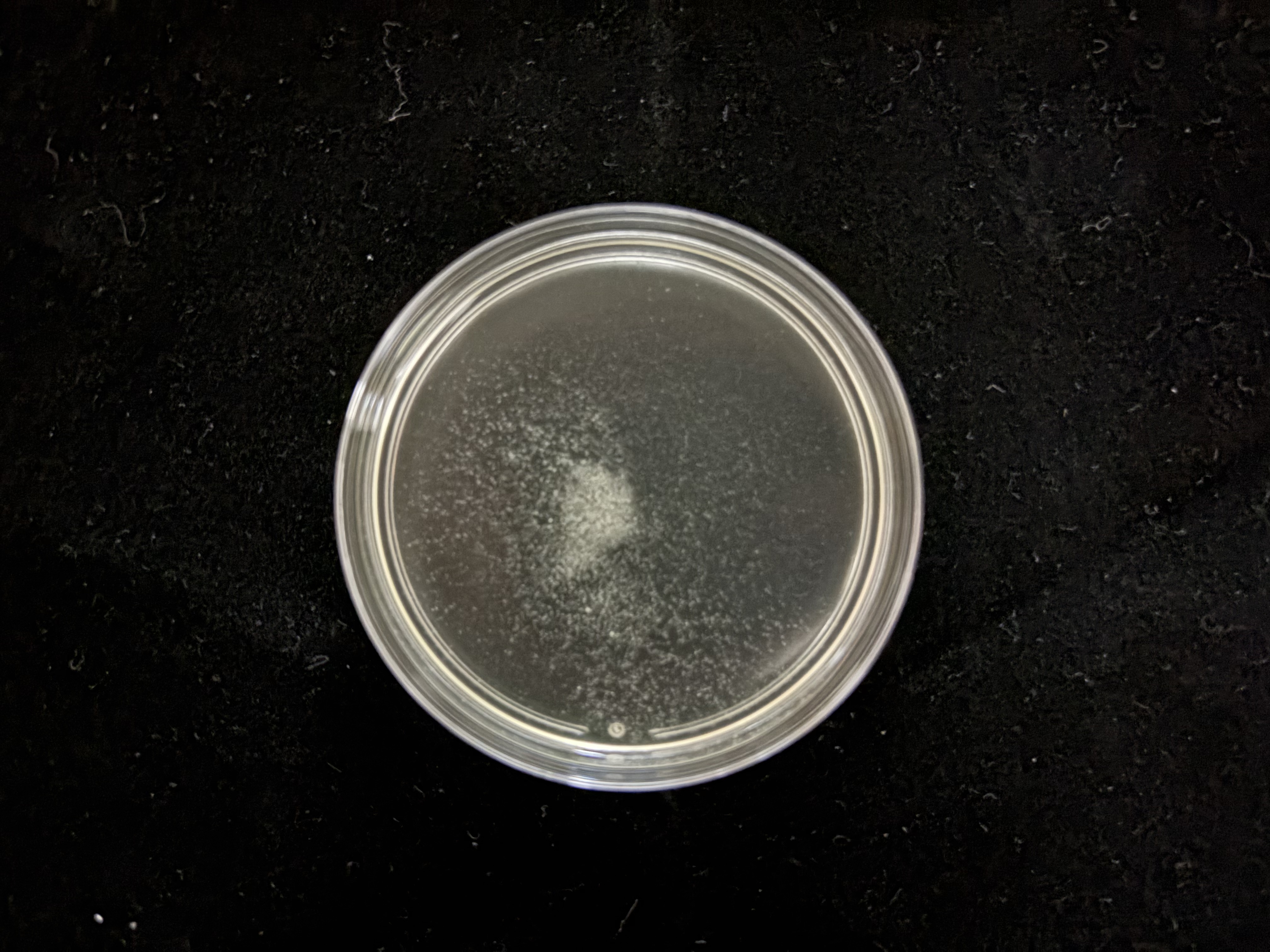

Supplement: Supplementary file 5 — Source data Fig. 3 [file 44319_2026_748_MOESM5_ESM.zip › Figure 3/3I/WT_Repeat 1.jpg]

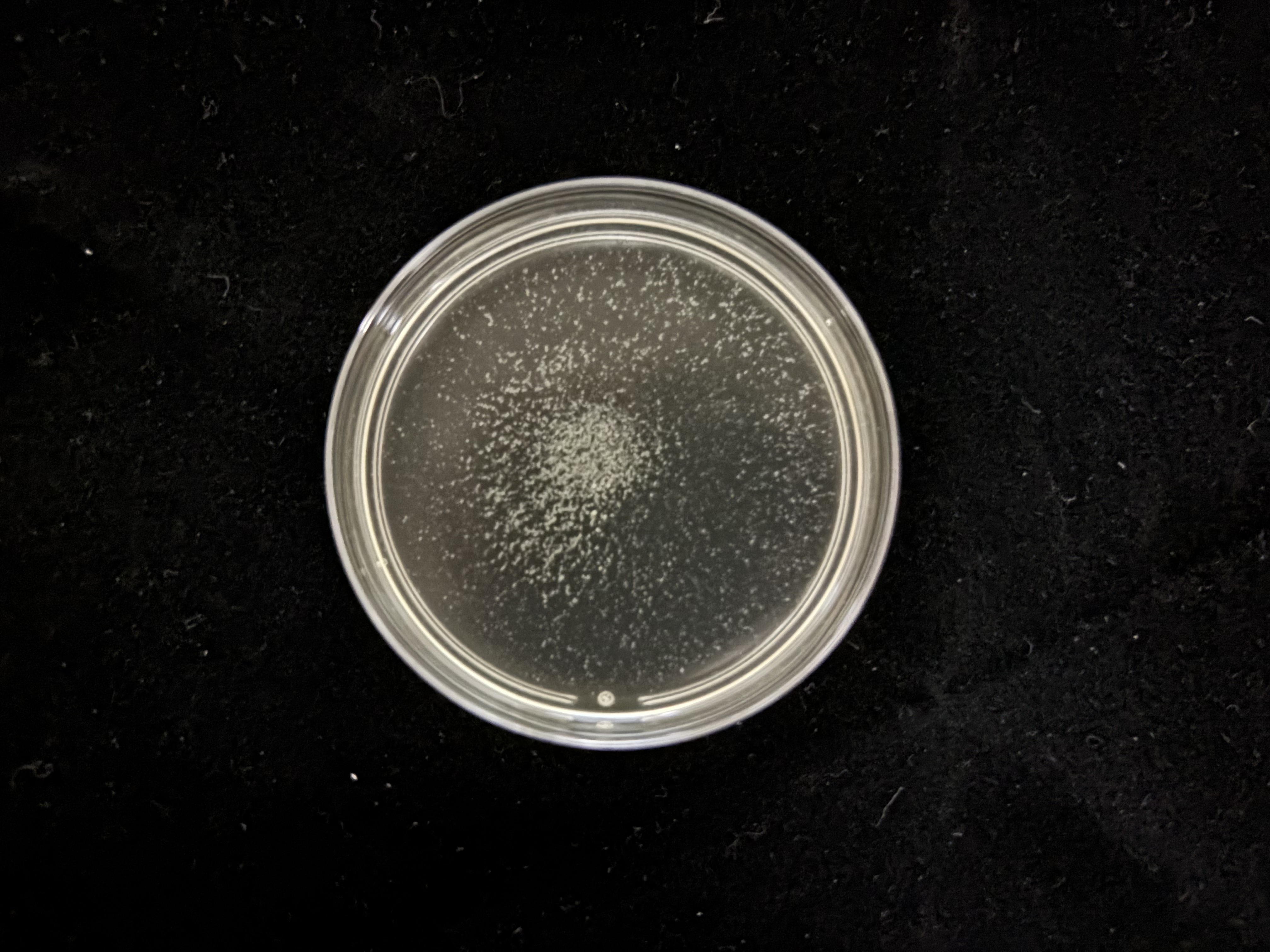

Supplement: Supplementary file 5 — Source data Fig. 3 [file 44319_2026_748_MOESM5_ESM.zip › Figure 3/3I/WT_Repeat 3.jpg]

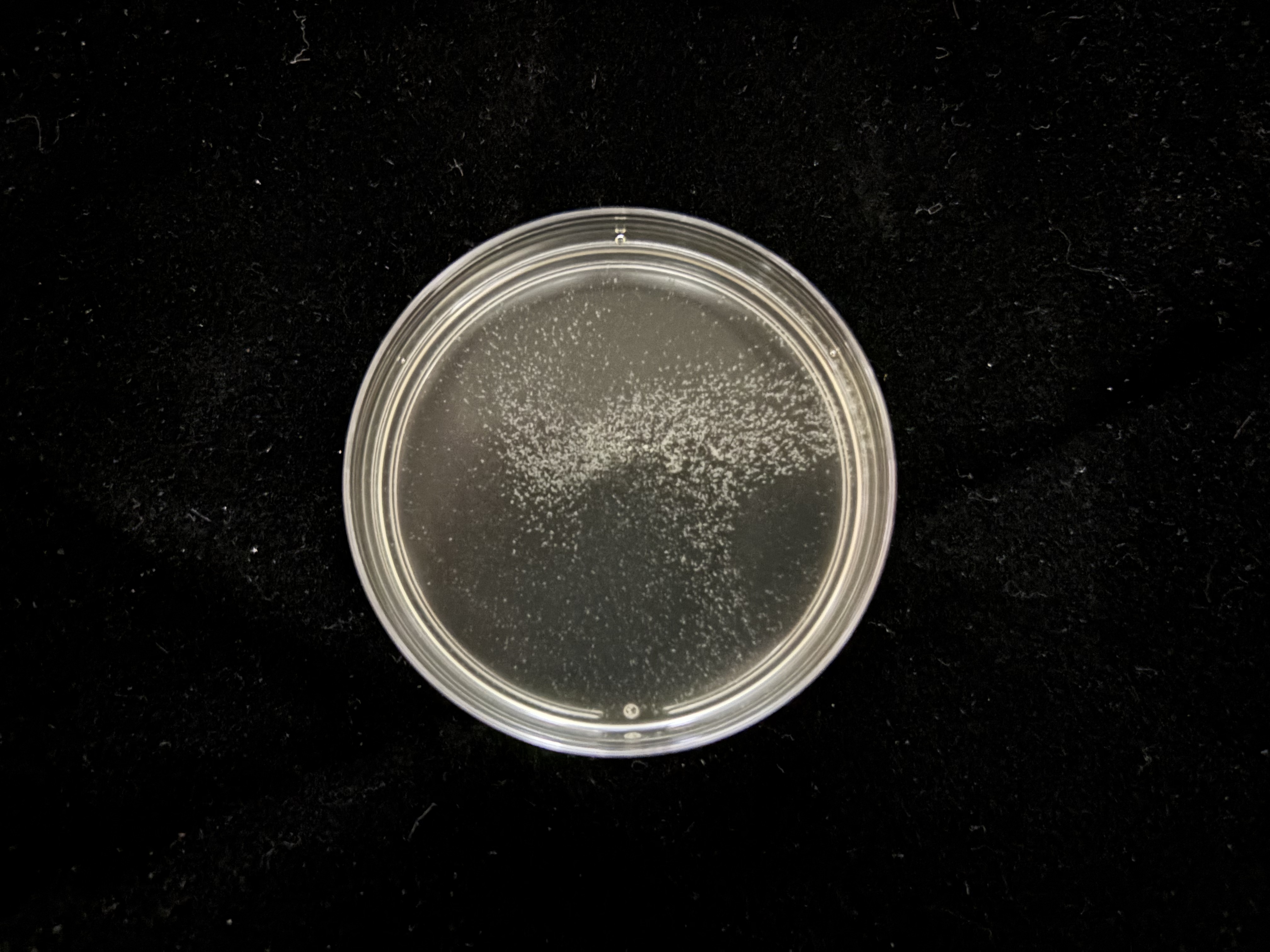

Supplement: Supplementary file 5 — Source data Fig. 3 [file 44319_2026_748_MOESM5_ESM.zip › Figure 3/3I/WT_Repeat 2.jpg]

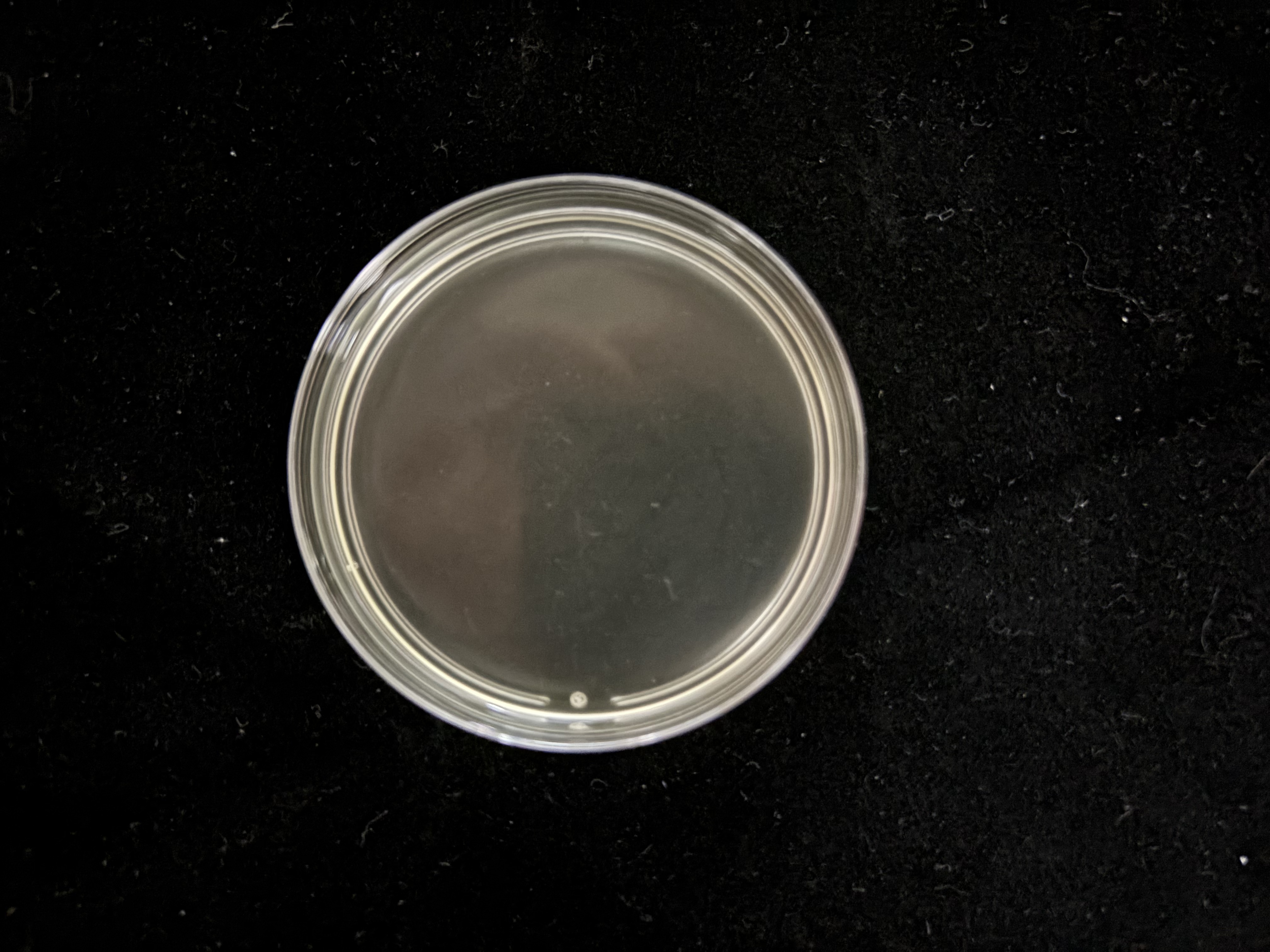

Supplement: Supplementary file 5 — Source data Fig. 3 [file 44319_2026_748_MOESM5_ESM.zip › Figure 3/3I/gsf2Γêå_Repeat 3.jpg]

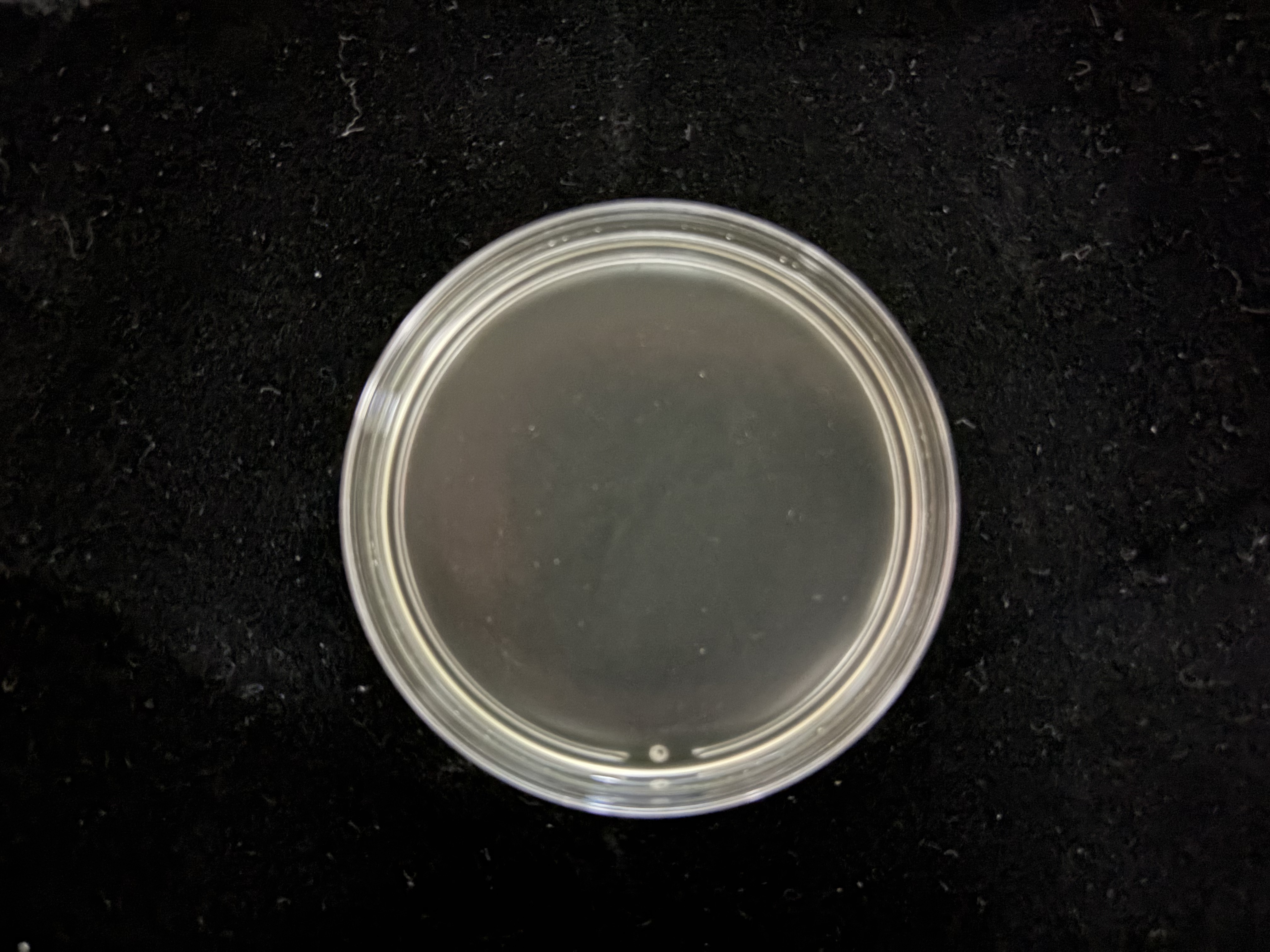

Supplement: Supplementary file 5 — Source data Fig. 3 [file 44319_2026_748_MOESM5_ESM.zip › Figure 3/3I/gsf2Γêå_Repeat 2.jpg]

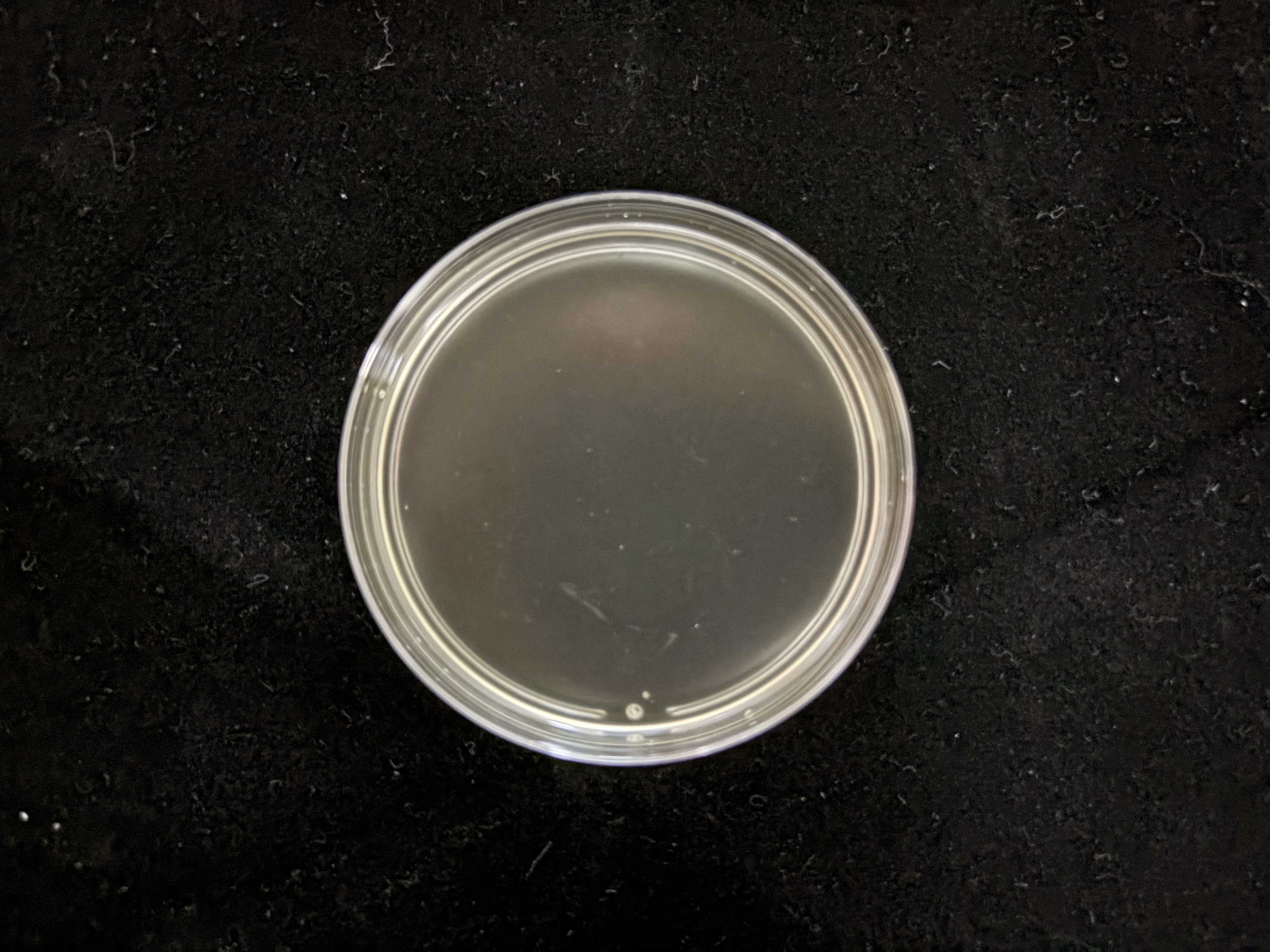

Supplement: Supplementary file 5 — Source data Fig. 3 [file 44319_2026_748_MOESM5_ESM.zip › Figure 3/3I/gsf2Γêå_Repeat 1.jpg]

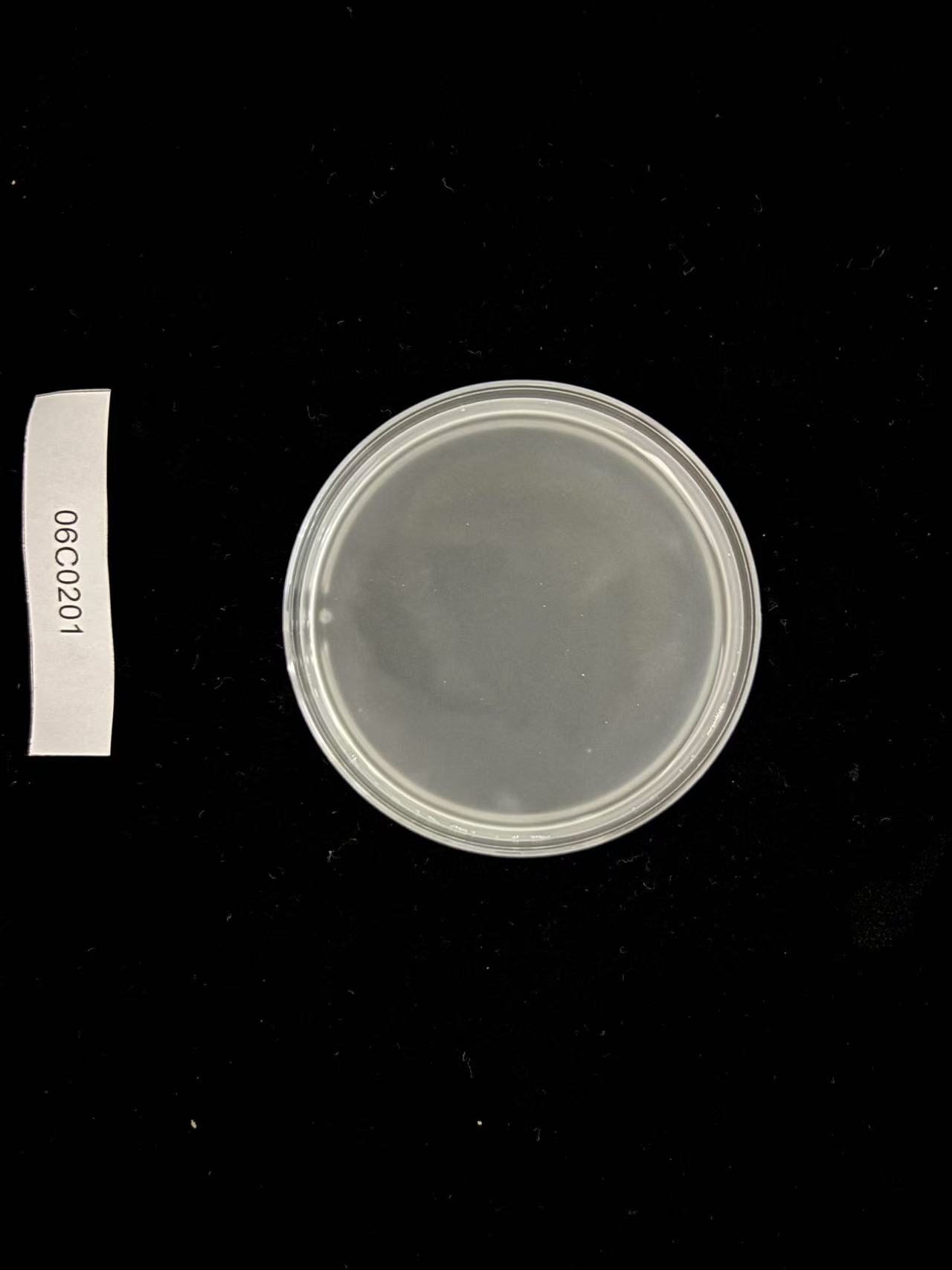

Supplement: Supplementary file 7 — Source data Fig. 6 [file 44319_2026_748_MOESM7_ESM.zip › Figure 6/6E/gsf2Γêå.jpg]

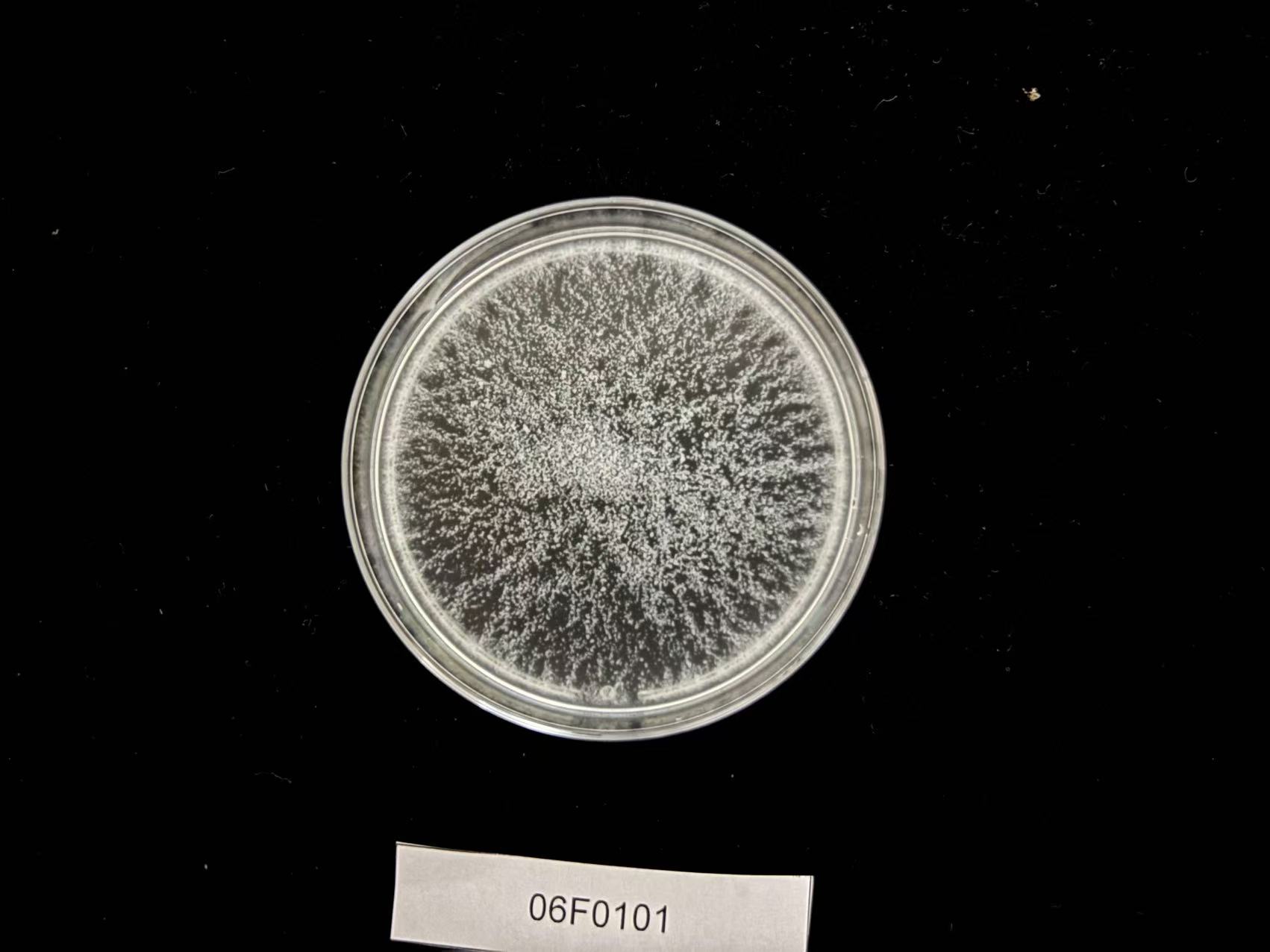

Supplement: Supplementary file 7 — Source data Fig. 6 [file 44319_2026_748_MOESM7_ESM.zip › Figure 6/6E/gsf2AIE.jpg]

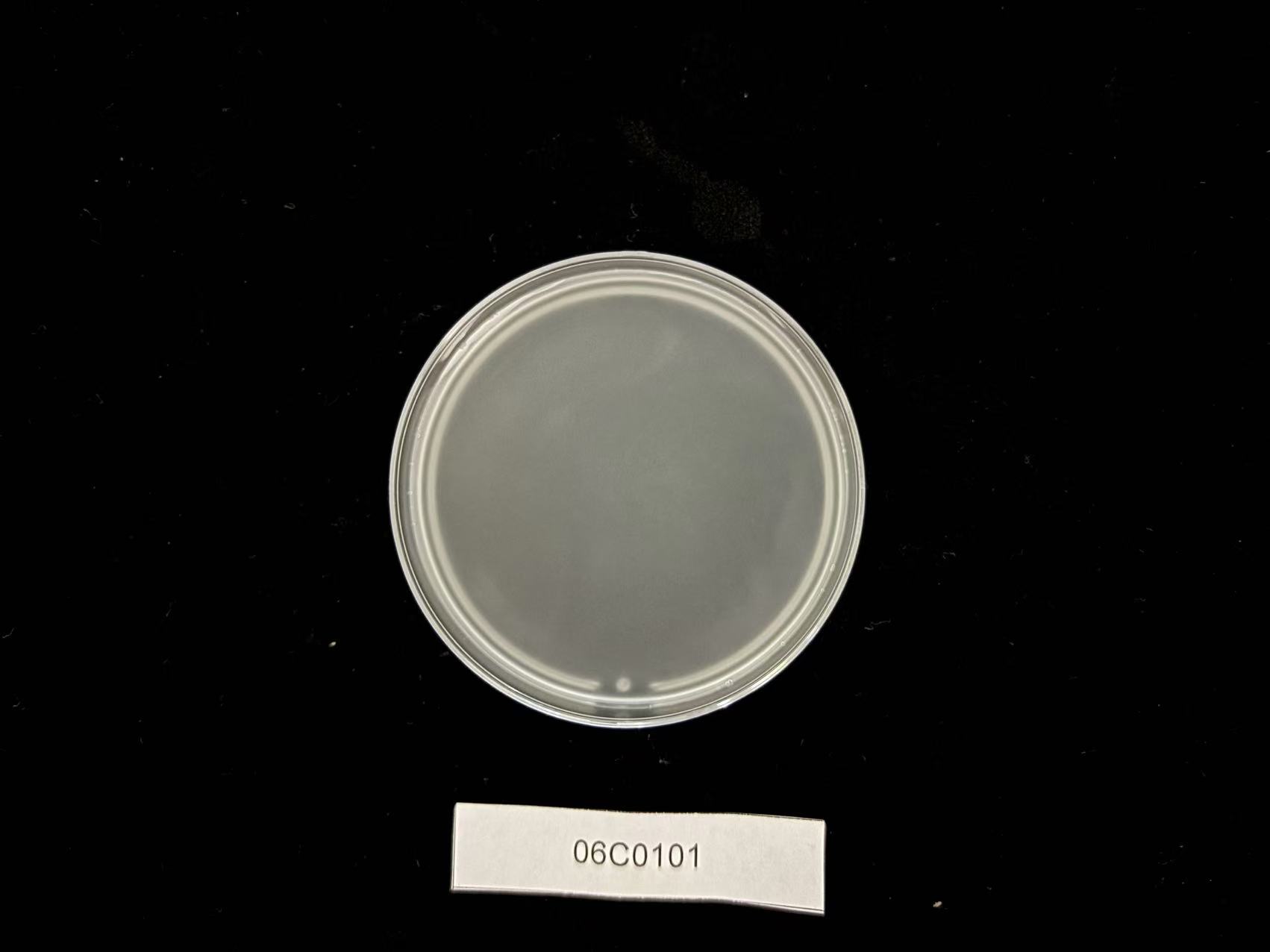

Supplement: Supplementary file 7 — Source data Fig. 6 [file 44319_2026_748_MOESM7_ESM.zip › Figure 6/6E/06c0101.jpg]

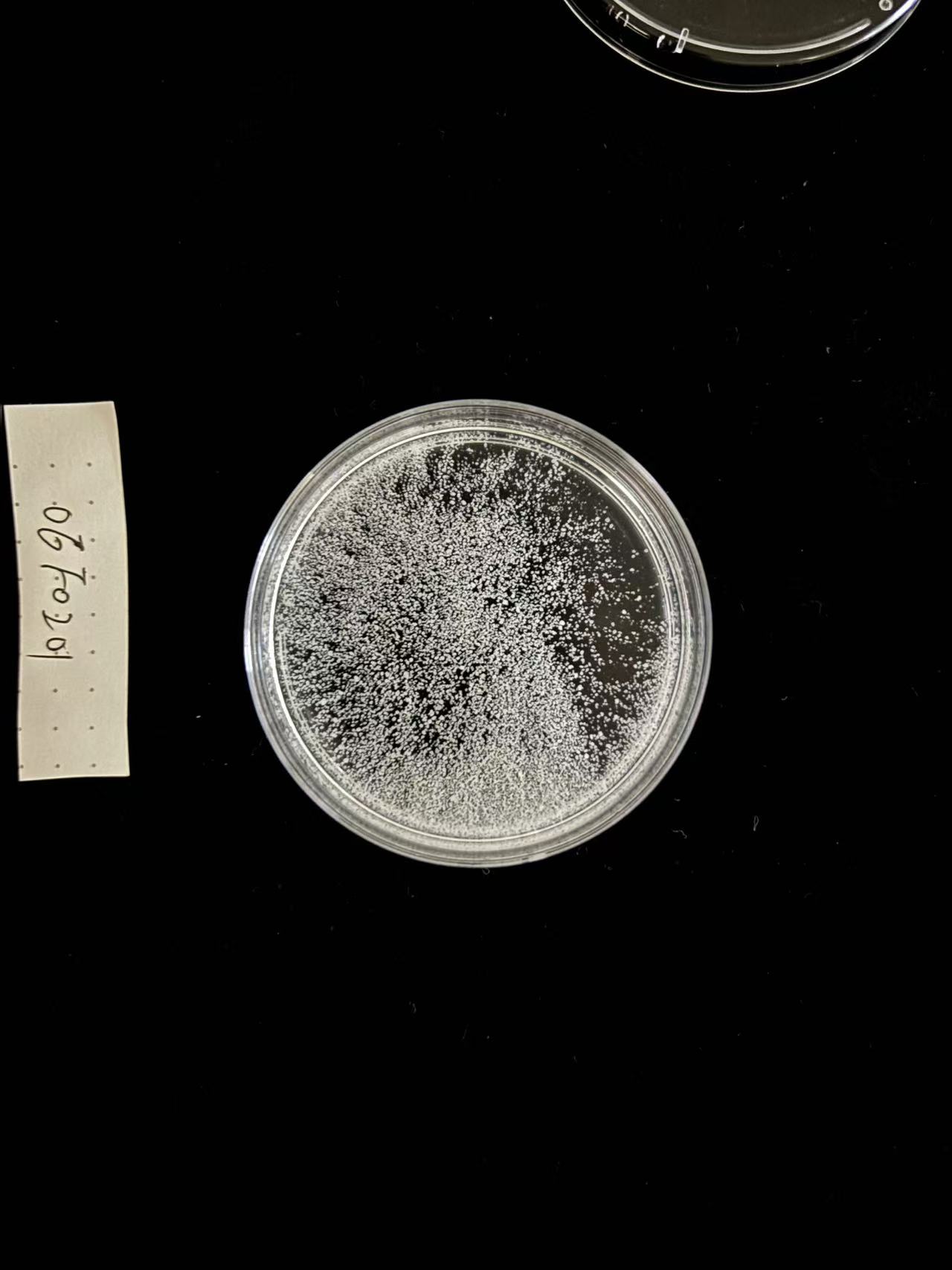

Supplement: Supplementary file 7 — Source data Fig. 6 [file 44319_2026_748_MOESM7_ESM.zip › Figure 6/6E/06F0201.jpg]

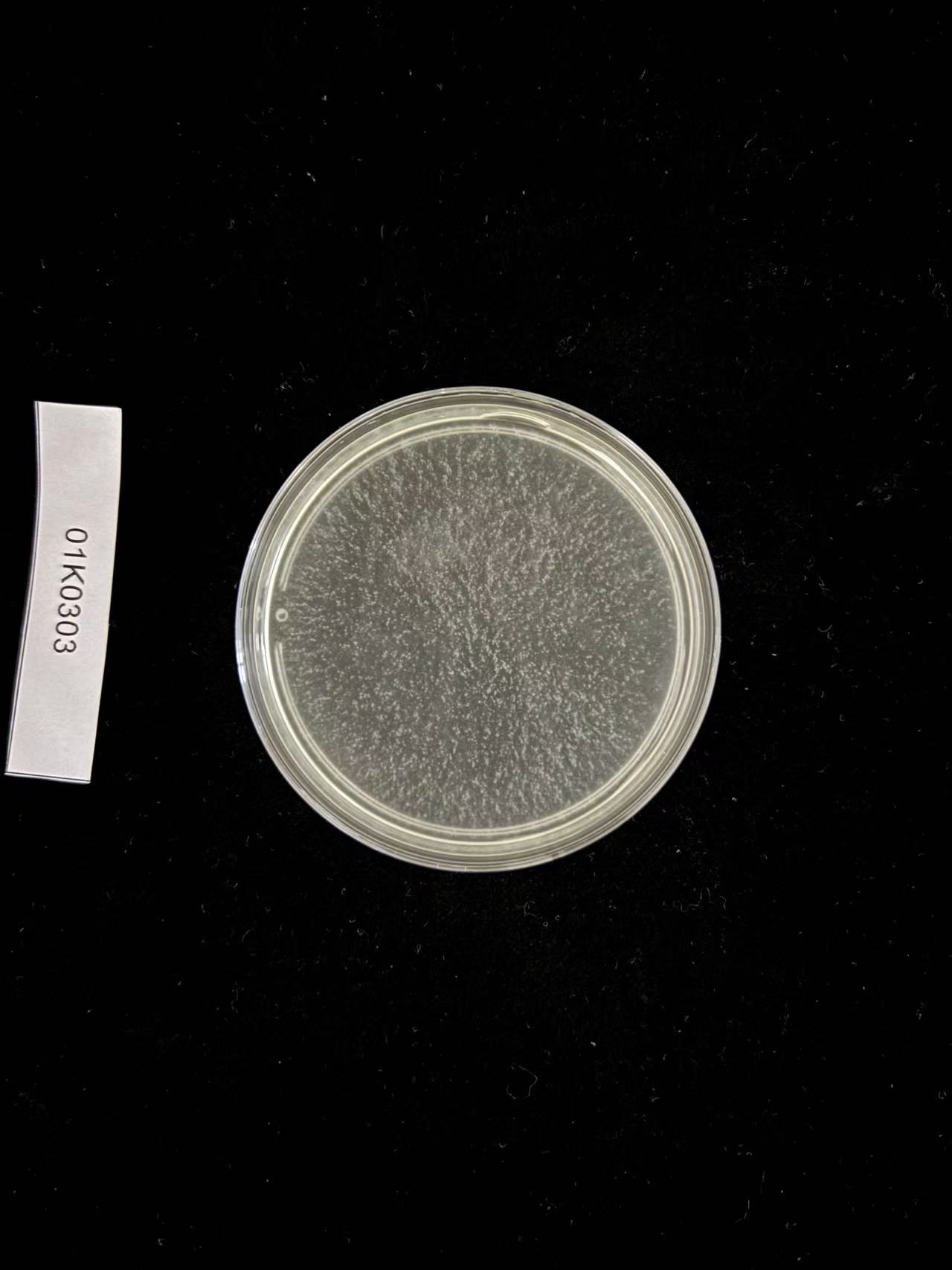

Supplement: Supplementary file 10 — Appendix Figure S1 Source Data [file 44319_2026_748_MOESM10_ESM.zip › Appendix Figure S1/S1F/gsf2IE_Repeat3.jpg]

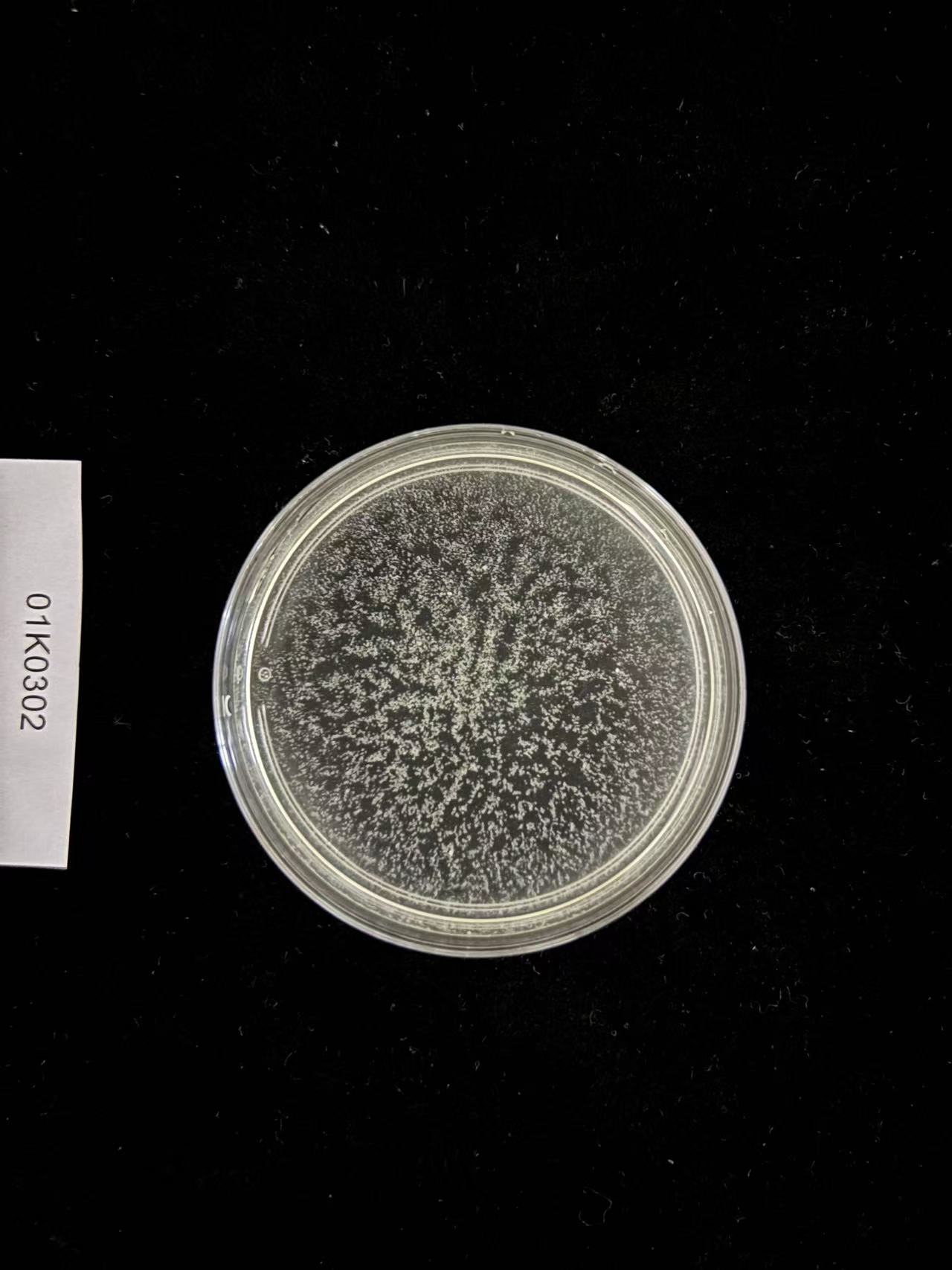

Supplement: Supplementary file 10 — Appendix Figure S1 Source Data [file 44319_2026_748_MOESM10_ESM.zip › Appendix Figure S1/S1F/gsf2IE_Repeat2.jpg]

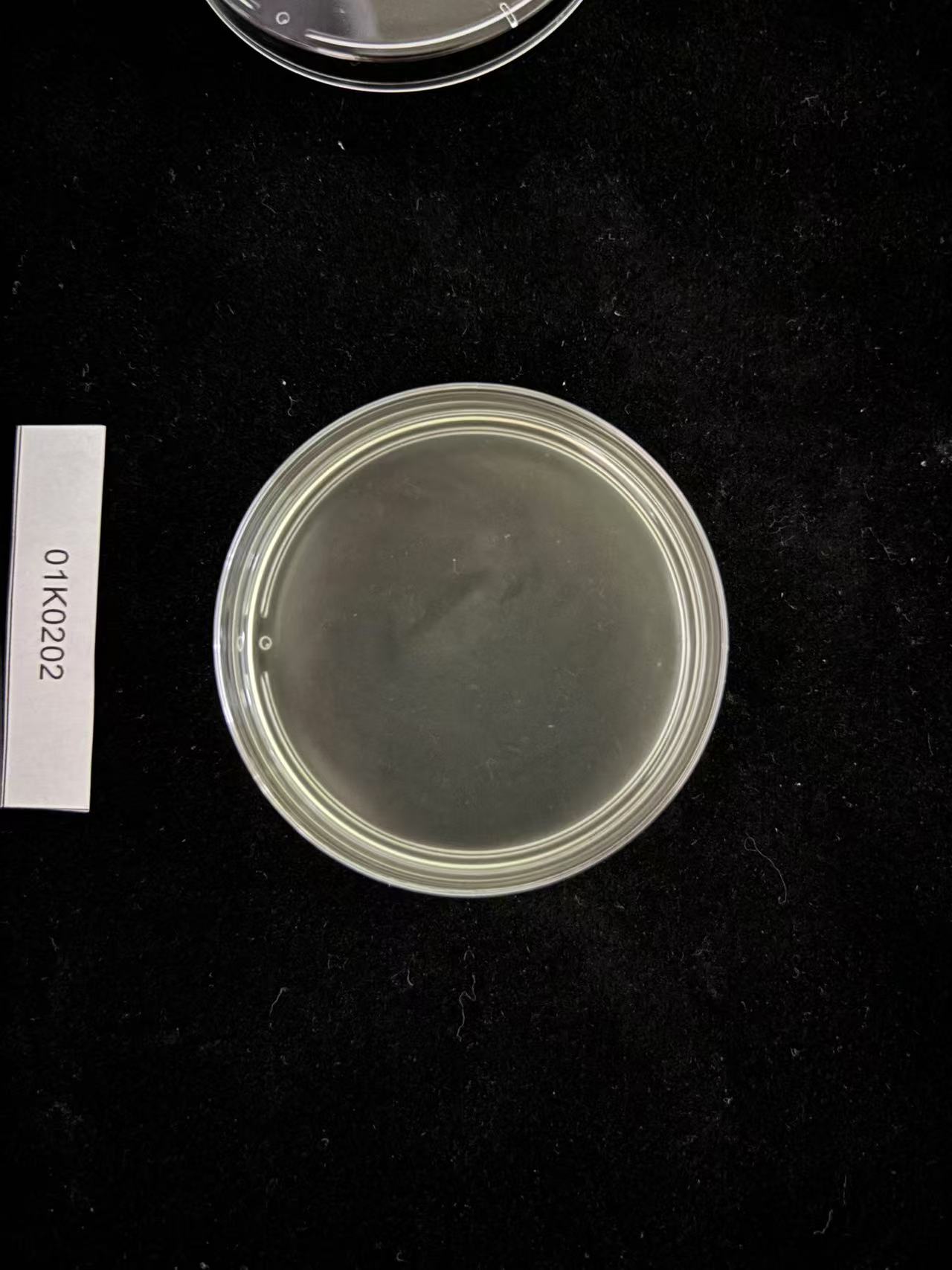

Supplement: Supplementary file 10 — Appendix Figure S1 Source Data [file 44319_2026_748_MOESM10_ESM.zip › Appendix Figure S1/S1F/gsf2Γêå_Repeat2.jpg]

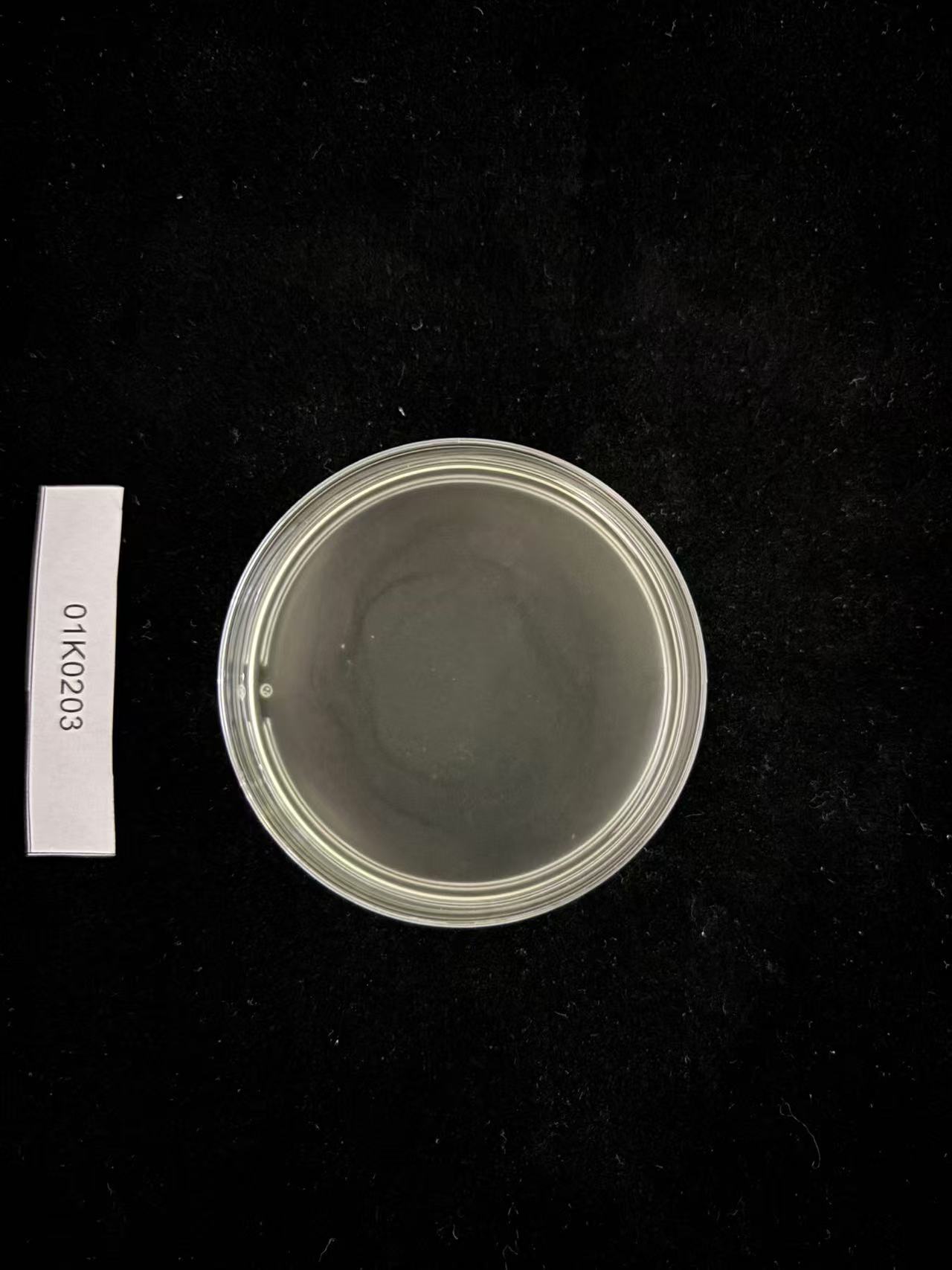

Supplement: Supplementary file 10 — Appendix Figure S1 Source Data [file 44319_2026_748_MOESM10_ESM.zip › Appendix Figure S1/S1F/gsf2Γêå_Repeat3.jpg]

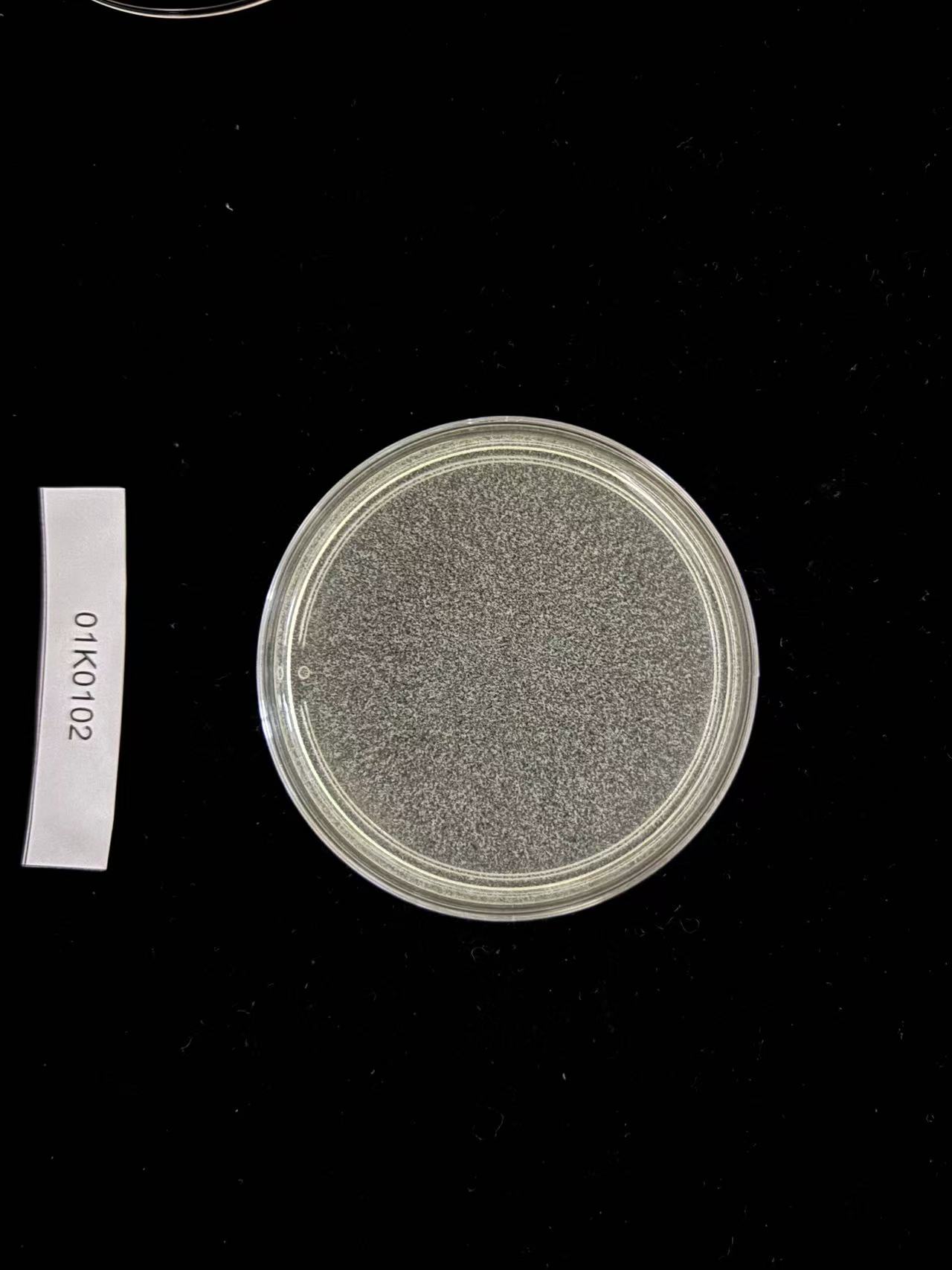

Supplement: Supplementary file 10 — Appendix Figure S1 Source Data [file 44319_2026_748_MOESM10_ESM.zip › Appendix Figure S1/S1F/WT_Repeat2.jpg]

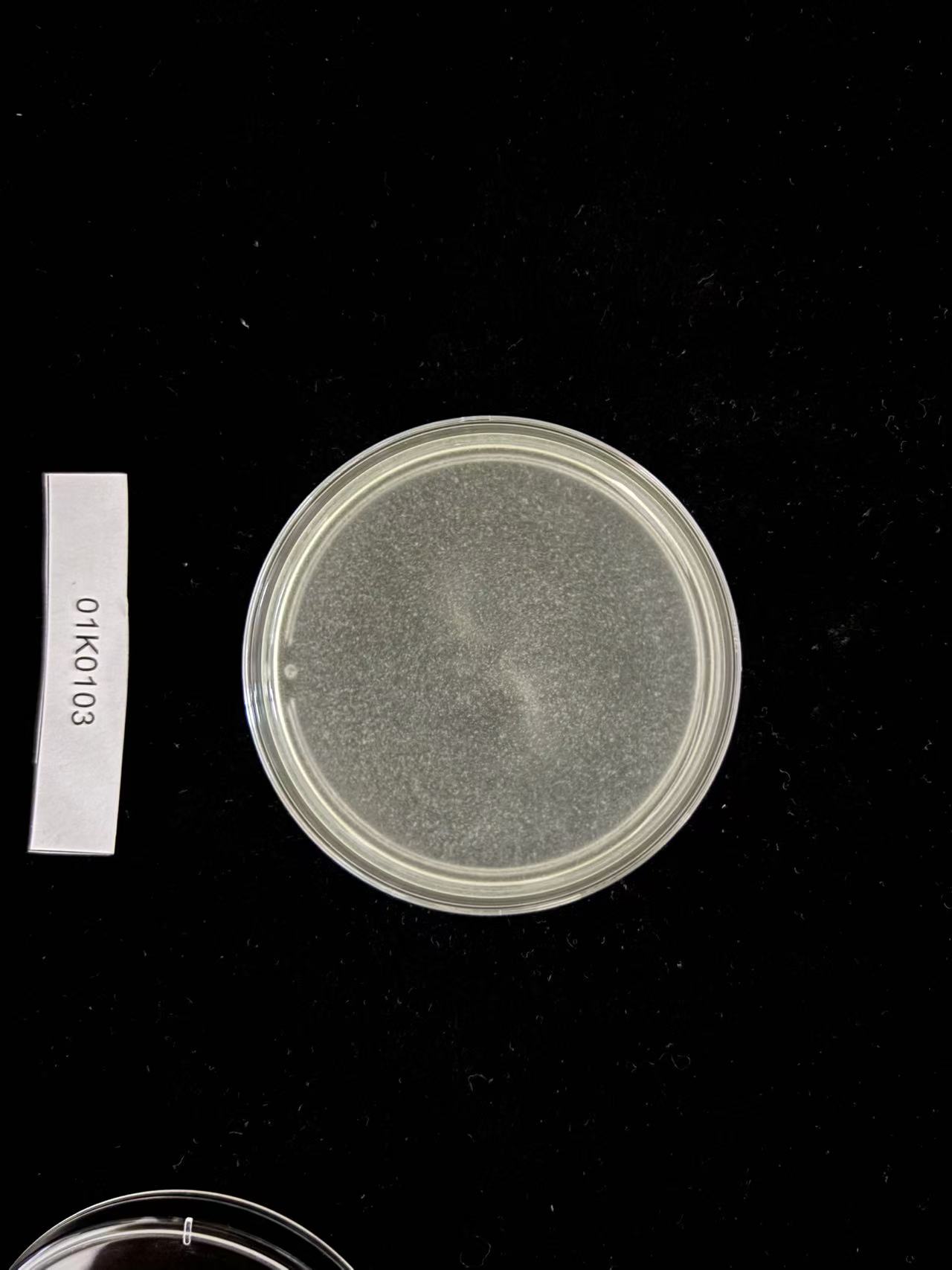

Supplement: Supplementary file 10 — Appendix Figure S1 Source Data [file 44319_2026_748_MOESM10_ESM.zip › Appendix Figure S1/S1F/WT_Repeat3.jpg]

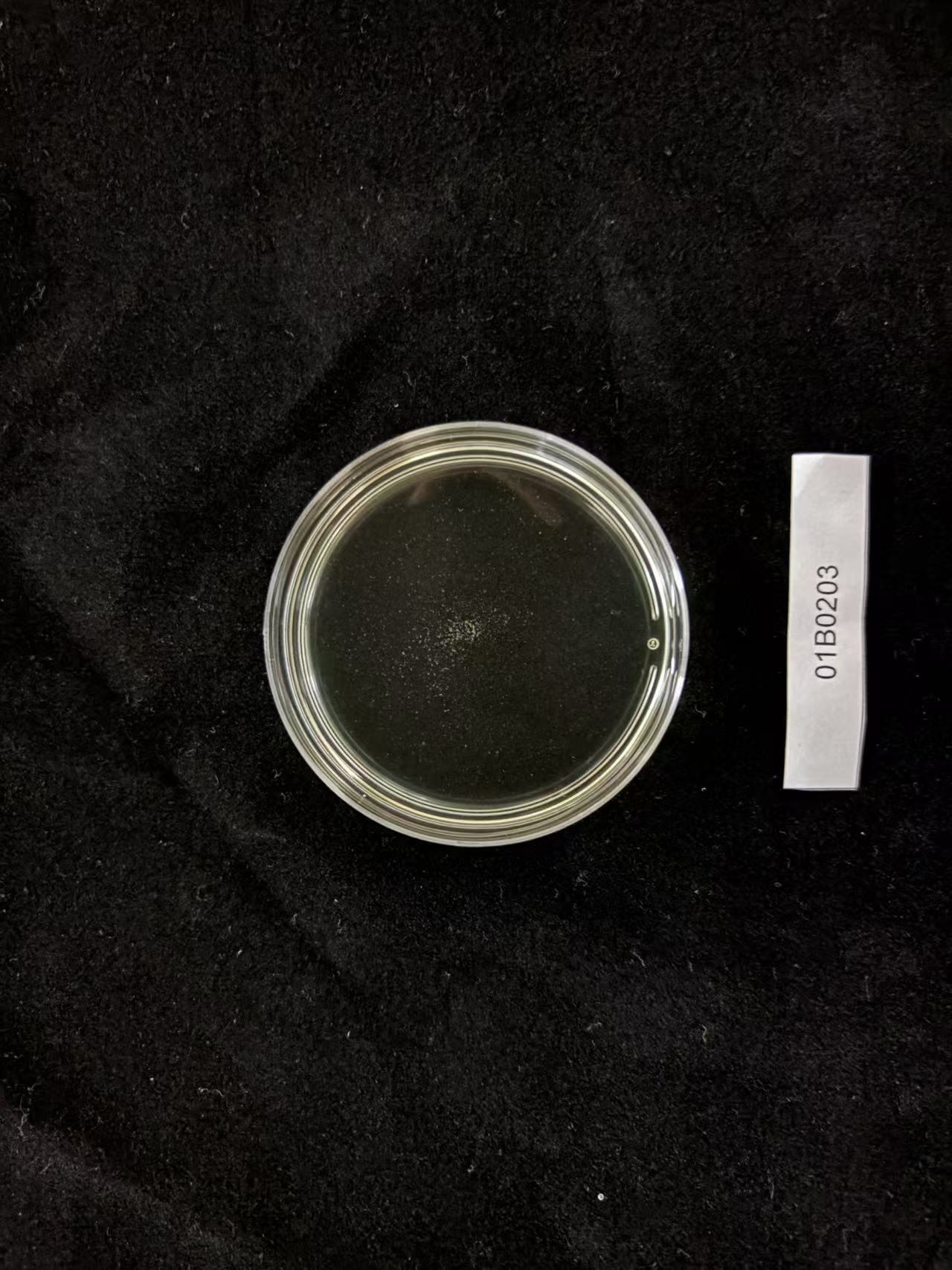

Supplement: Supplementary file 10 — Appendix Figure S1 Source Data [file 44319_2026_748_MOESM10_ESM.zip › Appendix Figure S1/S1A/Treated_Repeat3.jpg]

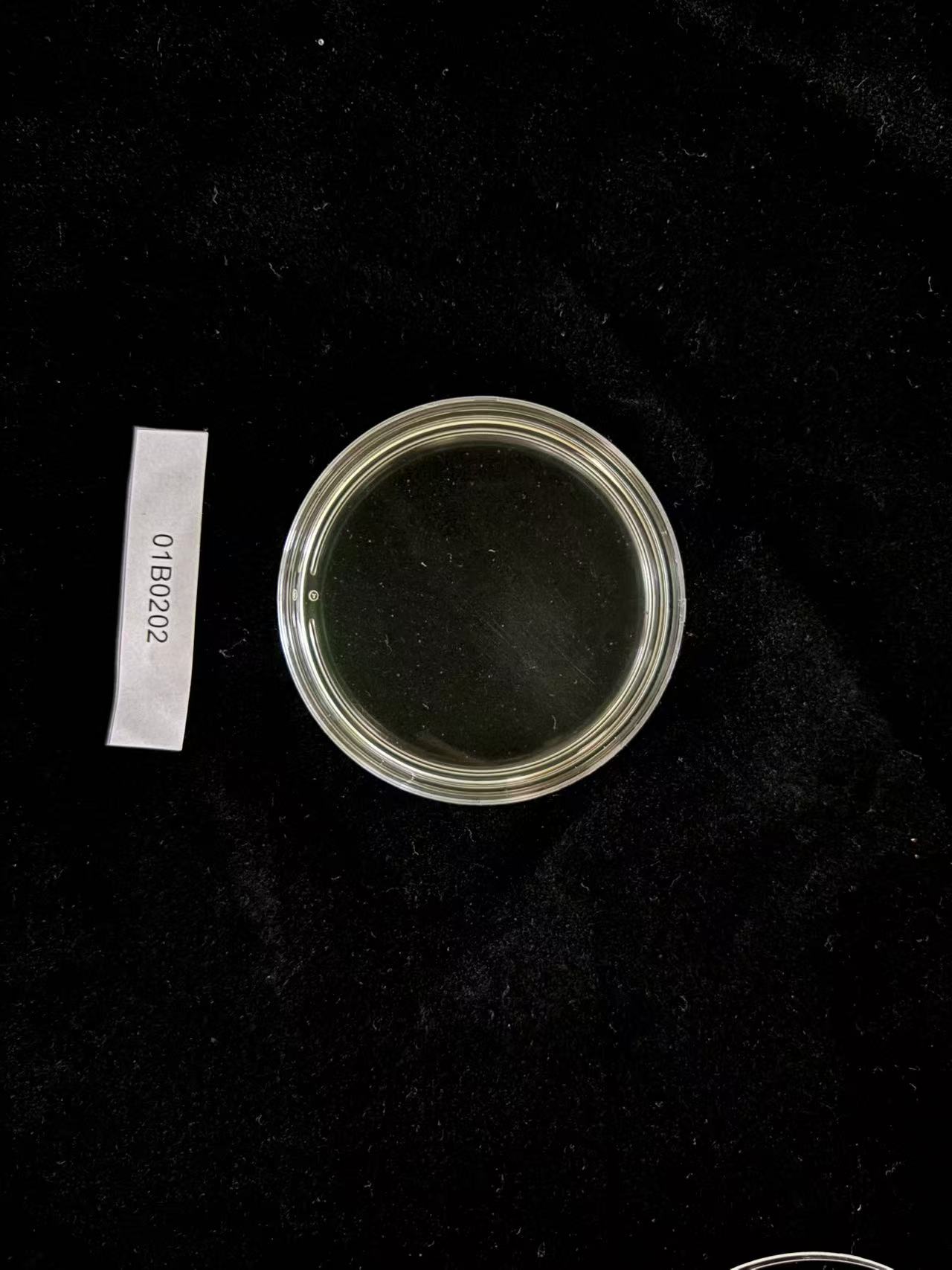

Supplement: Supplementary file 10 — Appendix Figure S1 Source Data [file 44319_2026_748_MOESM10_ESM.zip › Appendix Figure S1/S1A/Treated_Repeat2.jpg]

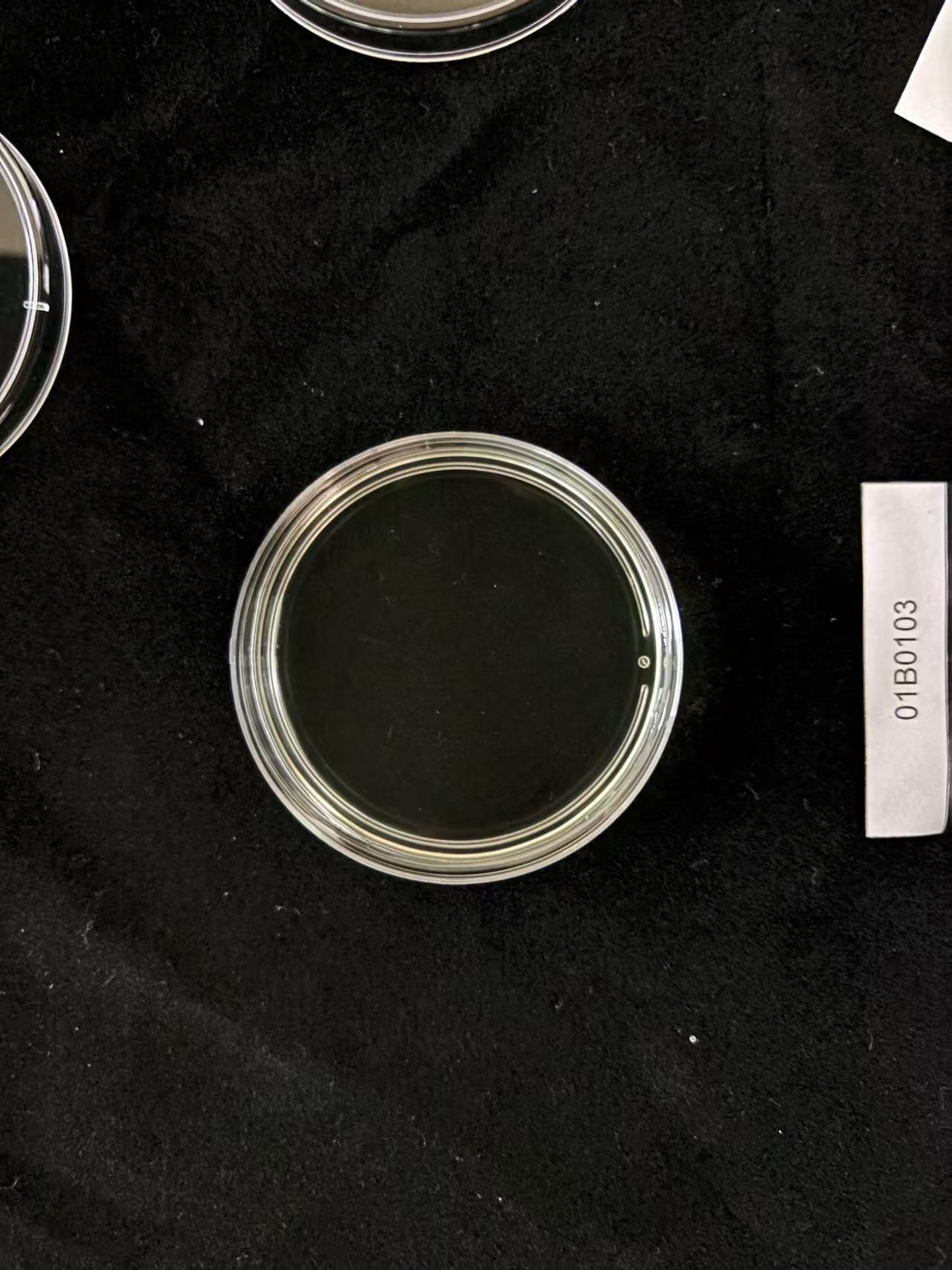

Supplement: Supplementary file 10 — Appendix Figure S1 Source Data [file 44319_2026_748_MOESM10_ESM.zip › Appendix Figure S1/S1A/Control_Repeat3.jpg]

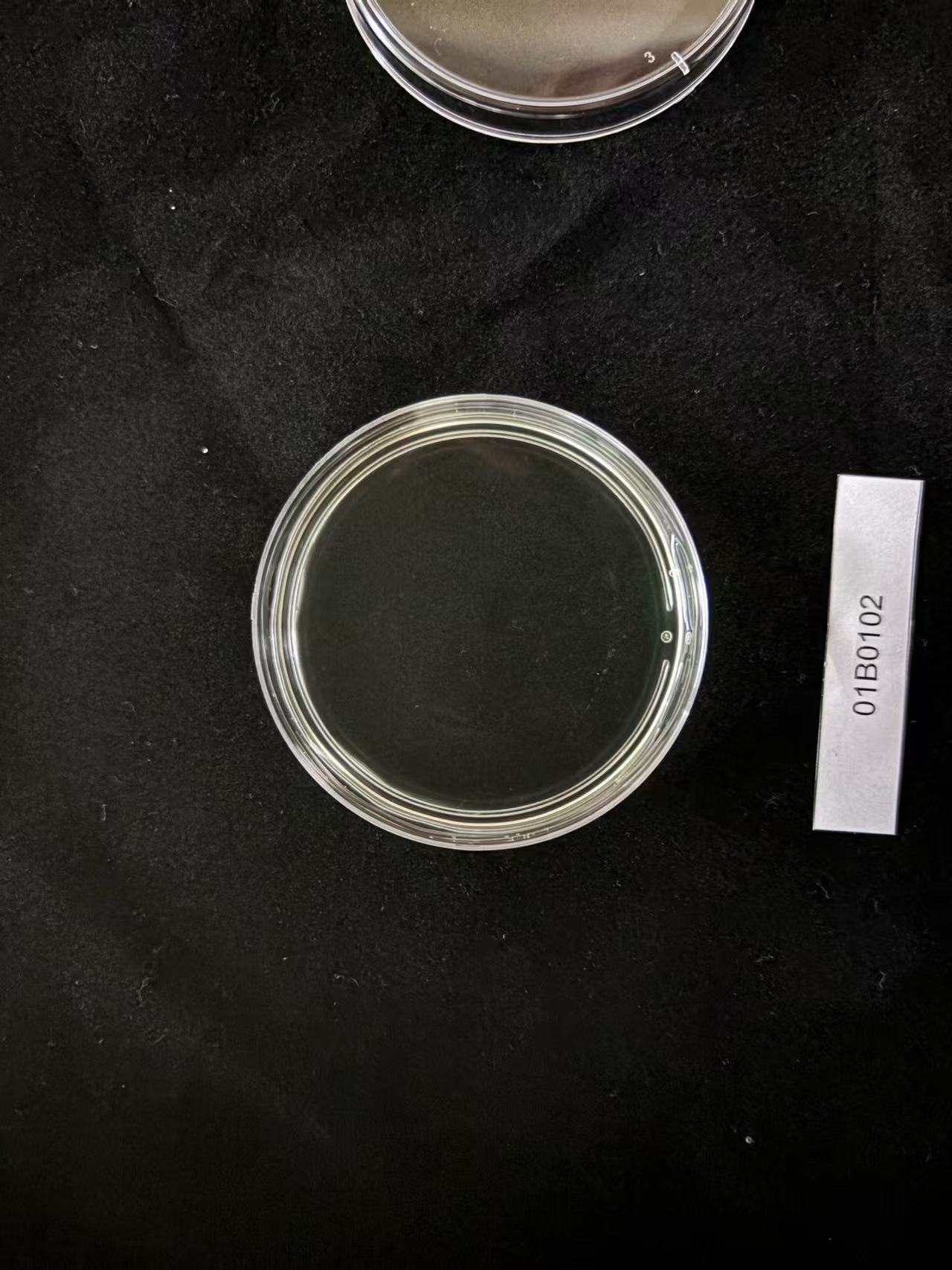

Supplement: Supplementary file 10 — Appendix Figure S1 Source Data [file 44319_2026_748_MOESM10_ESM.zip › Appendix Figure S1/S1A/Control_Repeat2.jpg]

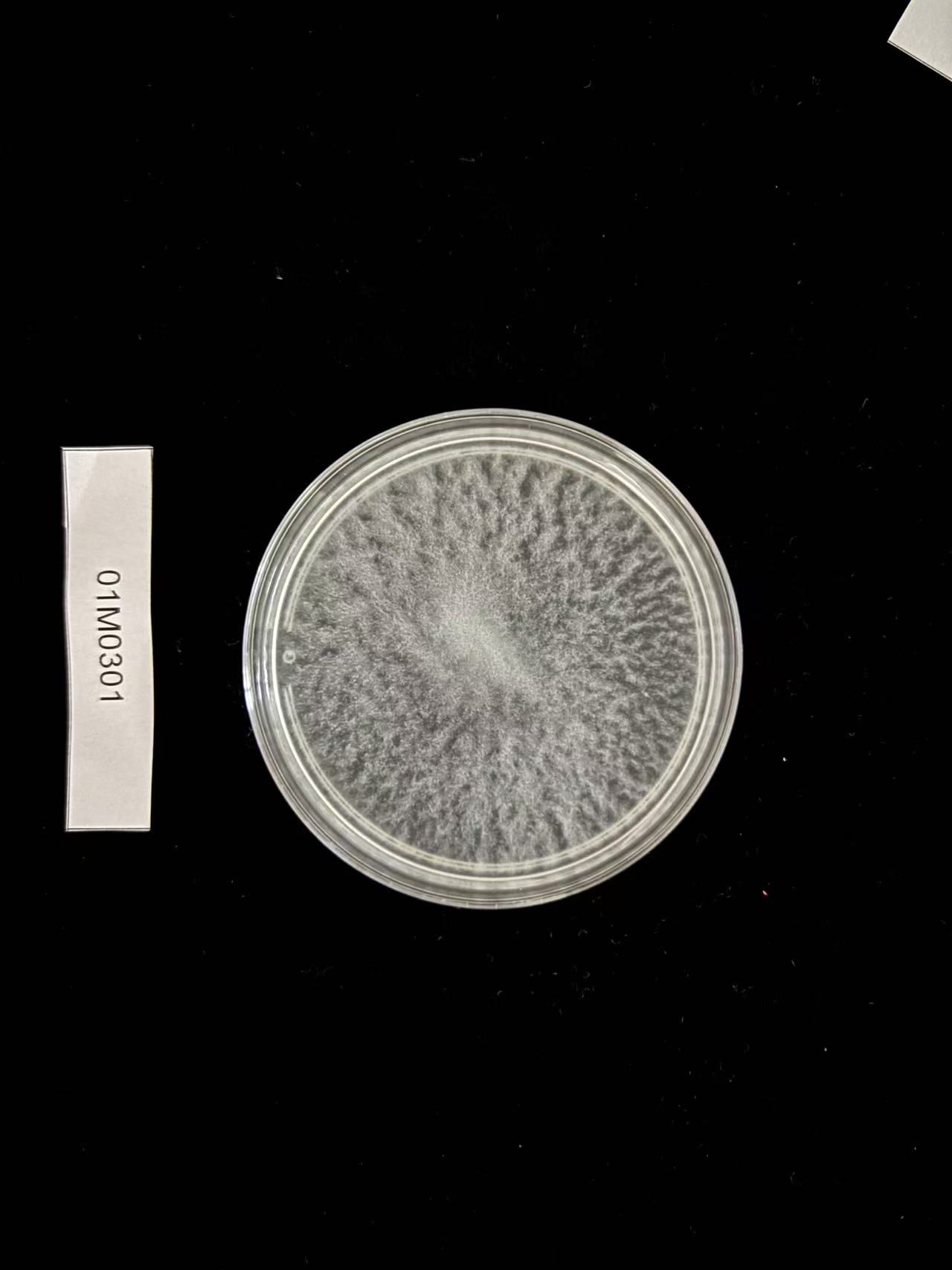

Supplement: Supplementary file 10 — Appendix Figure S1 Source Data [file 44319_2026_748_MOESM10_ESM.zip › Appendix Figure S1/S1D/gsf2IE_Repeat3.jpg]

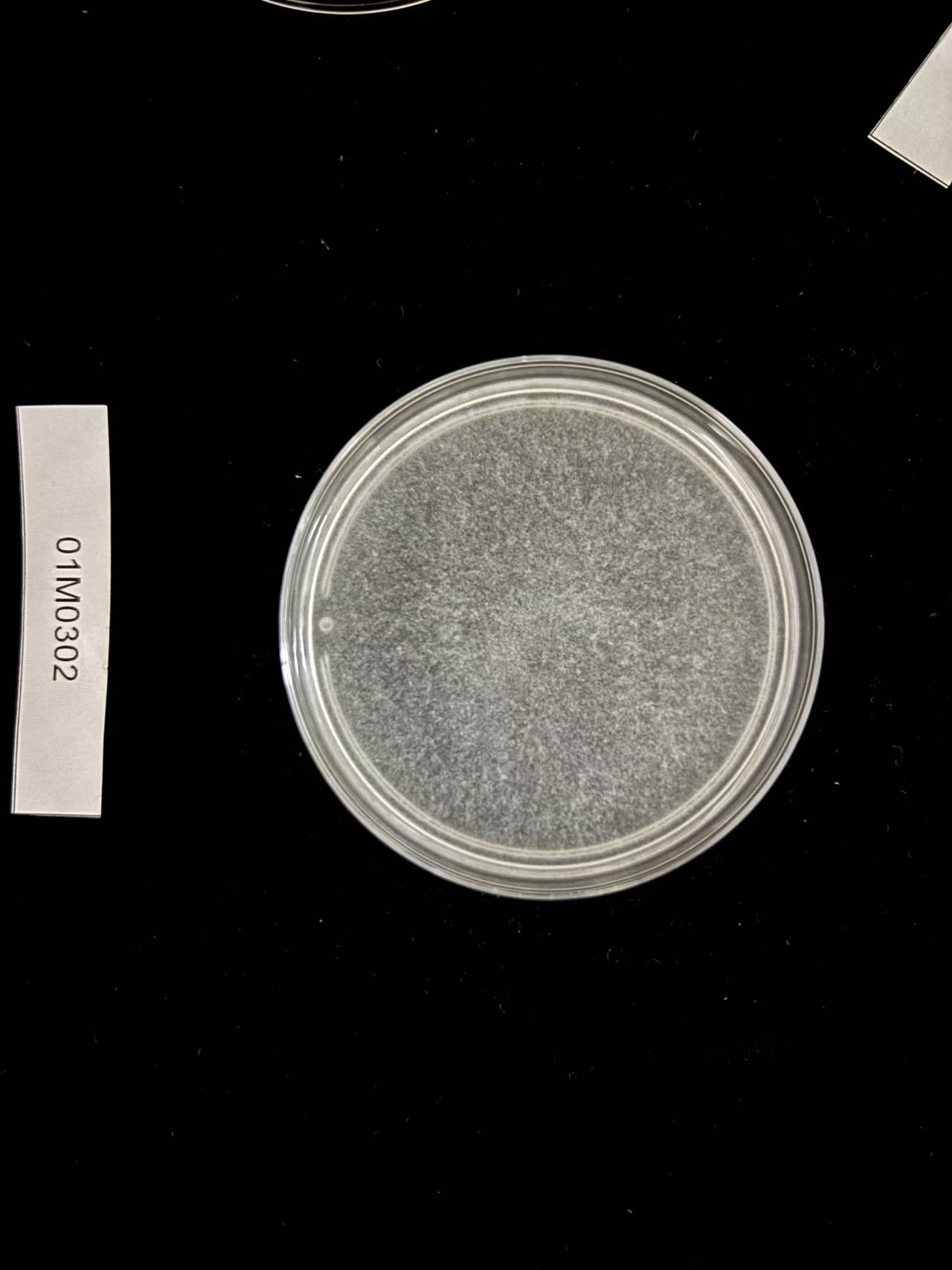

Supplement: Supplementary file 10 — Appendix Figure S1 Source Data [file 44319_2026_748_MOESM10_ESM.zip › Appendix Figure S1/S1D/gsf2IE_Repeat2.jpg]

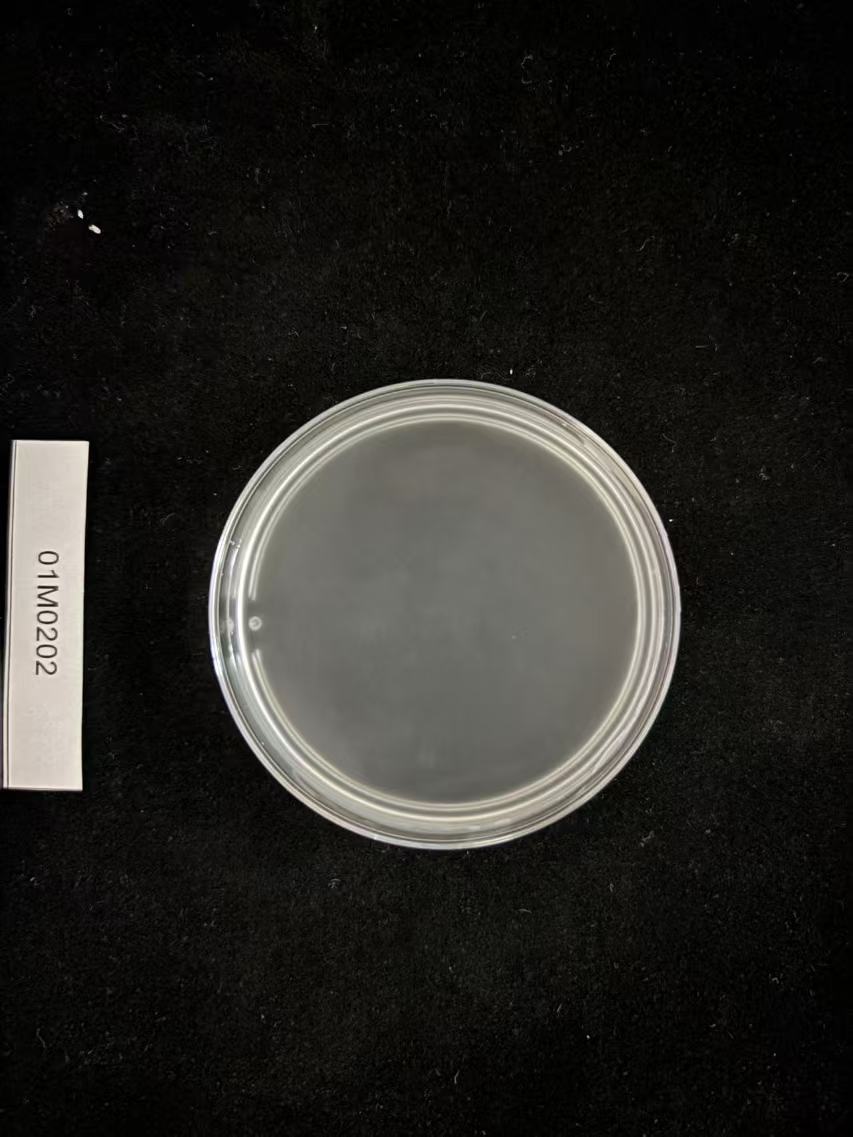

Supplement: Supplementary file 10 — Appendix Figure S1 Source Data [file 44319_2026_748_MOESM10_ESM.zip › Appendix Figure S1/S1D/gsf2Γêå_Repeat2.jpg]

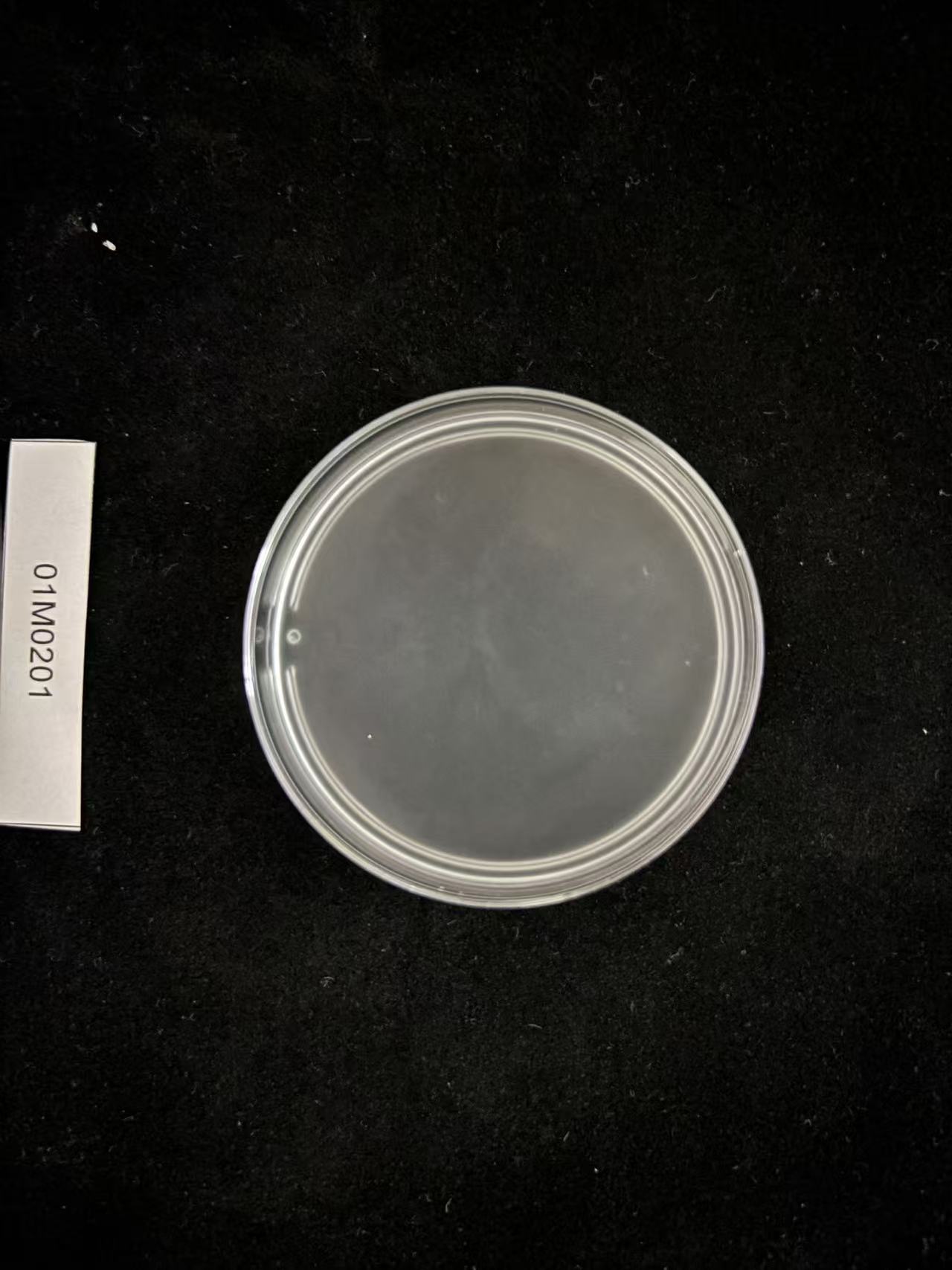

Supplement: Supplementary file 10 — Appendix Figure S1 Source Data [file 44319_2026_748_MOESM10_ESM.zip › Appendix Figure S1/S1D/gsf2Γêå_Repeat3.jpg]

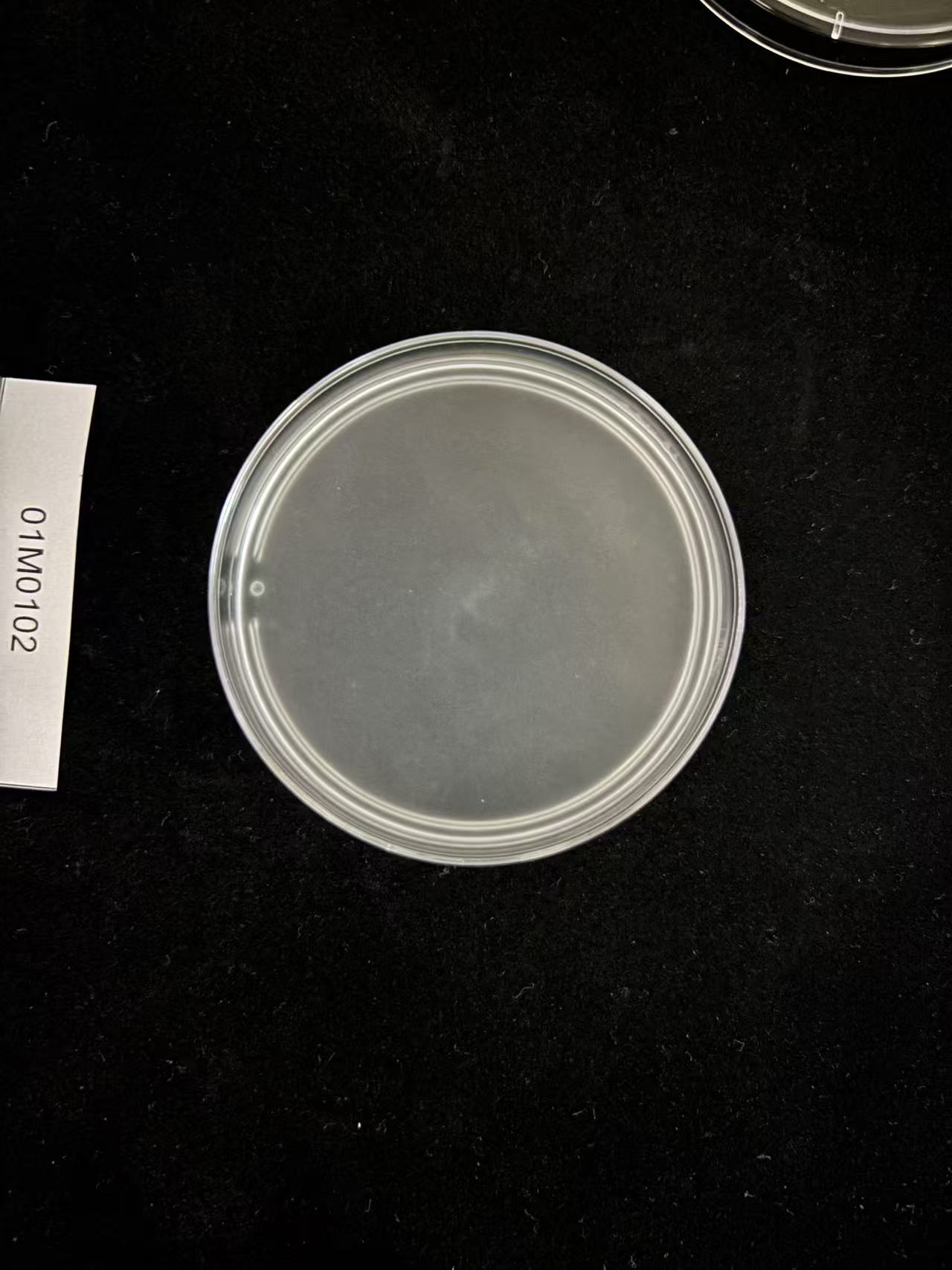

Supplement: Supplementary file 10 — Appendix Figure S1 Source Data [file 44319_2026_748_MOESM10_ESM.zip › Appendix Figure S1/S1D/WT_Repeat2.jpg]

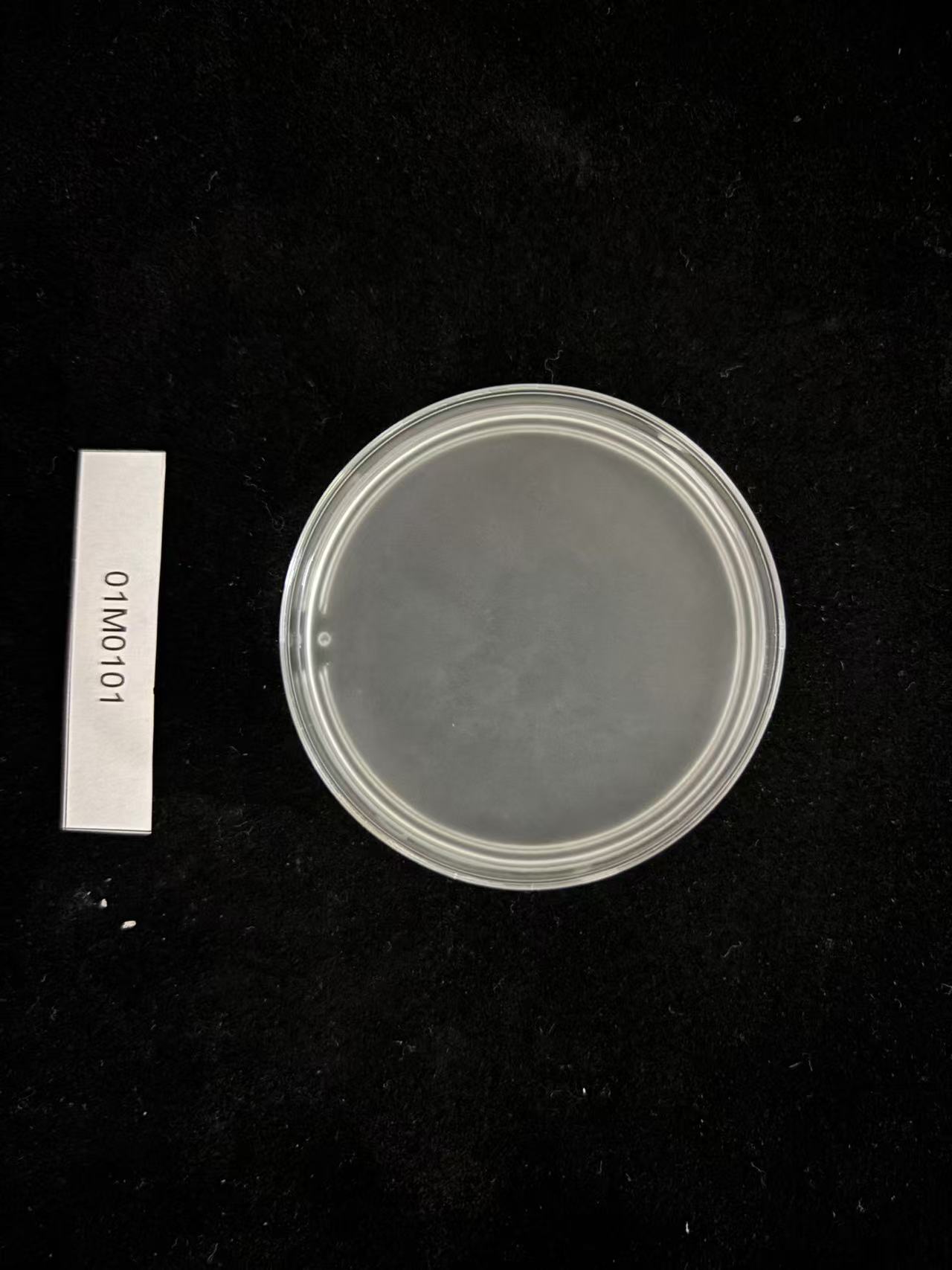

Supplement: Supplementary file 10 — Appendix Figure S1 Source Data [file 44319_2026_748_MOESM10_ESM.zip › Appendix Figure S1/S1D/WT_Repeat3.jpg]

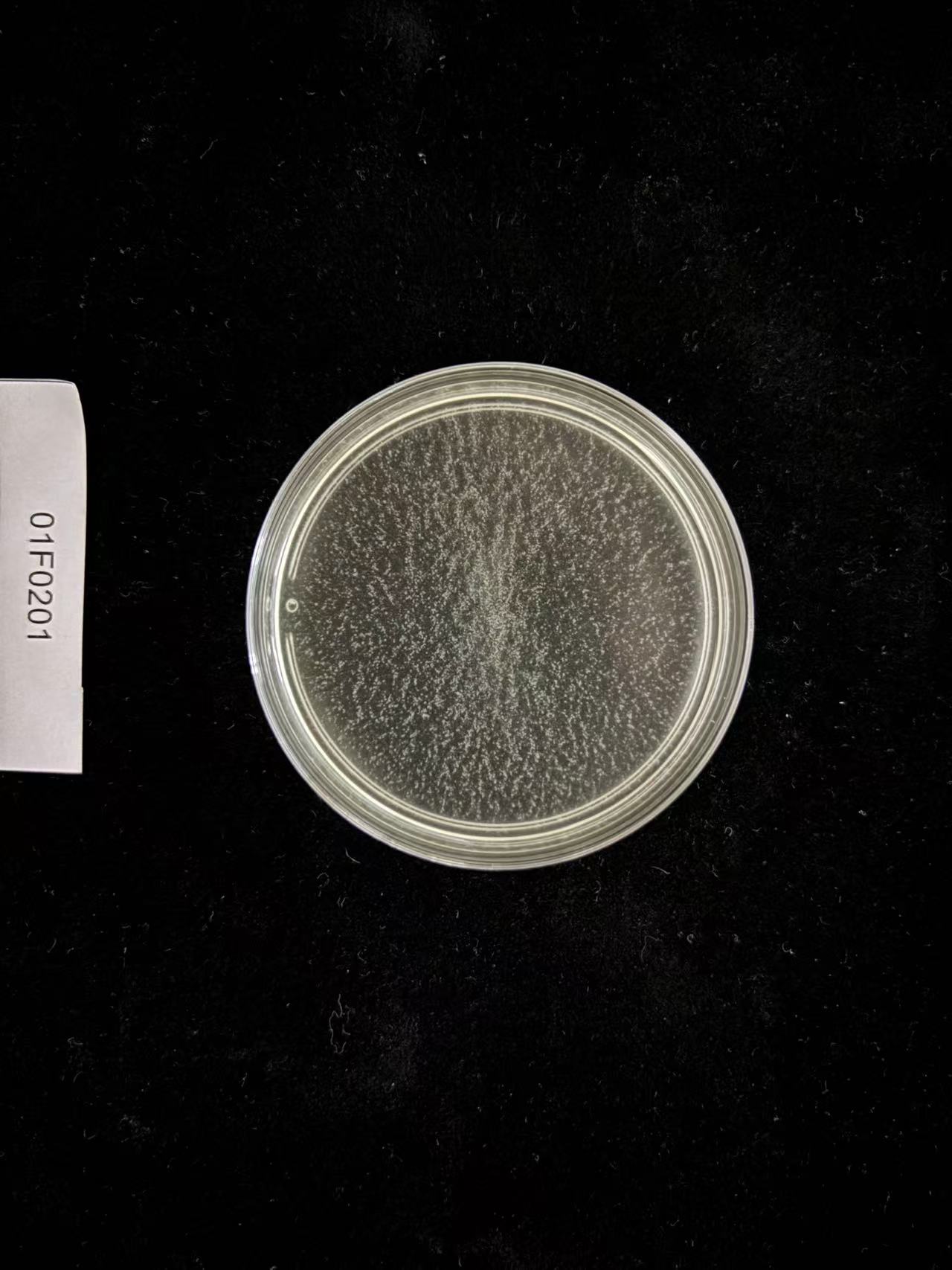

Supplement: Supplementary file 10 — Appendix Figure S1 Source Data [file 44319_2026_748_MOESM10_ESM.zip › Appendix Figure S1/S1C/Treated_Repeat3.jpg]

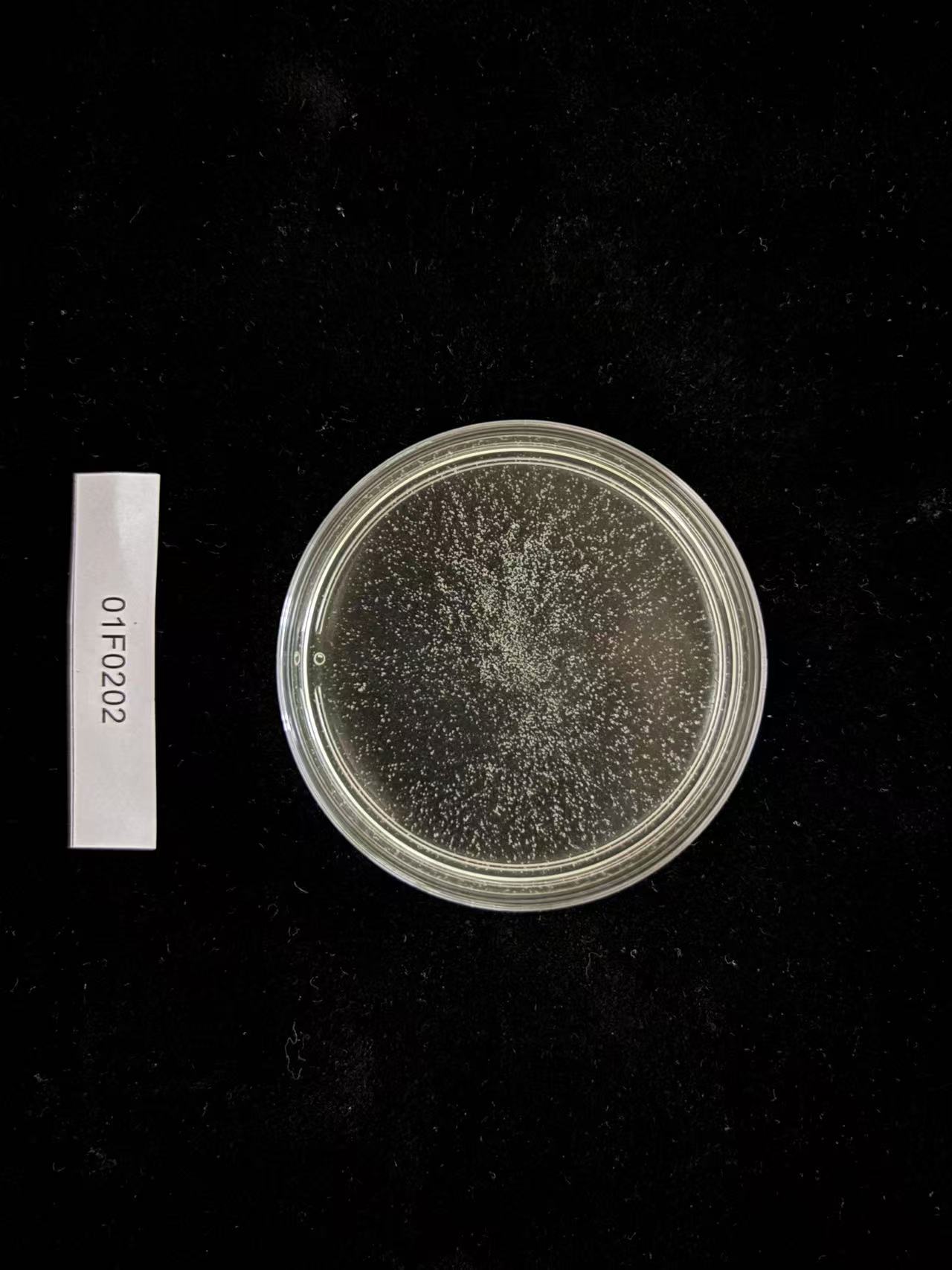

Supplement: Supplementary file 10 — Appendix Figure S1 Source Data [file 44319_2026_748_MOESM10_ESM.zip › Appendix Figure S1/S1C/Treated_Repeat2.jpg]

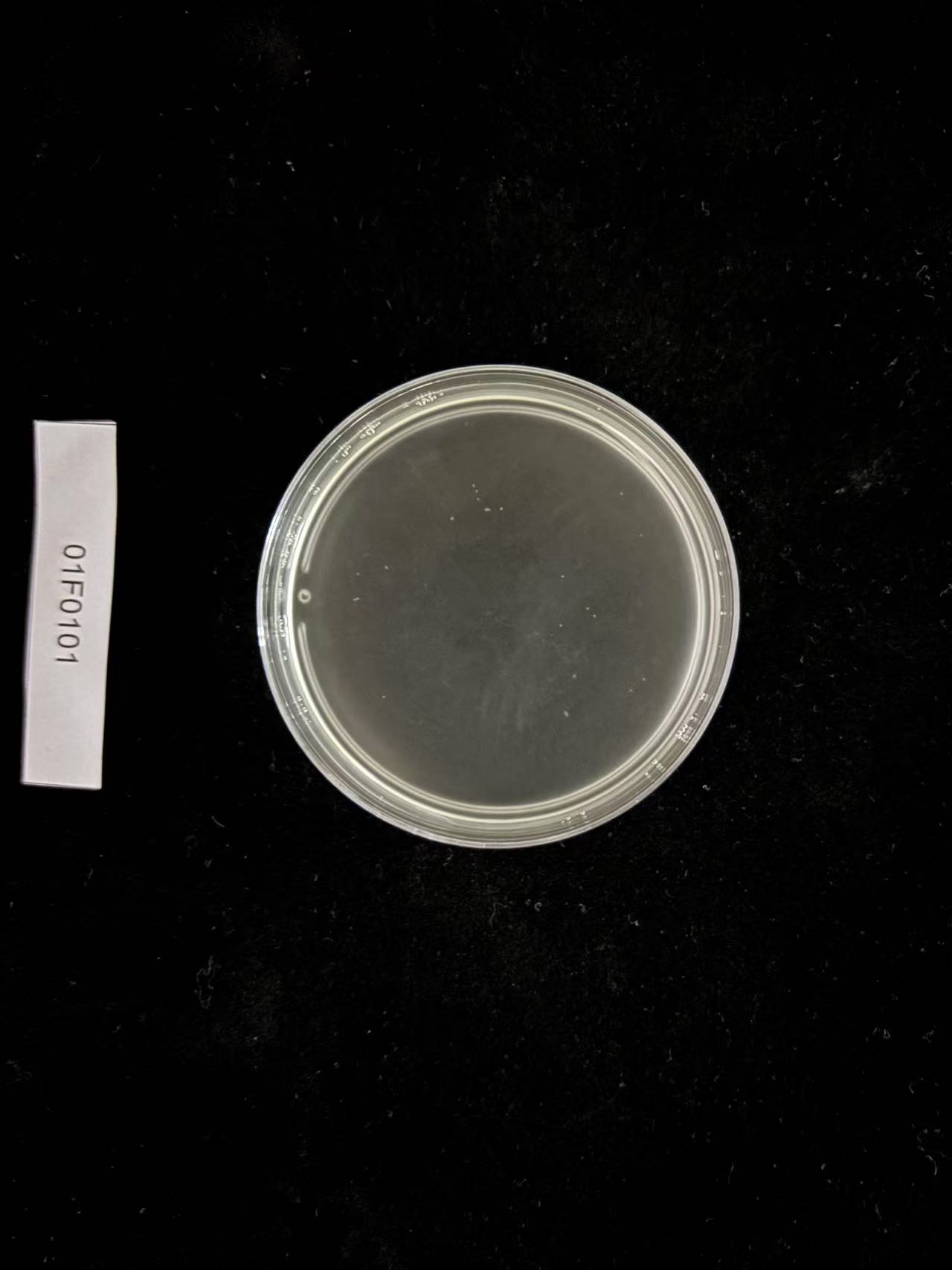

Supplement: Supplementary file 10 — Appendix Figure S1 Source Data [file 44319_2026_748_MOESM10_ESM.zip › Appendix Figure S1/S1C/Control_Repeat3.jpg]

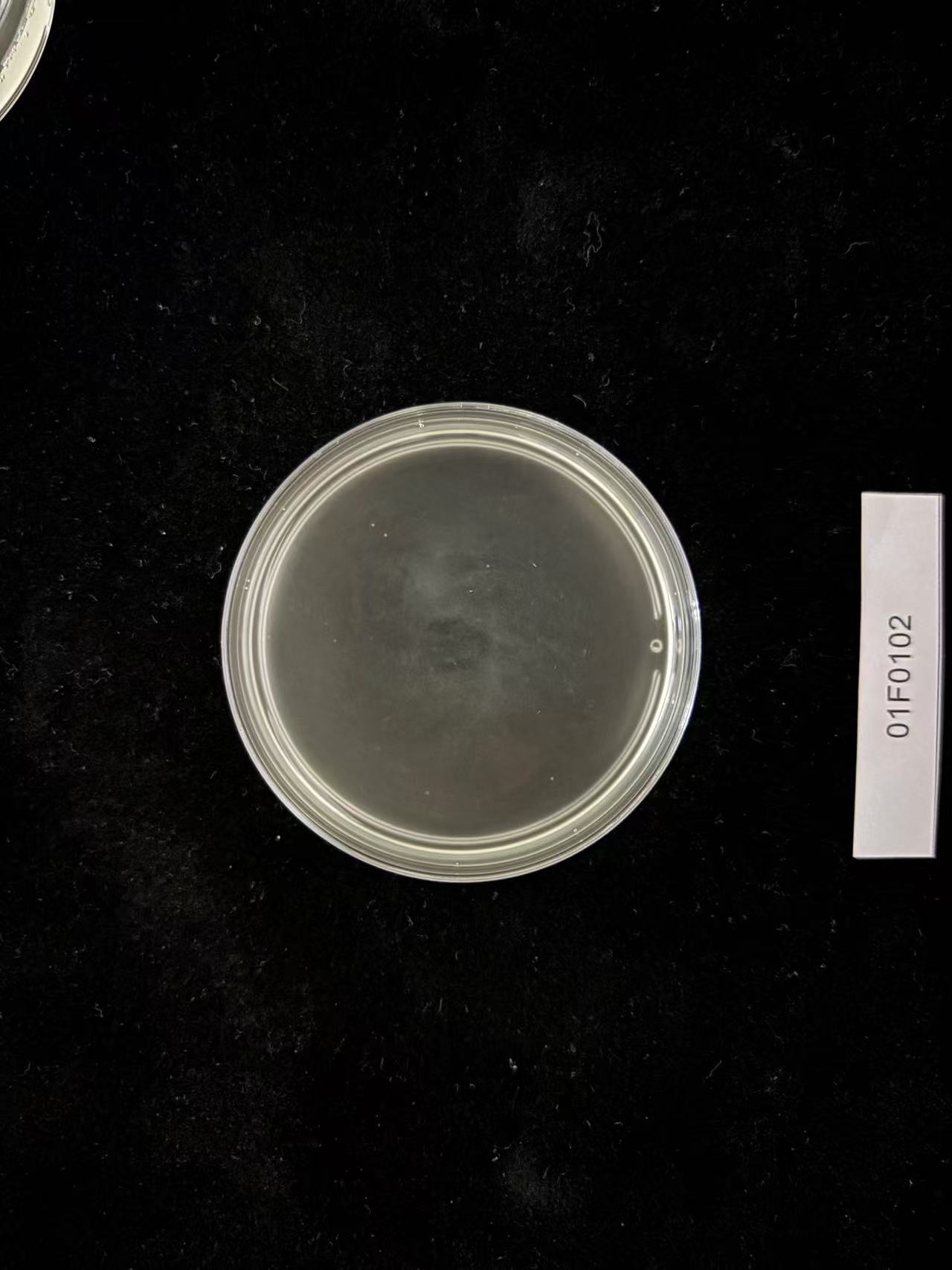

Supplement: Supplementary file 10 — Appendix Figure S1 Source Data [file 44319_2026_748_MOESM10_ESM.zip › Appendix Figure S1/S1C/Control_Repeat2.jpg]

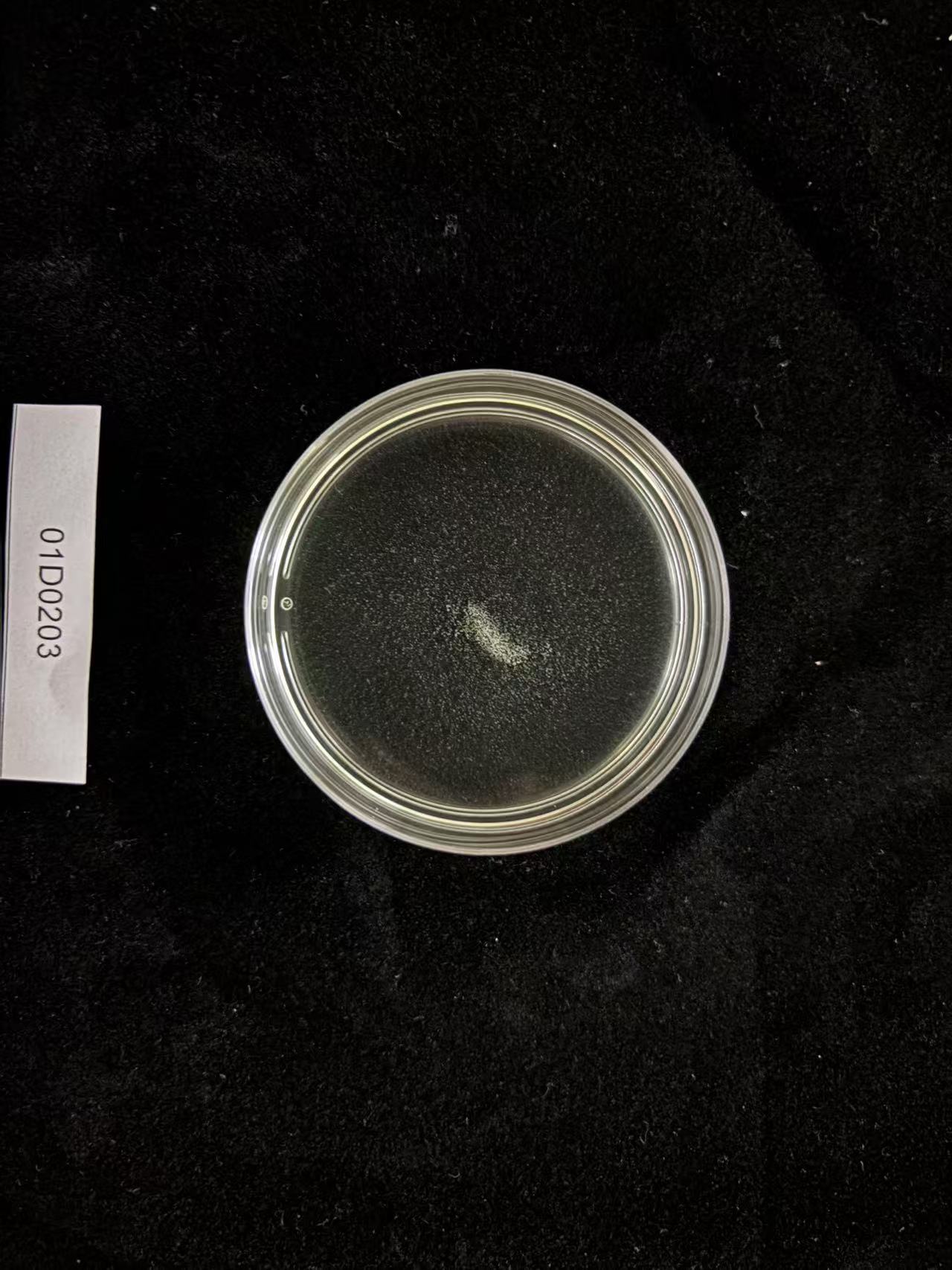

Supplement: Supplementary file 10 — Appendix Figure S1 Source Data [file 44319_2026_748_MOESM10_ESM.zip › Appendix Figure S1/S1B/Treated_Repeat3.jpg]

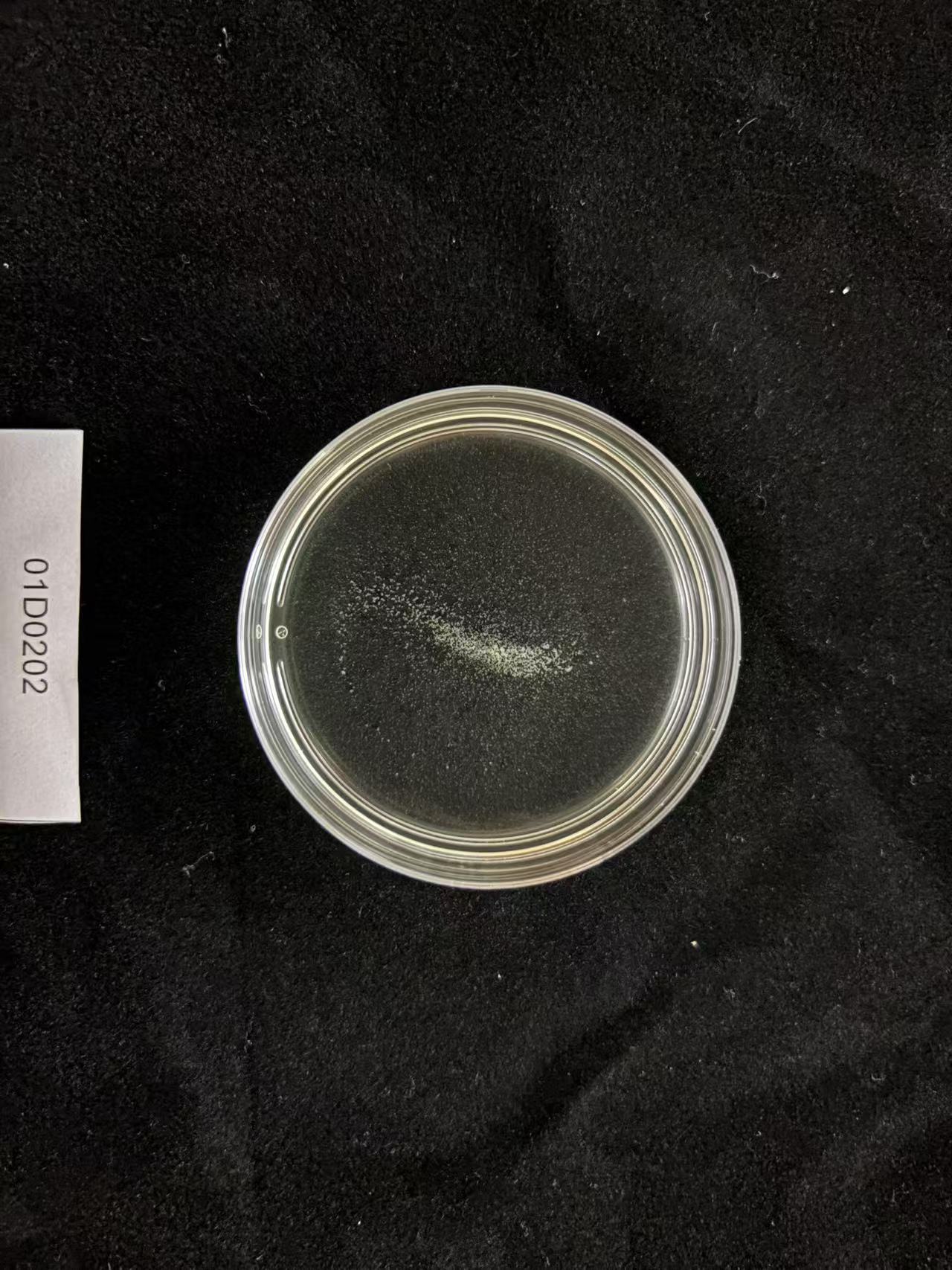

Supplement: Supplementary file 10 — Appendix Figure S1 Source Data [file 44319_2026_748_MOESM10_ESM.zip › Appendix Figure S1/S1B/Treated_Repeat2.jpg]

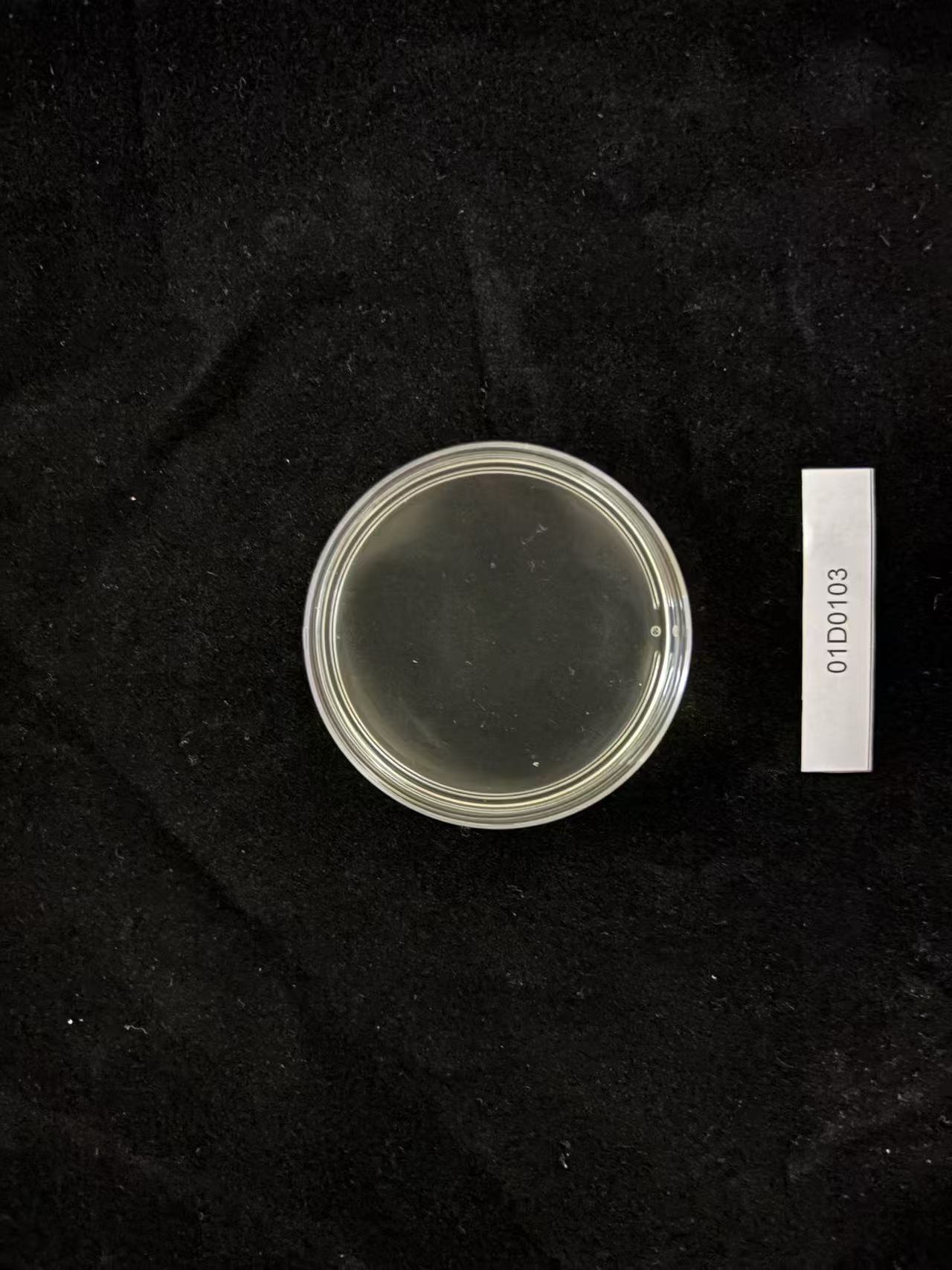

Supplement: Supplementary file 10 — Appendix Figure S1 Source Data [file 44319_2026_748_MOESM10_ESM.zip › Appendix Figure S1/S1B/Control_Repeat3.jpg]

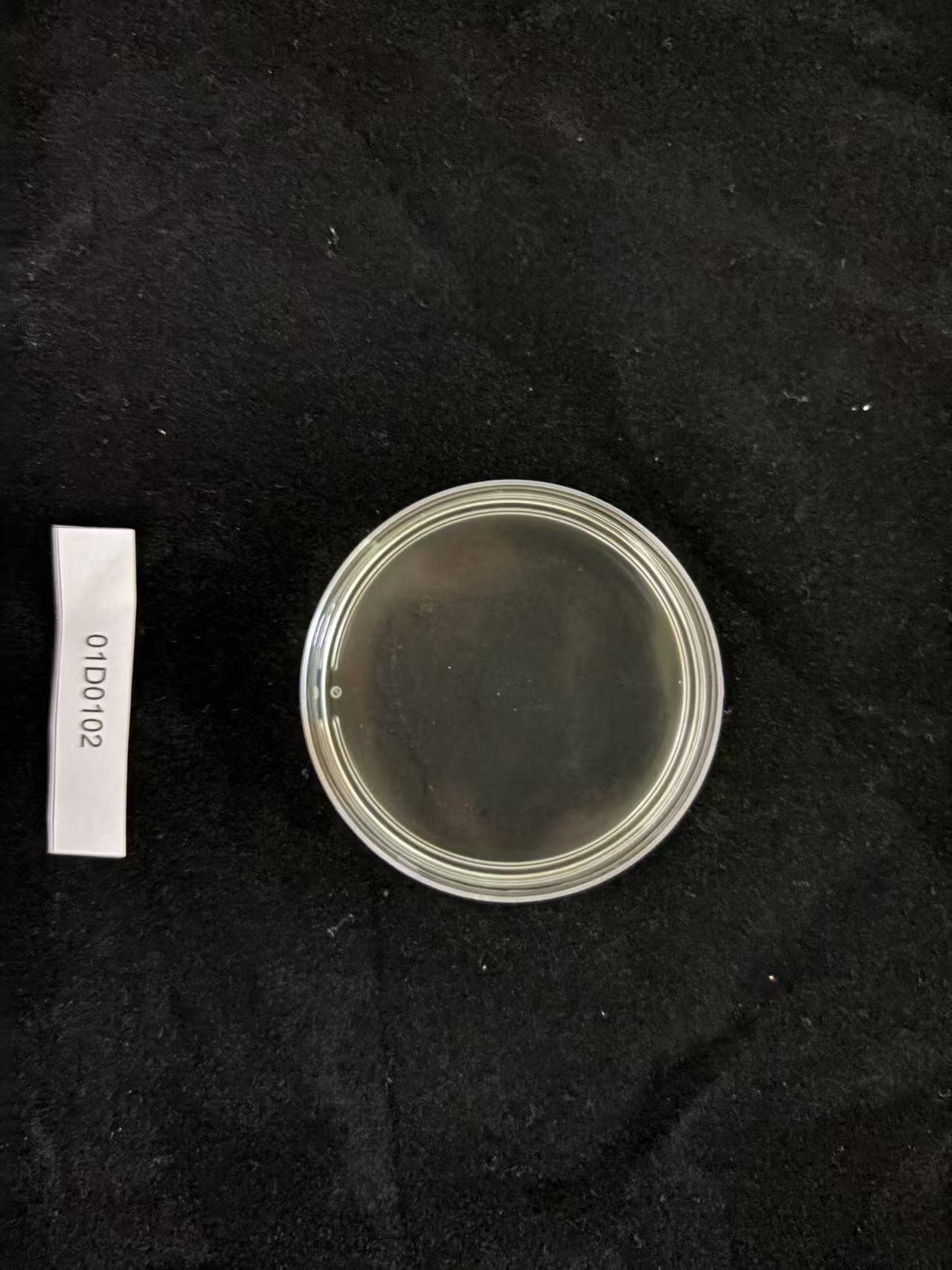

Supplement: Supplementary file 10 — Appendix Figure S1 Source Data [file 44319_2026_748_MOESM10_ESM.zip › Appendix Figure S1/S1B/Control_Repeat2.jpg]

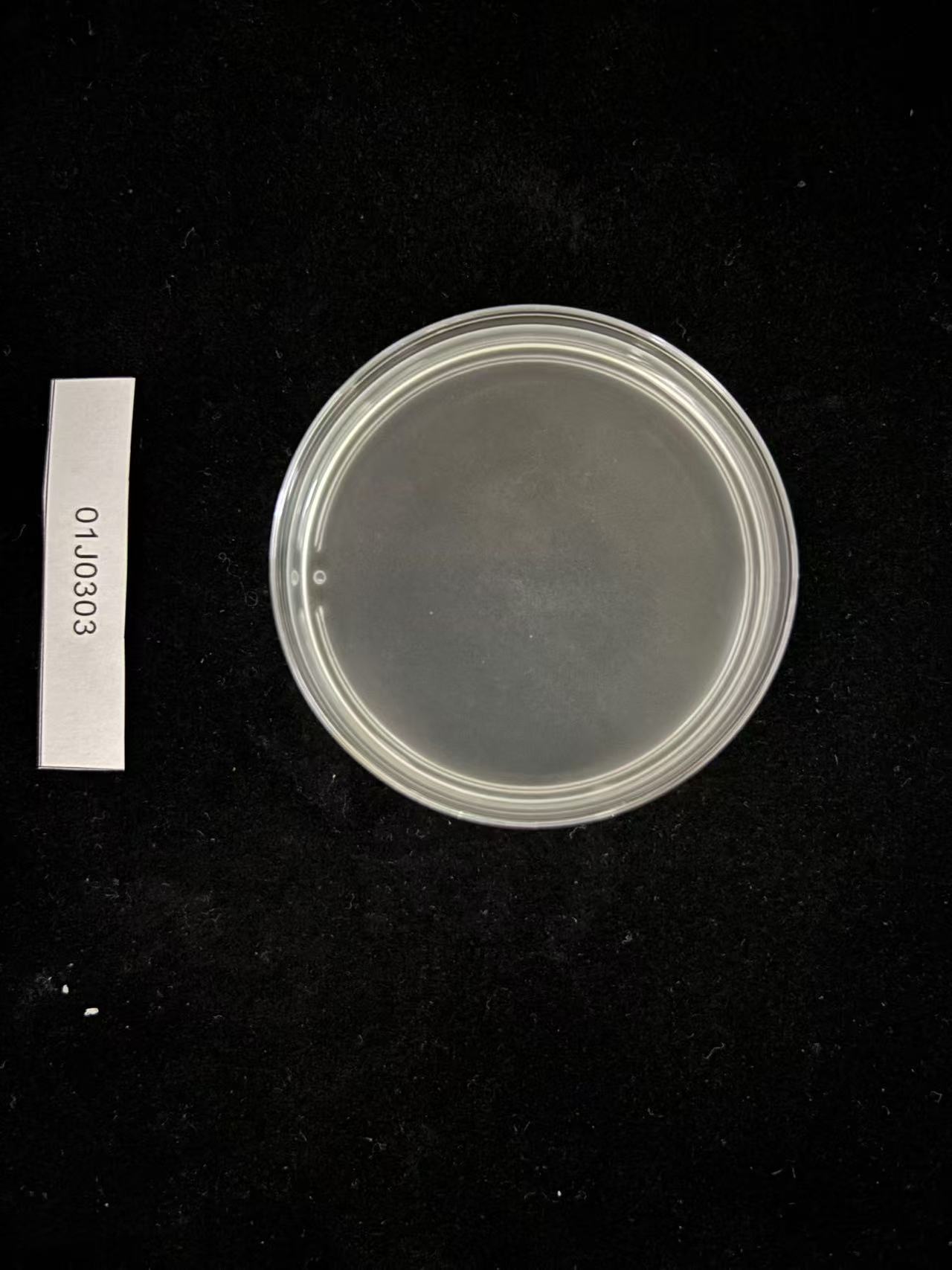

Supplement: Supplementary file 10 — Appendix Figure S1 Source Data [file 44319_2026_748_MOESM10_ESM.zip › Appendix Figure S1/S1E/gsf2IE_Repeat3.jpg]

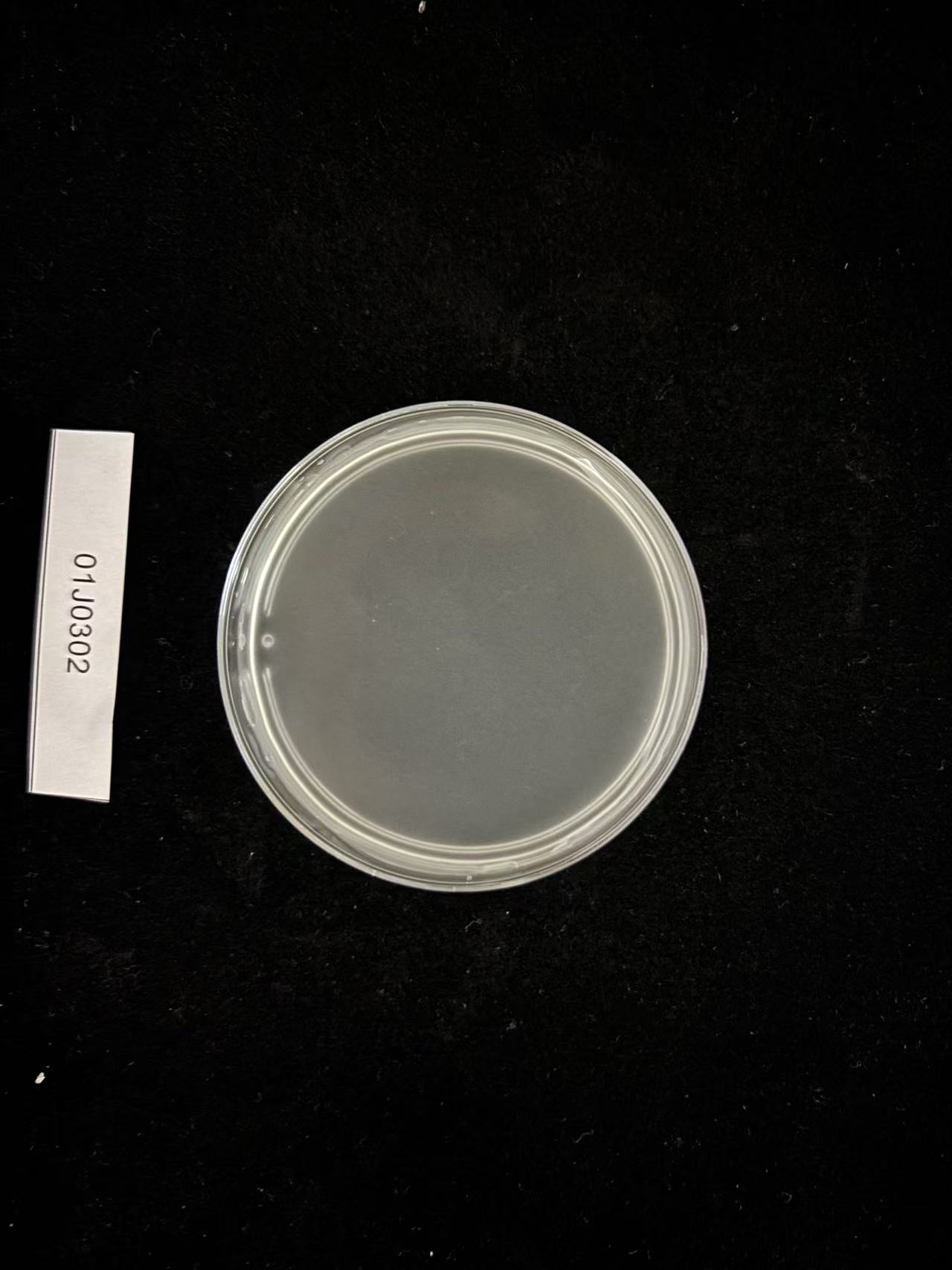

Supplement: Supplementary file 10 — Appendix Figure S1 Source Data [file 44319_2026_748_MOESM10_ESM.zip › Appendix Figure S1/S1E/gsf2IE_Repeat2.jpg]

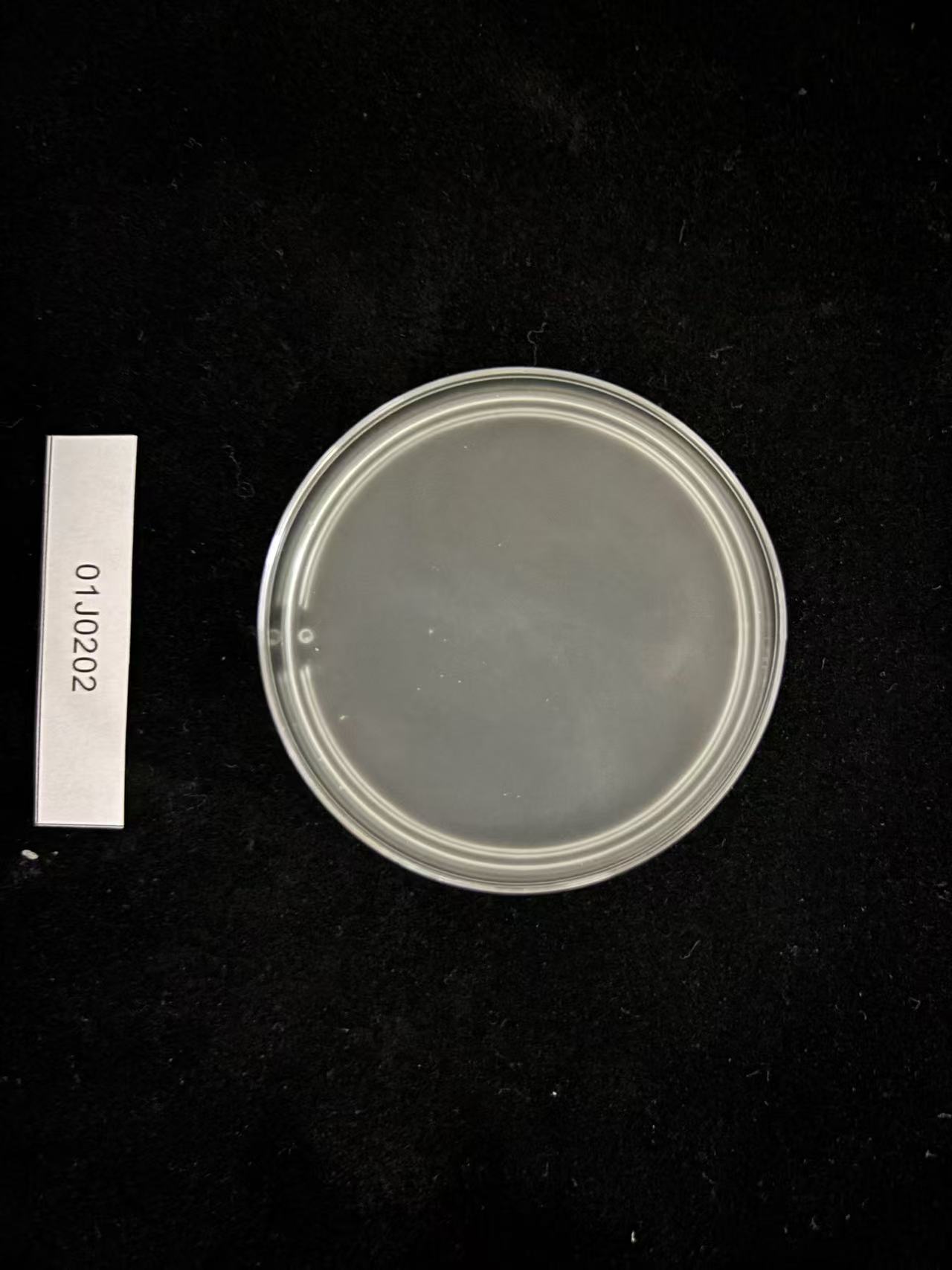

Supplement: Supplementary file 10 — Appendix Figure S1 Source Data [file 44319_2026_748_MOESM10_ESM.zip › Appendix Figure S1/S1E/gsf2Γêå_Repeat2.jpg]

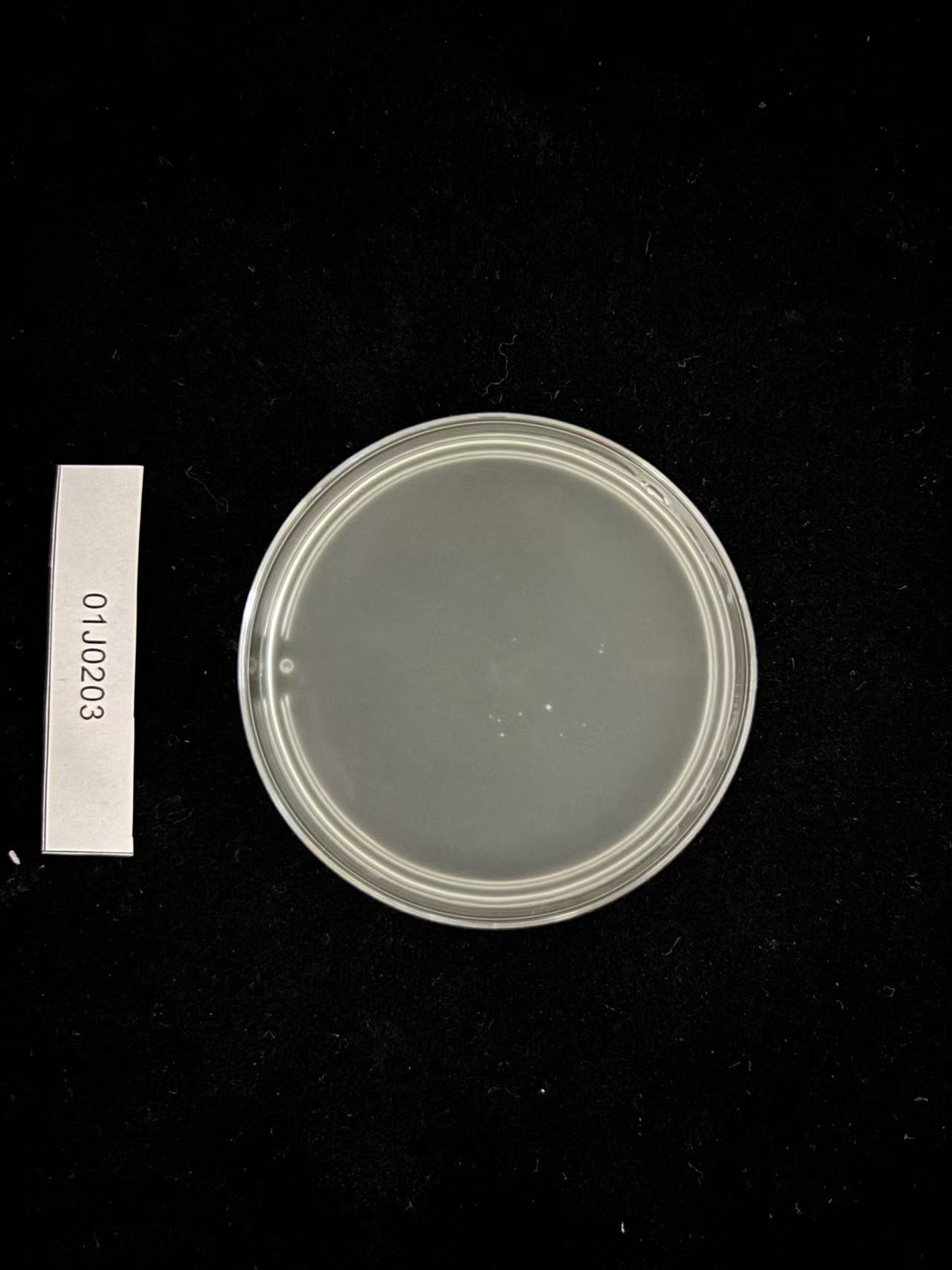

Supplement: Supplementary file 10 — Appendix Figure S1 Source Data [file 44319_2026_748_MOESM10_ESM.zip › Appendix Figure S1/S1E/gsf2Γêå_Repeat3.jpg]

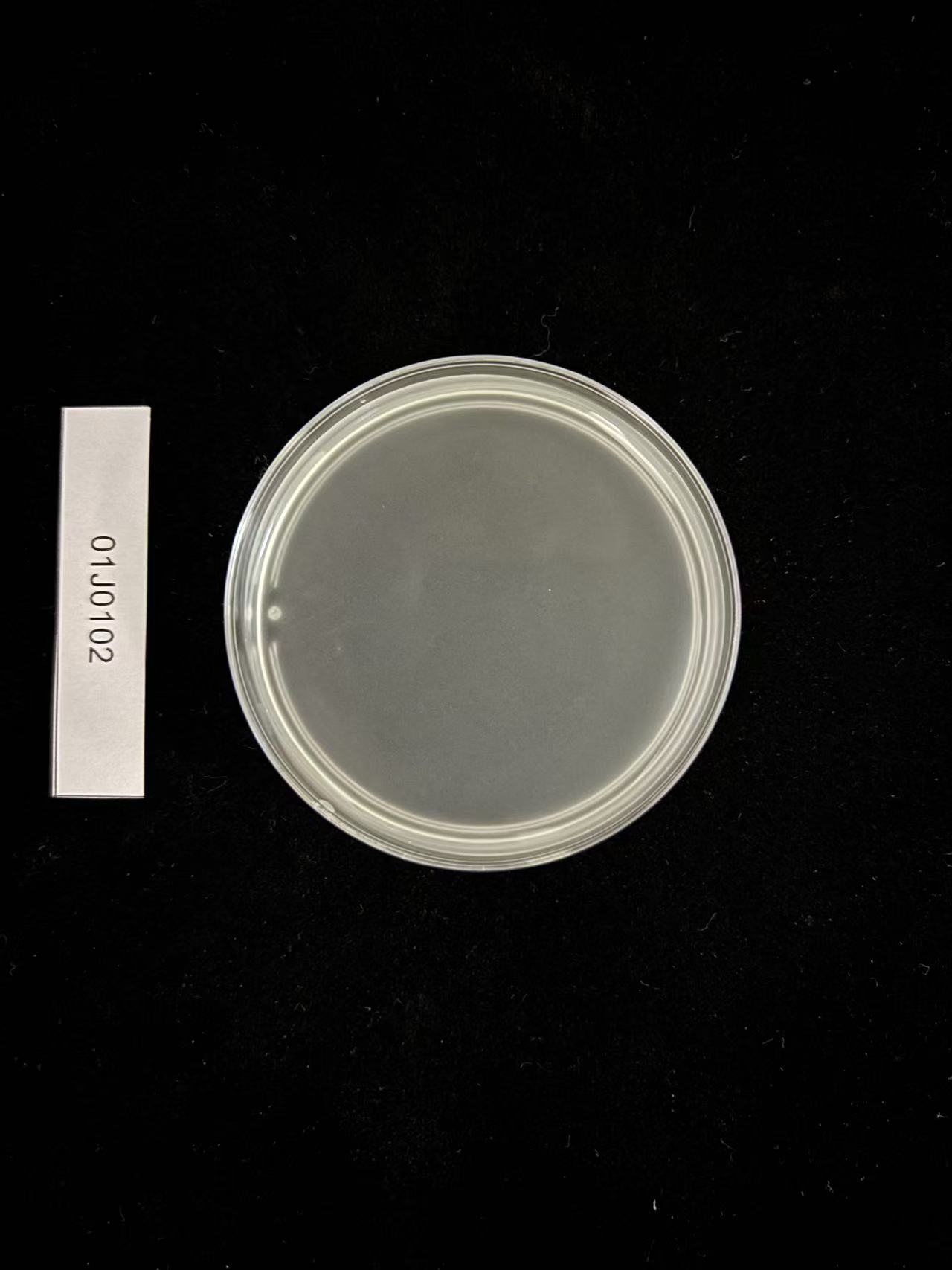

Supplement: Supplementary file 10 — Appendix Figure S1 Source Data [file 44319_2026_748_MOESM10_ESM.zip › Appendix Figure S1/S1E/WT_Repeat2.jpg]

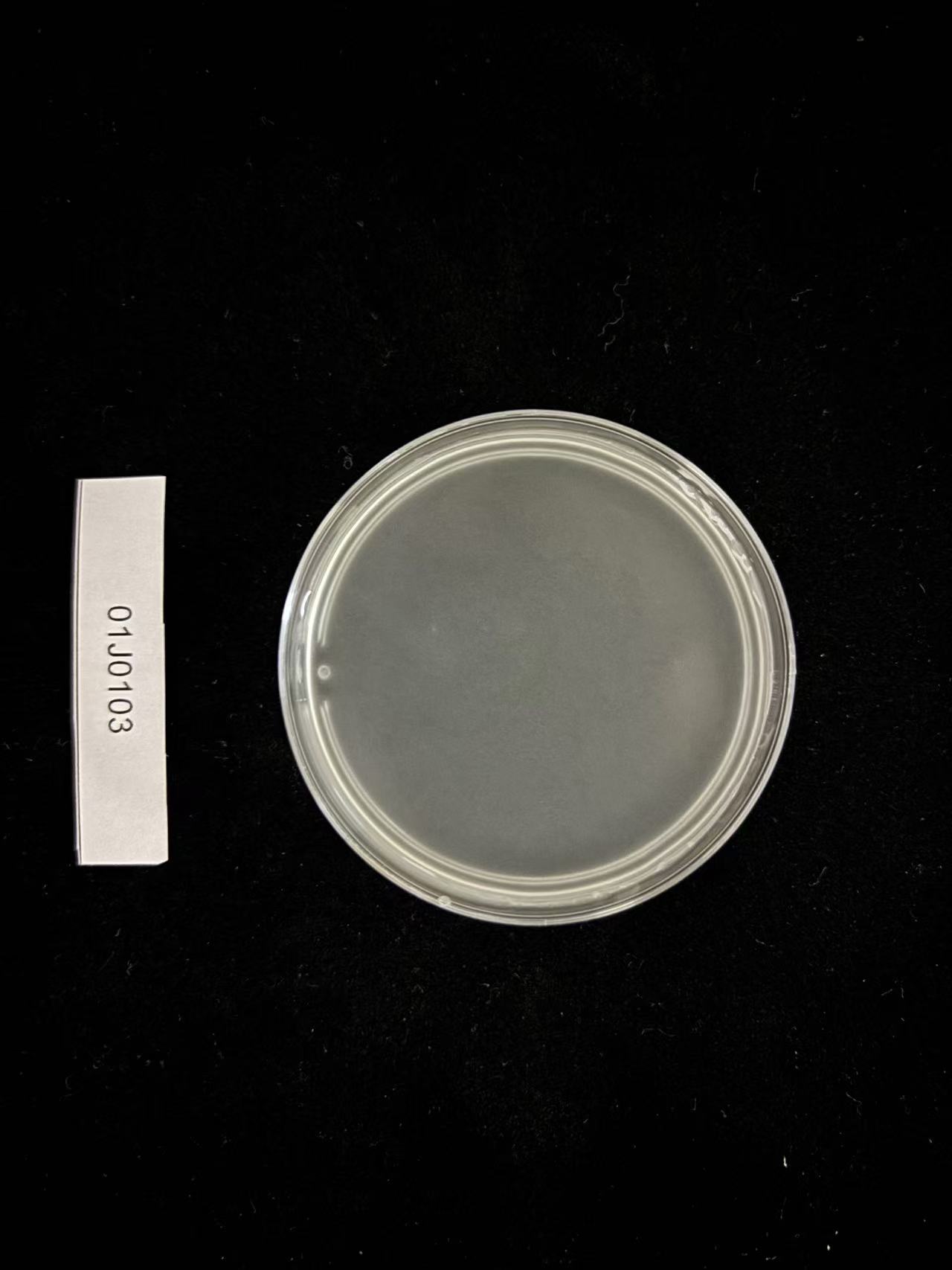

Supplement: Supplementary file 10 — Appendix Figure S1 Source Data [file 44319_2026_748_MOESM10_ESM.zip › Appendix Figure S1/S1E/WT_Repeat3.jpg]

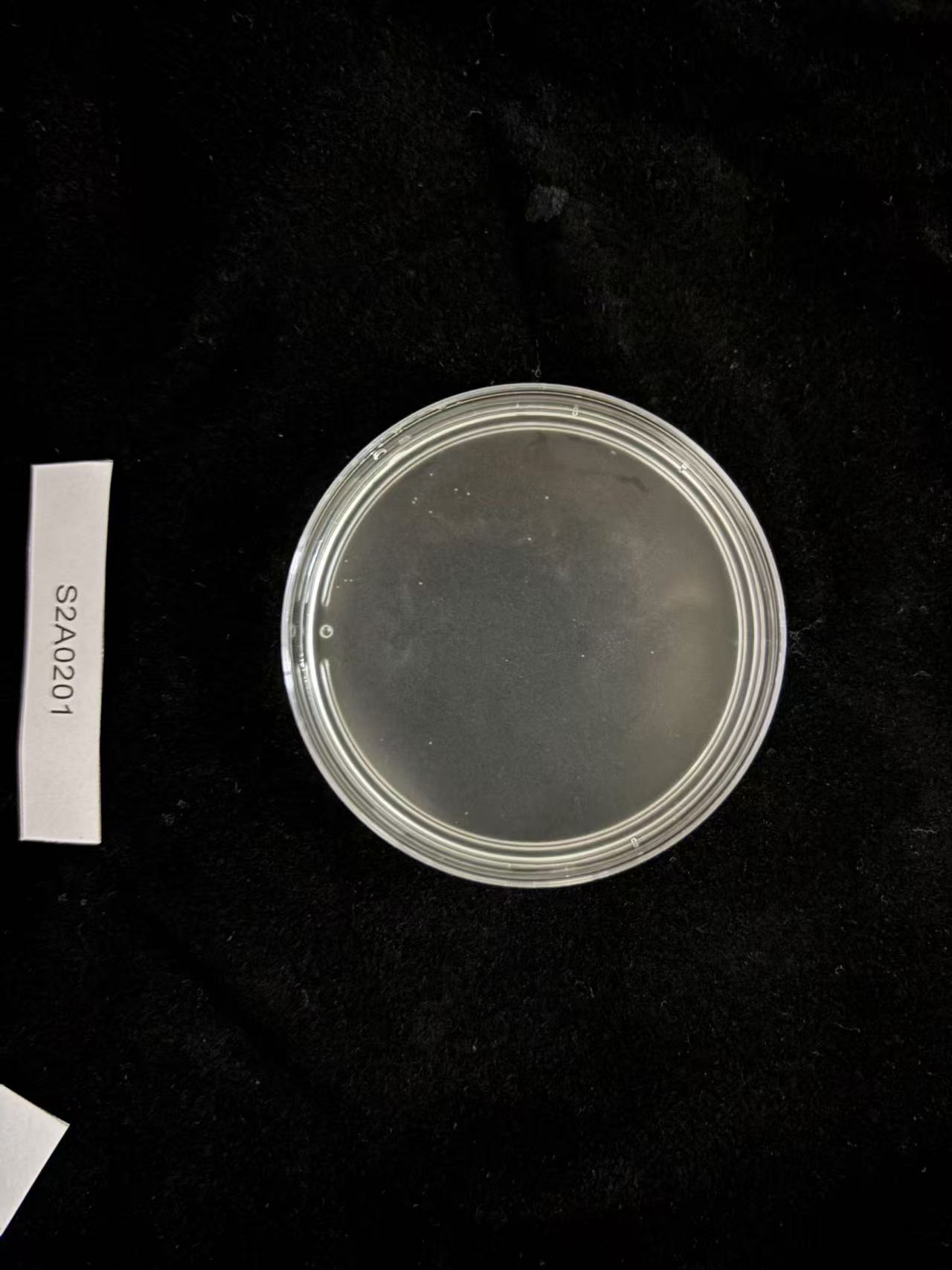

Supplement: Supplementary file 11 — Appendix Figure S2 Source Data [file 44319_2026_748_MOESM11_ESM.zip › Appendix Figure S2/S2A/hydrogen peroxide_repeat1.jpg]

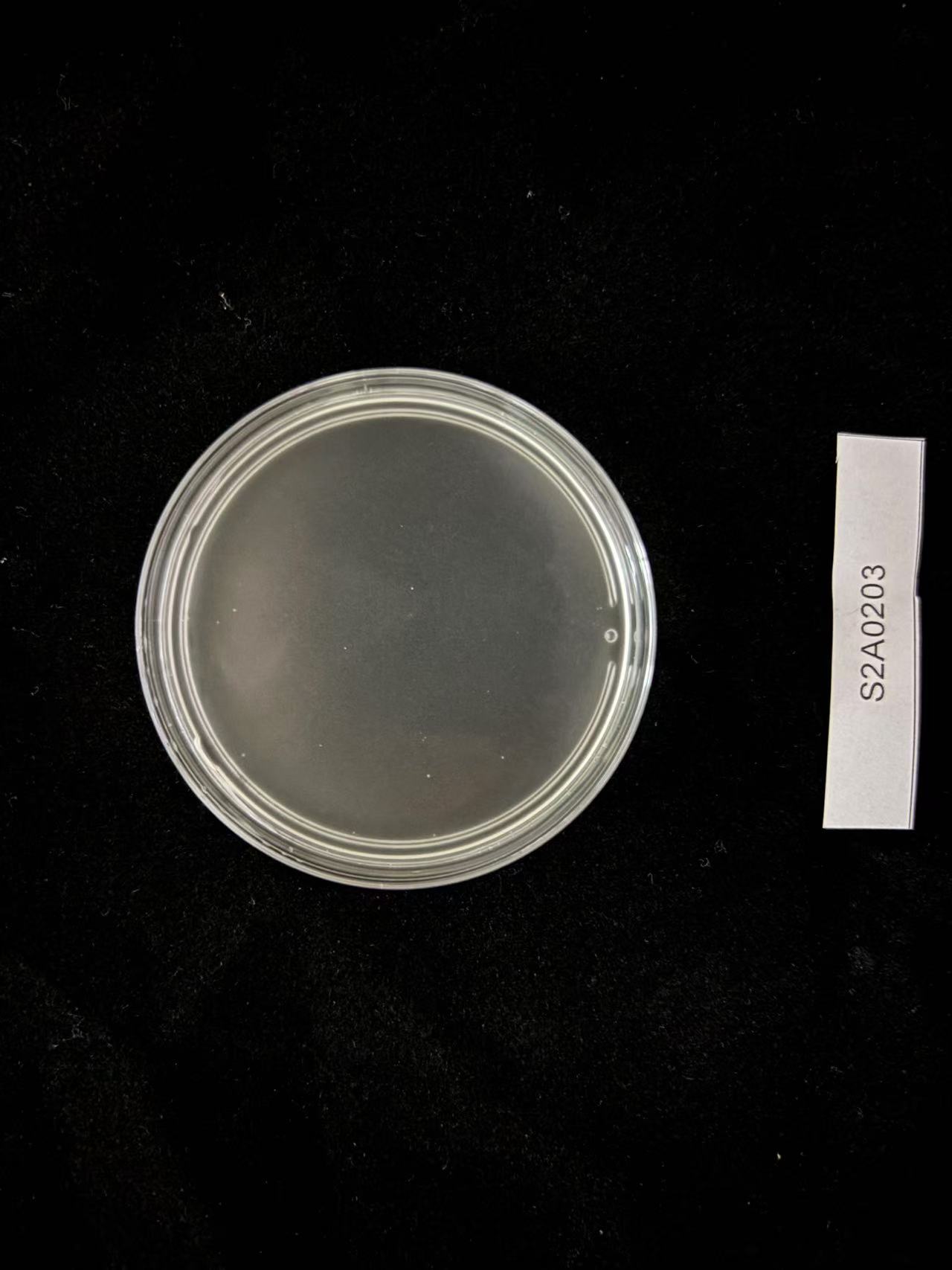

Supplement: Supplementary file 11 — Appendix Figure S2 Source Data [file 44319_2026_748_MOESM11_ESM.zip › Appendix Figure S2/S2A/hydrogen peroxide_repeat3.jpg]

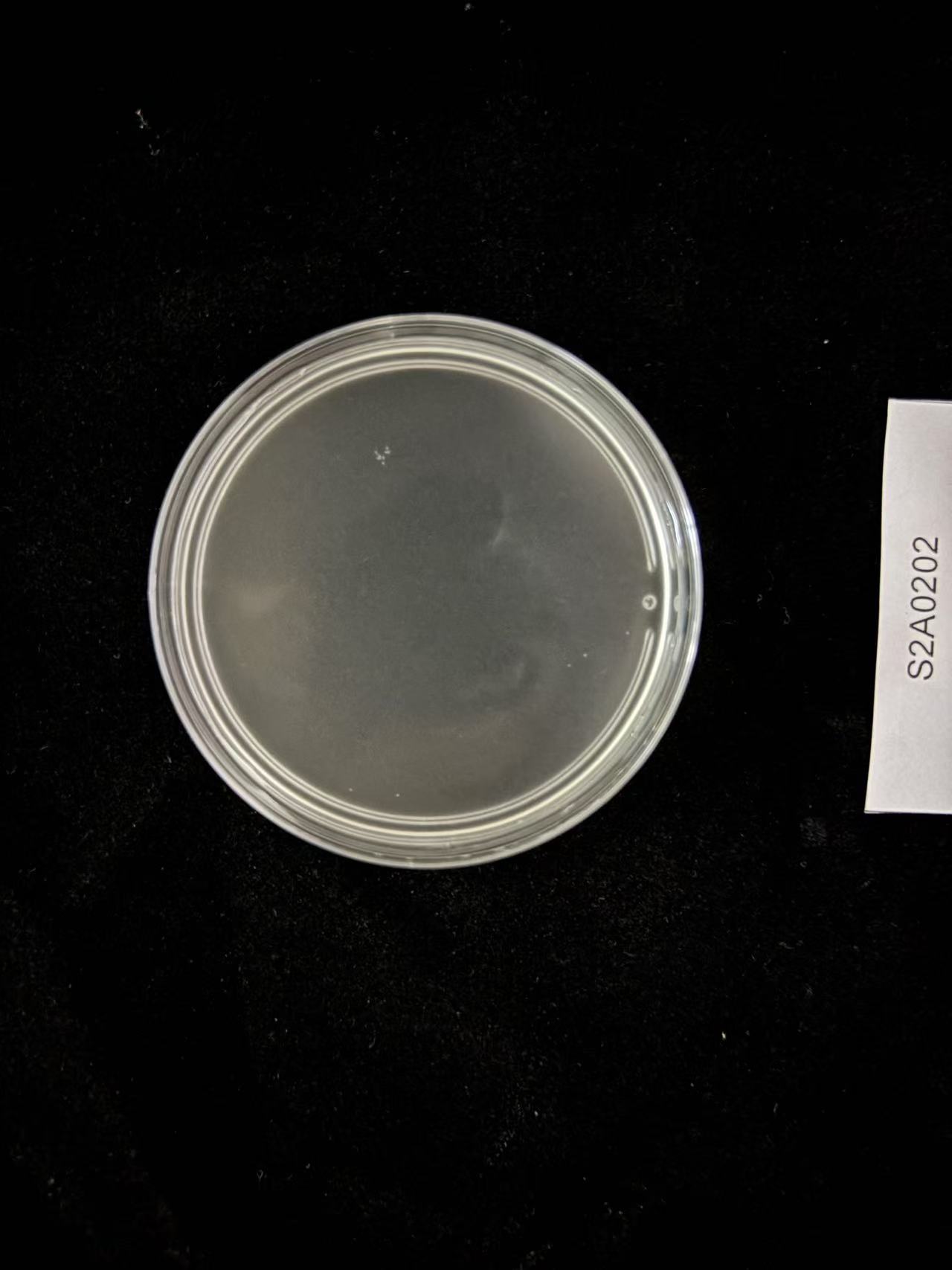

Supplement: Supplementary file 11 — Appendix Figure S2 Source Data [file 44319_2026_748_MOESM11_ESM.zip › Appendix Figure S2/S2A/hydrogen peroxide_repeat2.jpg]

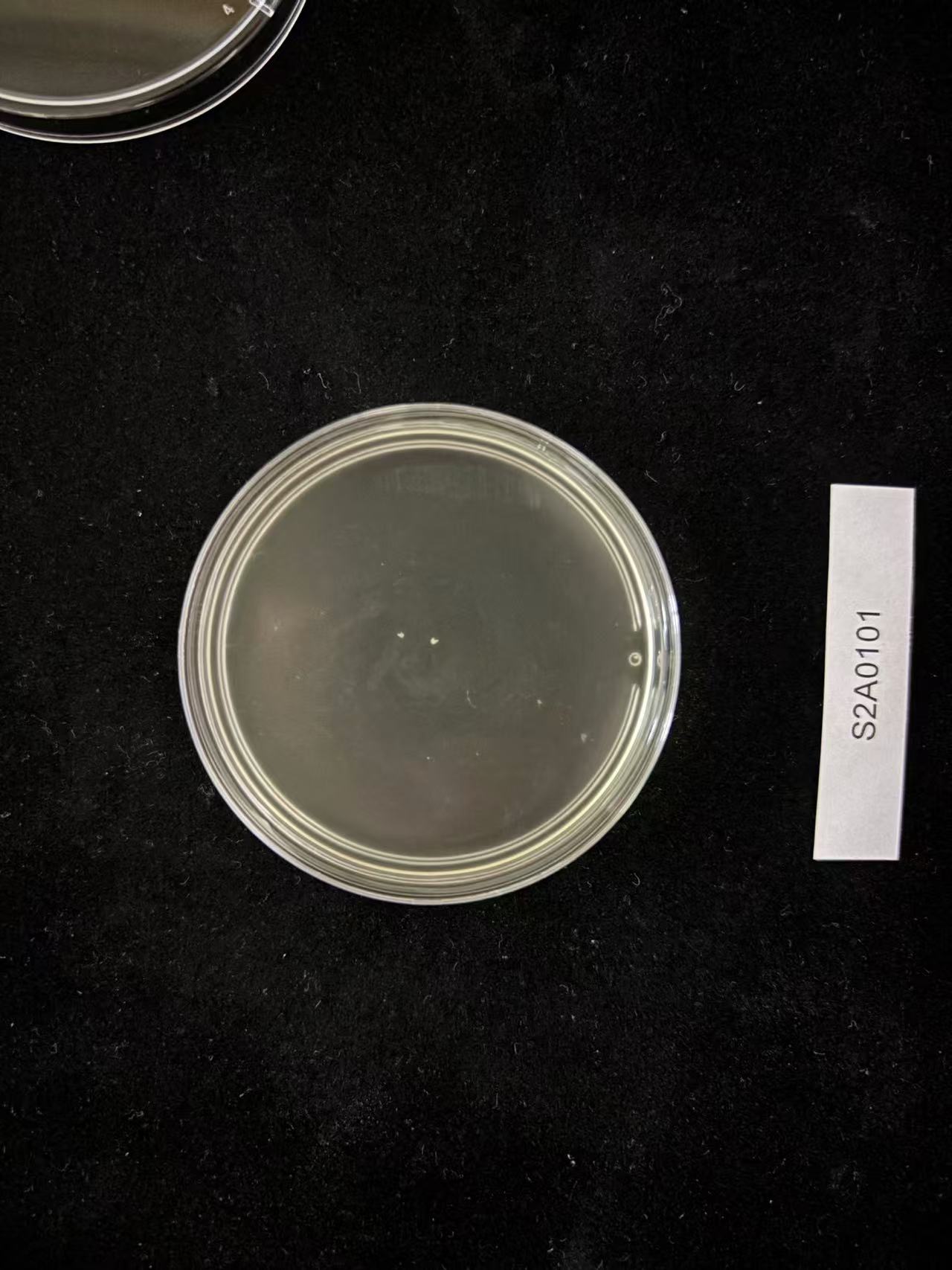

Supplement: Supplementary file 11 — Appendix Figure S2 Source Data [file 44319_2026_748_MOESM11_ESM.zip › Appendix Figure S2/S2A/Control_Repeat1.jpg]

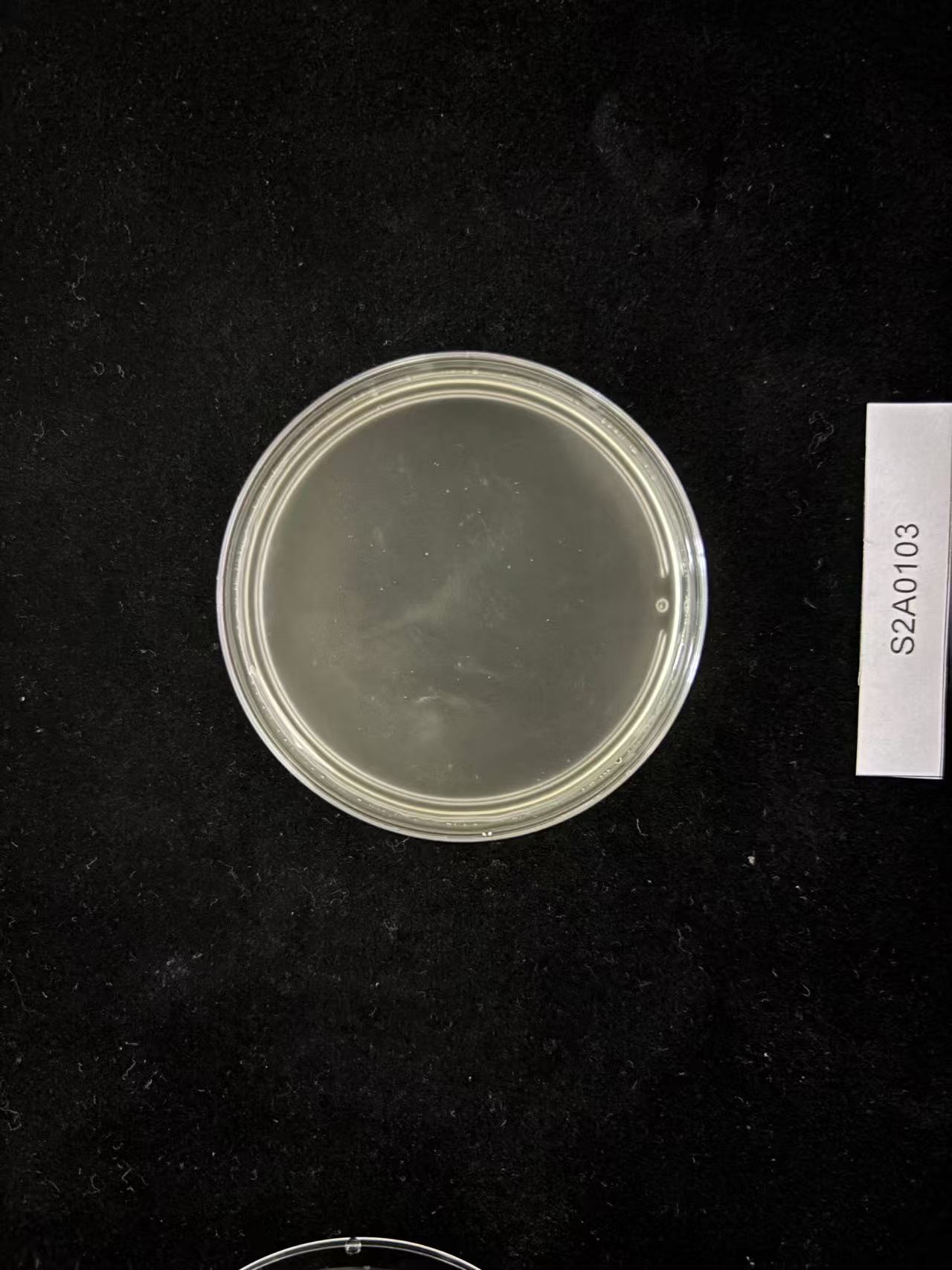

Supplement: Supplementary file 11 — Appendix Figure S2 Source Data [file 44319_2026_748_MOESM11_ESM.zip › Appendix Figure S2/S2A/Control_Repeat3.jpg]

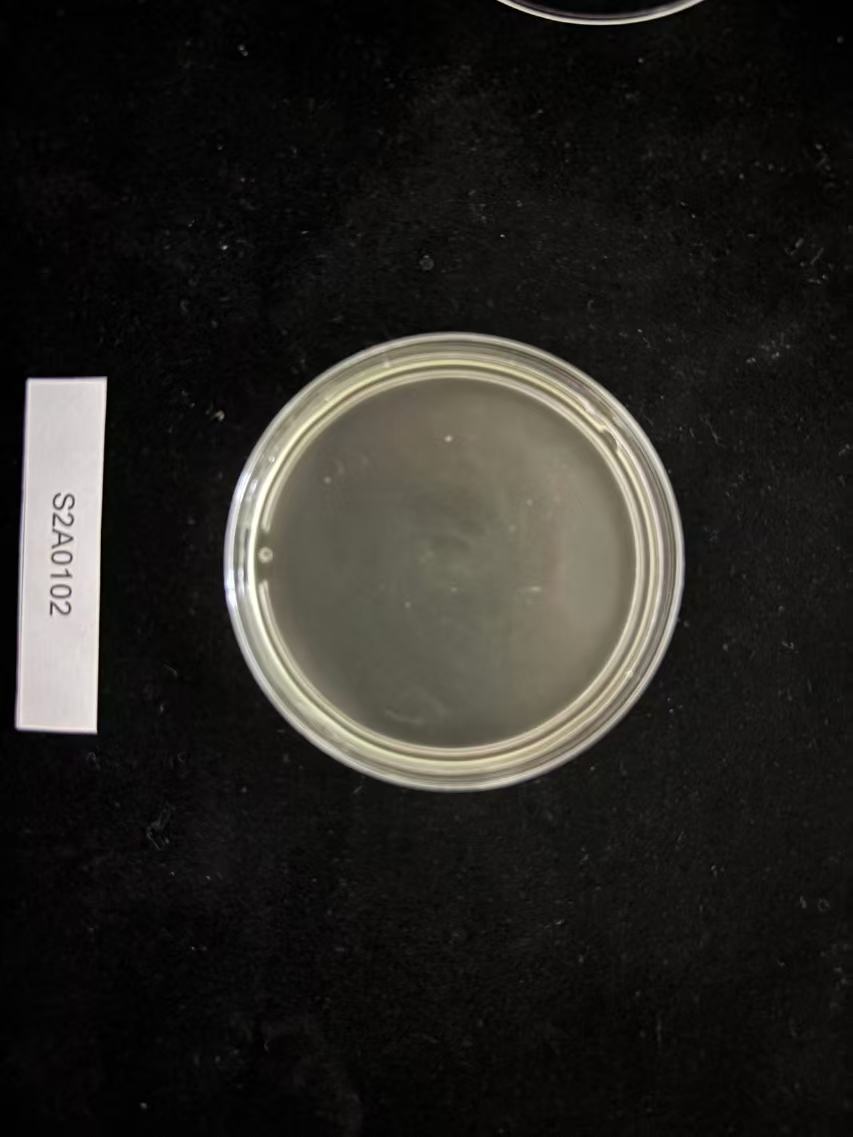

Supplement: Supplementary file 11 — Appendix Figure S2 Source Data [file 44319_2026_748_MOESM11_ESM.zip › Appendix Figure S2/S2A/Control_Repeat2.jpg]

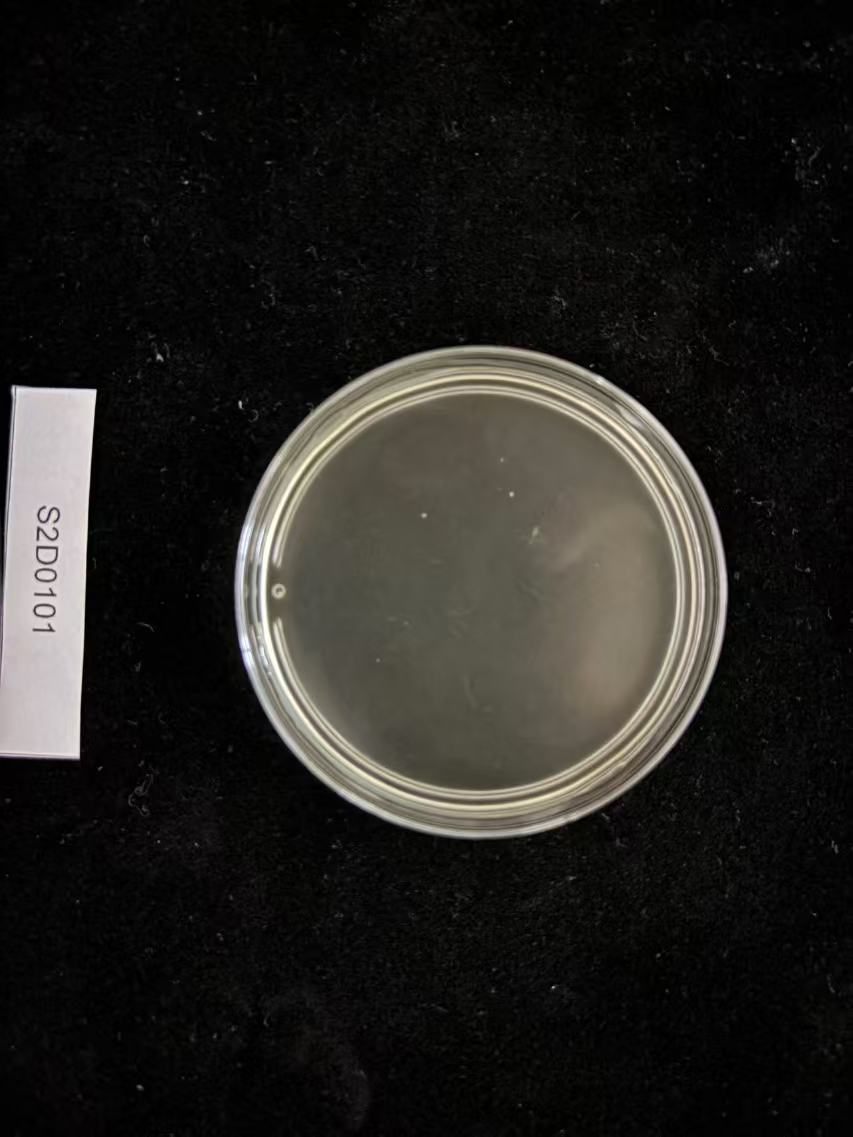

Supplement: Supplementary file 11 — Appendix Figure S2 Source Data [file 44319_2026_748_MOESM11_ESM.zip › Appendix Figure S2/S2B/Control_repeat1.jpg]

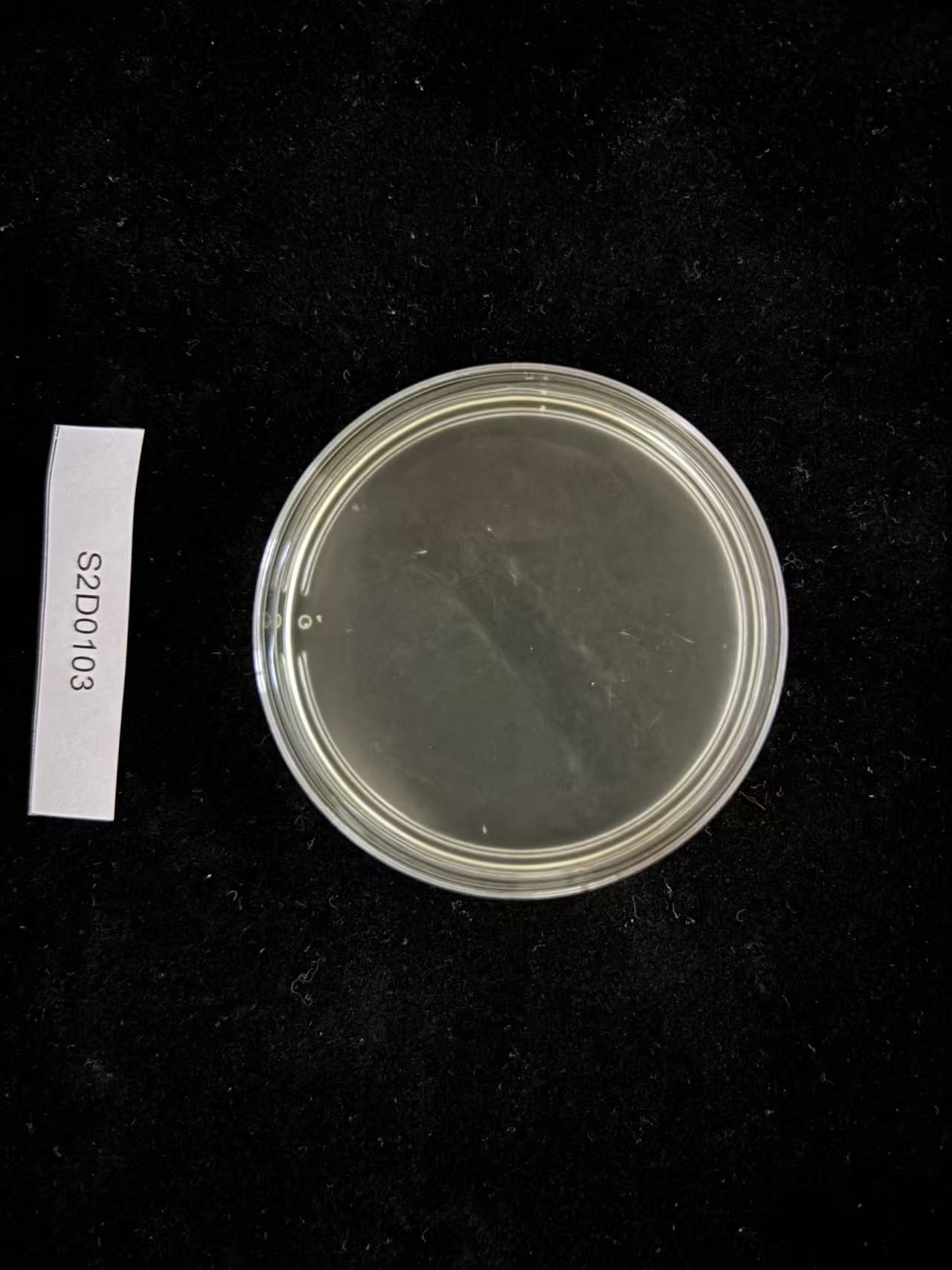

Supplement: Supplementary file 11 — Appendix Figure S2 Source Data [file 44319_2026_748_MOESM11_ESM.zip › Appendix Figure S2/S2B/Control_repeat3.jpg]

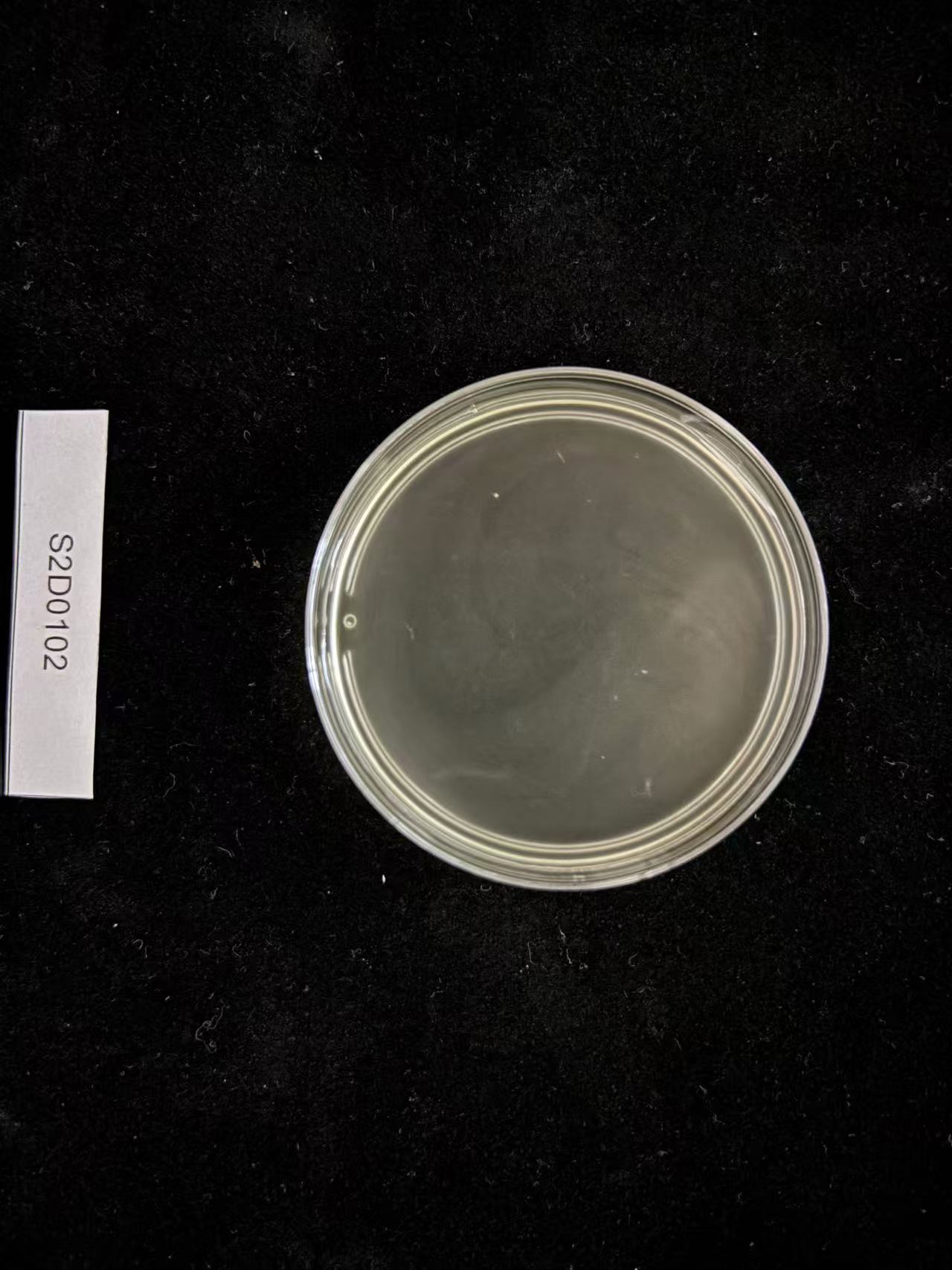

Supplement: Supplementary file 11 — Appendix Figure S2 Source Data [file 44319_2026_748_MOESM11_ESM.zip › Appendix Figure S2/S2B/Control_repeat2.jpg]

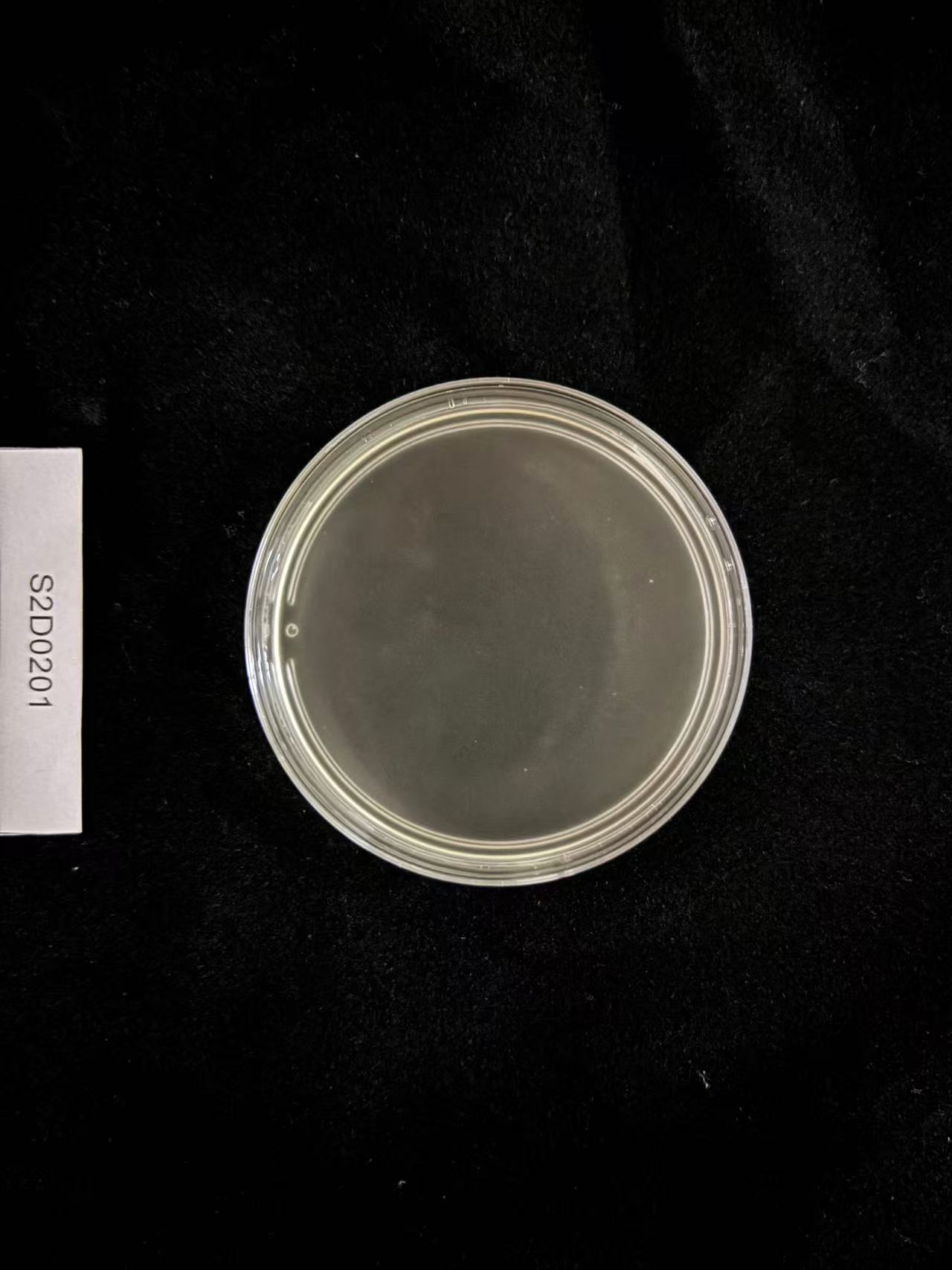

Supplement: Supplementary file 11 — Appendix Figure S2 Source Data [file 44319_2026_748_MOESM11_ESM.zip › Appendix Figure S2/S2B/CoCl2_repeat1.jpg]

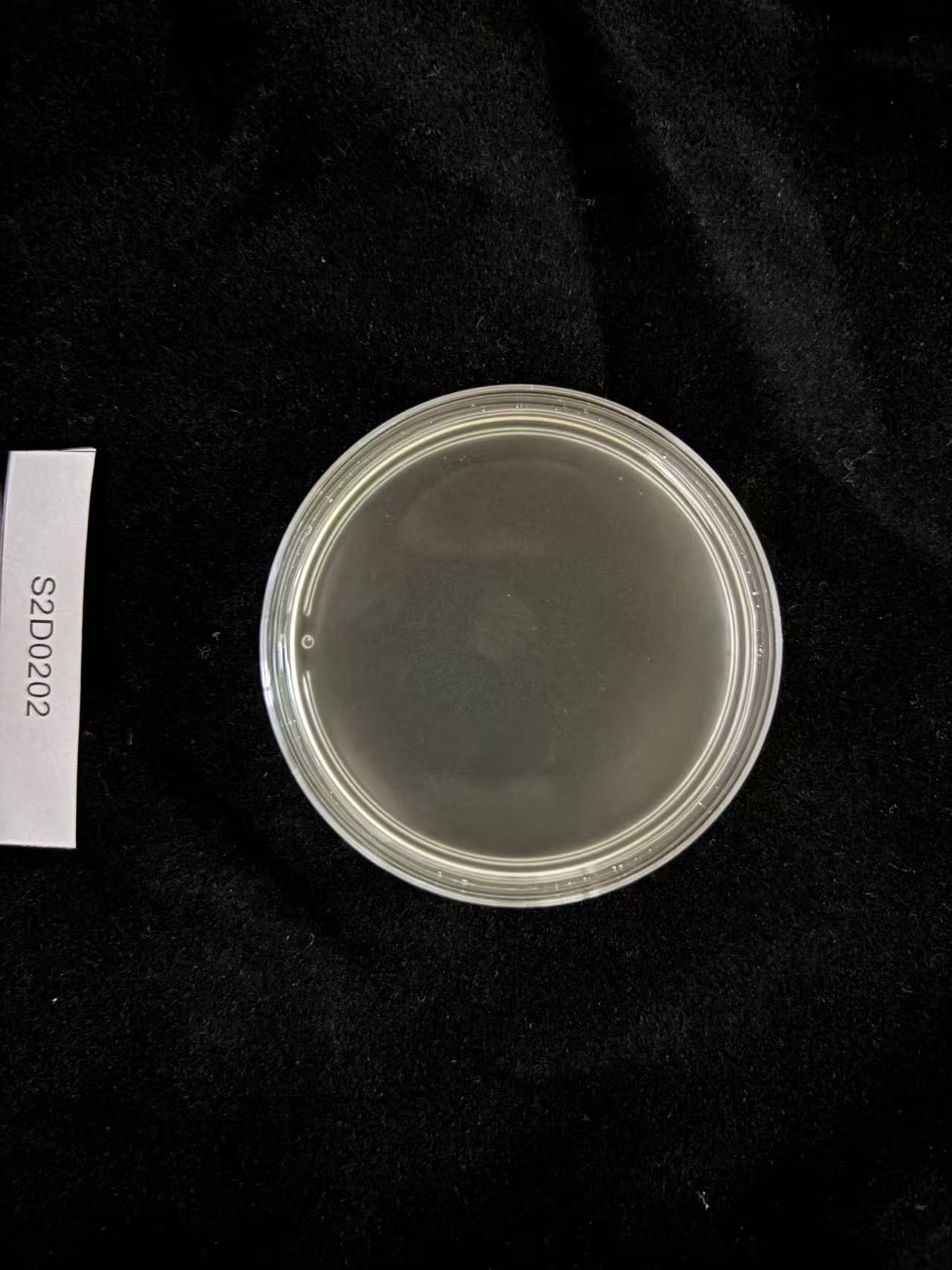

Supplement: Supplementary file 11 — Appendix Figure S2 Source Data [file 44319_2026_748_MOESM11_ESM.zip › Appendix Figure S2/S2B/CoCl2_repeat2.jpg]

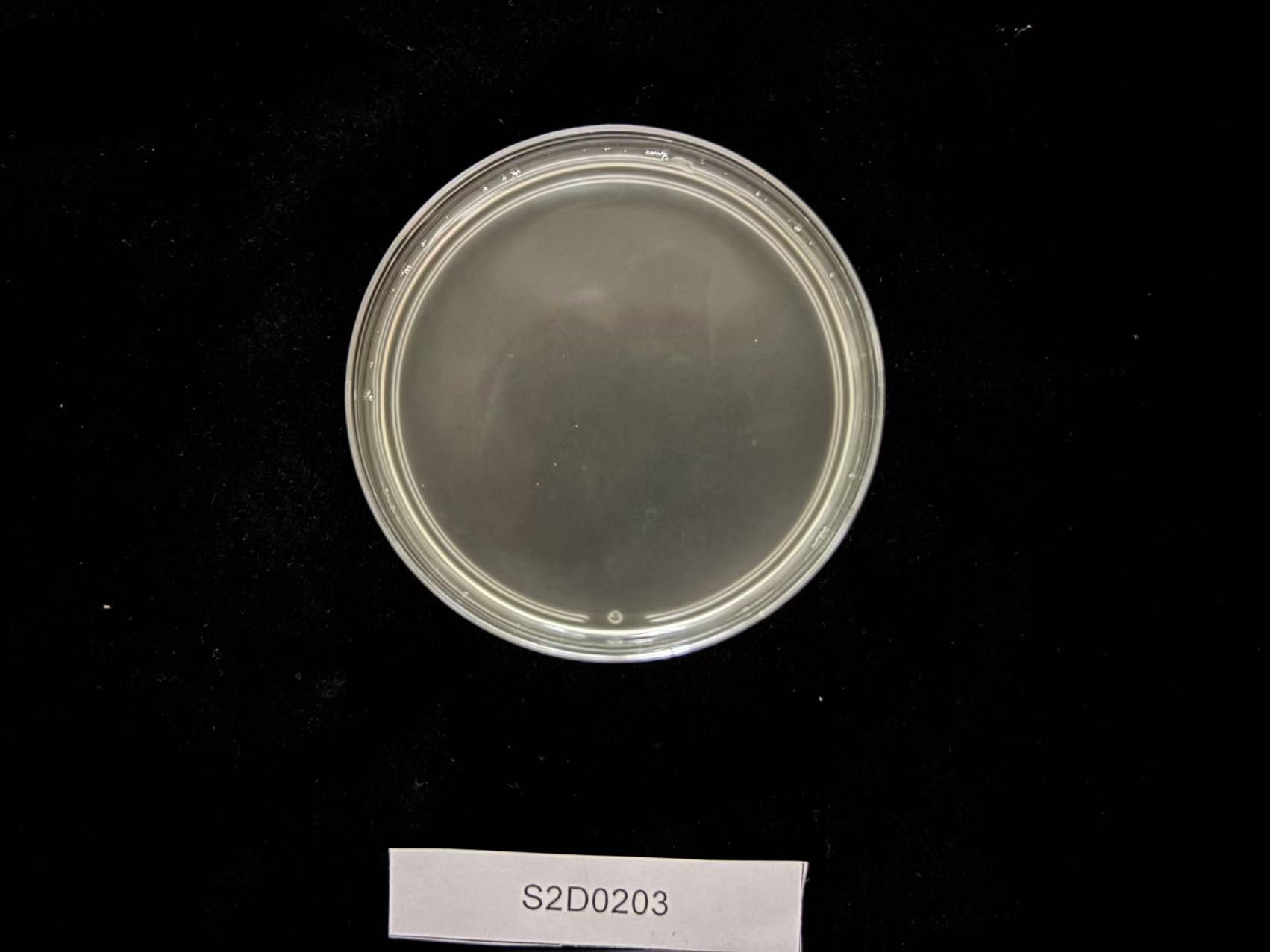

Supplement: Supplementary file 11 — Appendix Figure S2 Source Data [file 44319_2026_748_MOESM11_ESM.zip › Appendix Figure S2/S2B/CoCl2_repeat3.jpg]

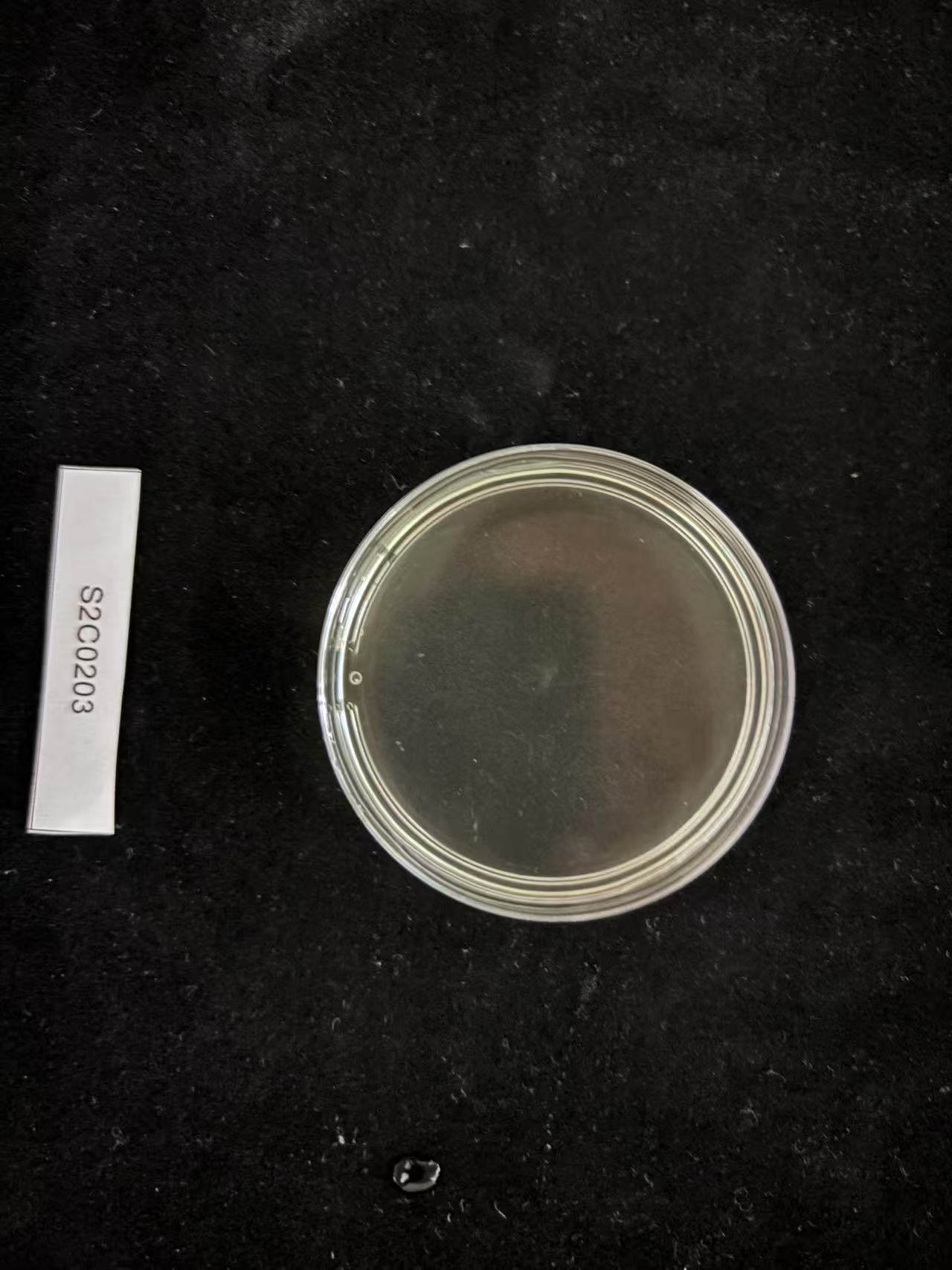

Supplement: Supplementary file 11 — Appendix Figure S2 Source Data [file 44319_2026_748_MOESM11_ESM.zip › Appendix Figure S2/S2C/CdCl2_Repeat.jpg]

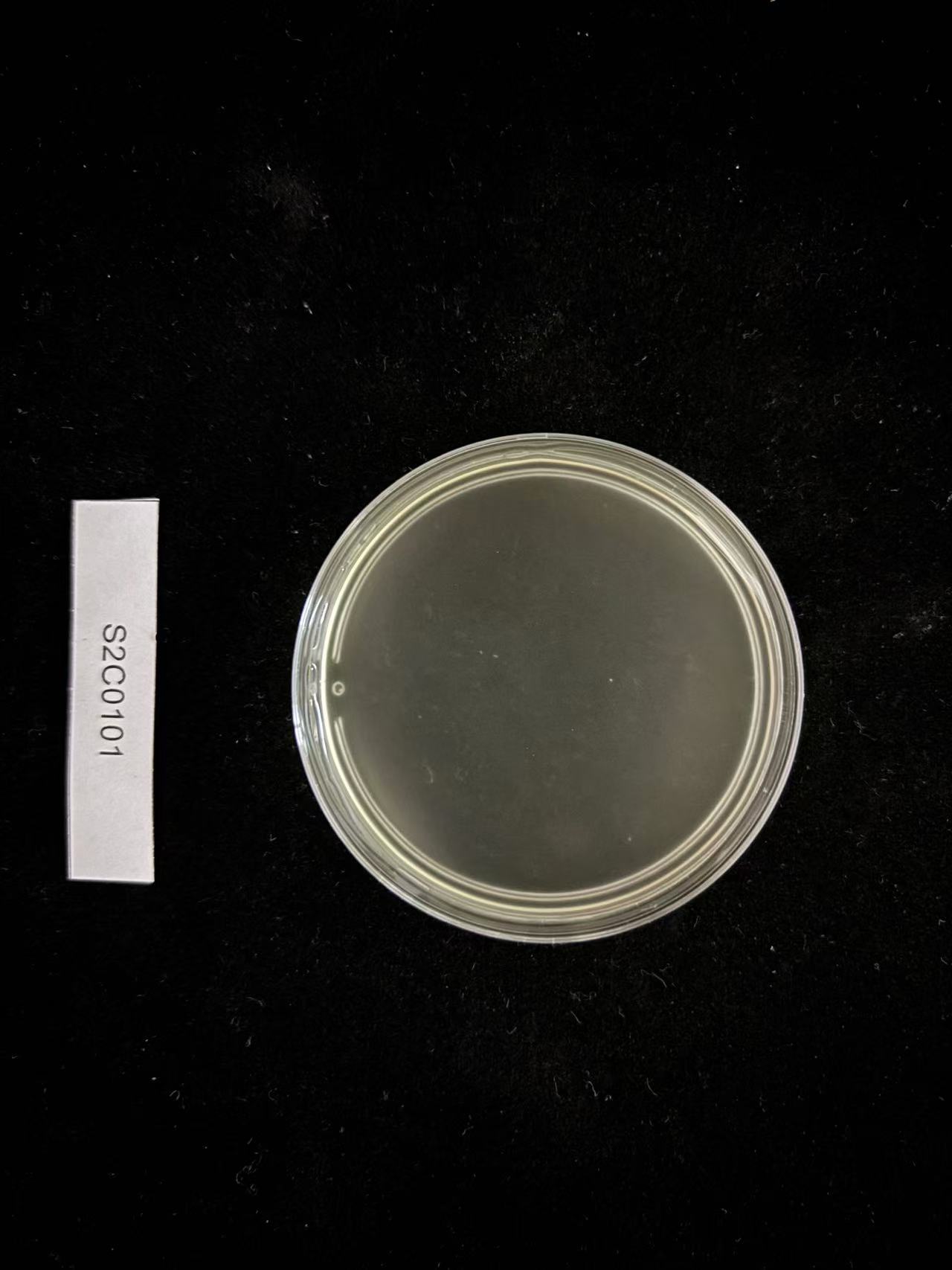

Supplement: Supplementary file 11 — Appendix Figure S2 Source Data [file 44319_2026_748_MOESM11_ESM.zip › Appendix Figure S2/S2C/Control_Repeat1.jpg]

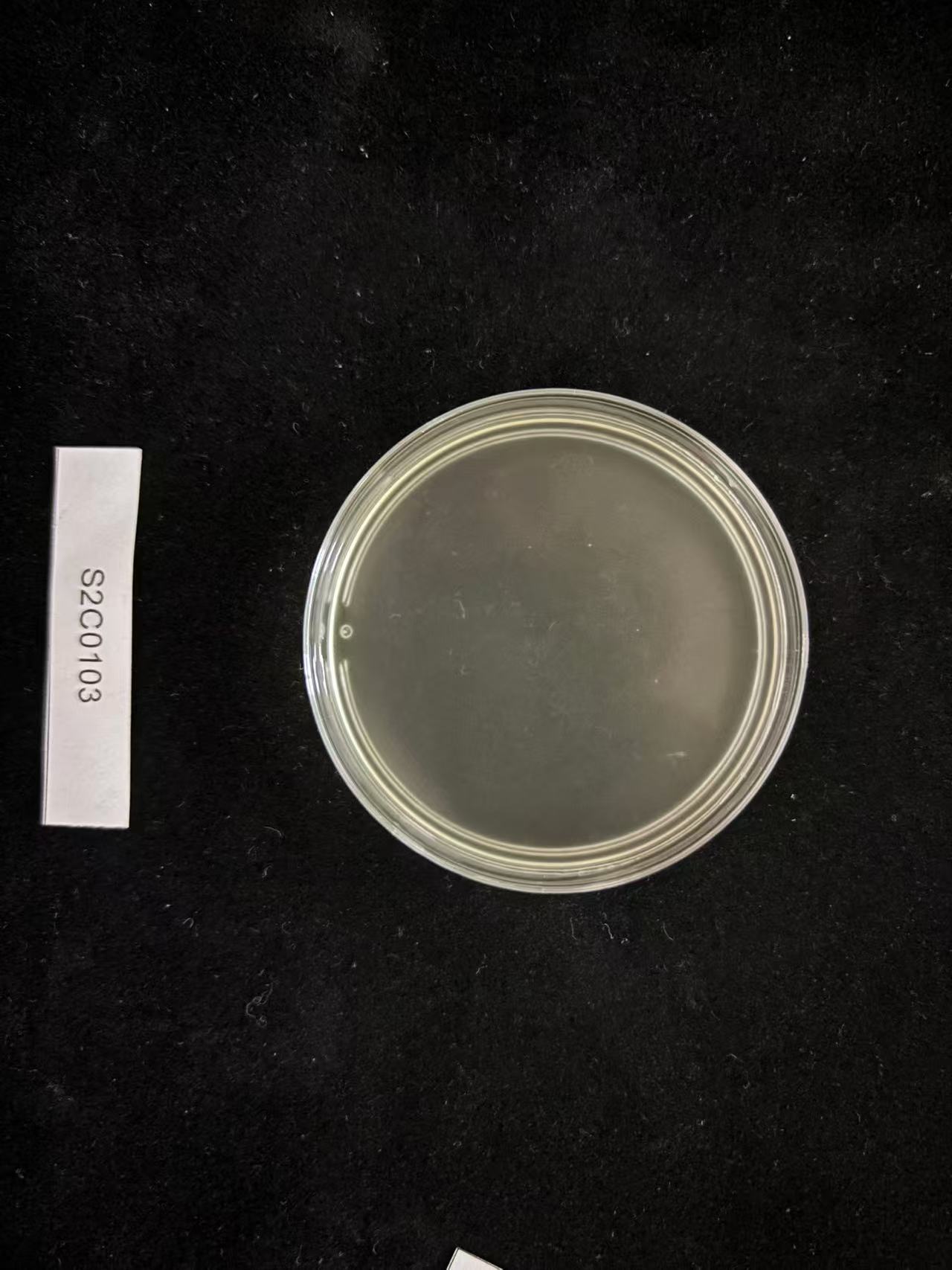

Supplement: Supplementary file 11 — Appendix Figure S2 Source Data [file 44319_2026_748_MOESM11_ESM.zip › Appendix Figure S2/S2C/Control_Repeat3.jpg]

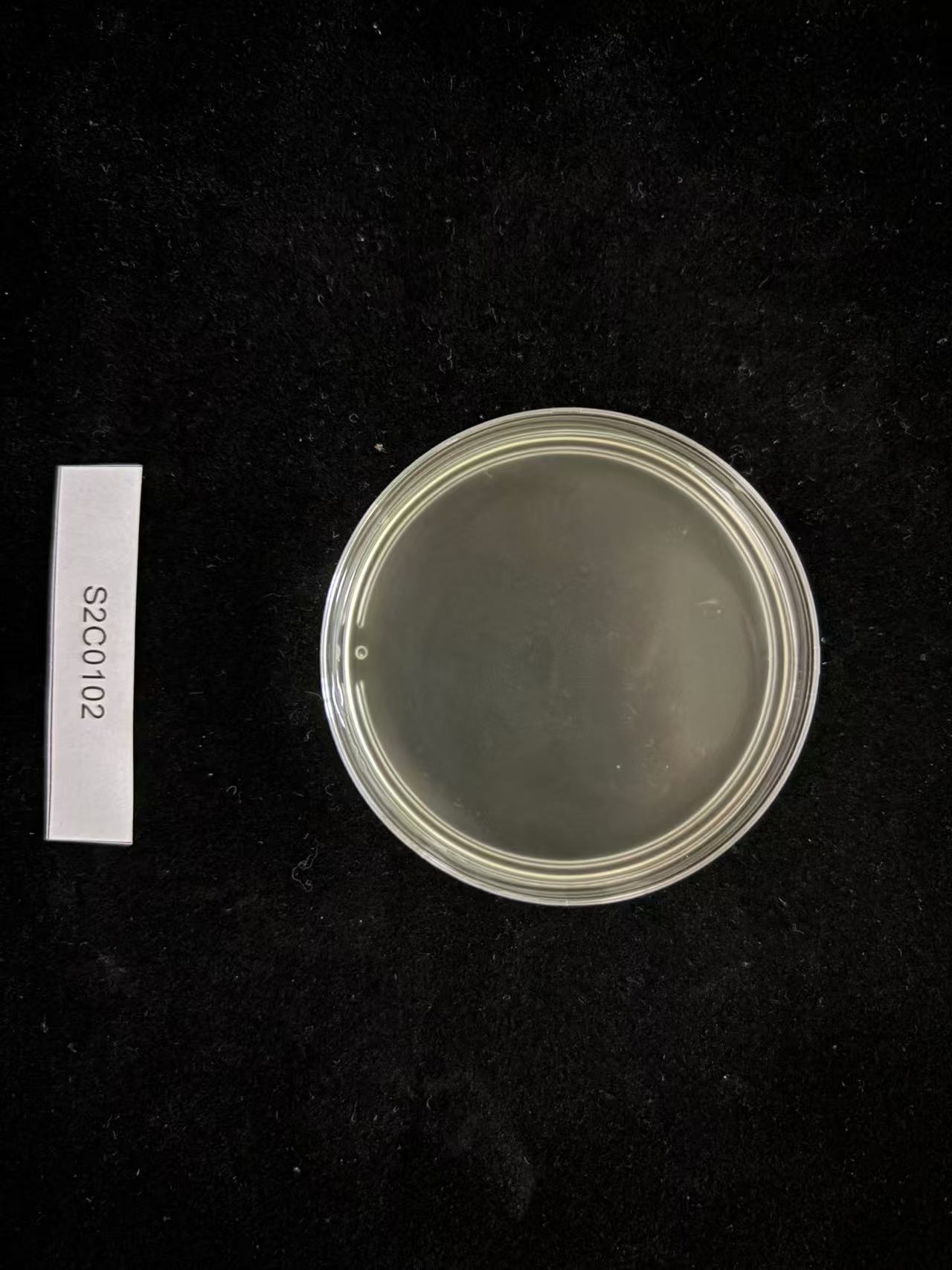

Supplement: Supplementary file 11 — Appendix Figure S2 Source Data [file 44319_2026_748_MOESM11_ESM.zip › Appendix Figure S2/S2C/Control_Repeat2.jpg]

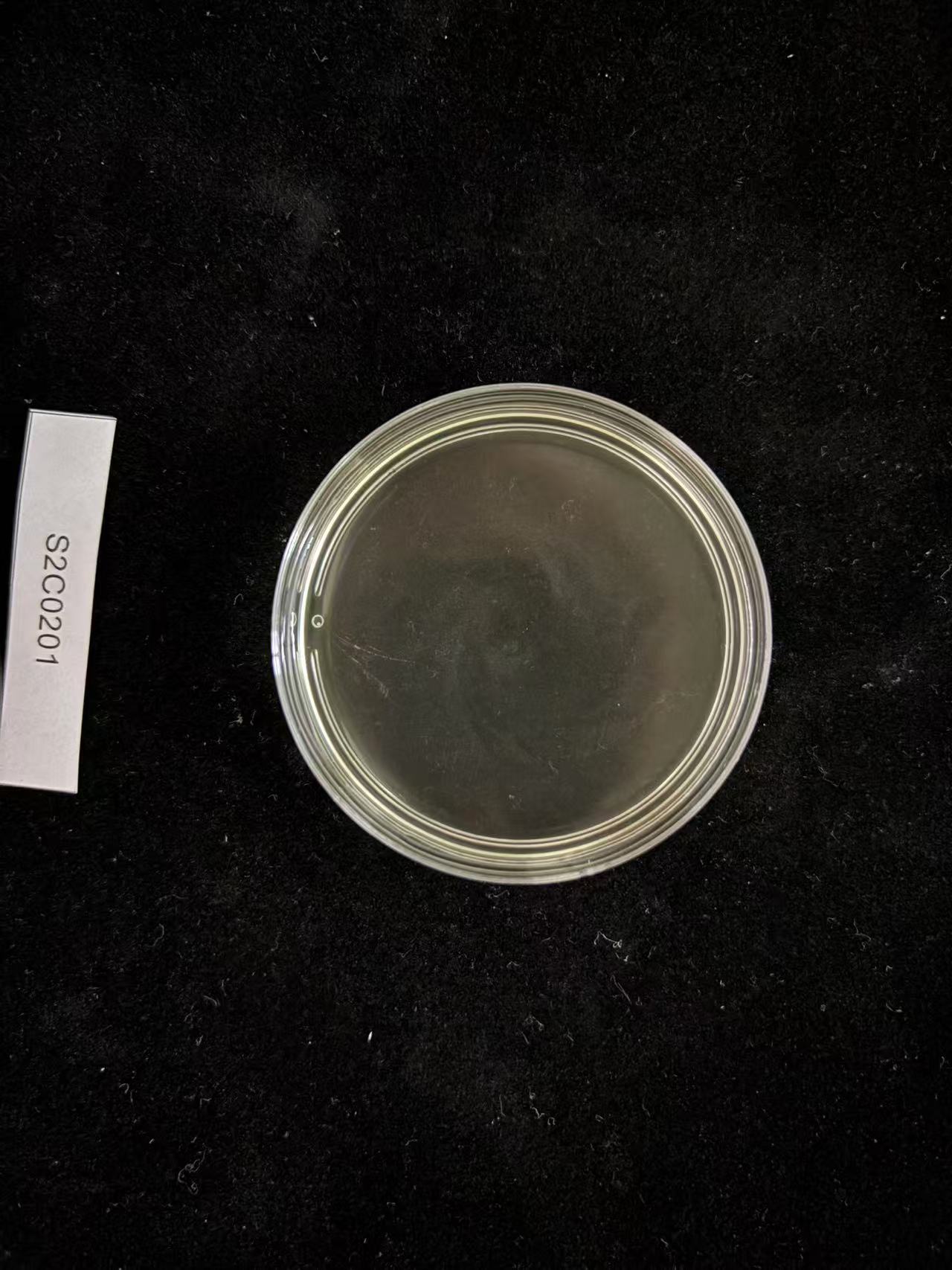

Supplement: Supplementary file 11 — Appendix Figure S2 Source Data [file 44319_2026_748_MOESM11_ESM.zip › Appendix Figure S2/S2C/CdCl2_Repeat1.jpg]

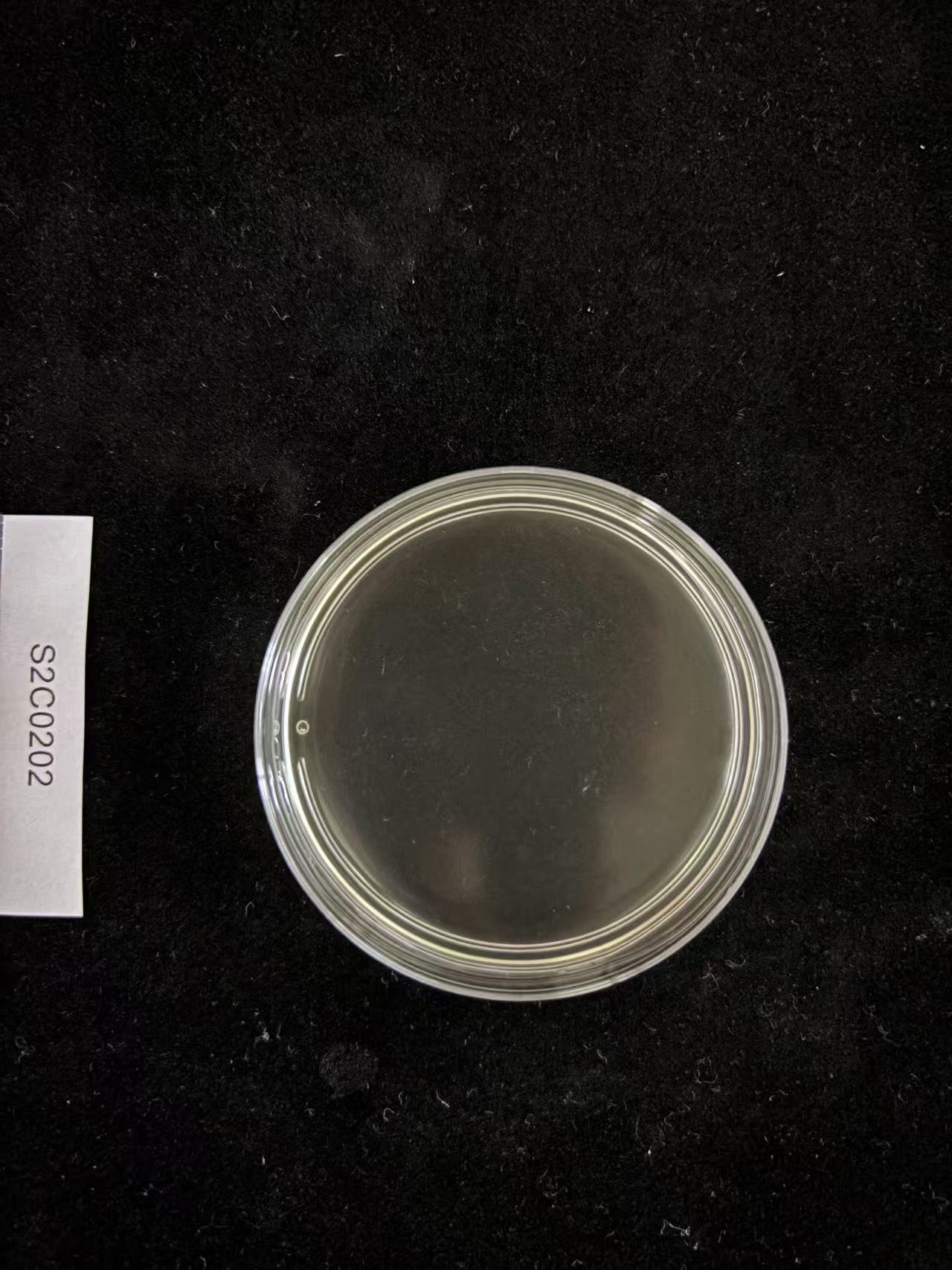

Supplement: Supplementary file 11 — Appendix Figure S2 Source Data [file 44319_2026_748_MOESM11_ESM.zip › Appendix Figure S2/S2C/CdCl2_Repeat2.jpg]

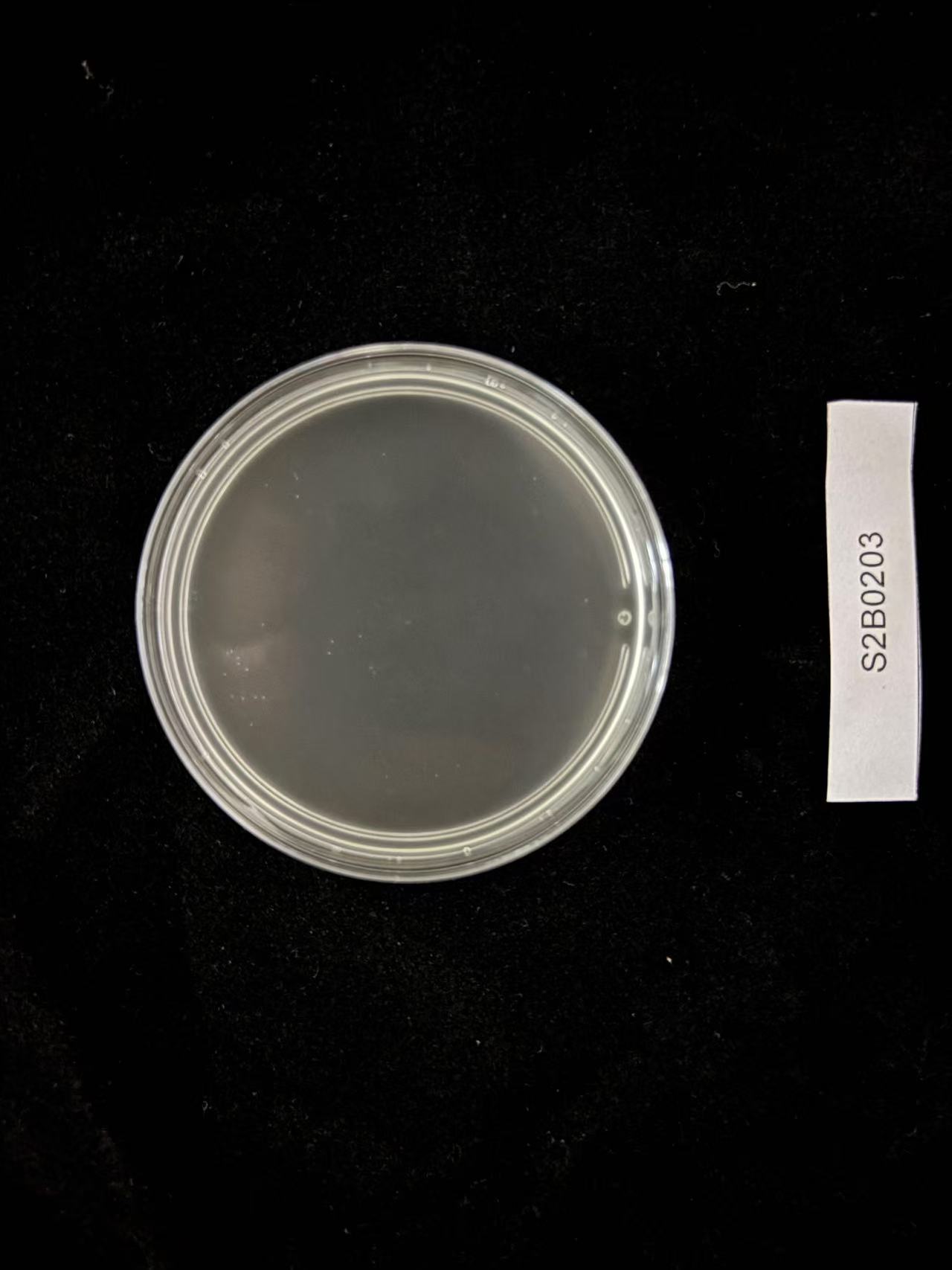

Supplement: Supplementary file 11 — Appendix Figure S2 Source Data [file 44319_2026_748_MOESM11_ESM.zip › Appendix Figure S2/S2D/37Γäâ_Repeat3.jpg]

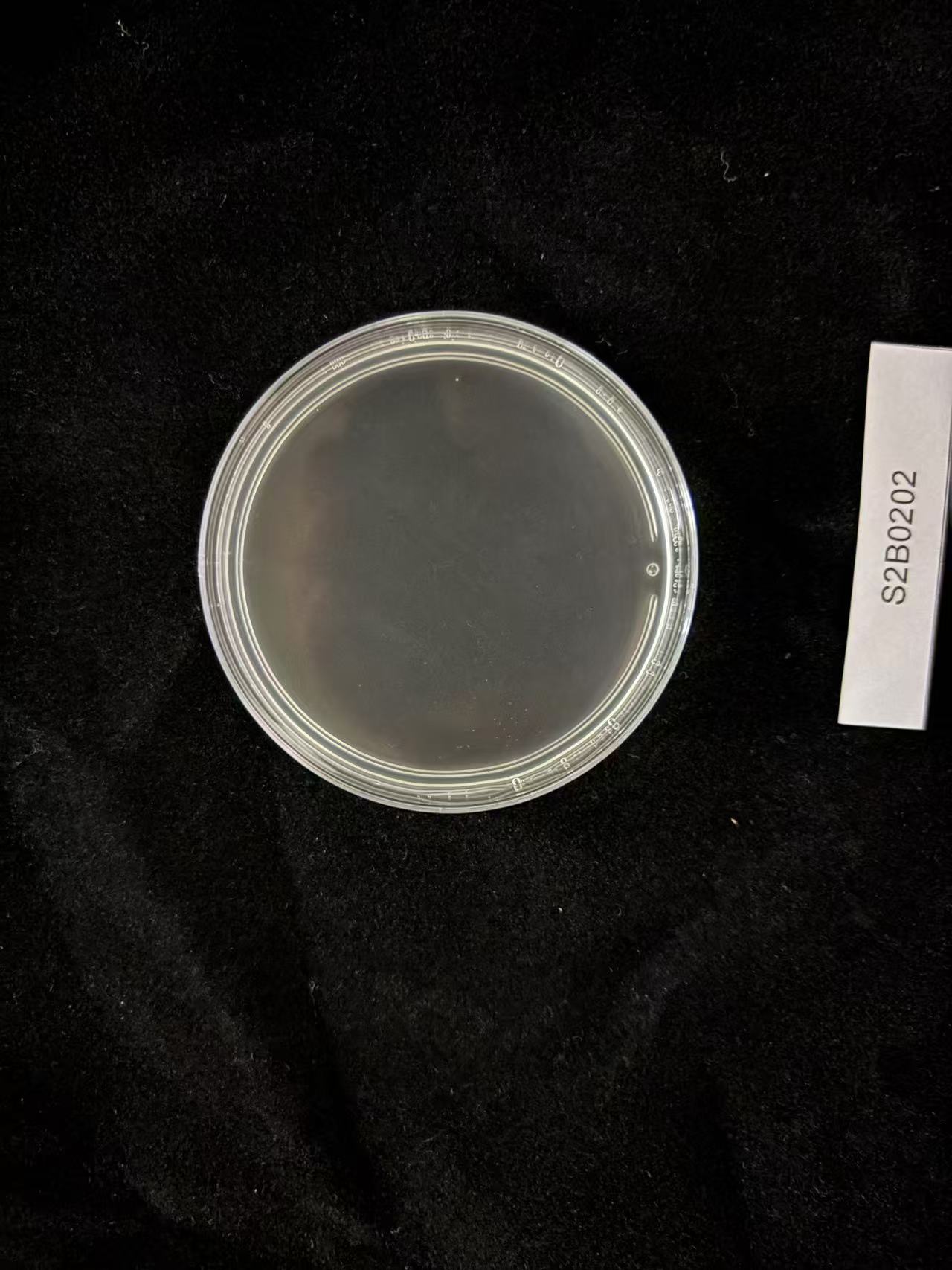

Supplement: Supplementary file 11 — Appendix Figure S2 Source Data [file 44319_2026_748_MOESM11_ESM.zip › Appendix Figure S2/S2D/37Γäâ_Repeat2.jpg]

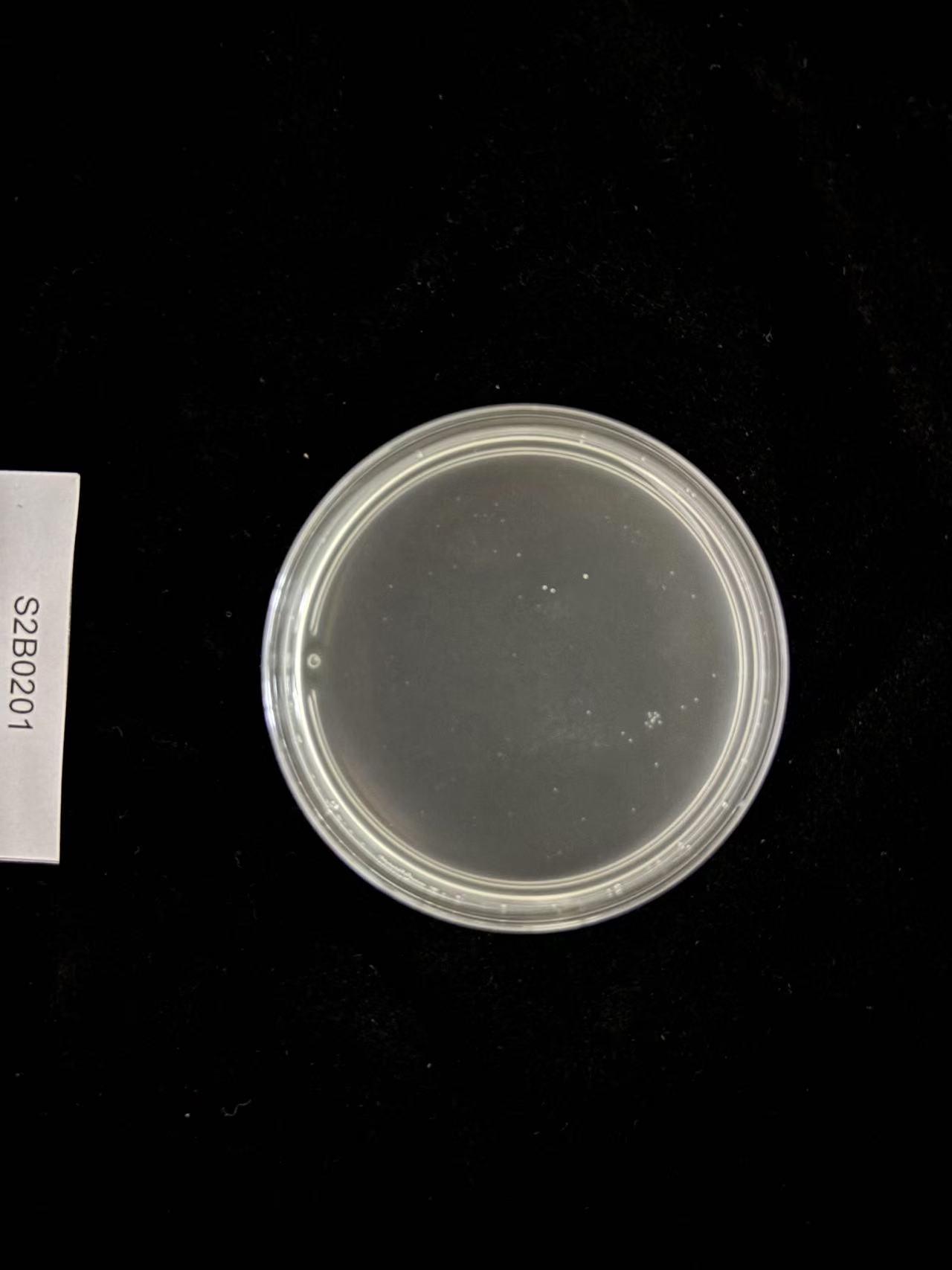

Supplement: Supplementary file 11 — Appendix Figure S2 Source Data [file 44319_2026_748_MOESM11_ESM.zip › Appendix Figure S2/S2D/37Γäâ_Repeat1.jpg]

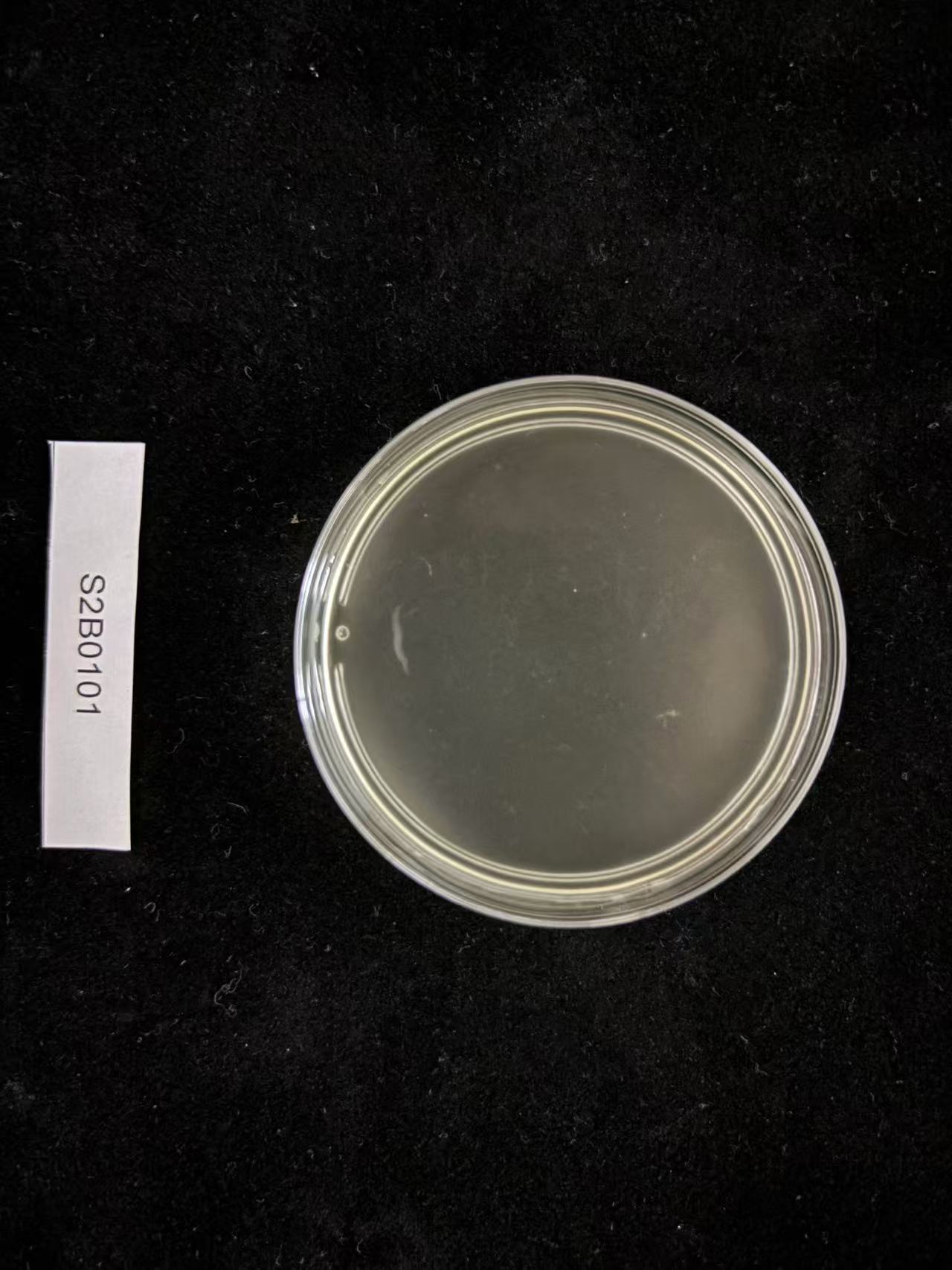

Supplement: Supplementary file 11 — Appendix Figure S2 Source Data [file 44319_2026_748_MOESM11_ESM.zip › Appendix Figure S2/S2D/Control_Repeat1.jpg]

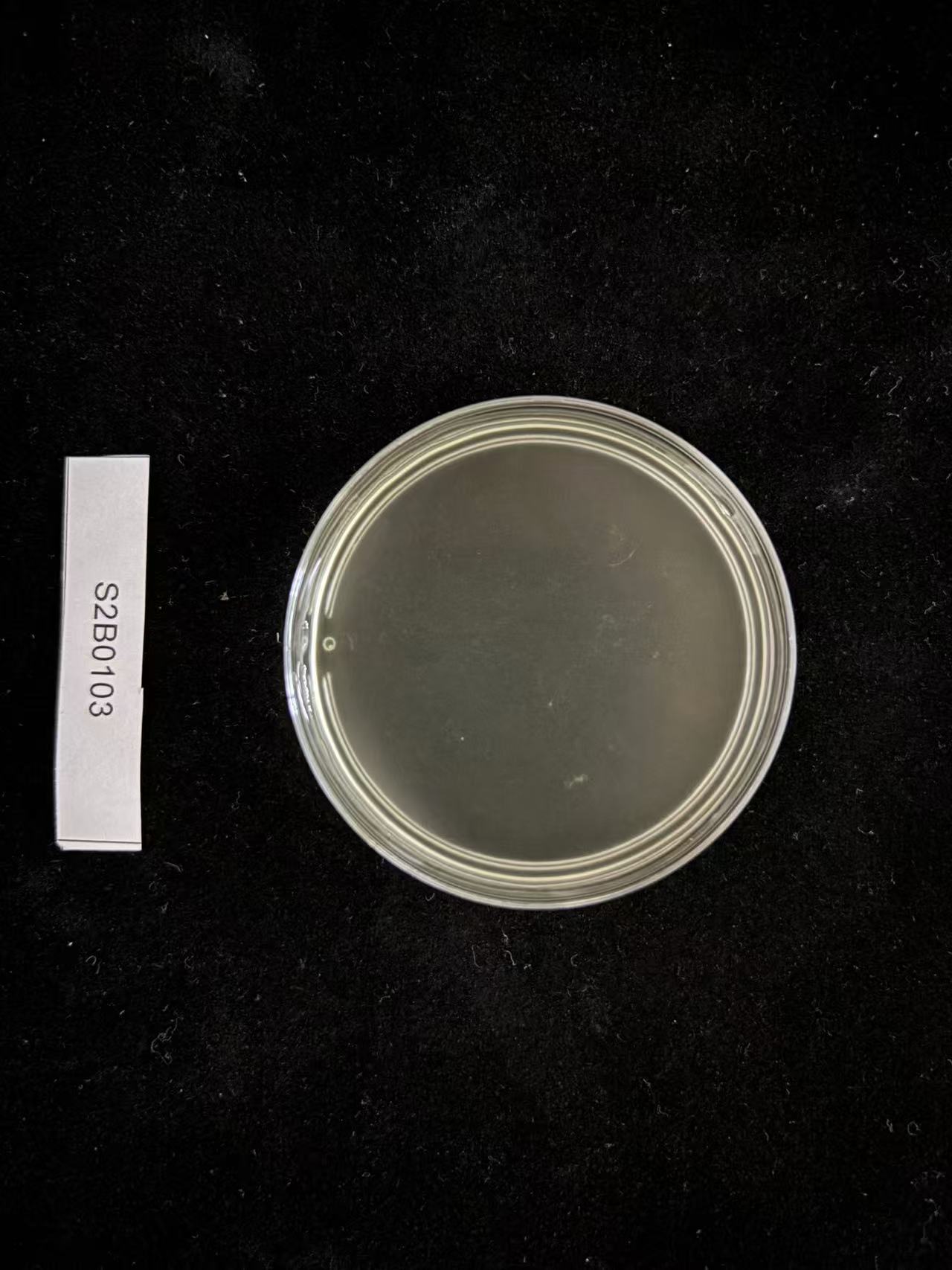

Supplement: Supplementary file 11 — Appendix Figure S2 Source Data [file 44319_2026_748_MOESM11_ESM.zip › Appendix Figure S2/S2D/Control_Repeat3.jpg]

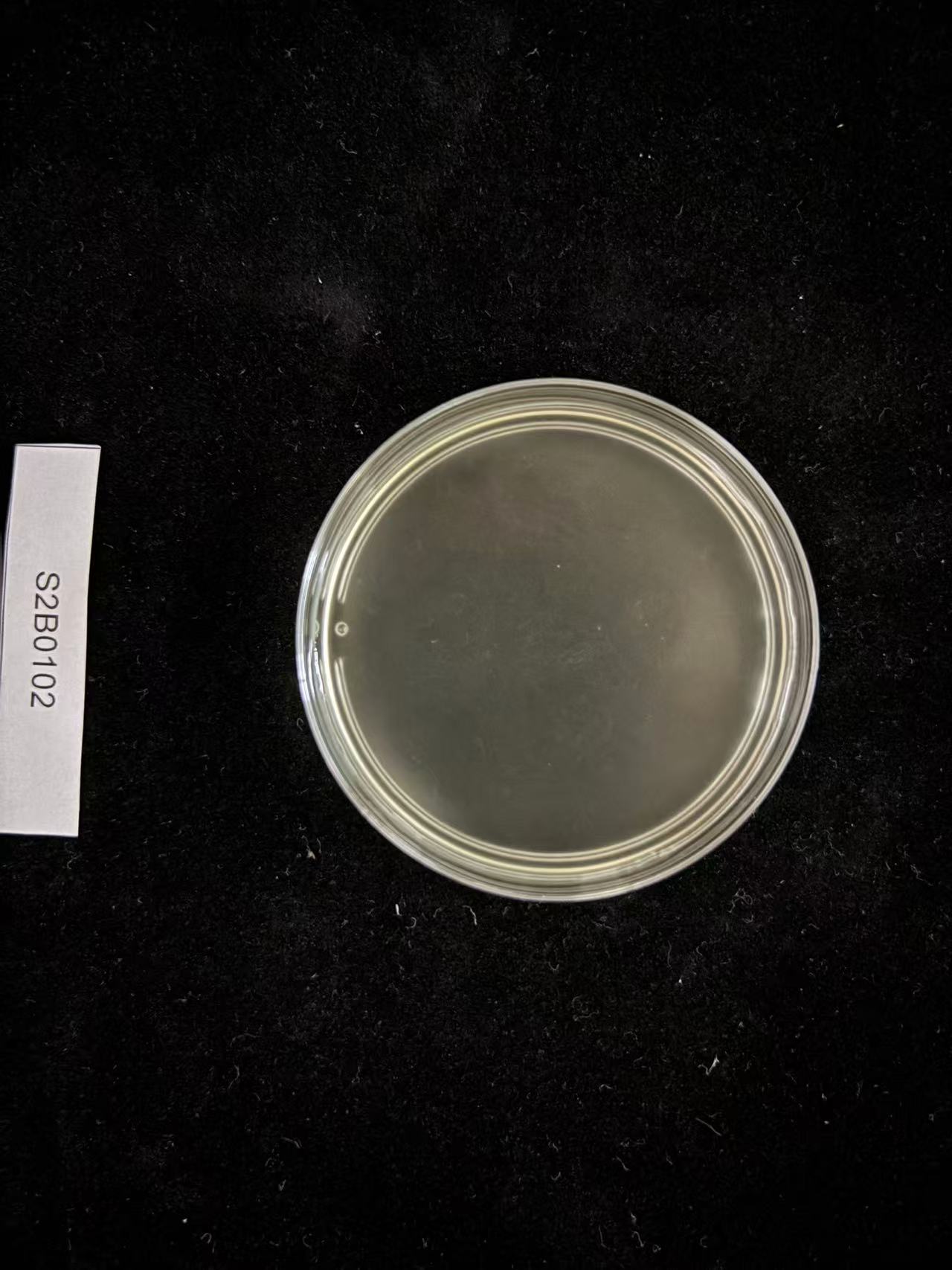

Supplement: Supplementary file 11 — Appendix Figure S2 Source Data [file 44319_2026_748_MOESM11_ESM.zip › Appendix Figure S2/S2D/Control_Repeat2.jpg]

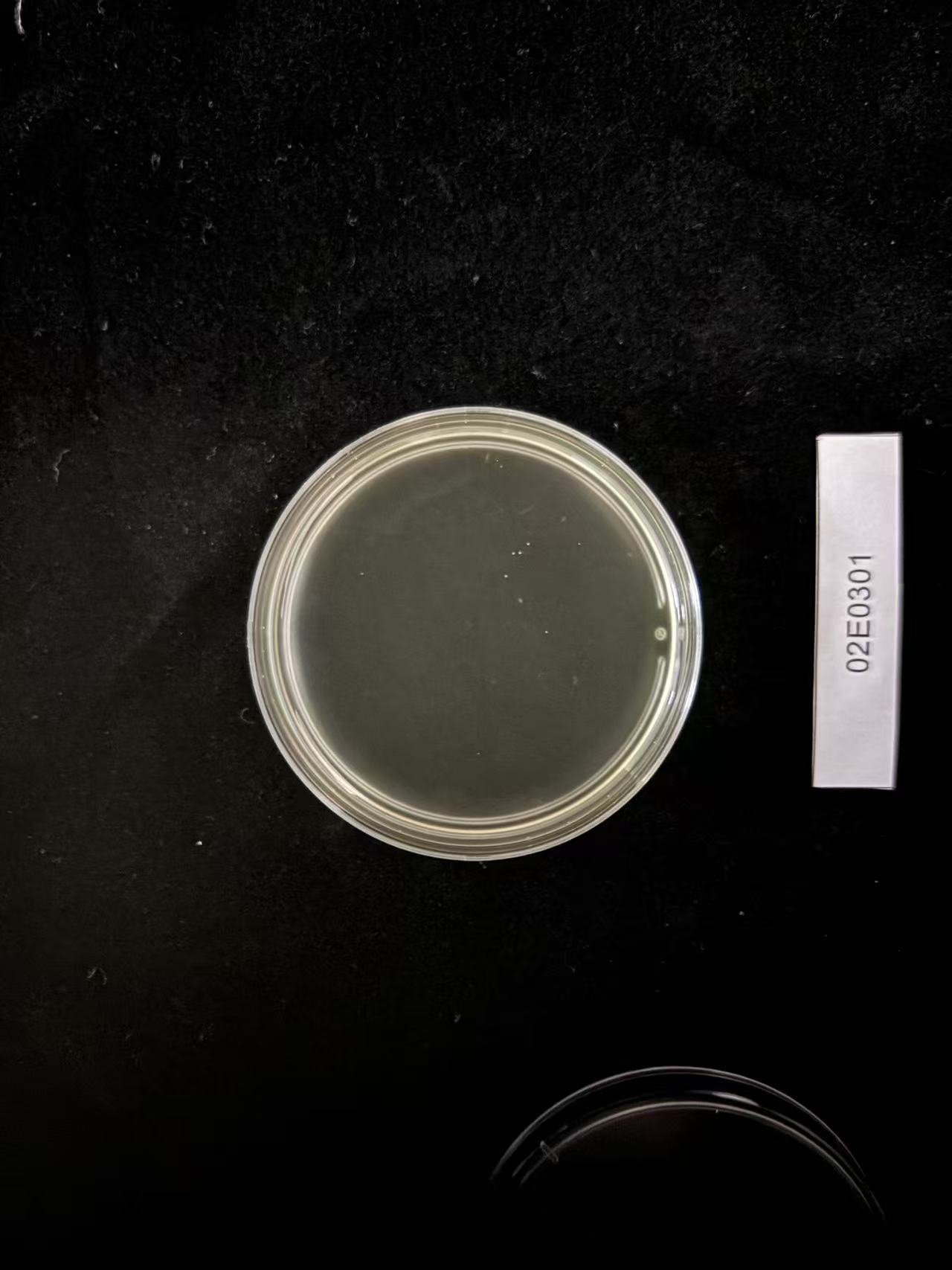

Supplement: Supplementary file 12 — Appendix Figure S3 Source Data [file 44319_2026_748_MOESM12_ESM.zip › Appendix Figure S3/S3C/oxalic acid_galactose_repeat2.jpg]

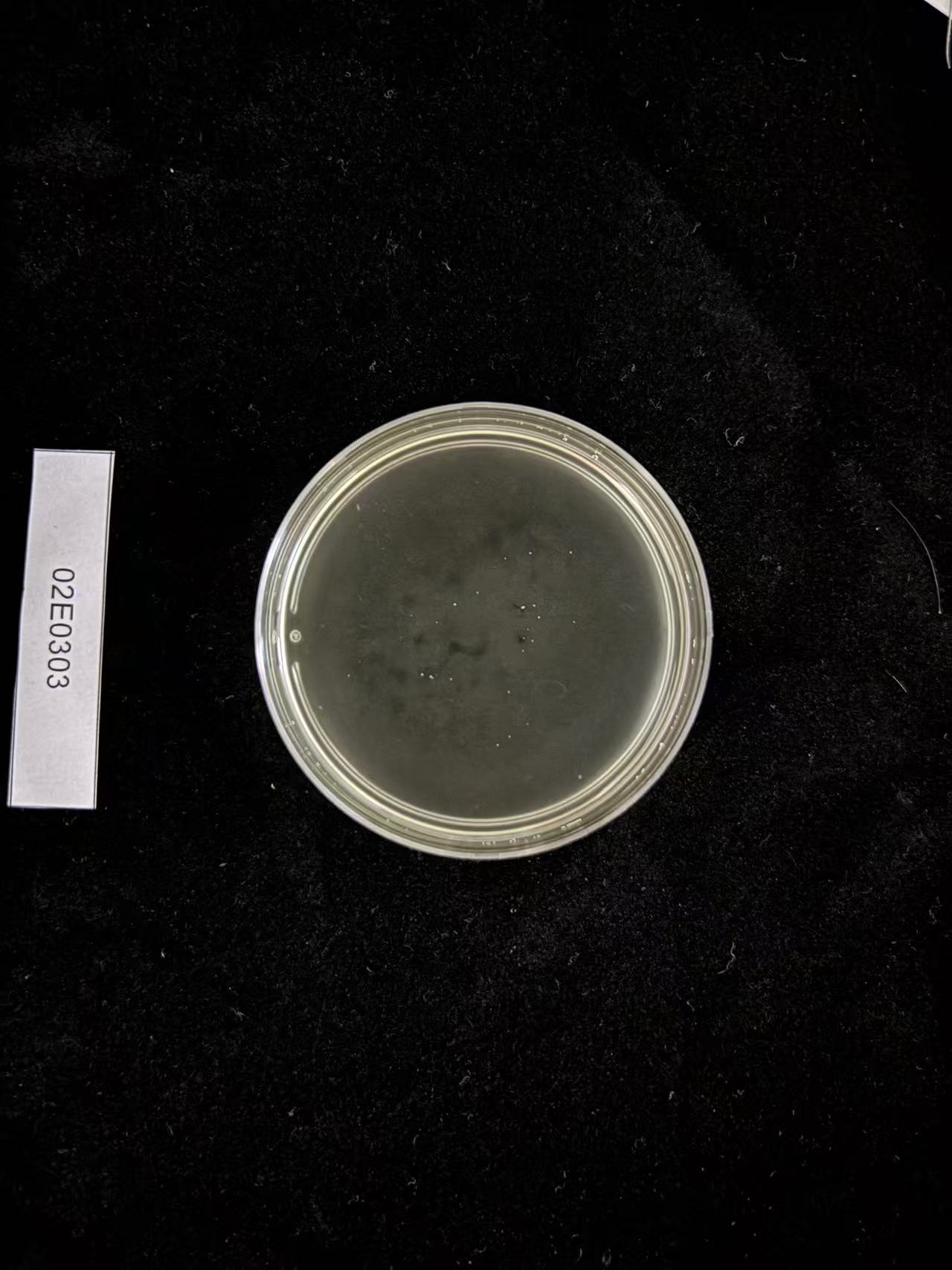

Supplement: Supplementary file 12 — Appendix Figure S3 Source Data [file 44319_2026_748_MOESM12_ESM.zip › Appendix Figure S3/S3C/oxalic acid_galactose_repeat3.jpg]

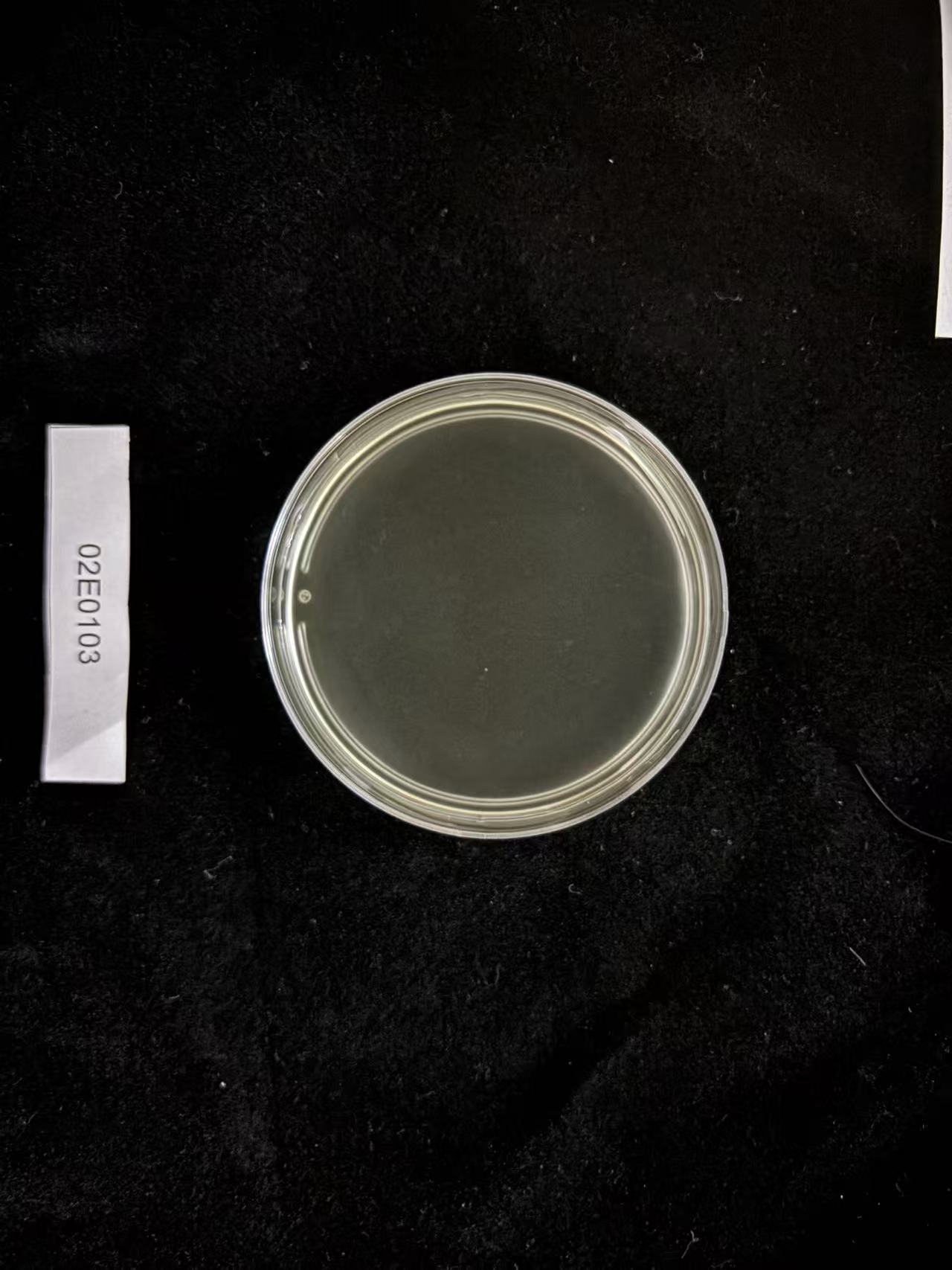

Supplement: Supplementary file 12 — Appendix Figure S3 Source Data [file 44319_2026_748_MOESM12_ESM.zip › Appendix Figure S3/S3C/Control_Repeat3.jpg]
