# Supplementary figures and images for: A gradient green-beard gene in fission yeast (part 2 of 2)
Source: EMBO Rep. 2026 Mar 16;27(8):1904–17. doi: 10.1038/s44319-026-00748-x (PMC13121626; doi:10.1038/s44319-026-00748-x)

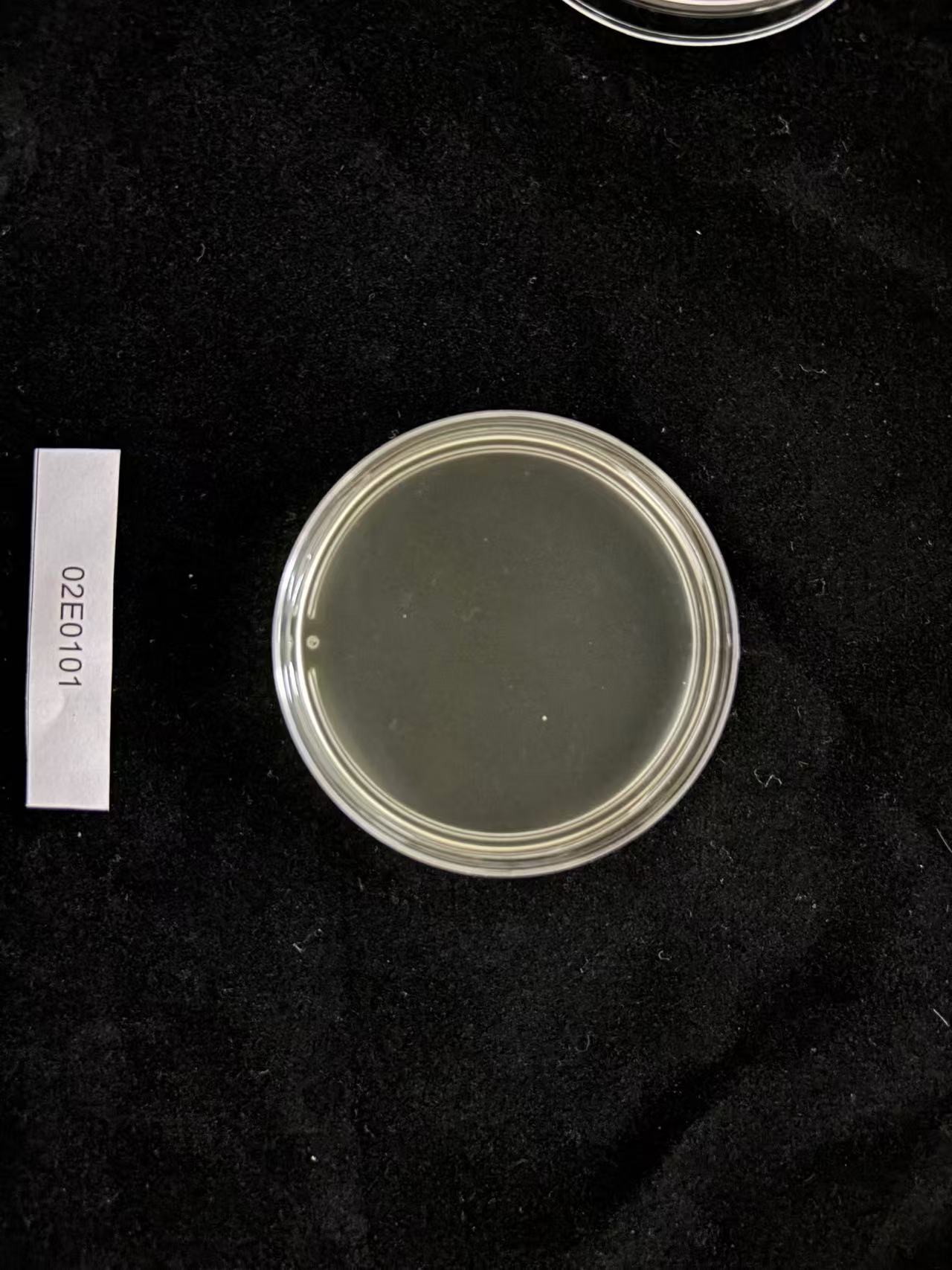

Supplement: Supplementary file 12 — Appendix Figure S3 Source Data [file 44319_2026_748_MOESM12_ESM.zip › Appendix Figure S3/S3C/Control_Repeat2.jpg]

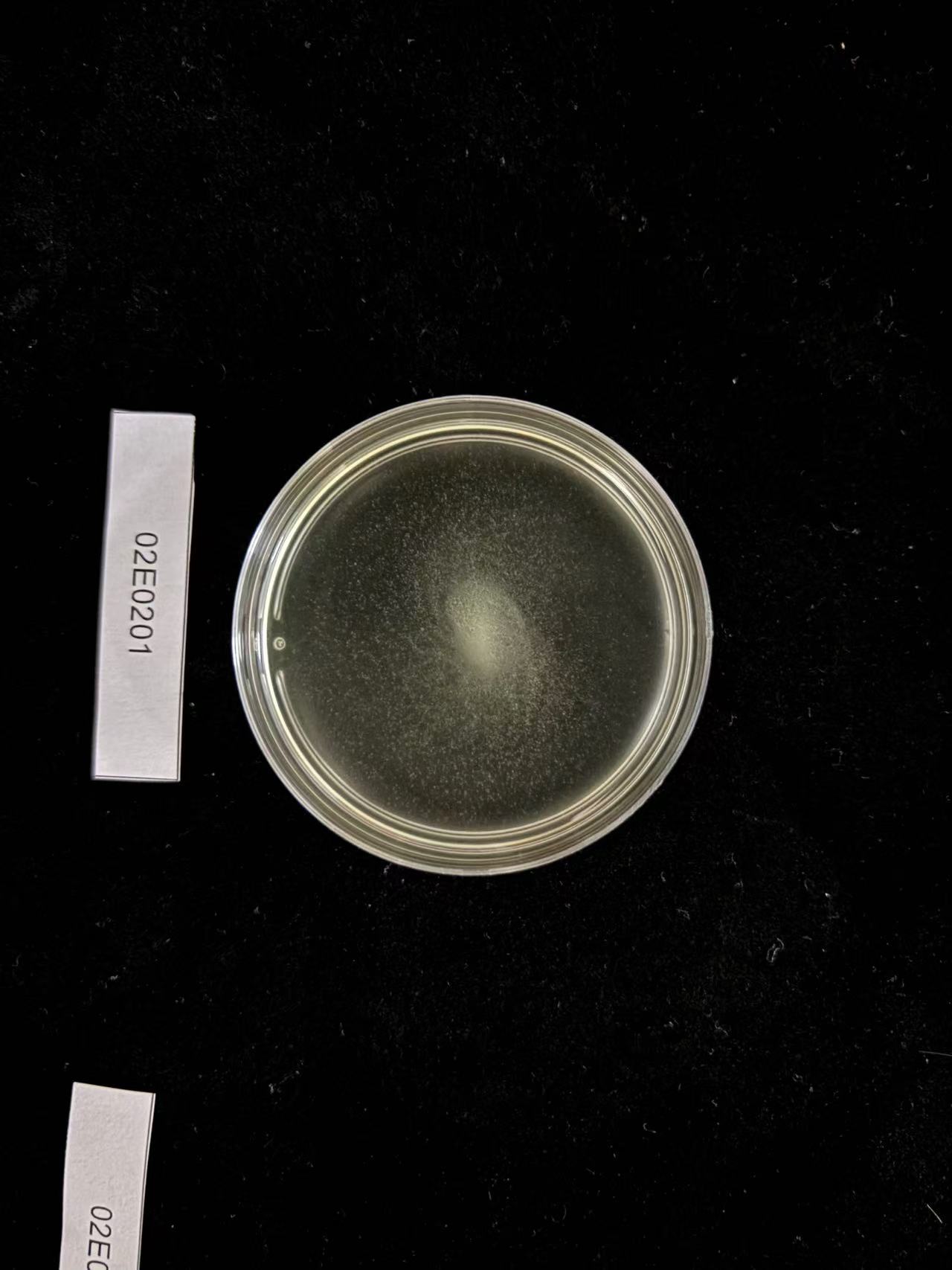

Supplement: Supplementary file 12 — Appendix Figure S3 Source Data [file 44319_2026_748_MOESM12_ESM.zip › Appendix Figure S3/S3C/oxalic acid_repeat2.jpg]

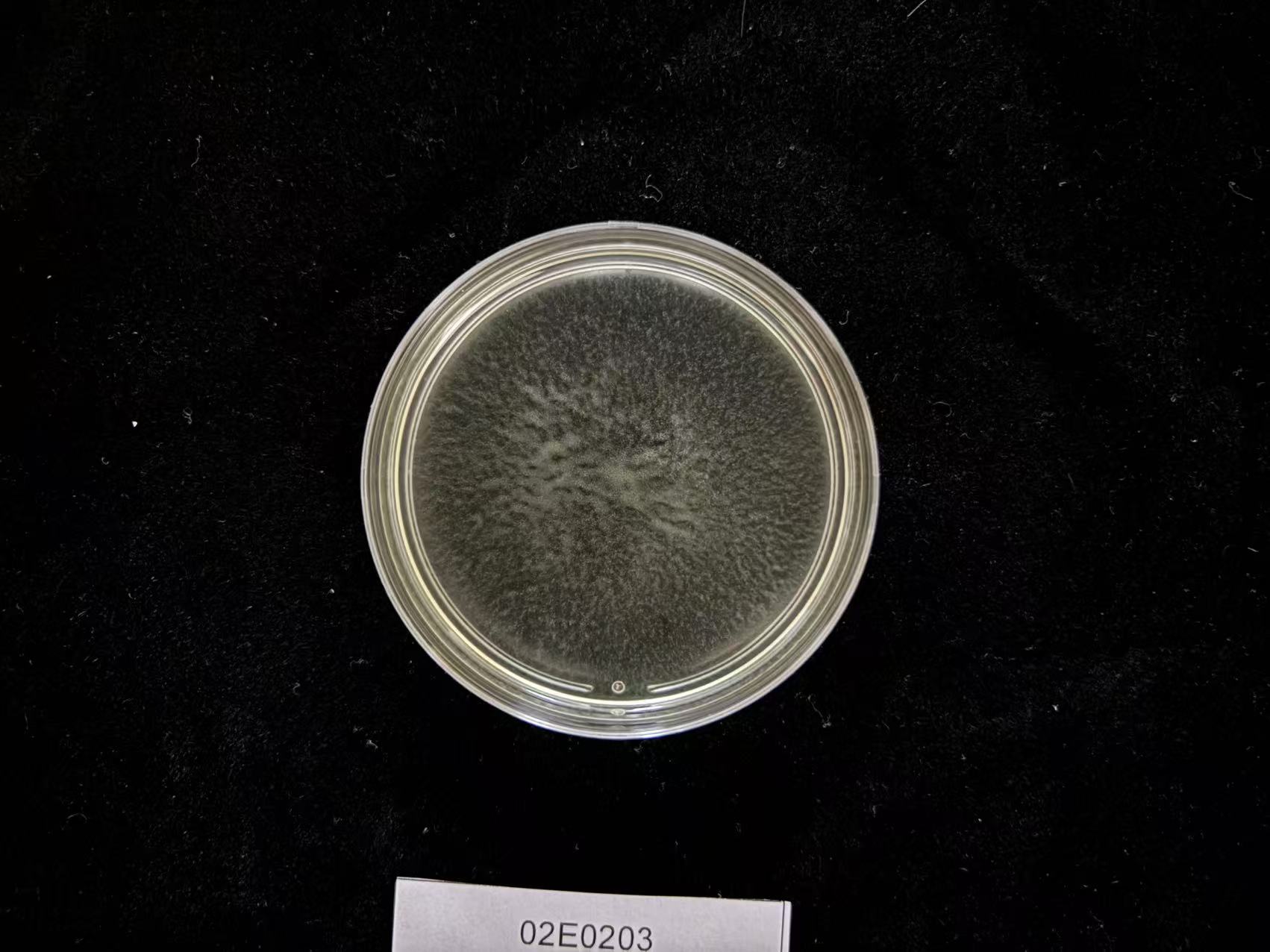

Supplement: Supplementary file 12 — Appendix Figure S3 Source Data [file 44319_2026_748_MOESM12_ESM.zip › Appendix Figure S3/S3C/oxalic acid_repeat3.jpg]

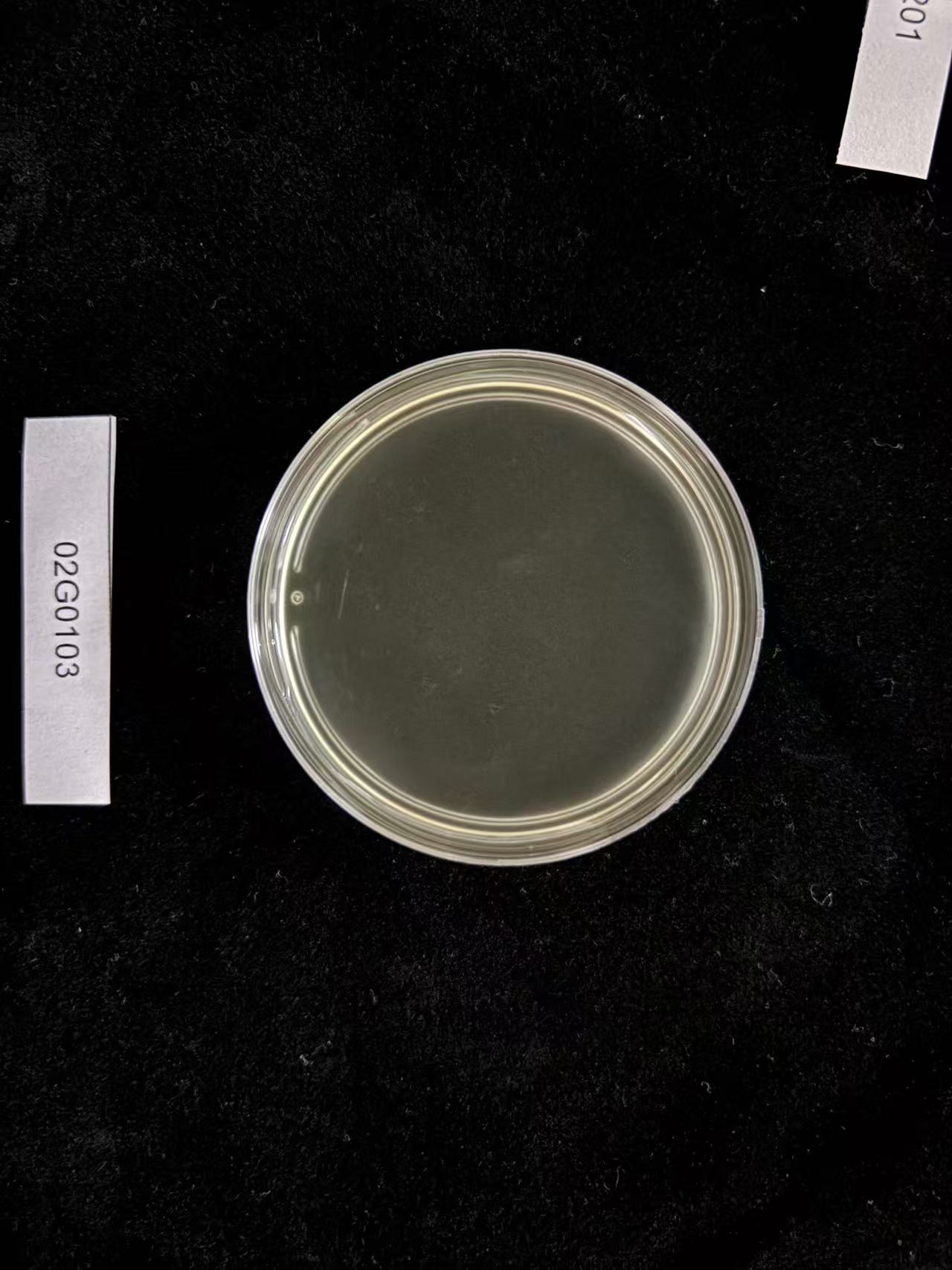

Supplement: Supplementary file 12 — Appendix Figure S3 Source Data [file 44319_2026_748_MOESM12_ESM.zip › Appendix Figure S3/S3D/Control_Repeat3.jpg]

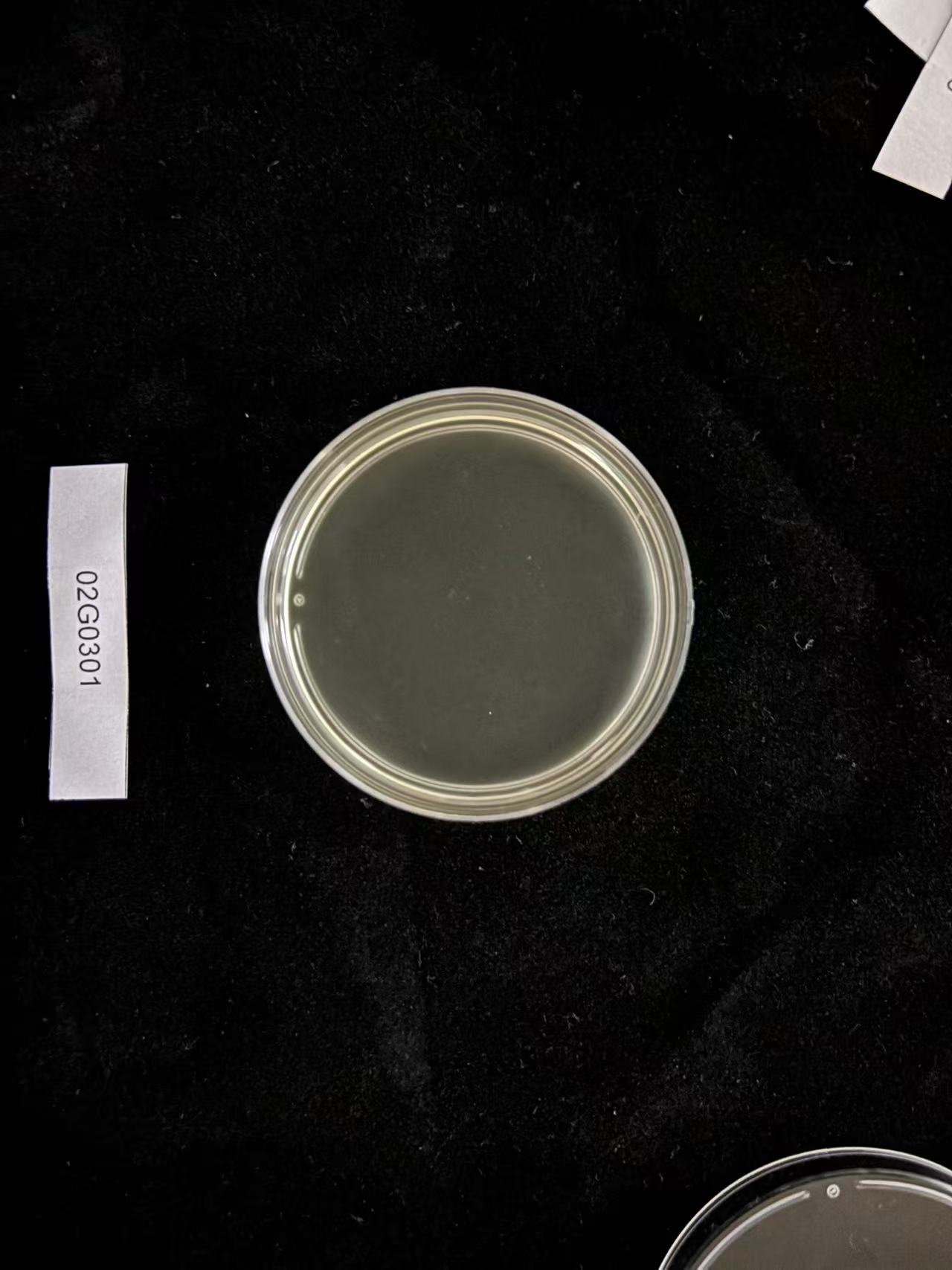

Supplement: Supplementary file 12 — Appendix Figure S3 Source Data [file 44319_2026_748_MOESM12_ESM.zip › Appendix Figure S3/S3D/nitric acid_galactose_repeat2.jpg]

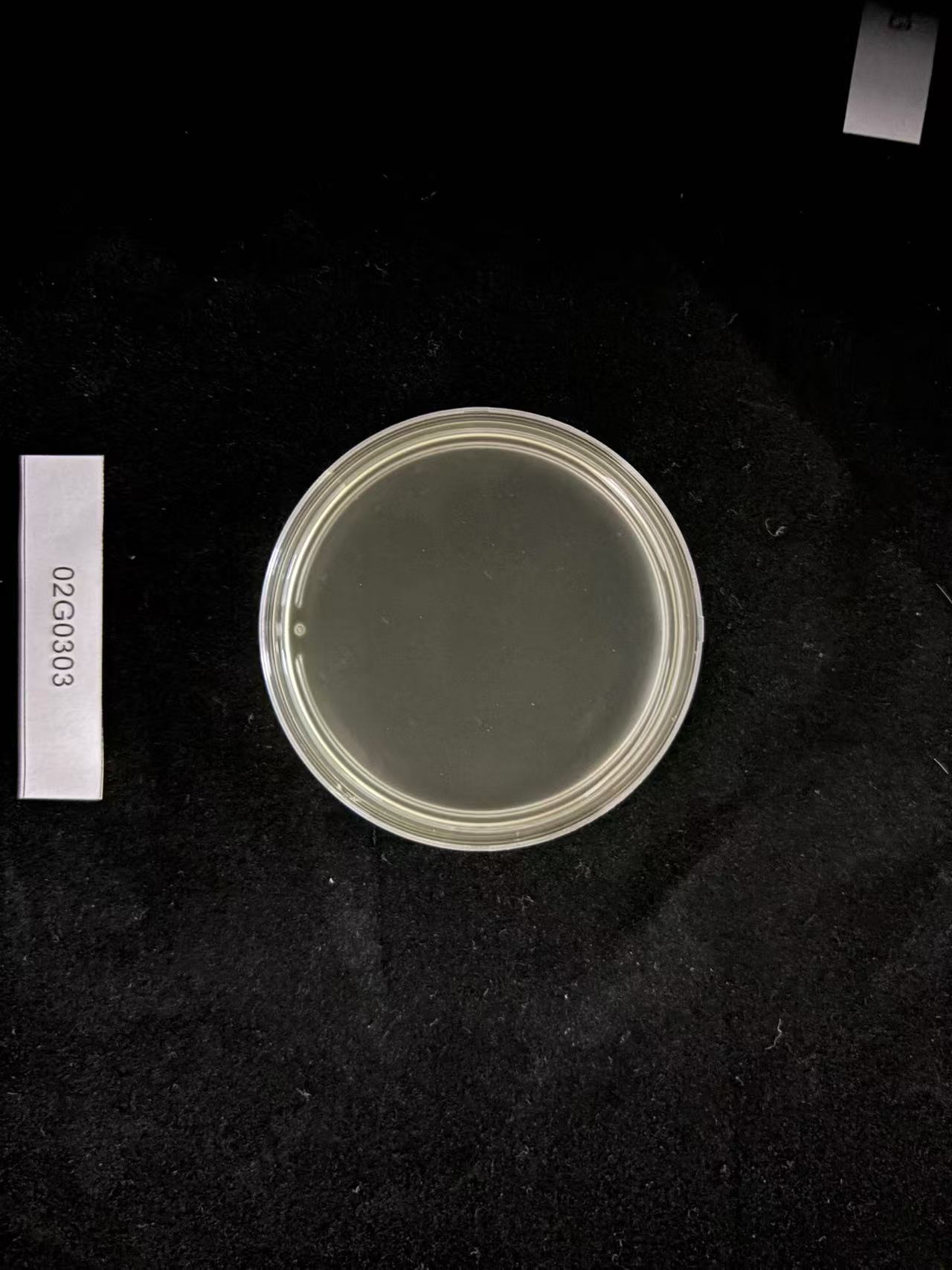

Supplement: Supplementary file 12 — Appendix Figure S3 Source Data [file 44319_2026_748_MOESM12_ESM.zip › Appendix Figure S3/S3D/nitric acid_galactose_repeat3.jpg]

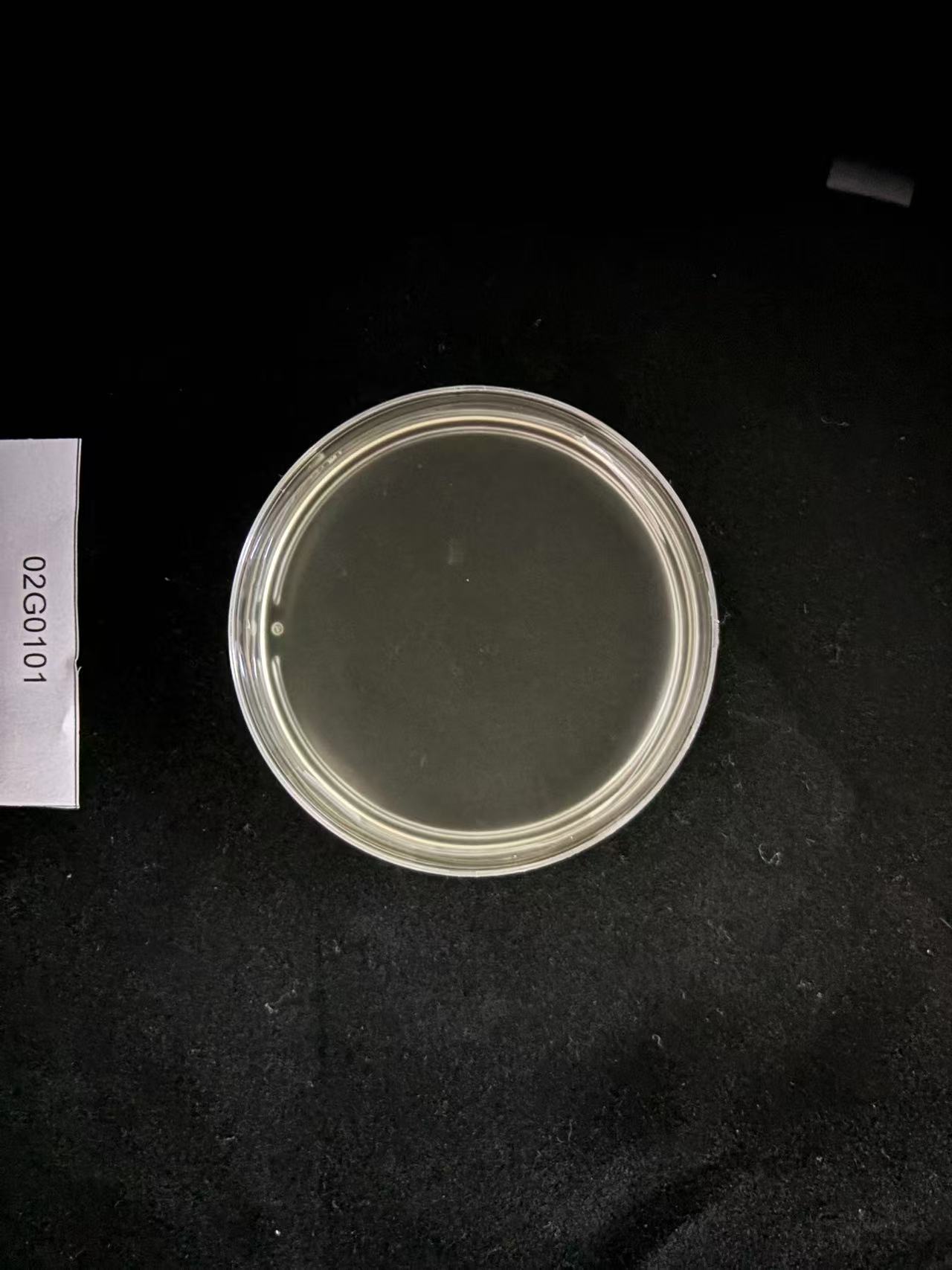

Supplement: Supplementary file 12 — Appendix Figure S3 Source Data [file 44319_2026_748_MOESM12_ESM.zip › Appendix Figure S3/S3D/Control_Repeat2.jpg]

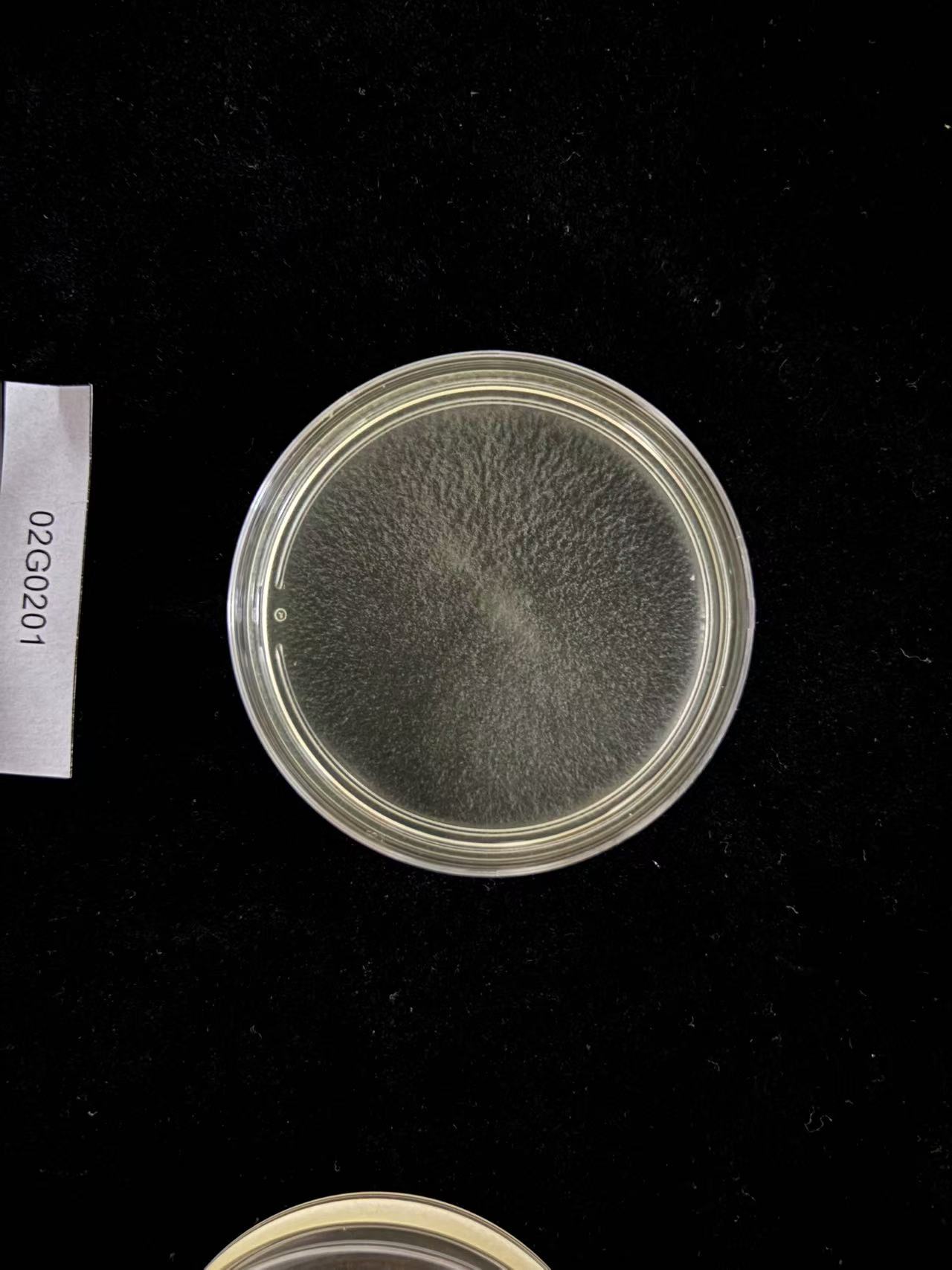

Supplement: Supplementary file 12 — Appendix Figure S3 Source Data [file 44319_2026_748_MOESM12_ESM.zip › Appendix Figure S3/S3D/nitric acid_repeat2.jpg]

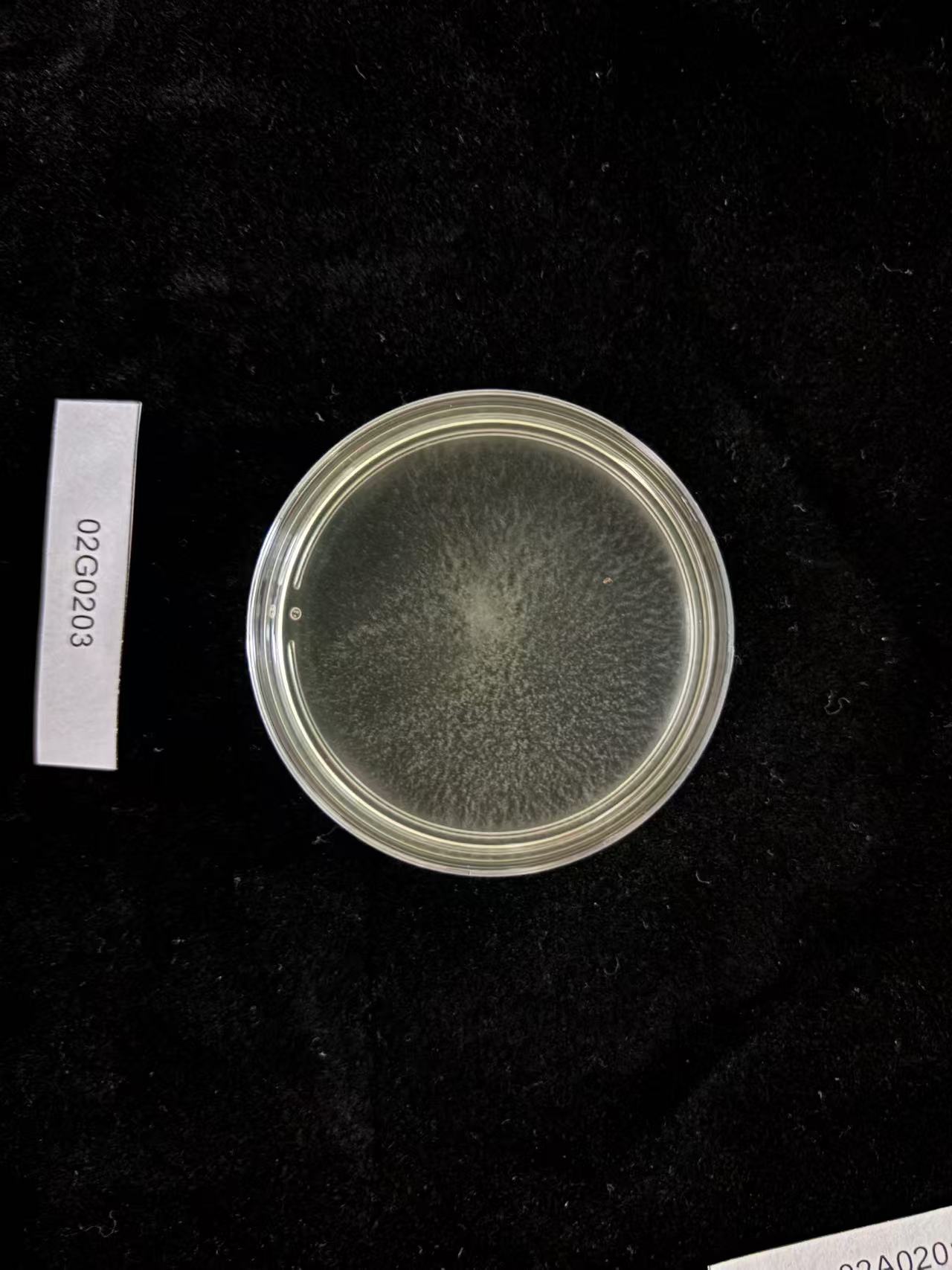

Supplement: Supplementary file 12 — Appendix Figure S3 Source Data [file 44319_2026_748_MOESM12_ESM.zip › Appendix Figure S3/S3D/nitric acid_repeat3.jpg]

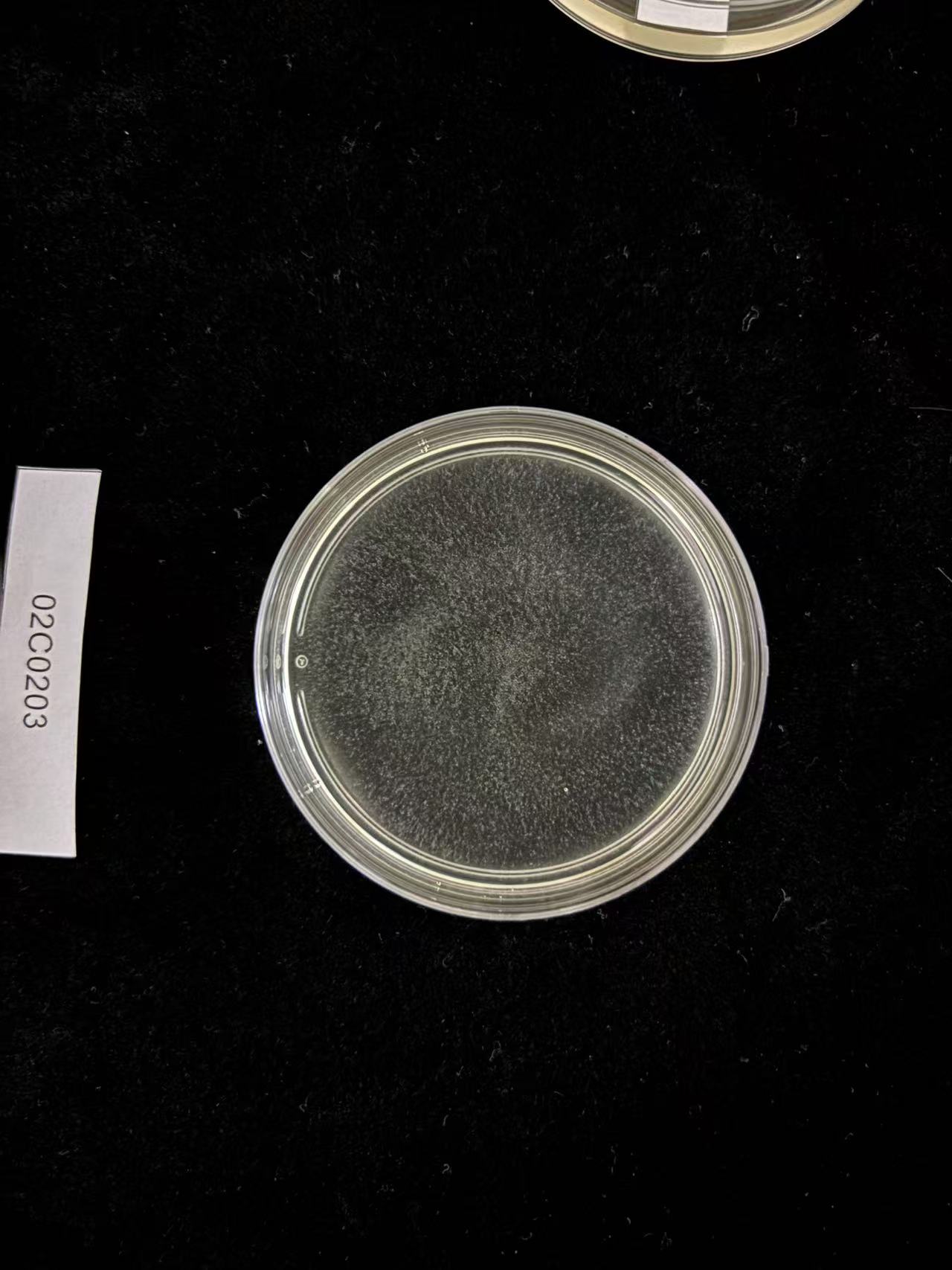

Supplement: Supplementary file 12 — Appendix Figure S3 Source Data [file 44319_2026_748_MOESM12_ESM.zip › Appendix Figure S3/S3B/hydrochloric_acid_Repeat3.jpg]

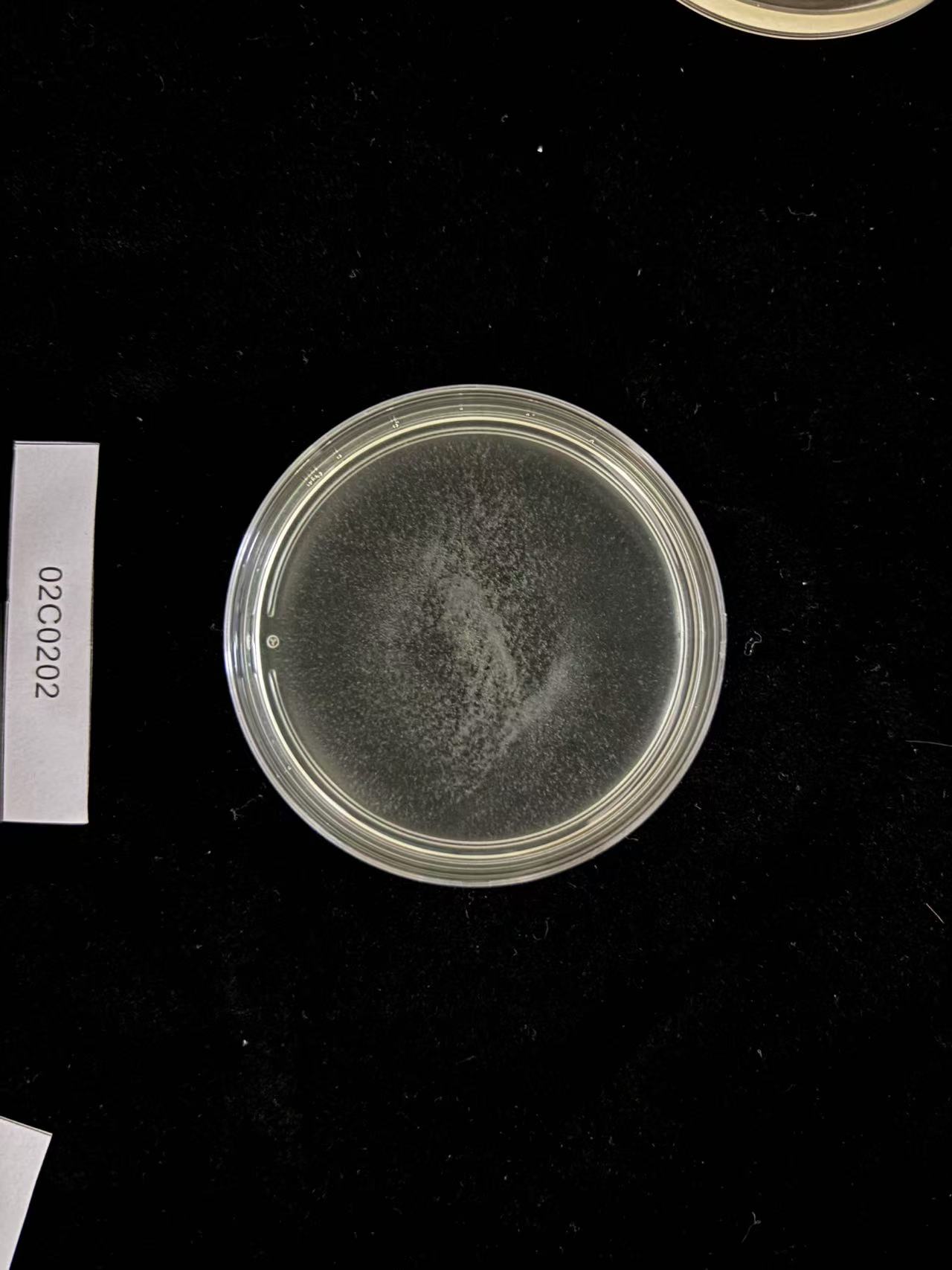

Supplement: Supplementary file 12 — Appendix Figure S3 Source Data [file 44319_2026_748_MOESM12_ESM.zip › Appendix Figure S3/S3B/hydrochloric_acid_Repeat2.jpg]

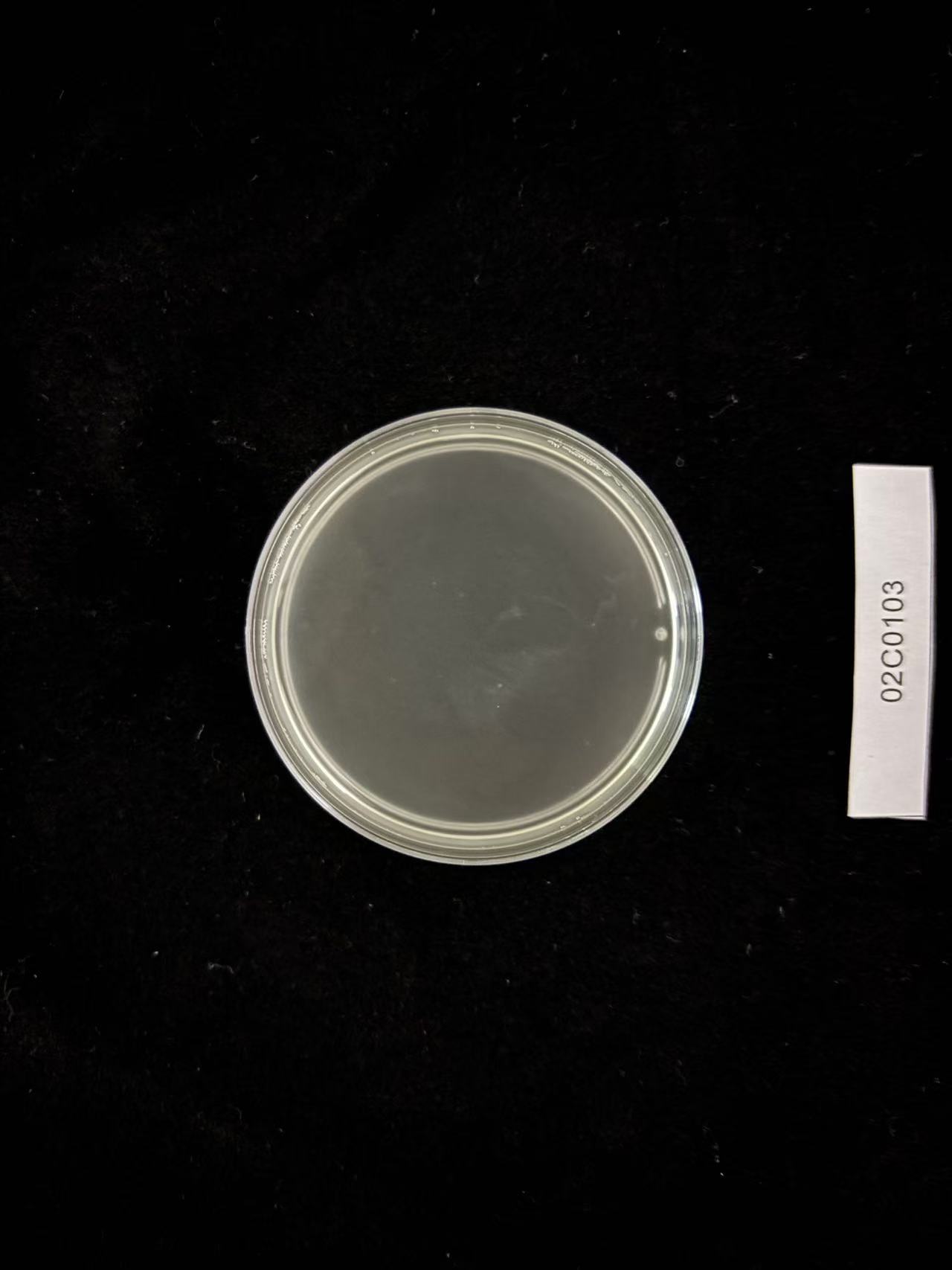

Supplement: Supplementary file 12 — Appendix Figure S3 Source Data [file 44319_2026_748_MOESM12_ESM.zip › Appendix Figure S3/S3B/Control_Repeat3.jpg]

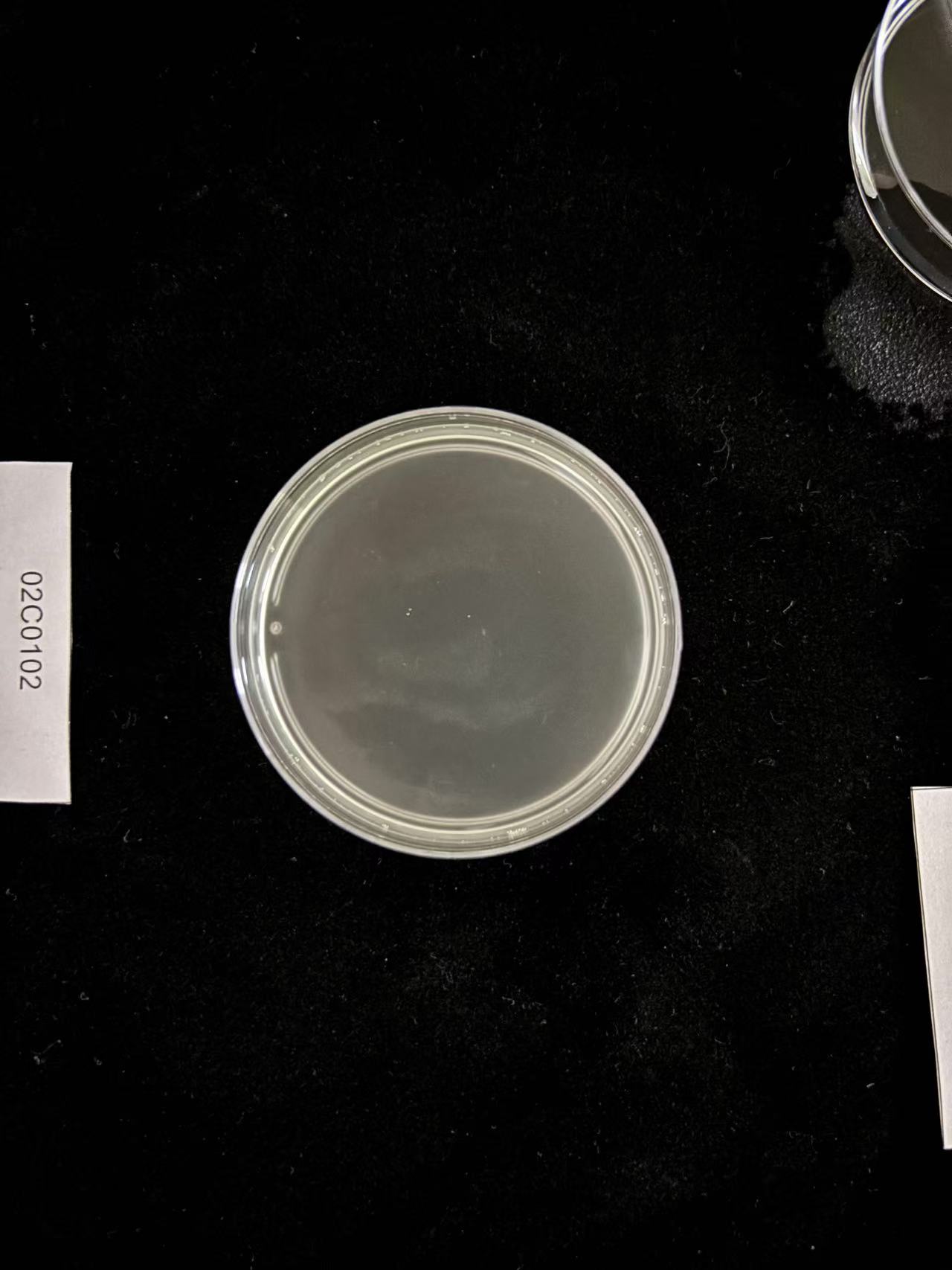

Supplement: Supplementary file 12 — Appendix Figure S3 Source Data [file 44319_2026_748_MOESM12_ESM.zip › Appendix Figure S3/S3B/Control_Repeat2.jpg]

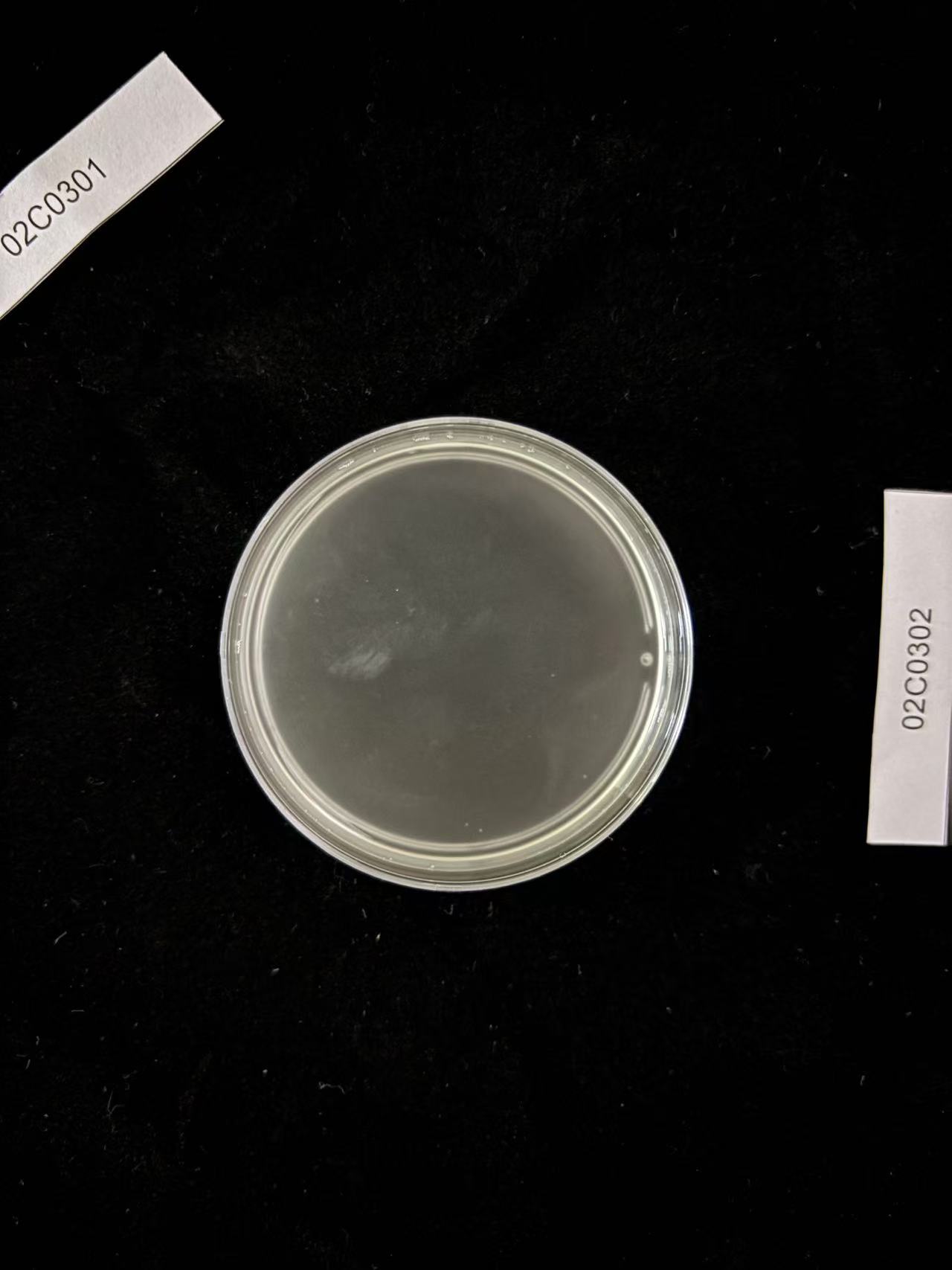

Supplement: Supplementary file 12 — Appendix Figure S3 Source Data [file 44319_2026_748_MOESM12_ESM.zip › Appendix Figure S3/S3B/hydrochloric_acid_galactose2.jpg]

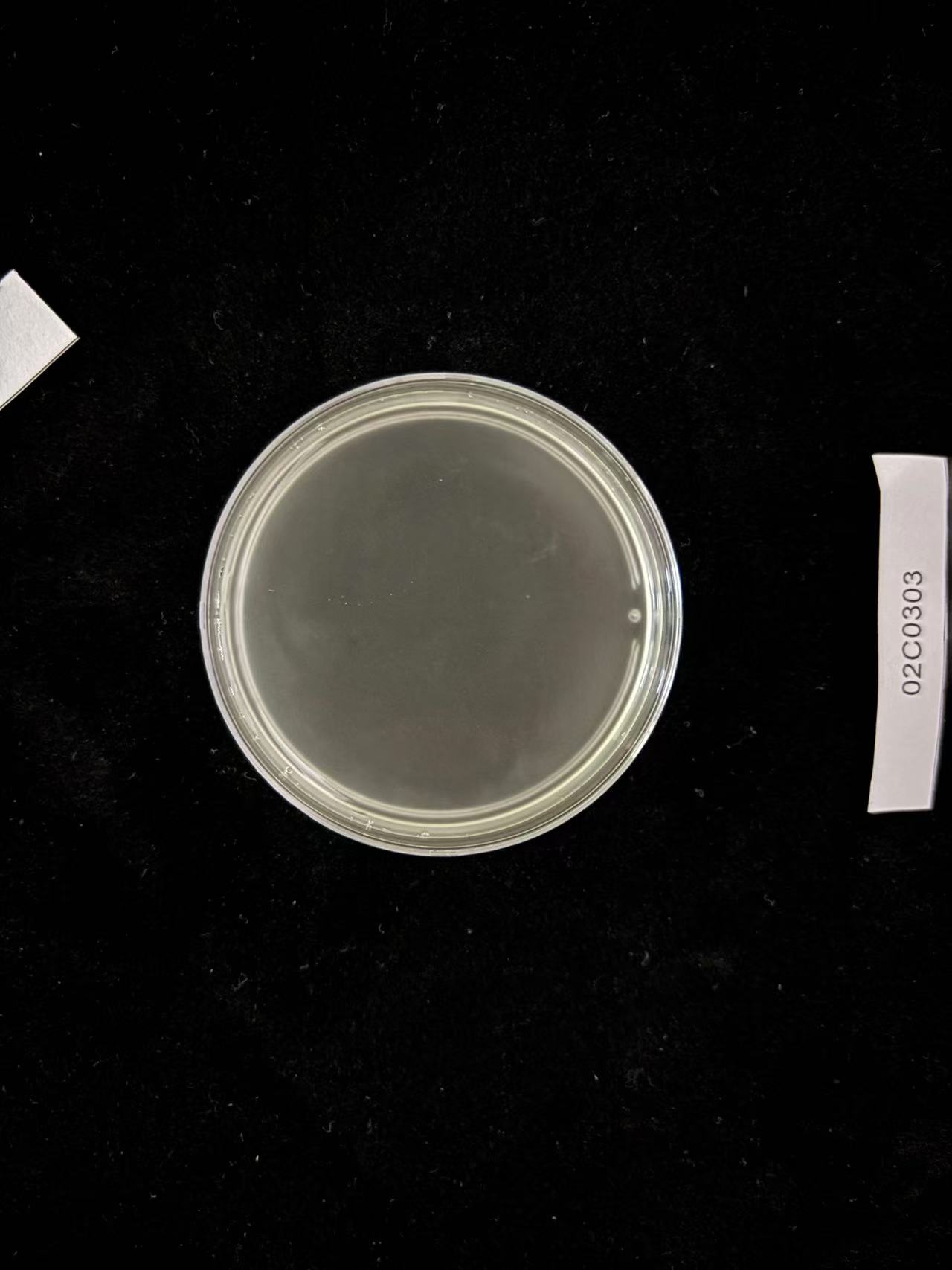

Supplement: Supplementary file 12 — Appendix Figure S3 Source Data [file 44319_2026_748_MOESM12_ESM.zip › Appendix Figure S3/S3B/hydrochloric_acid_galactose3.jpg]

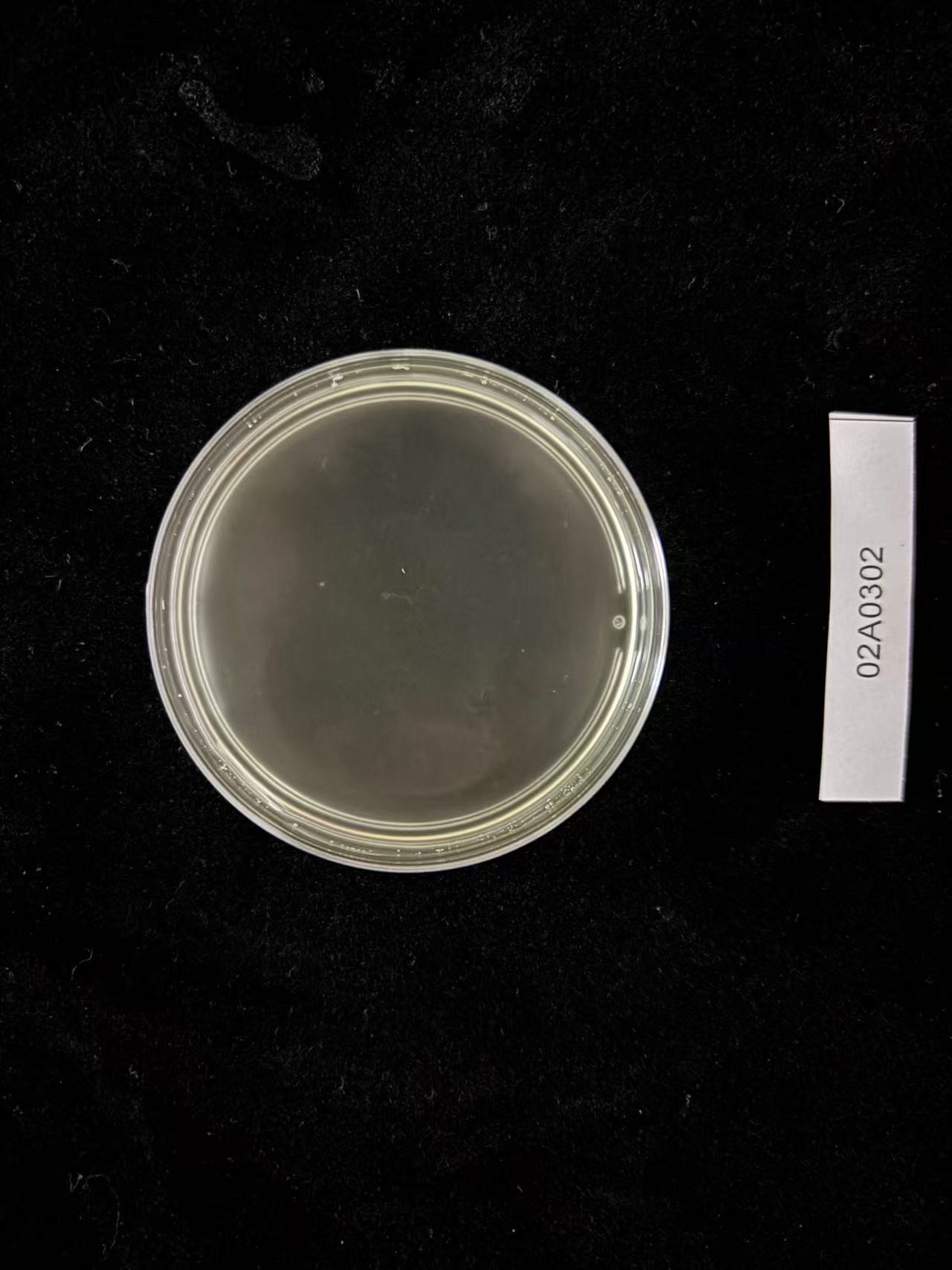

Supplement: Supplementary file 12 — Appendix Figure S3 Source Data [file 44319_2026_748_MOESM12_ESM.zip › Appendix Figure S3/S3A/acetic acid_galactose_repeat2.jpg]

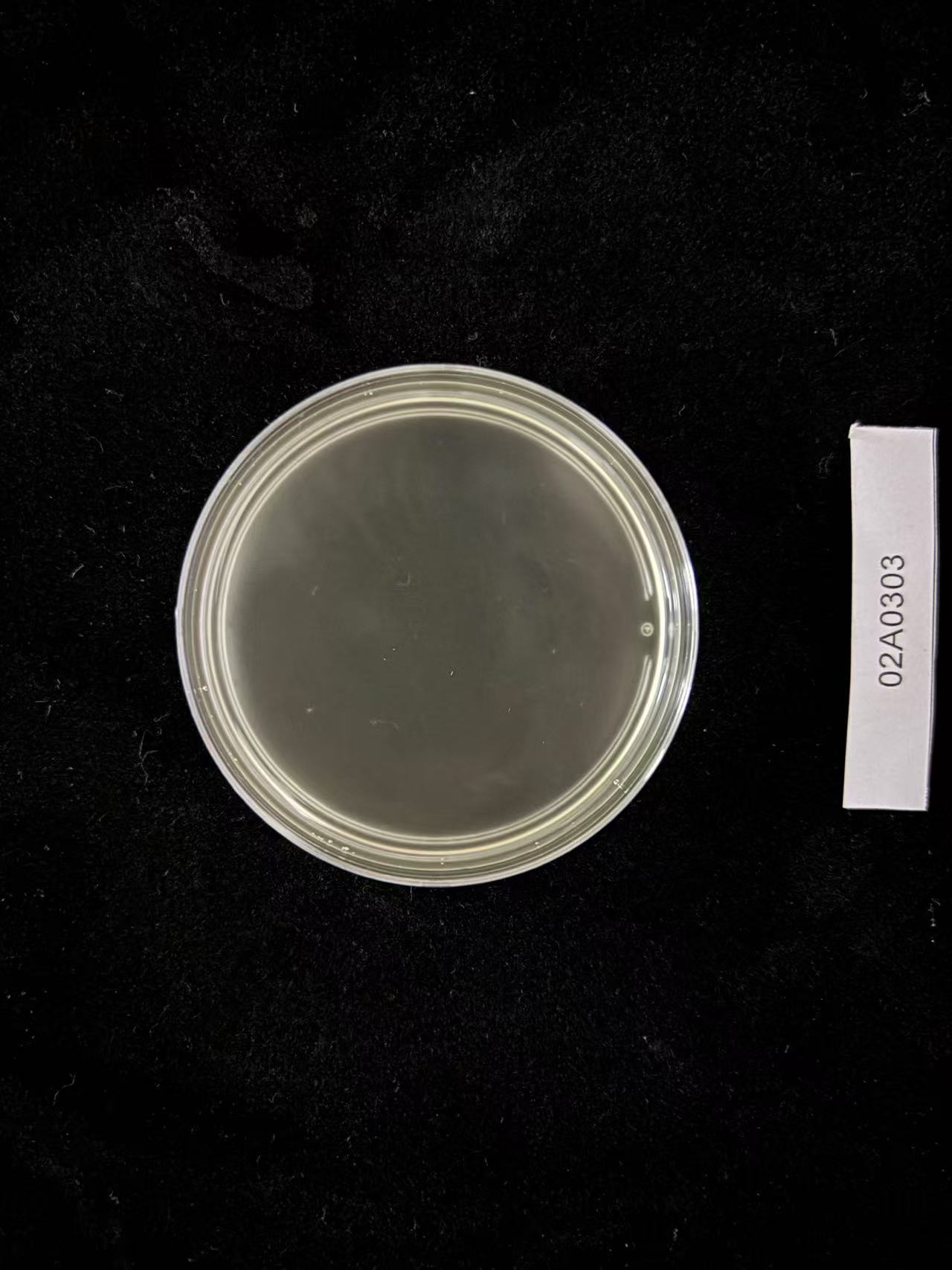

Supplement: Supplementary file 12 — Appendix Figure S3 Source Data [file 44319_2026_748_MOESM12_ESM.zip › Appendix Figure S3/S3A/acetic acid_galactose_repeat3.jpg]

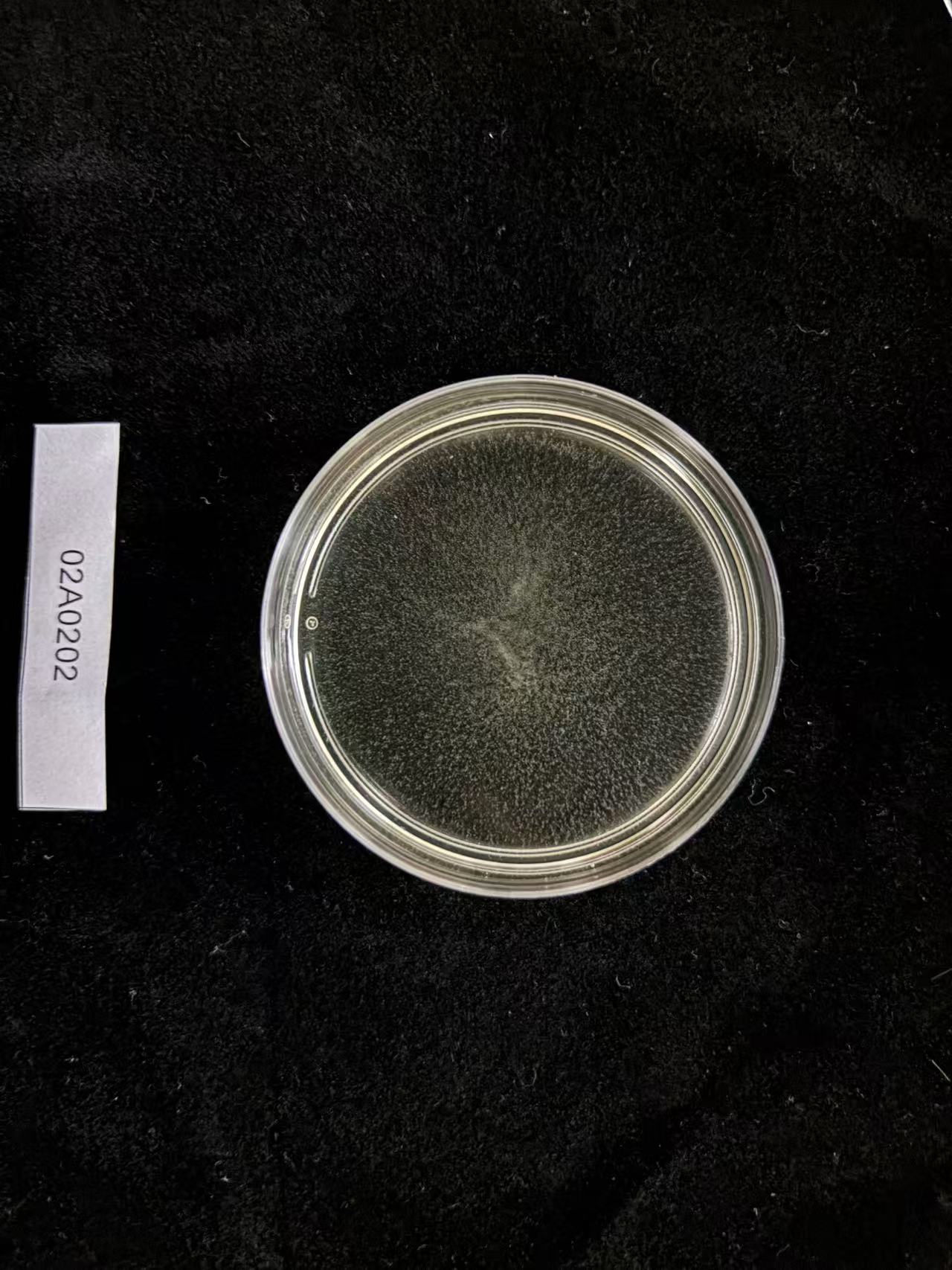

Supplement: Supplementary file 12 — Appendix Figure S3 Source Data [file 44319_2026_748_MOESM12_ESM.zip › Appendix Figure S3/S3A/acetic acid_Repeat2.jpg]

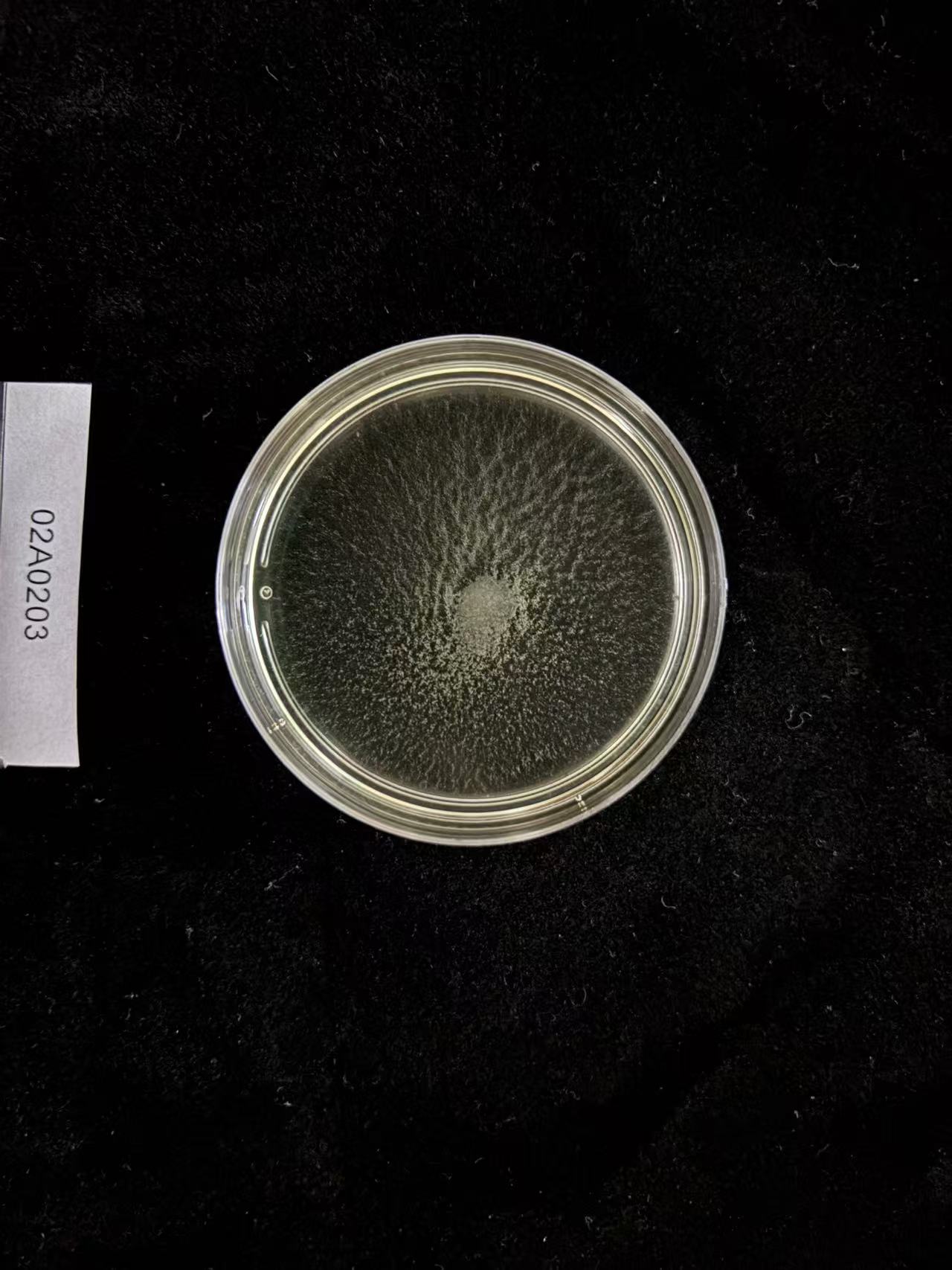

Supplement: Supplementary file 12 — Appendix Figure S3 Source Data [file 44319_2026_748_MOESM12_ESM.zip › Appendix Figure S3/S3A/acetic acid_Repeat3.jpg]

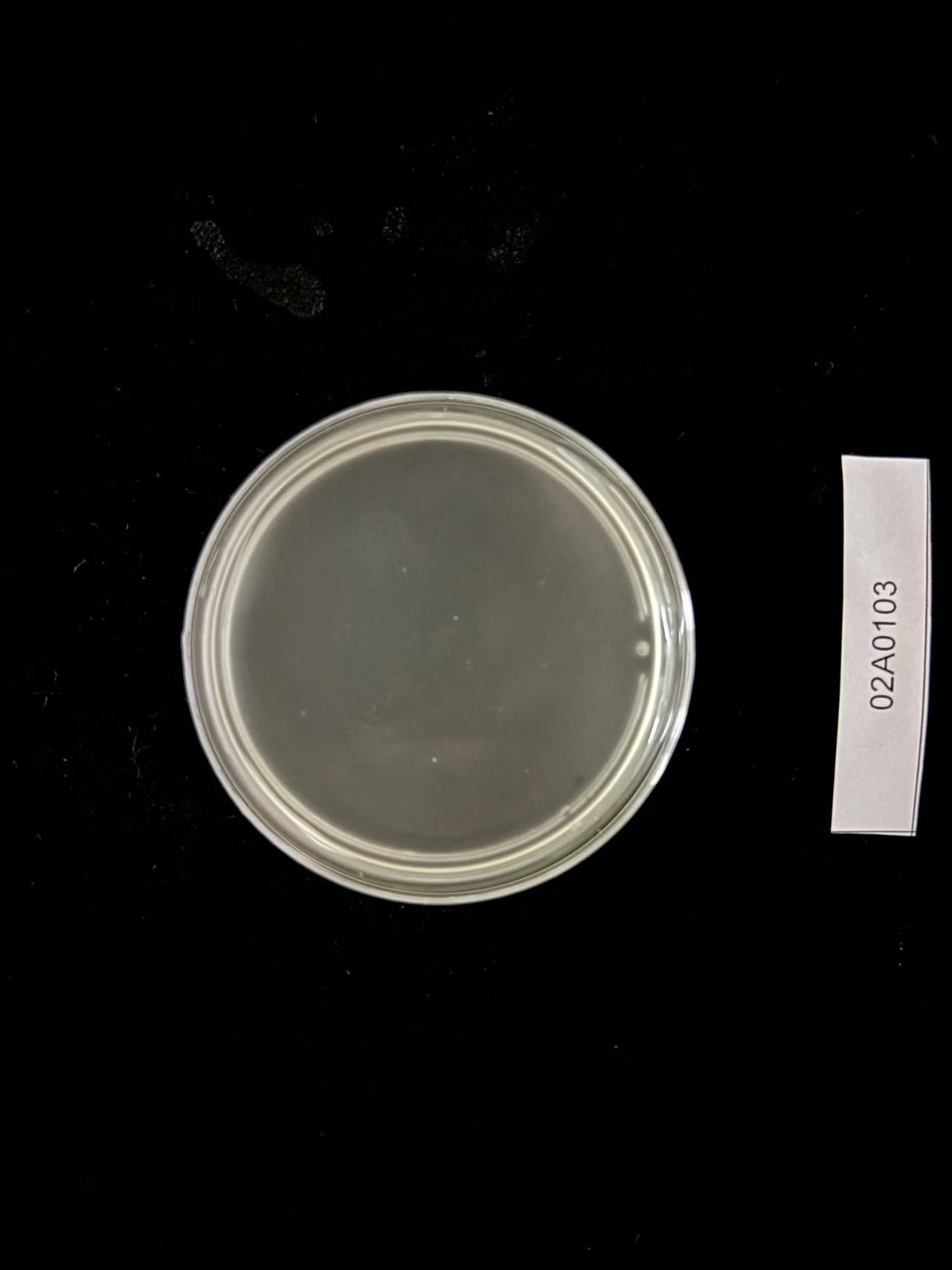

Supplement: Supplementary file 12 — Appendix Figure S3 Source Data [file 44319_2026_748_MOESM12_ESM.zip › Appendix Figure S3/S3A/Control_Repeat3.jpg]

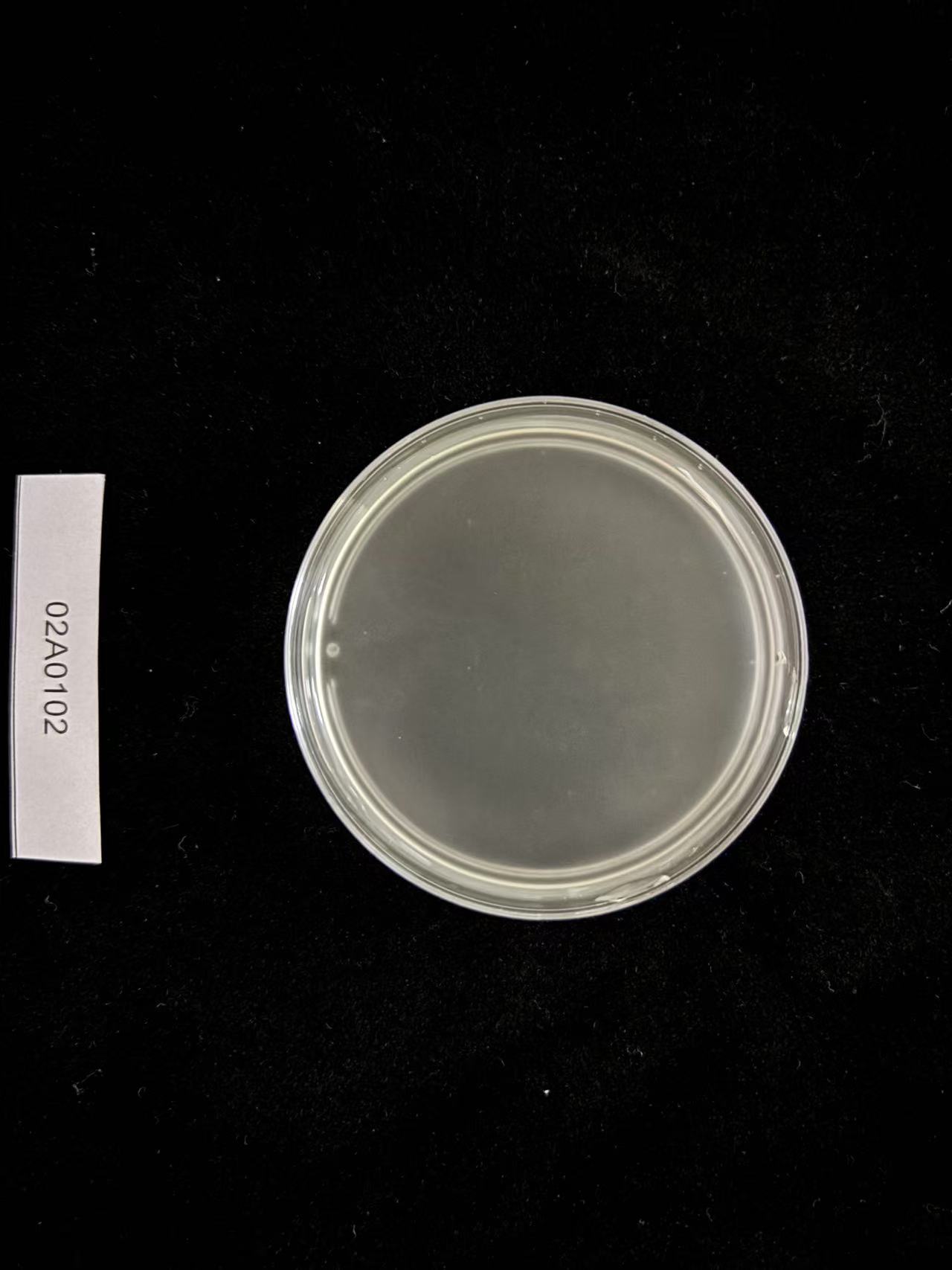

Supplement: Supplementary file 12 — Appendix Figure S3 Source Data [file 44319_2026_748_MOESM12_ESM.zip › Appendix Figure S3/S3A/Control_Repeat2.jpg]

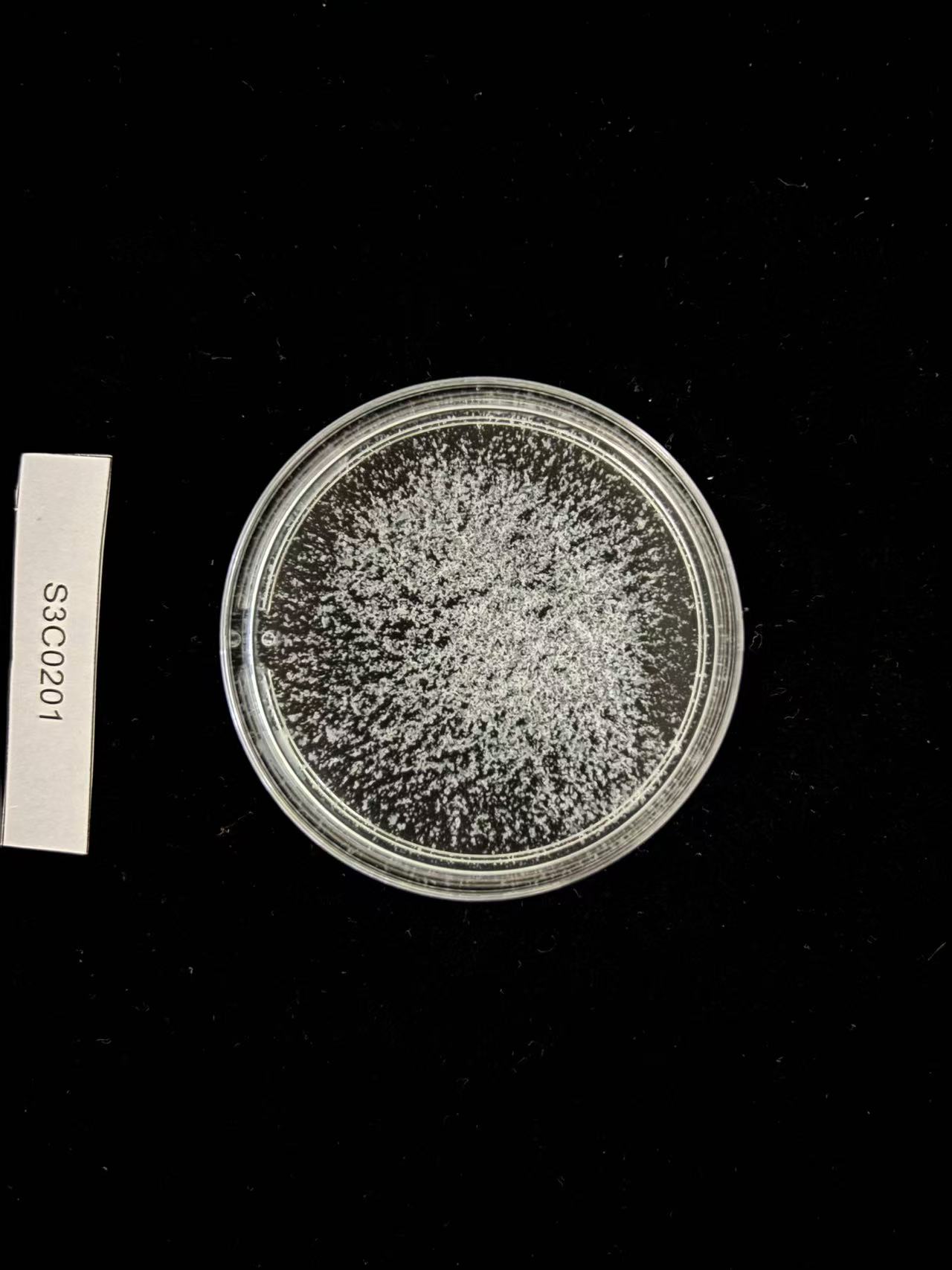

Supplement: Supplementary file 13 — Appendix Figure S4 Source Data [file 44319_2026_748_MOESM13_ESM.zip › Appendix Figure S4/S4C/gsf2IE_Repeat 1.jpg]

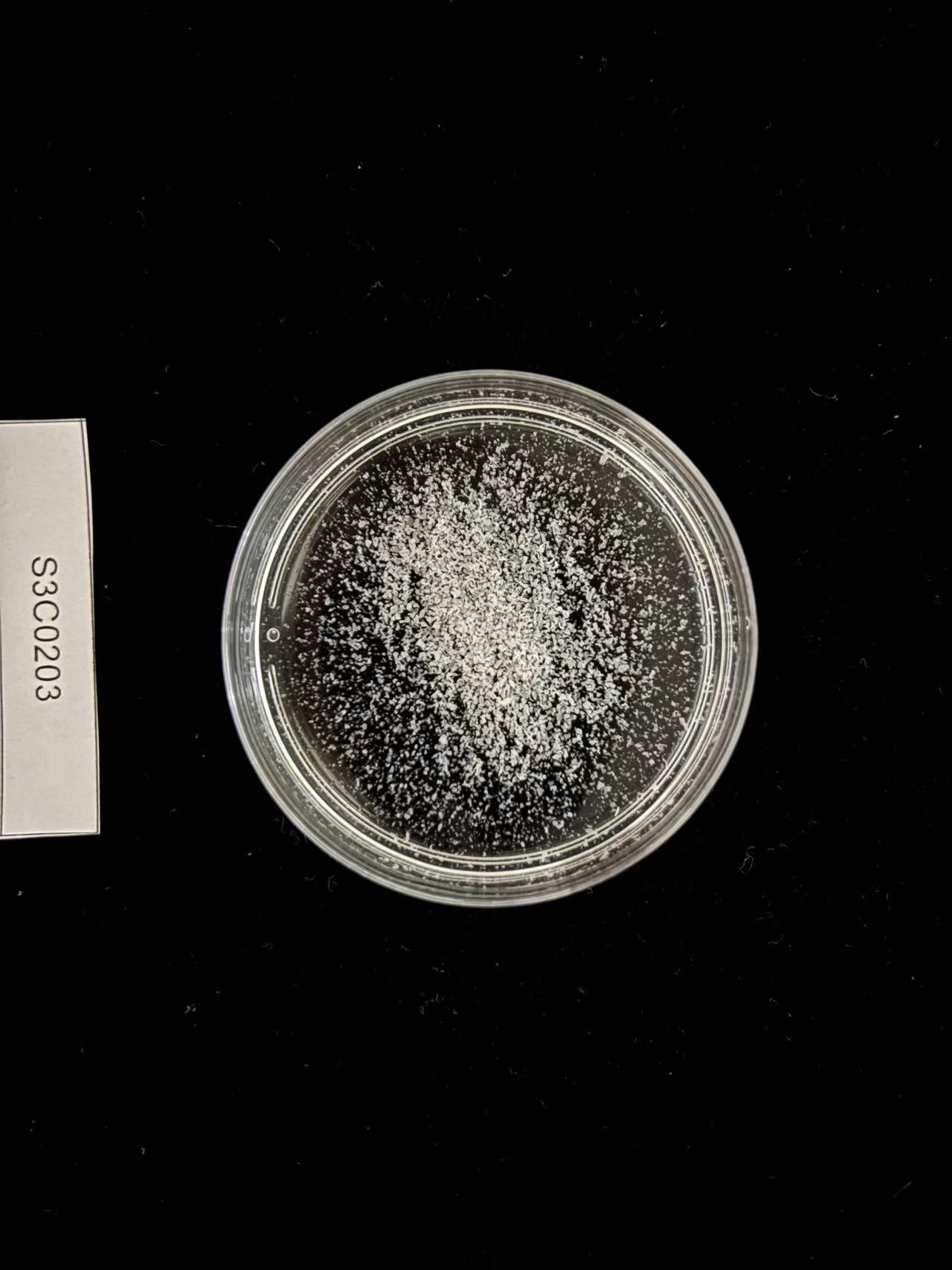

Supplement: Supplementary file 13 — Appendix Figure S4 Source Data [file 44319_2026_748_MOESM13_ESM.zip › Appendix Figure S4/S4C/gsf2IE_Repeat 3.jpg]

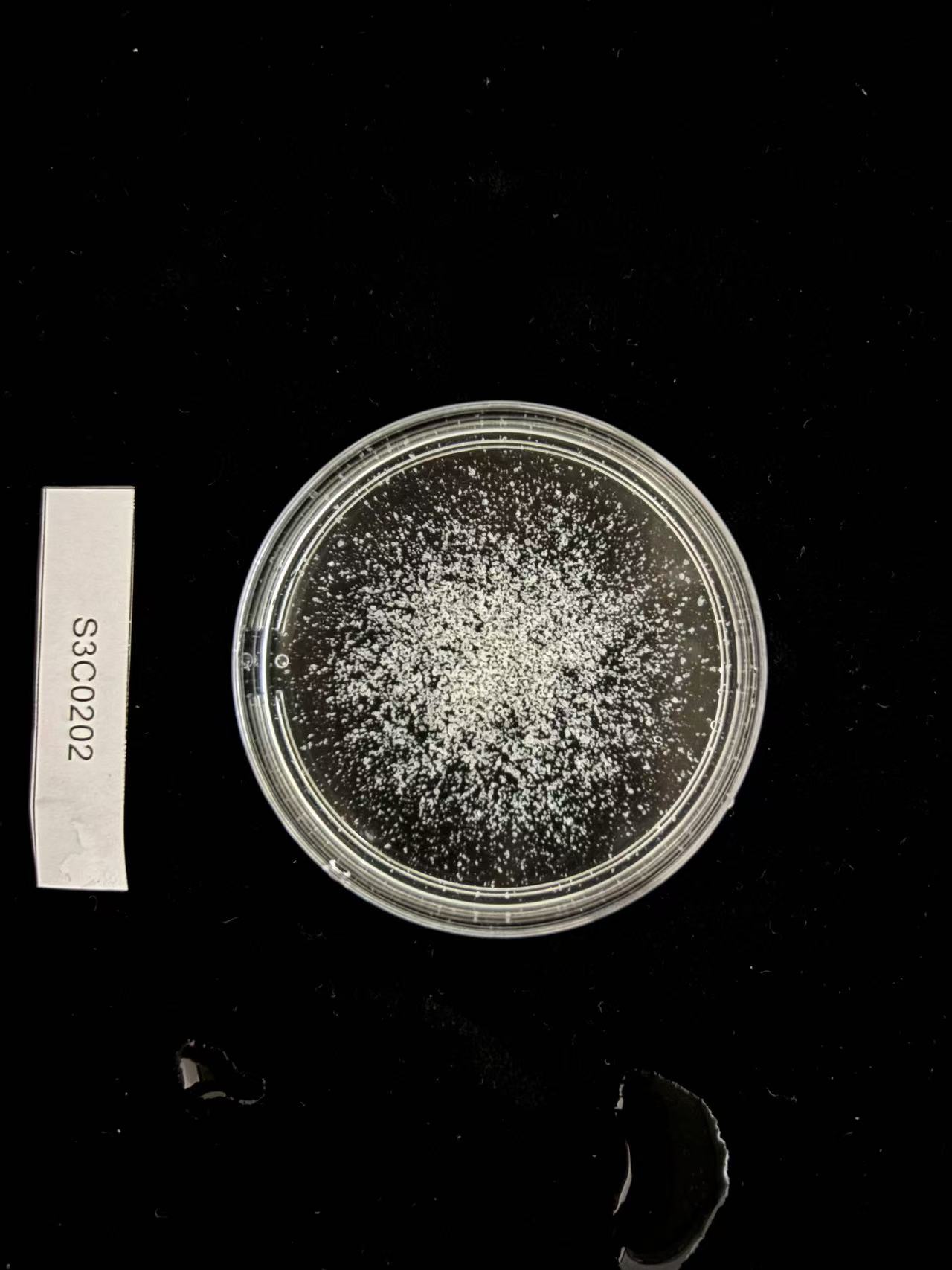

Supplement: Supplementary file 13 — Appendix Figure S4 Source Data [file 44319_2026_748_MOESM13_ESM.zip › Appendix Figure S4/S4C/gsf2IE_Repeat 2.jpg]

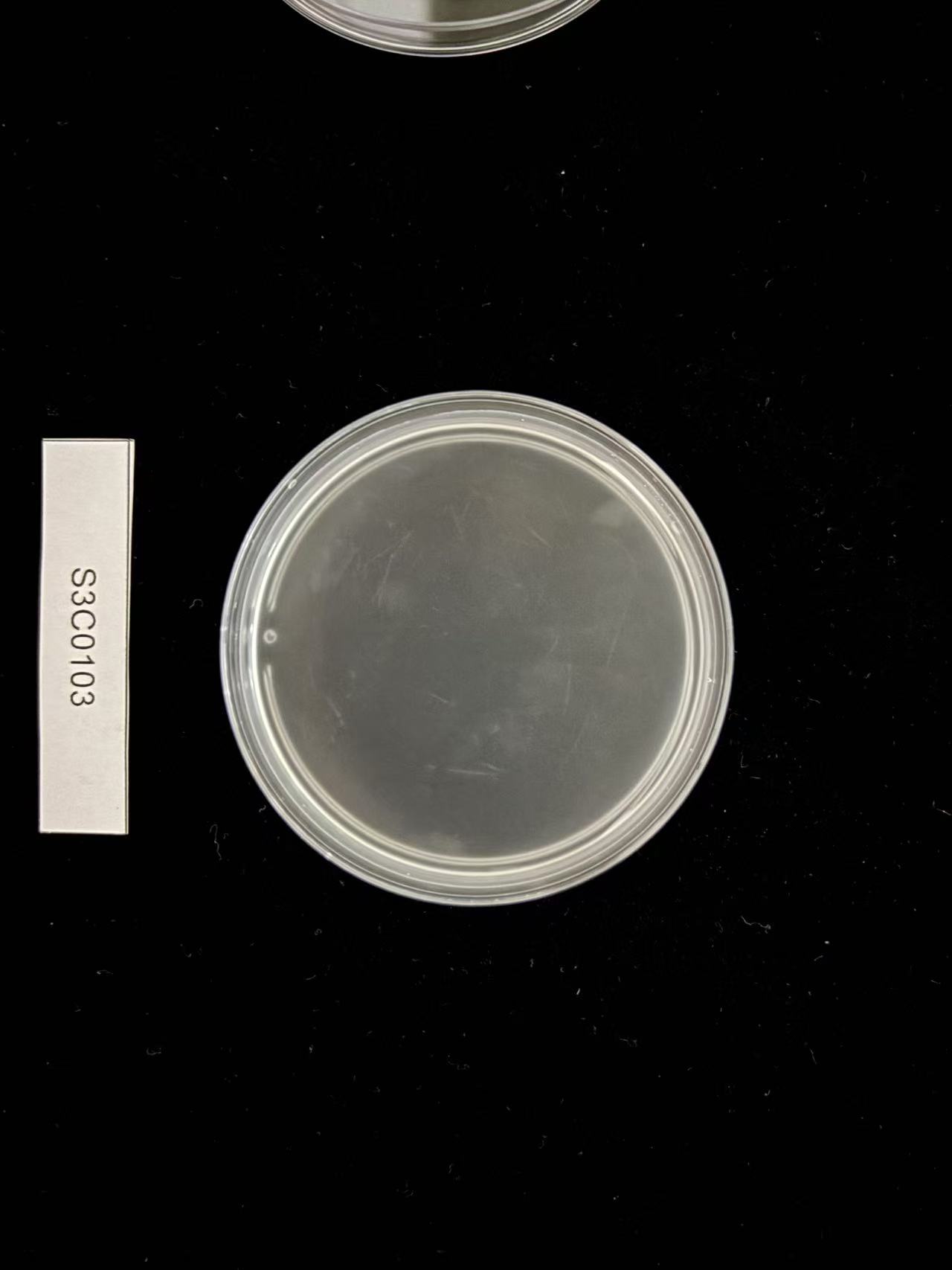

Supplement: Supplementary file 13 — Appendix Figure S4 Source Data [file 44319_2026_748_MOESM13_ESM.zip › Appendix Figure S4/S4C/gsf2Γêå_Repeat 3.jpg]

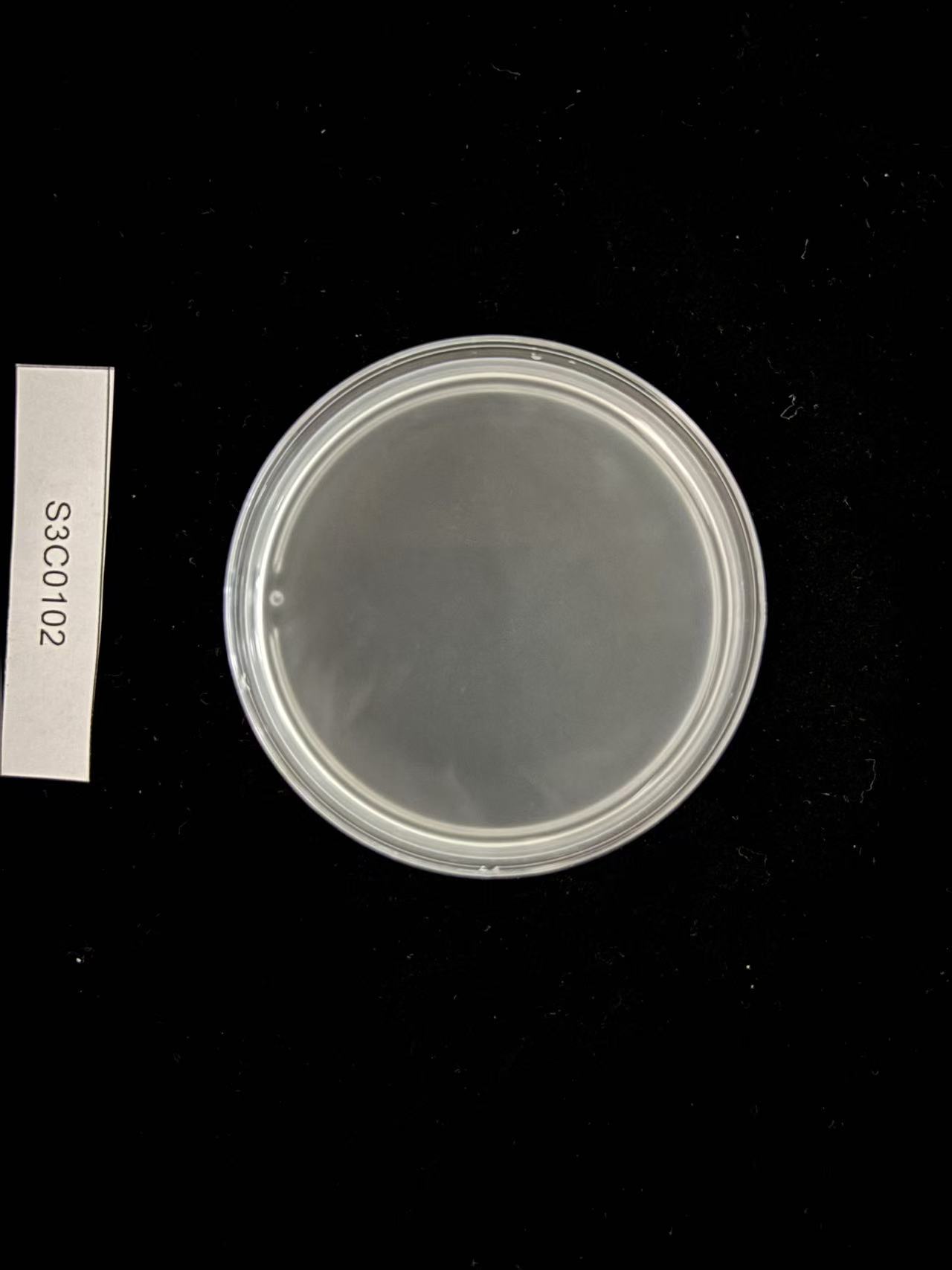

Supplement: Supplementary file 13 — Appendix Figure S4 Source Data [file 44319_2026_748_MOESM13_ESM.zip › Appendix Figure S4/S4C/gsf2Γêå_Repeat 2.jpg]

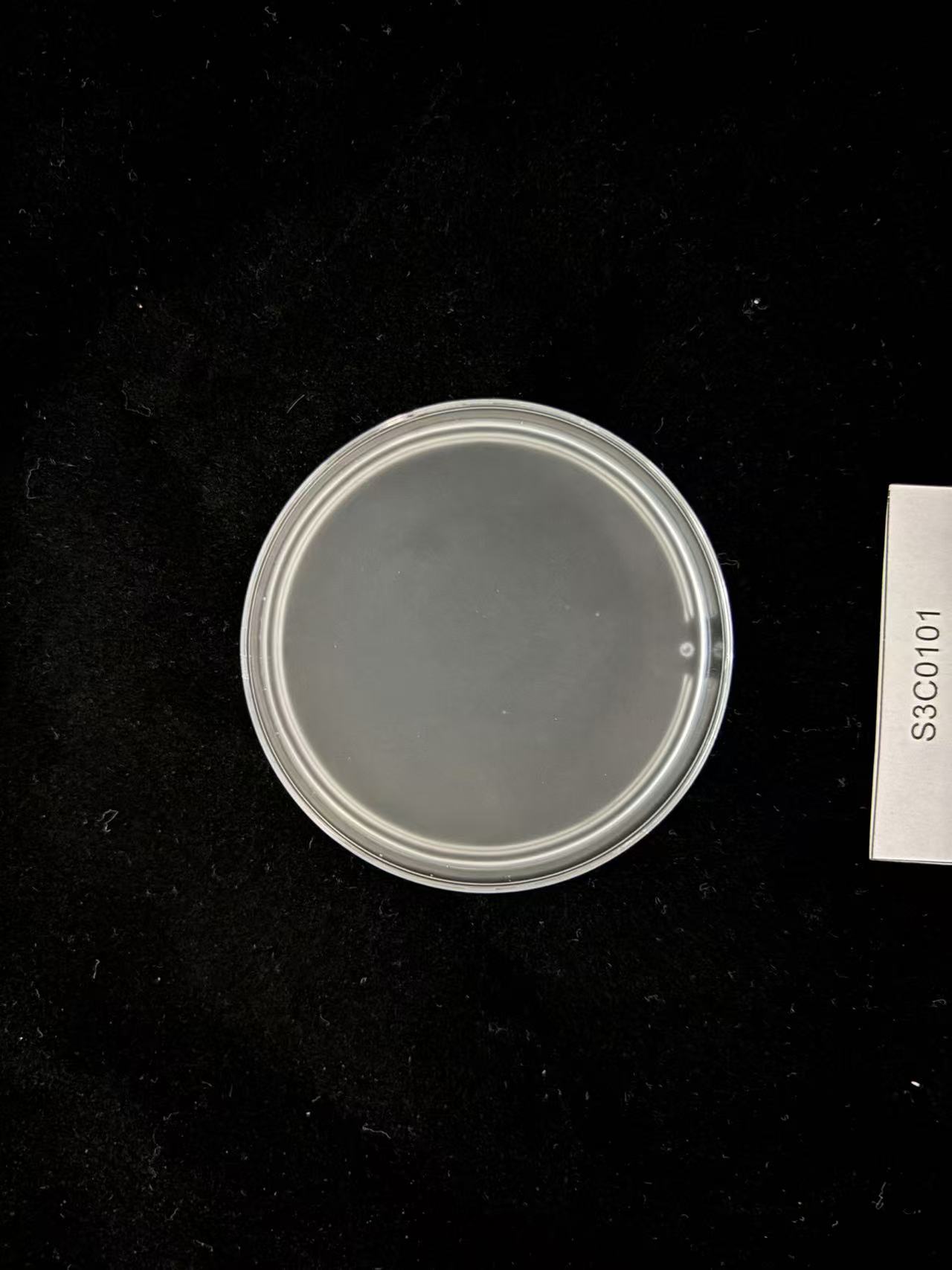

Supplement: Supplementary file 13 — Appendix Figure S4 Source Data [file 44319_2026_748_MOESM13_ESM.zip › Appendix Figure S4/S4C/gsf2Γêå_Repeat 1.jpg]

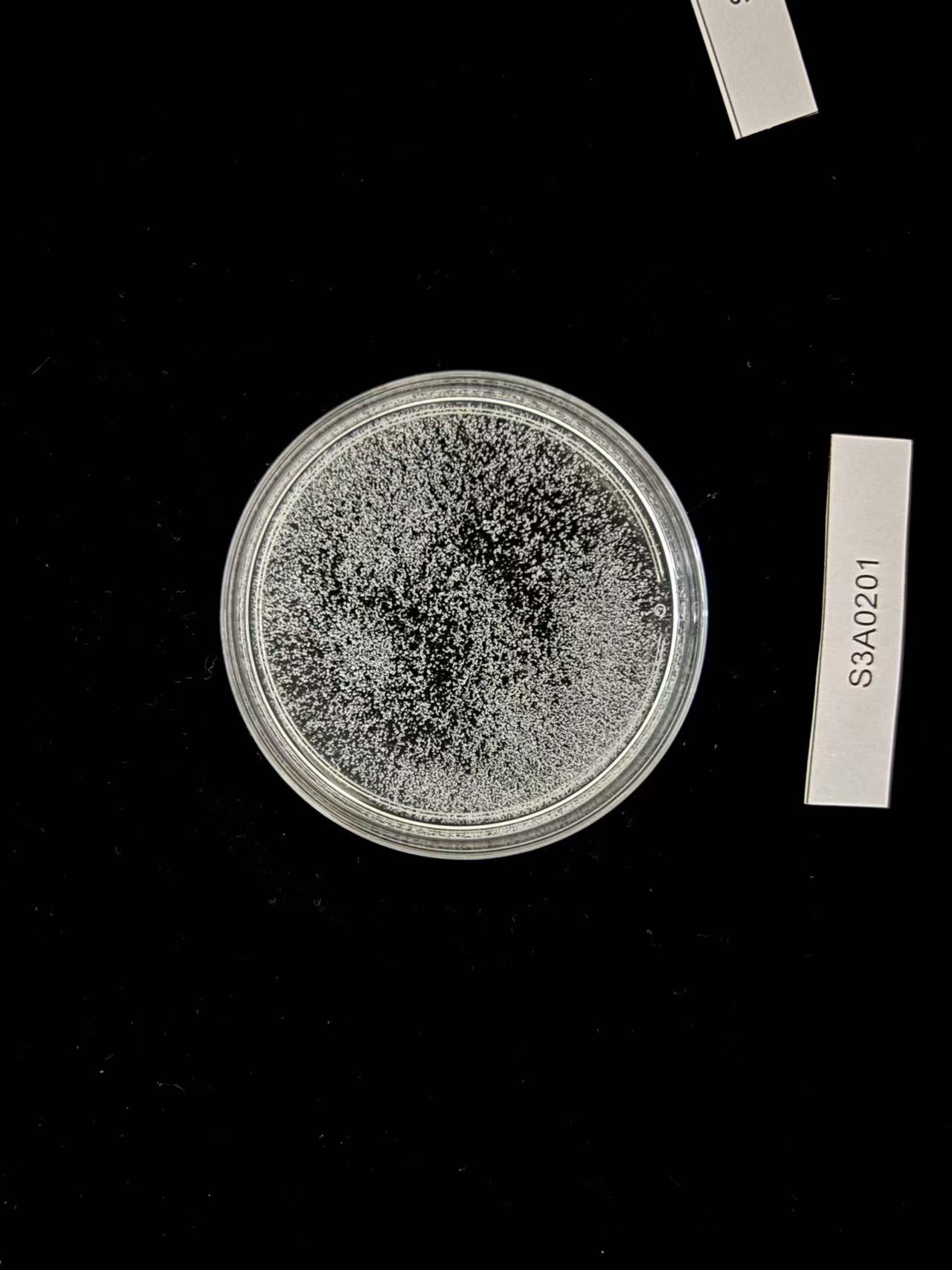

Supplement: Supplementary file 13 — Appendix Figure S4 Source Data [file 44319_2026_748_MOESM13_ESM.zip › Appendix Figure S4/S4A/gsf2IE_Repeat 1.jpg]

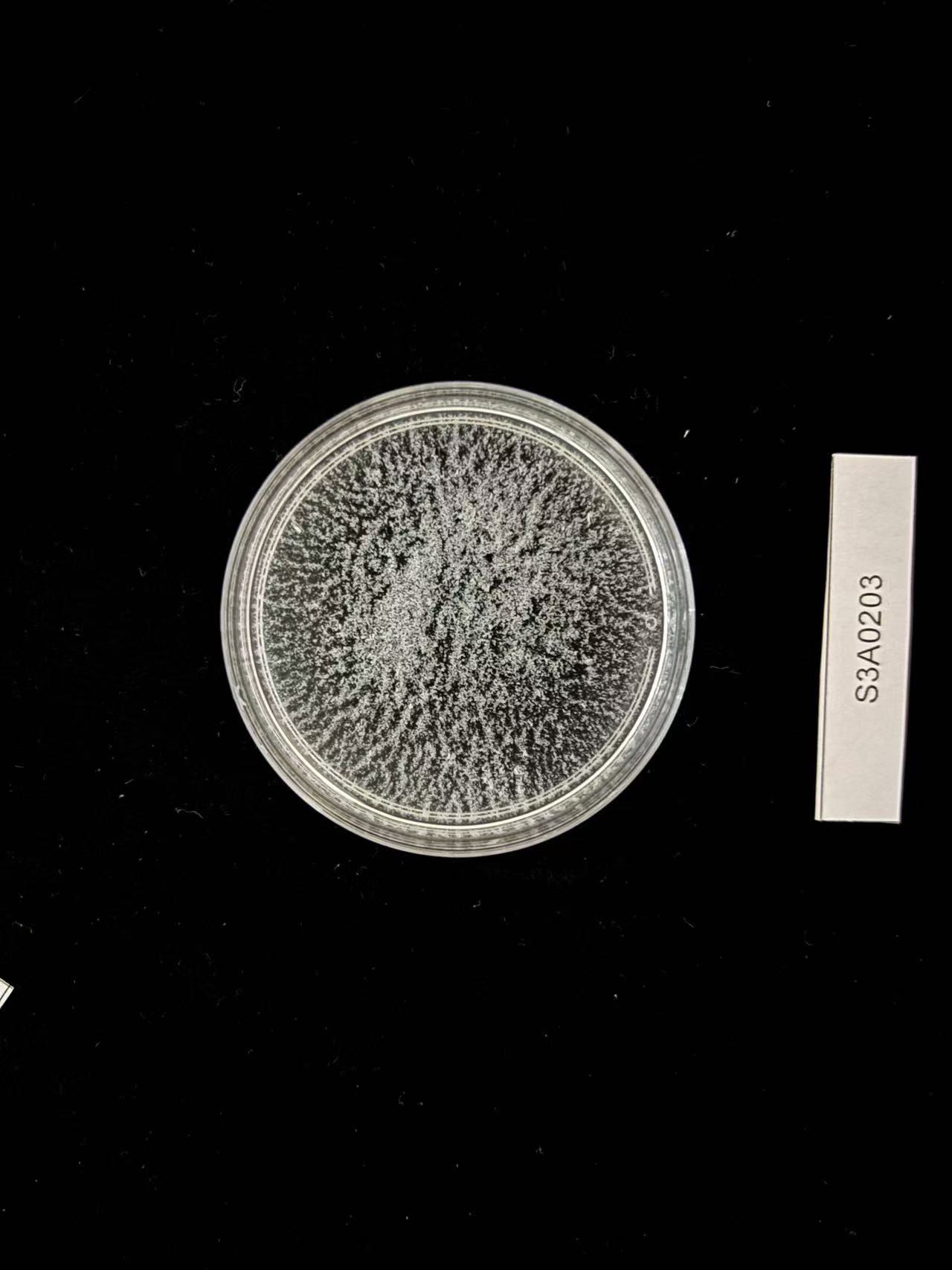

Supplement: Supplementary file 13 — Appendix Figure S4 Source Data [file 44319_2026_748_MOESM13_ESM.zip › Appendix Figure S4/S4A/gsf2IE_Repeat 3.jpg]

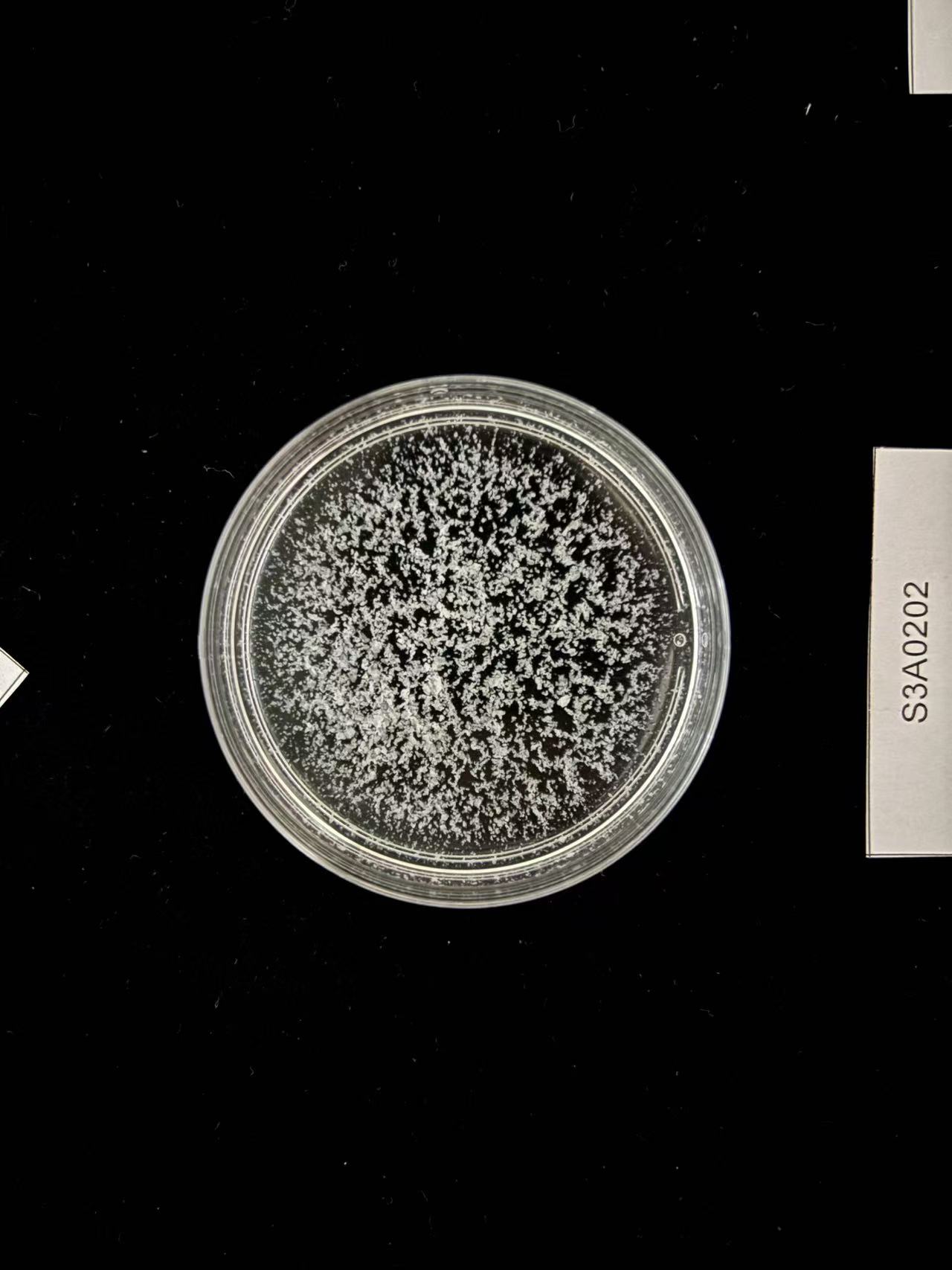

Supplement: Supplementary file 13 — Appendix Figure S4 Source Data [file 44319_2026_748_MOESM13_ESM.zip › Appendix Figure S4/S4A/gsf2IE_Repeat 2.jpg]

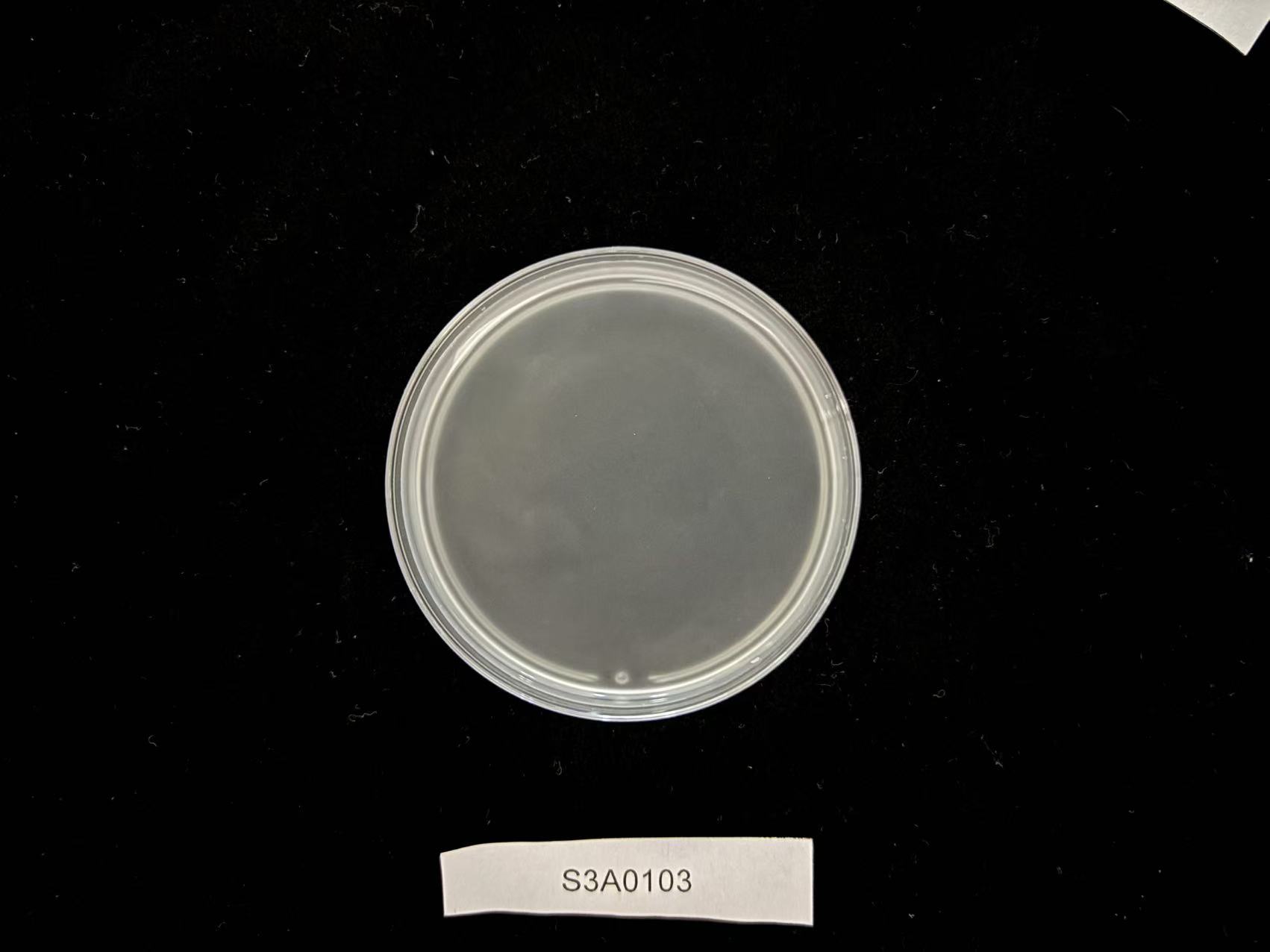

Supplement: Supplementary file 13 — Appendix Figure S4 Source Data [file 44319_2026_748_MOESM13_ESM.zip › Appendix Figure S4/S4A/gsf2Γêå_Repeat 3.jpg]

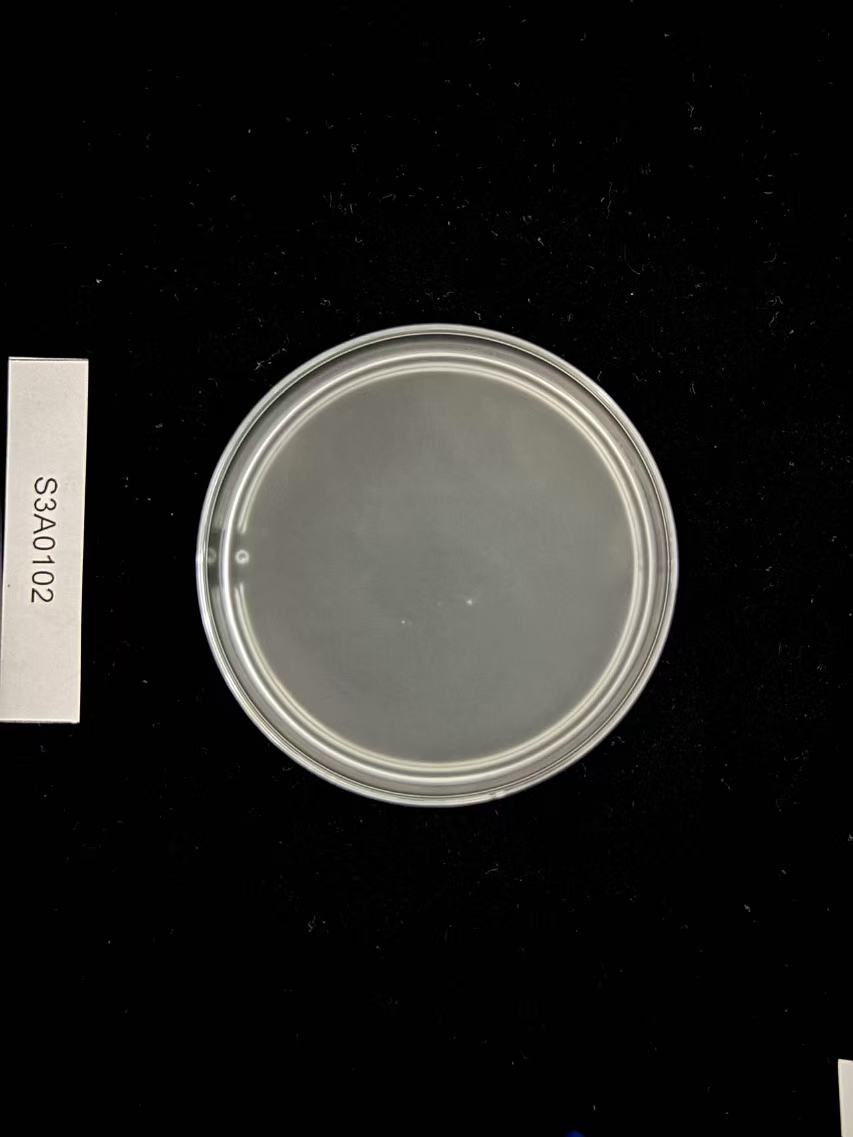

Supplement: Supplementary file 13 — Appendix Figure S4 Source Data [file 44319_2026_748_MOESM13_ESM.zip › Appendix Figure S4/S4A/gsf2Γêå_Repeat 2.jpg]

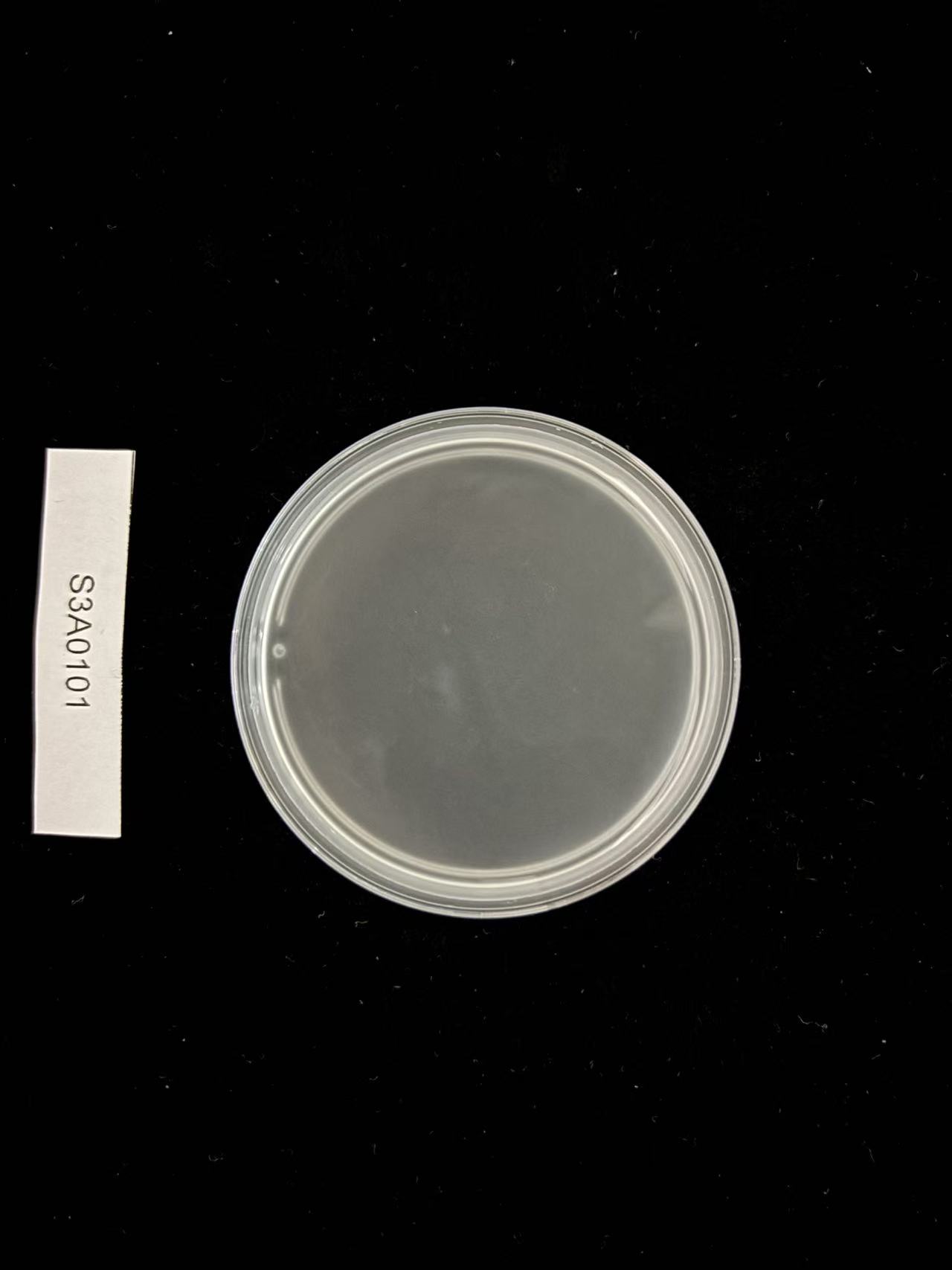

Supplement: Supplementary file 13 — Appendix Figure S4 Source Data [file 44319_2026_748_MOESM13_ESM.zip › Appendix Figure S4/S4A/gsf2Γêå_Repeat 1.jpg]

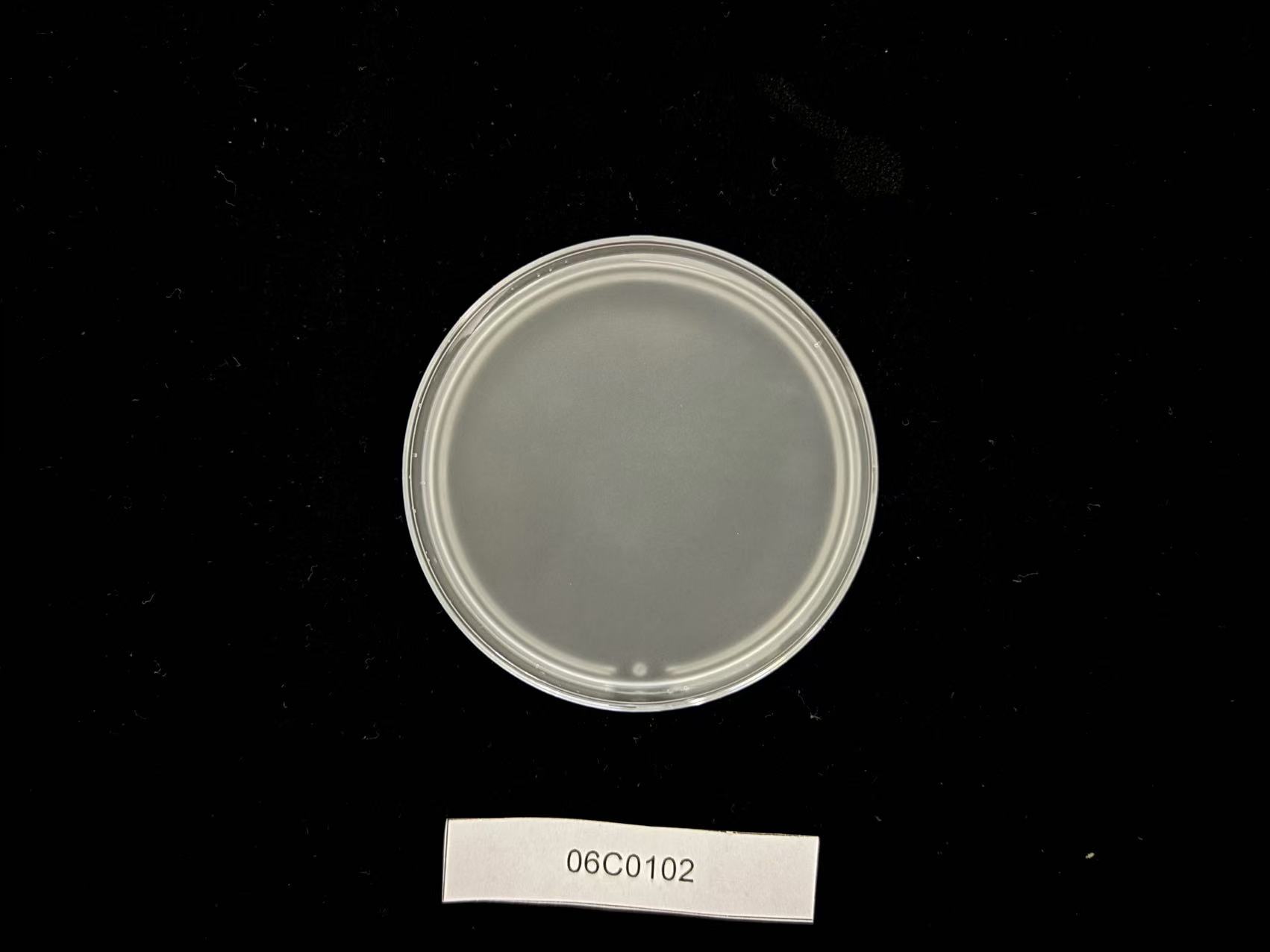

Supplement: Supplementary file 14 — Appendix Figure S7 Source Data [file 44319_2026_748_MOESM14_ESM.zip › Appendix Figure S7/S7B/gsf2AΓêå_Repeat2.jpg]

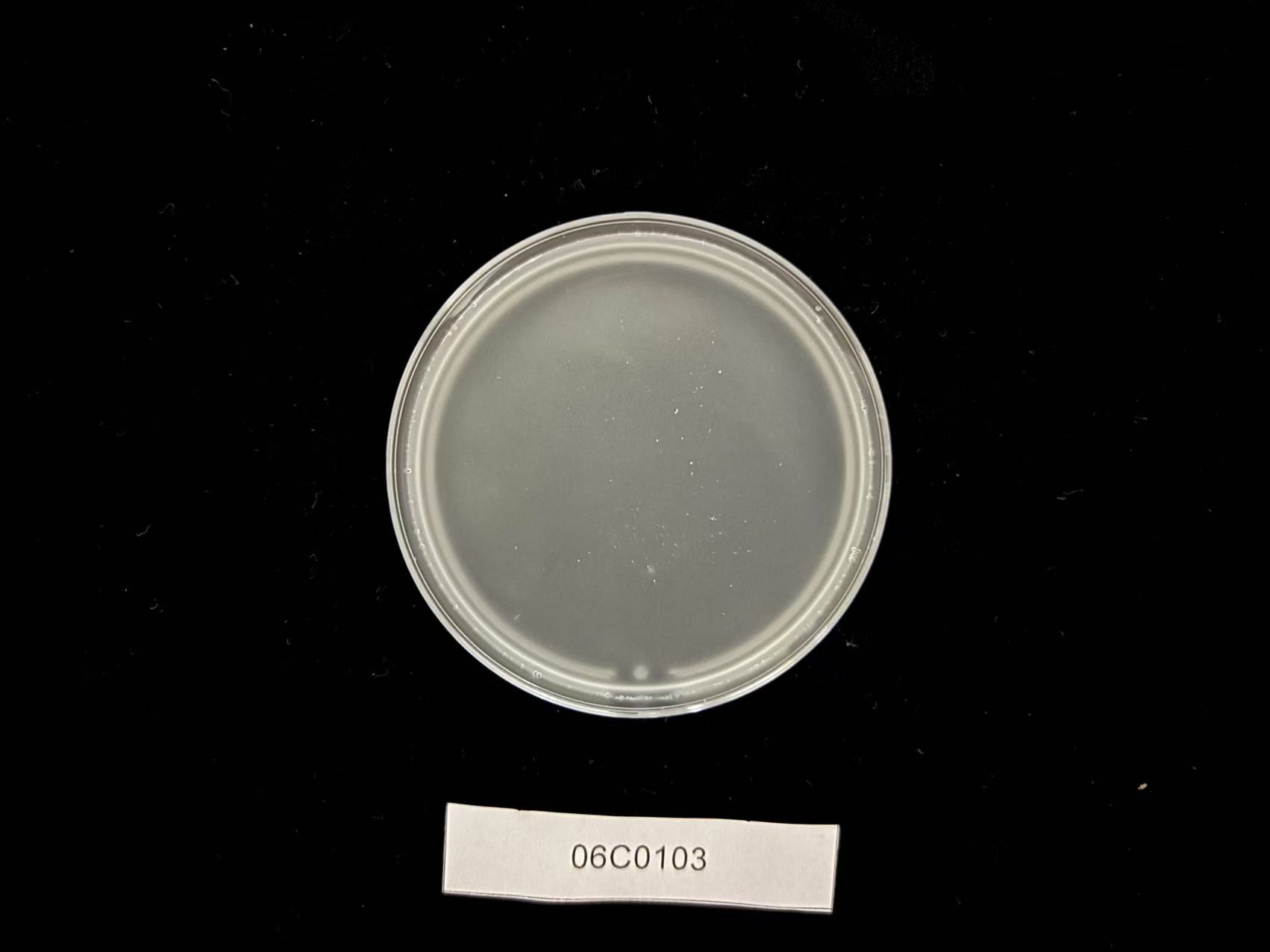

Supplement: Supplementary file 14 — Appendix Figure S7 Source Data [file 44319_2026_748_MOESM14_ESM.zip › Appendix Figure S7/S7B/gsf2AΓêå_Repeat3.jpg]

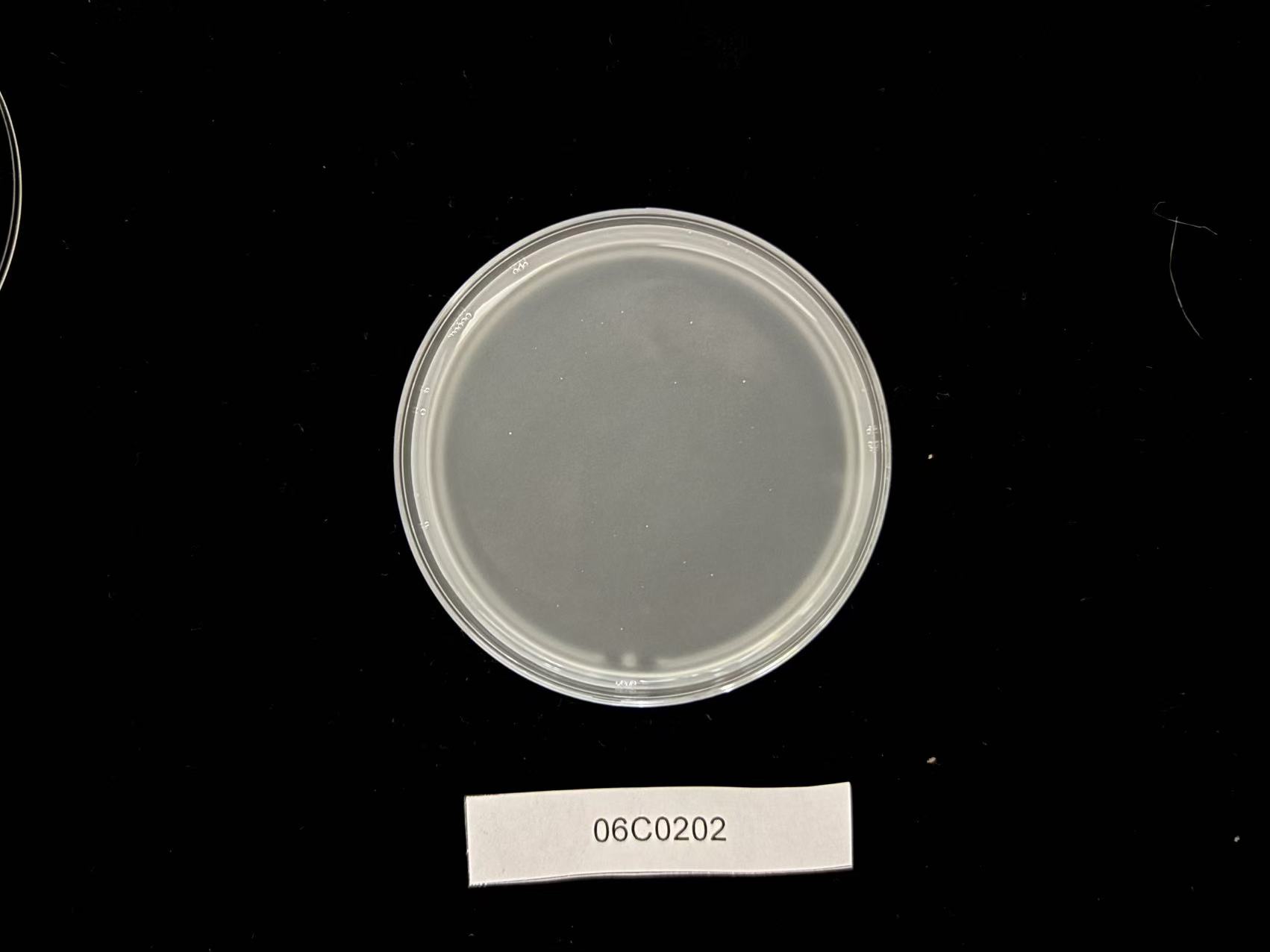

Supplement: Supplementary file 14 — Appendix Figure S7 Source Data [file 44319_2026_748_MOESM14_ESM.zip › Appendix Figure S7/S7B/gsf2Γêå_Repeat2.jpg]

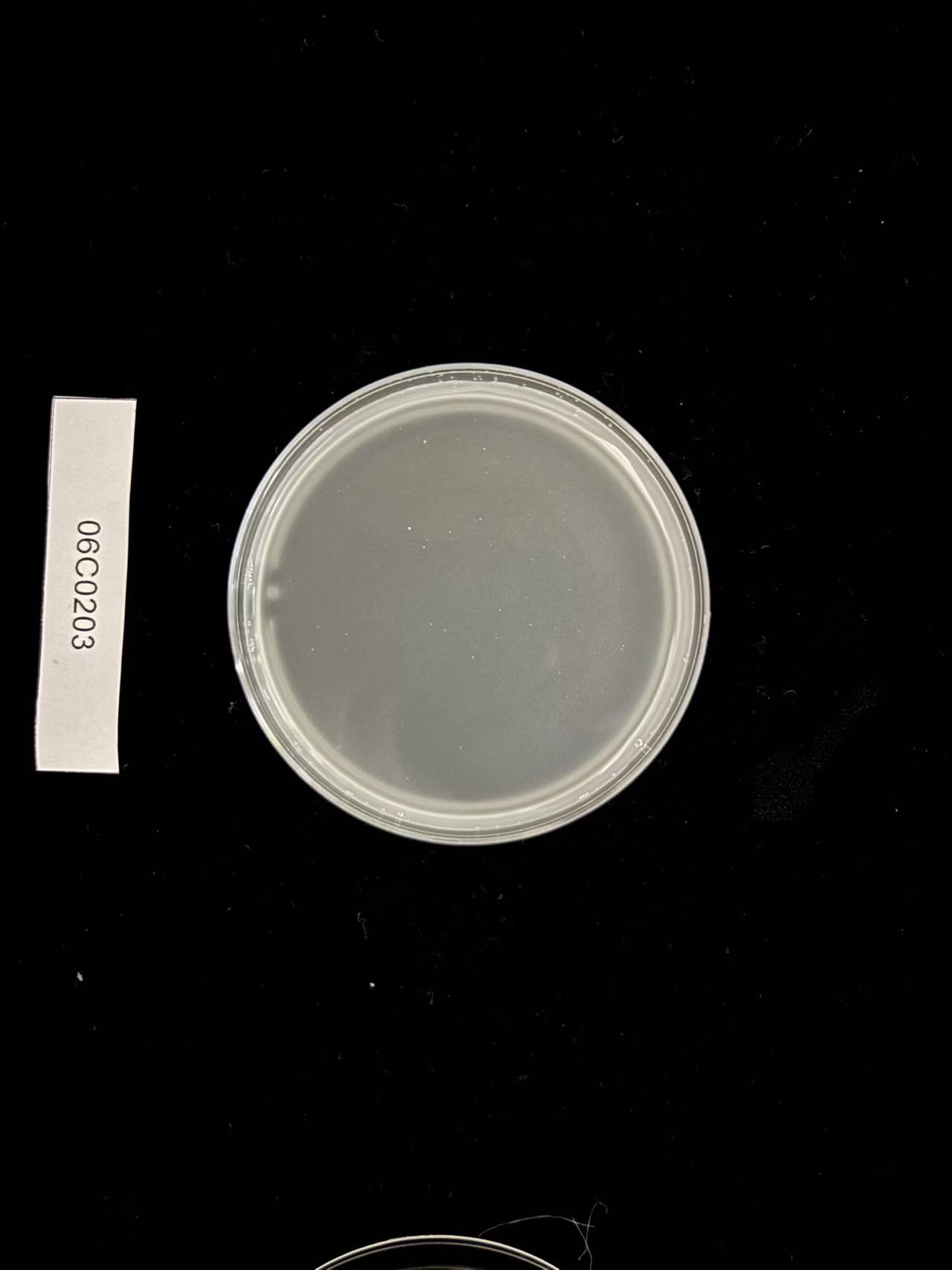

Supplement: Supplementary file 14 — Appendix Figure S7 Source Data [file 44319_2026_748_MOESM14_ESM.zip › Appendix Figure S7/S7B/gsf2Γêå_Repeat3.jpg]

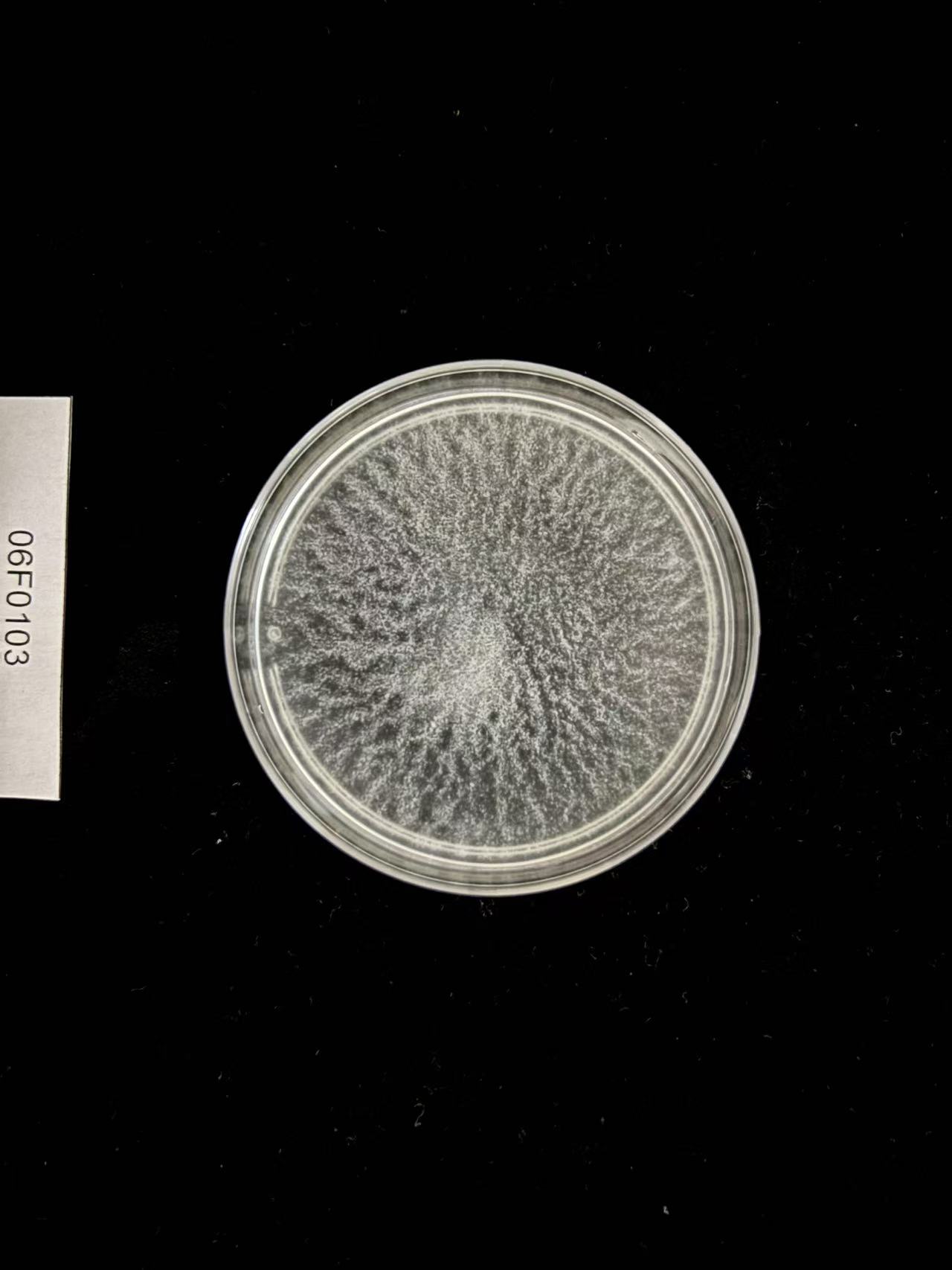

Supplement: Supplementary file 14 — Appendix Figure S7 Source Data [file 44319_2026_748_MOESM14_ESM.zip › Appendix Figure S7/S7A/gsf2AIE_Repeat3.jpg]

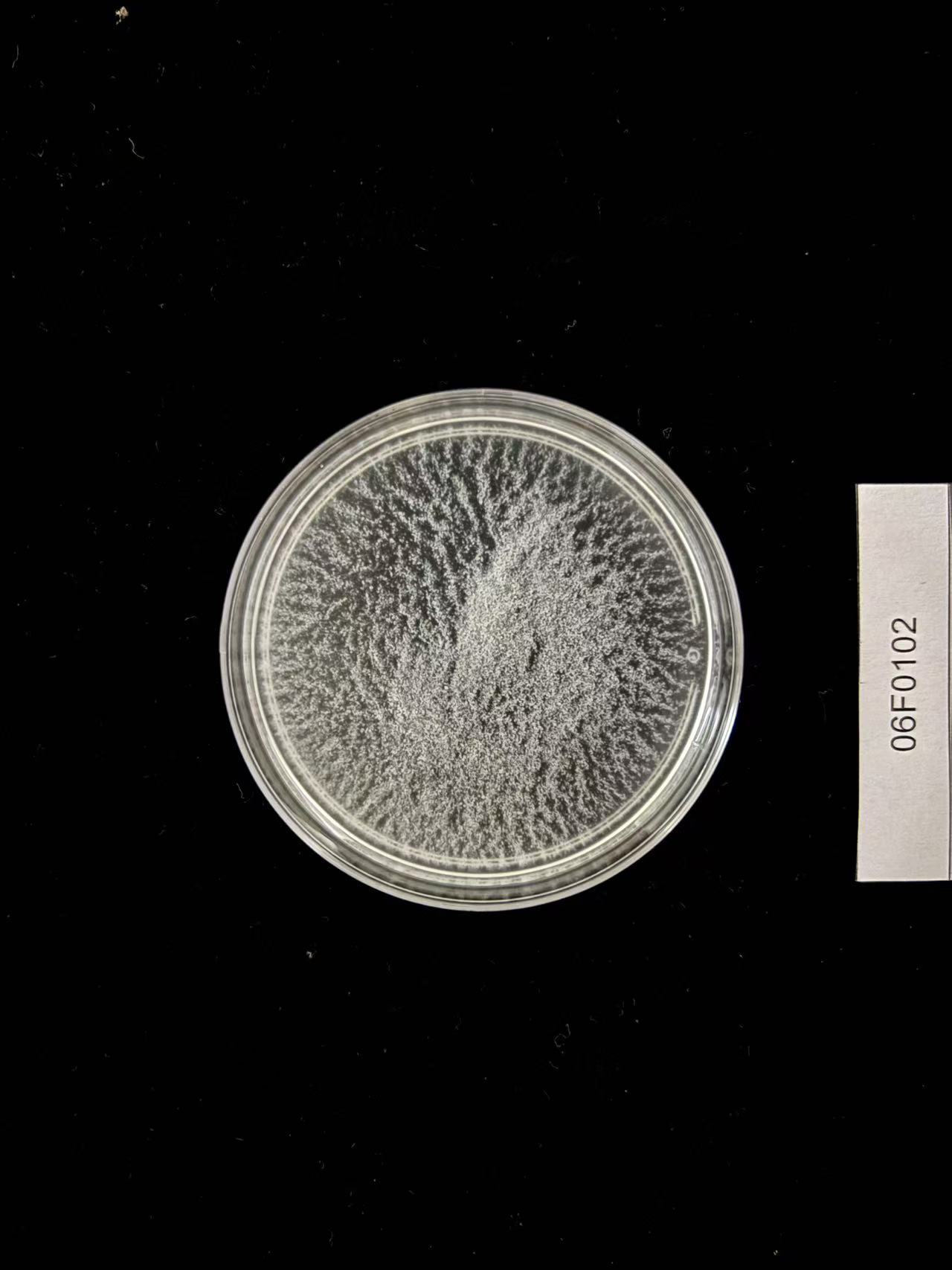

Supplement: Supplementary file 14 — Appendix Figure S7 Source Data [file 44319_2026_748_MOESM14_ESM.zip › Appendix Figure S7/S7A/gsf2AIE_Repeat2.jpg]

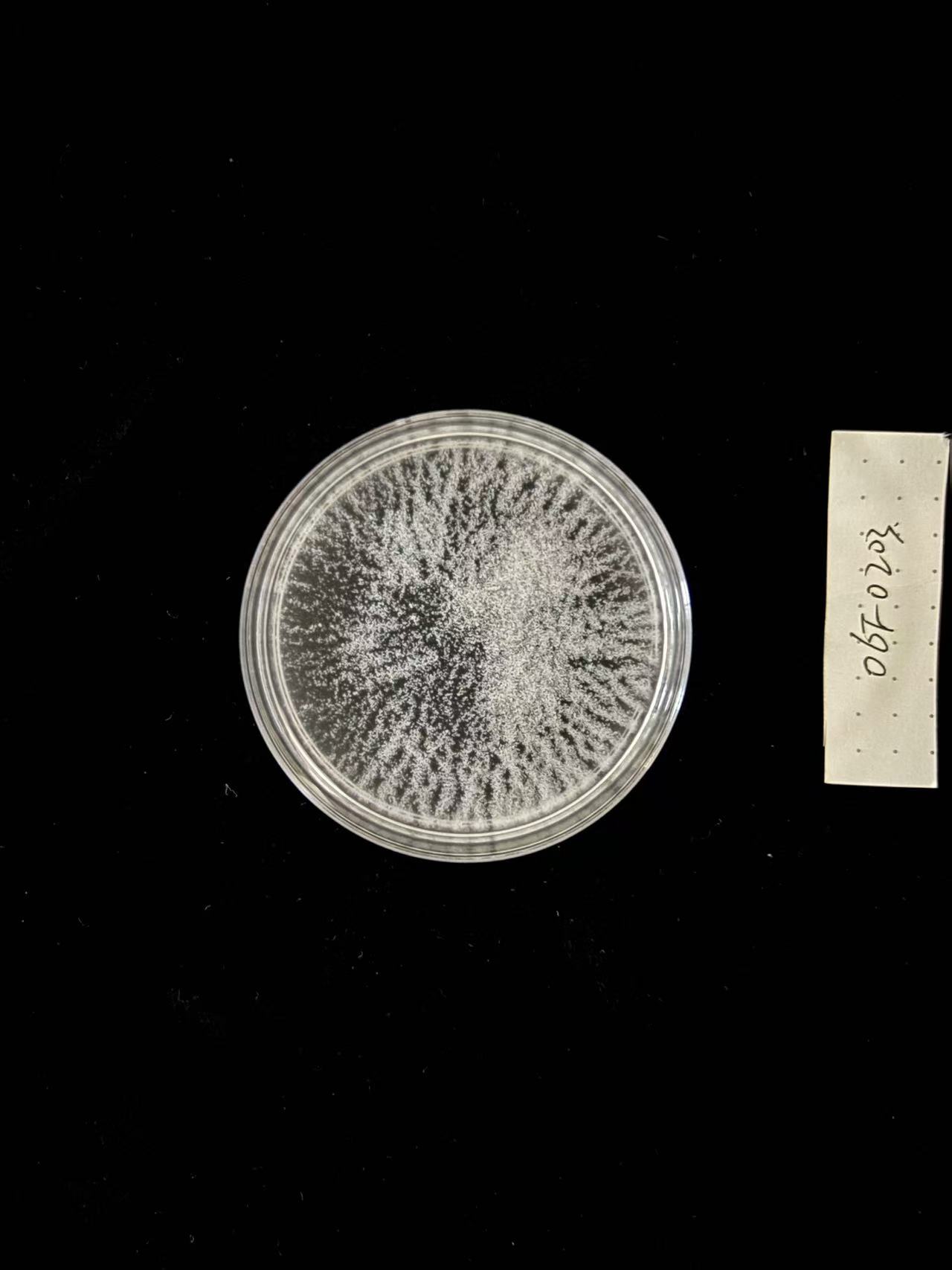

Supplement: Supplementary file 14 — Appendix Figure S7 Source Data [file 44319_2026_748_MOESM14_ESM.zip › Appendix Figure S7/S7A/gsf2IE_Repeat3.jpg]

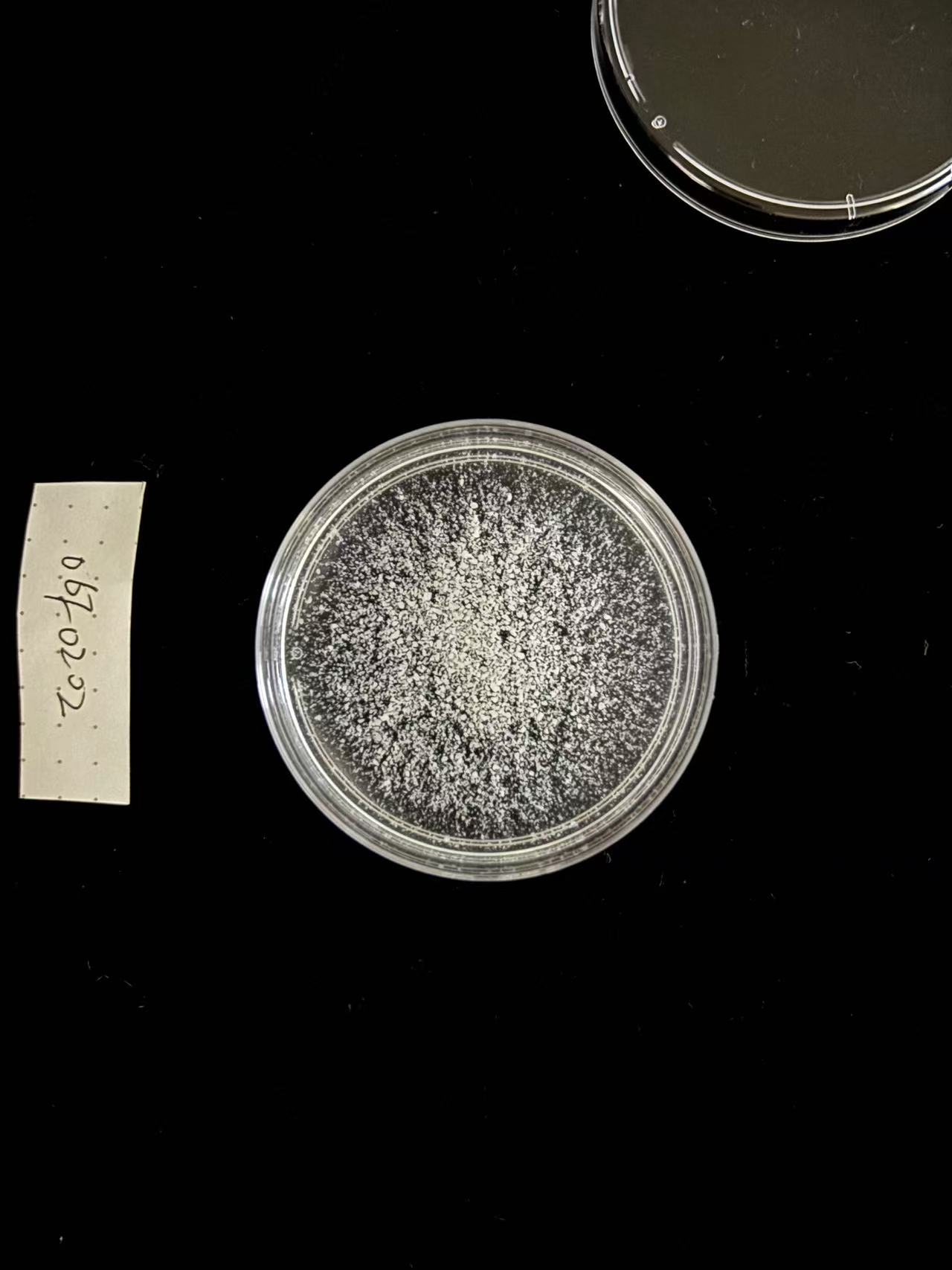

Supplement: Supplementary file 14 — Appendix Figure S7 Source Data [file 44319_2026_748_MOESM14_ESM.zip › Appendix Figure S7/S7A/gsf2IE_Repeat2.jpg]
