# Supplementary material for: A gradient green-beard gene in fission yeast
Source: EMBO Rep. 2026 Mar 16;27(8):1904–17. doi: 10.1038/s44319-026-00748-x (PMC13121626; doi:10.1038/s44319-026-00748-x)
Supplement: Supplementary file 15 — Expanded View Figures [file 44319_2026_748_MOESM15_ESM.pdf]

Expanded View Figures

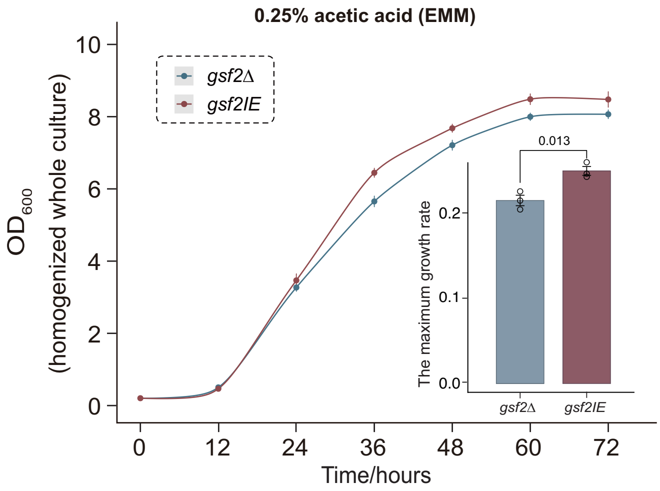

Figure EV1. ***gsf2*** **confers a growth advantage under acetic acid stress.**

Growth curves of *gsf2*Δ and *gsf2IE* strains cultivated in EMM medium supplemented with 0.25% acetic acid, under *gsf2*-inducing conditions. The maximum growth rates for both strains are quantified. Error bars represent mean ± SD of three biological replicates. Statistical significance was determined using a two-tailed Student's t-test. Source data are available online for this figure.

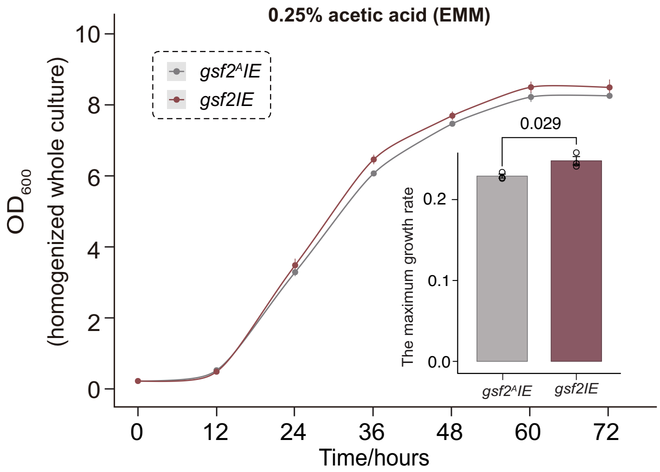

Figure EV2. The growth dynamics of *gsf2^AIE* and *gsf2IE* strains under acetic acid stress.

The maximum growth rates for both strains were also shown. Error bars represent the mean ± SD of three biological replicates. Significance was determined using Student's t-test. Source data are available online for this figure.

 © The Author(s)
